# First-line durvalumab in combination with trastuzumab deruxtecan in women with locally advanced unresectable or metastatic, hormone-receptor-negative, HER2-low breast cancer: multicenter, open-label, phase 1b/2 BEGONIA platform trial

**DOI:** 10.1038/s43018-026-01181-8

**Published:** 2026-06-08

**Authors:** Peter Schmid, Seock-Ah Im, Zbigniew Nowecki, Piotr J. Wysocki, Jacek Jassem, Kyung Hae Jung, Simon Lord, Jon Armstrong, Ross Stewart, Petra Vuković, Neelima Denduluri, Yeon Hee Park

**Affiliations:** 1 Barts Cancer Institute, Queen Mary University of London, London, UK; 2 Seoul National University Hospital, Cancer Research Institute, Seoul National University College of Medicine, Seoul National University, Seoul, Republic of Korea; 3 Maria Skłodowska-Curie National Research Institute of Oncology, Warsaw, Poland; 4 Department of Oncology, Jagiellonian University – Medical College, Krakow, Poland; 5 Medical University of Gdańsk, Gdańsk, Poland; 6 Asan Medical Center, University of Ulsan, College of Medicine, Seoul, Republic of Korea; 7 Medical Sciences Division, University of Oxford, Oxford, UK; 8 AstraZeneca, Cambridge, UK; 9 AstraZeneca, Gaithersburg, MD USA; 10 Samsung Medical Center, Sungkyunkwan University School of Medicine, Seoul, Republic of Korea

**Keywords:** Breast cancer, Drug development, Tumour biomarkers, Cancer

## Abstract

More effective therapies are required for advanced breast cancer. We report results from 58 women with locally advanced unresectable or metastatic hormone-receptor (HR)-negative, human epidermal growth factor receptor 2 (HER2)-low breast cancer enrolled in arm 6 of the multicenter, open-label phase 1b/2 BEGONIA platform trial, who received durvalumab (1,120 mg) plus trastuzumab deruxtecan (T-DXd; 5.4 mg kg^−1^) intravenously every 3 weeks as first-line treatment. Objective response rate (ORR) and safety were primary endpoints; duration of response (DoR), progression-free survival (PFS) and overall survival (OS) were secondary endpoints. Median follow-up was 20.6 months (range: 1–37). ORR was 62.1% (95% confidence interval (CI): 48.4–74.5), which did not meet the protocol-specified objective of 38/57 (66.6%) responses. Median DoR was 15.2 months (95% CI: 8.44–not calculable), PFS was 12.6 months (95% CI: 8.4–16.3) and OS was 30.3 months (95% CI: 18.8–not calculable). The safety profile of the combination treatment was consistent with those of the individual therapies. Adjudicated, drug-related interstitial lung disease or pneumonitis occurred in 20.7% of participants (grades 1 and 2, 19.0%; grade 5, 1.7%). Durvalumab plus T-DXd demonstrated clinically relevant efficacy for first-line treatment of metastatic HR-negative, HER2-low breast cancer, with no unexpected toxicities observed. ClinicalTrials.gov identifier: NCT03742102.

## Abstract (web summary)

In this phase 1/2 BEGONIA platform trial, Schmid and colleagues evaluated the safety and efficacy of durvalumab and trastuzumab deruxtecan in participants with locally advanced unresectable or metastatic hormone-receptor-negative breast cancer with low human epidermal growth factor receptor 2.

## Main

Approximately 15–20% of all breast cancers are diagnosed as triple-negative breast cancers (TNBCs)^1^, defined by the lack of estrogen receptor (ER) and progesterone receptor (PR) expression and the absence of human epidermal growth factor receptor 2 (HER2) overexpression. Compared to other breast cancer subtypes, TNBC typically follows an aggressive disease course, with a high recurrence rate and greater potential for metastases^1^.

Chemotherapy remains the mainstay of first-line treatment for most persons with advanced TNBC. Current recommendations offer other types of therapy on the basis of biomarker status. Around 20–40% of TNBCs are programmed death ligand 1 (PD-L1)-positive^2,3^ and the recommended first-line treatment of PD-L1-positive unresectable locally advanced or metastatic TNBC is a combination of chemotherapy with an immune checkpoint inhibitor^4^. However, treatment outcomes for advanced TNBC remain poor^5^. Disease progression occurs at approximately 6 months for persons with PD-L1-negative disease and up to 10 months for persons with PD-L1-positive disease in first-line treatment of advanced TNBC^6–8^. Median overall survival (OS) for persons with advanced TNBC remains around 2 years^6–9^.

Around one third of TNBCs have been found to be HER2-low, defined as an immunohistochemistry (IHC) analysis score of 1+ or 2+ and lack of *ERBB2* amplification detected by in situ hybridization (ISH) techniques^10^. The emergence of the HER2-low status as a biomarker for treatment has provided the potential for further therapy options in breast cancer^11,12^.

Trastuzumab deruxtecan (T-DXd; formerly DS-8201) is an antibody–drug conjugate composed of a humanized IgG1 monoclonal antibody specifically targeting HER2, a tetrapeptide-based cleavable linker and a potent topoisomerase I inhibitor payload. In addition to delivering the cytotoxic payload (drug-to-antibody ratio of 8:1) directly to HER2-expressing cells, a bystander effect occurs following target cell destruction, where the cytotoxic payload is released into the tumor microenvironment and internalized by neighboring cells, exerting a cytotoxic effect regardless of HER2 expression level^13–15^.

T-DXd has been approved in HER2-positive (IHC 3+ or ISH+), HER2-low and HER2-ultralow (IHC 0 with membrane staining) breast cancers^16–18^. Unlike traditional anti-HER2 therapies, T-DXd has shown efficacy in HER2-low breast cancer and was approved following the phase 3 DESTINY-Breast04 study, which showed significantly improved outcomes in participants with HER2-low breast cancer compared to chemotherapy^17^. In the subgroup of participants with HR-negative disease, median progression-free survival (PFS) was 8.5 versus 2.9 months (hazard ratio (HR) = 0.46; 95% confidence interval (CI): 0.24–0.89), median OS was 18.2 versus 8.3 months (HR = 0.48; 95% CI: 0.24–0.95) and confirmed objective response was 50.0% (95% CI: 33.8–66.2) versus 16.7% (95% CI: 3.6–41.4) for T-DXd compared to chemotherapy.

The combination of T-DXd with an immune checkpoint inhibitor has the potential to further improve outcomes in metastatic breast cancer, including HR-negative, HER2-low disease. In preclinical studies, T-DXd treatment increased tumor-infiltrating CD8 T cells and tumor PD-L1 expression in murine models, suggesting potential priming of antitumor immunity^19^. Such priming may be enhanced by subsequent anti-PD(L)1 treatment and, in keeping with this, T-DXd antitumor activity was improved in murine tumor models with the addition of an anti-PD1 antibody^19^. Durvalumab is a selective, high-affinity human IgG1ƙ monoclonal antibody that binds PD-L1 and blocks PD-L1 from binding to PD1 and CD80, resulting in T cell activation. Durvalumab is approved as monotherapy and in combinations across several solid tumor types^20^ and has shown promise in TNBC^21,22^.

BEGONIA (NCT03742102) is an ongoing multicenter, multiarm platform study evaluating the safety and efficacy of durvalumab with or without paclitaxel, in combination with novel oncology therapies as first-line treatment for locally advanced unresectable or metastatic breast cancer that would be considered TNBC in clinical practice. Here, we report results of the combination of durvalumab and T-DXd from arm 6 of BEGONIA, which specifically enrolled participants with locally advanced unresectable or metastatic HR-negative, HER2-low breast cancer.

## Results

### Participants

**Fig. 1:**
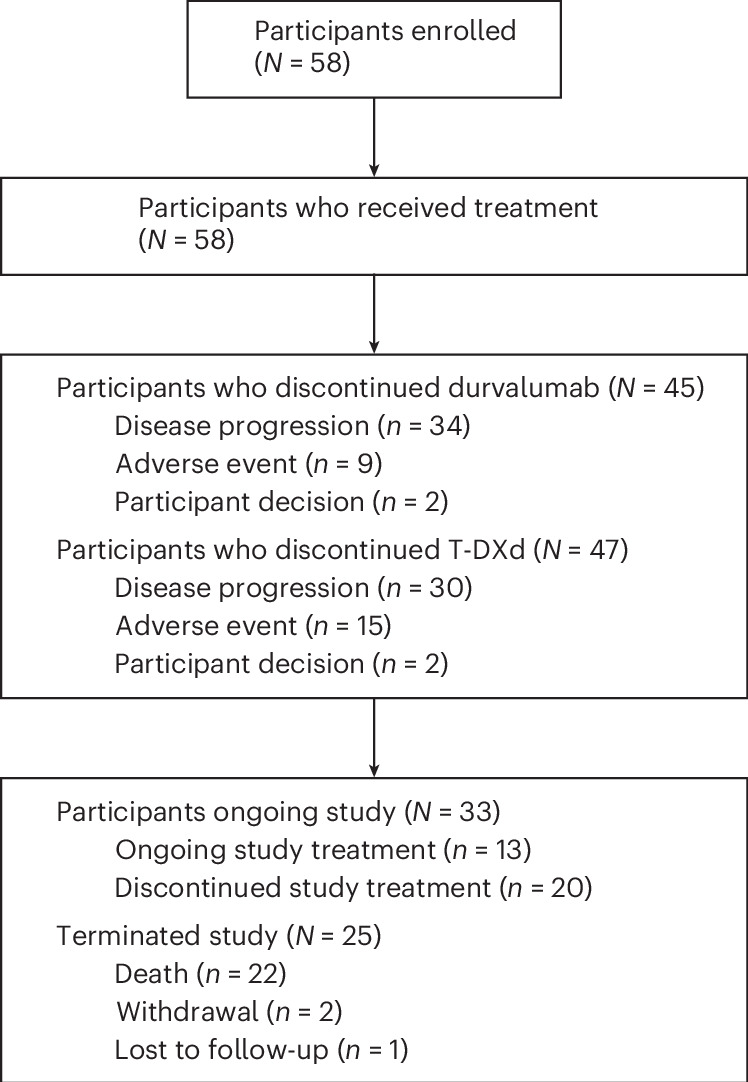
Participant disposition for durvalumab plus T-DXd arm of BEGONIA. All values reported are the number of participants.

Between May 28, 2020 and March 28, 2022, 58 participants with HR-negative, HER2-low breast cancer were enrolled in the durvalumab plus T‑DXd arm (arm 6) during part 1 and part 2 of the BEGONIA study (Fig. 1). All enrolled participants received study treatment. A total of 33 participants were ongoing in the study at data cutoff (June 30, 2023), including 13 who remained on study treatment.

The median age of participants was 54 years; 67.2% of participants were White, 27.6% were Asian and 3.4% were Black or African American. Around one quarter of participants did not receive any prior treatment for breast cancer (all de novo metastatic). Most participants (79.3%) had a PD-L1-negative tumor status (tumor area positivity (TAP) score < 10%) as assessed using the VENTANA PD-L1 (SP263) assay. Additional participant and disease characteristics are shown in Table 1.

**Table 1:** Baseline participant demographics and disease characteristics

Parameter	Durvalumab + T-DXd (*N* = 58)
**Median age (range), years**	54 (29–81)
**Age group,** ***n*** **(%), years**	
<65	47 (81.0)
≥65–<75	9 (15.5)
≥75	2 (3.4)
**Race,** ***n*** **(%)**	
White	39 (67.2)
Asian	16 (27.6)
Black or African American	2 (3.4)
Other	1 (1.7)
**No prior treatment for breast cancer,** ***n*** **(%)**^**a**^	16 (27.6)
**Treatment for prior breast cancer,** ***n*** **(%)**	
Chemotherapy	37 (63.8)
Anthracycline and related substances^b^	37 (63.8)
Cyclophosphamide	36 (62.1)
Taxanes	32 (55.2)
Radiotherapy	35 (60.3)
Hormonal therapy	15 (25.9)
Targeted therapy^c^	2 (3.4)
**Treatment-free interval,** ***n*** **(%)**^**d**^	
De novo metastatic	17 (29.3)
<12 months	10 (17.2)
≥12 months	31 (53.4)
**Number of metastatic sites,** ***n*** **(%)**	
1–2	36 (62.1)
≥3	22 (37.9)
**Visceral metastases,** ***n*** **(%)**^**e**^	40 (69.0)
**Eastern Cooperative Oncology Group performance status,** ***n*** **(%)**	
0	30 (51.7)
1	28 (48.3)
**PD-L1 expression,** ***n*** **(%)**^**f**^	
PD-L1 TAP ≥ 10% (positive)	7 (12.1)
PD-L1 TAP < 10% (negative)	46 (79.3)
Missing score	5 (8.6)
**Locally assessed HER2 status,** ***n*** **(%)**	
1+/ISH untested	34 (58.6)
1+/ISH^−^	4 (6.9)
2+/ISH^−^	20 (34.5)

^a^Participants had to have no prior treatment for metastatic (stage IV) TNBC. Prior treatment with curative intent for stage I–III TNBC (that subsequently became metastatic) was acceptable provided ≥6 months had elapsed between completion of treatment and the first documented distant disease recurrence.

^b^Included doxorubicin and epirubicin.

^c^HER2 targeted therapy (*n* = 1) or CDK4/6 inhibitors (*n* = 1).

^d^Treatment-free interval was defined as the time from breast cancer surgery or from completion of prior anticancer therapy to the date of disease progression.

^e^Visceral metastases was defined as liver, hepatic and/or respiratory metastases.

^f^Assessed centrally and retrospectively by IHC using the VENTANA PD-L1 (SP263) assay (Roche Diagnostics). Expression was defined as the percentage of the tumor area populated by tumor cells with membranous PD-L1 staining or immune cells with membranous, cytoplasmic or punctate PD-L1 staining at any intensity (TAP score). A TAP score ≥10% was considered positive, whereas a TAP score <10% was considered negative.

The median duration of follow-up was 20.6 months (range: 1–37). The median number of doses was 14.5 (range: 1–43) for durvalumab and 13.0 (range: 1–43) for T-DXd. Median treatment duration was 11 months (range: 1–32) for durvalumab and 10 months (range: 1–32) for T-DXd. Dose delays occurred in 29 participants (50.0%) and 28 participants (48.3%) for durvalumab and T-DXd, respectively. Infusion interruptions occurred in one participant each (1.7%) for durvalumab and T-DXd. Dose reductions were not permitted for durvalumab but were reported for T-DXd in nine participants (15.5%). A total of 45 participants (77.6%) discontinued durvalumab; the most common reason for discontinuation was disease progression in 34 participants (58.5%). A total of 47 participants (81.0%) discontinued T-DXd treatment; the most common reason for discontinuation was disease progression in 30 participants (51.7%).

Among 45 participants who had discontinued study treatment, 30 (66.7%) received subsequent anticancer therapy. Subsequent therapy was initiated after progression in 25 participants (55.6%) and before progression in four participants (8.9%); there was no progression in one participant (2.2%).

### Antitumor efficacy and survival

**Fig. 2:**
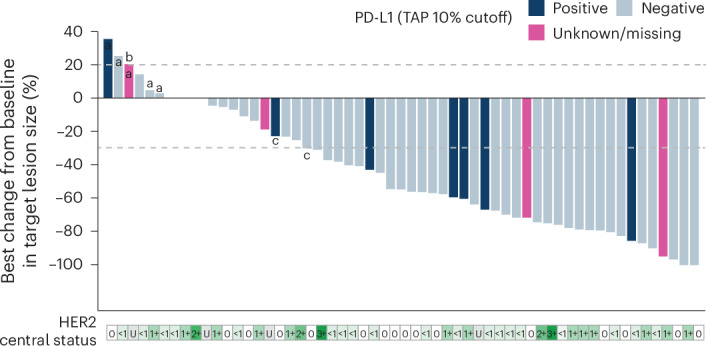
Best change from baseline in target lesion size. Best change in target lesion size is the maximum reduction from baseline or the minimum increase from baseline in the absence of reduction. Dotted lines at −30% and 20% indicate the threshold for partial response and progressive disease, respectively. PD-L1 expression was assessed using the VENTANA PD-L1 (SP263) assay (Roche Diagnostics). Expression was defined as the percentage of the tumor area populated by tumor cells with membranous PD-L1 staining or immune cells with membranous, cytoplasmic or punctate PD-L1 staining at any intensity (TAP score). A TAP score ≥10% was considered positive, whereas a TAP score <10% was considered negative. Central HER2 IHC testing was not possible for four participants because of test failure related to the provided sample having no or insufficient tumor present (*n* = 3) or because of no sample being provided (*n* = 1). ^a^Participants with progressive disease as best overall response. ^b^If the best percentage change from baseline of target lesions could not be calculated because of progression, withdrawal or death, the value was imputed at +20%. ^c^Unconfirmed response. U, central HER2 status unknown; 0, central HER2 IHC 0 absent membrane staining; <1, central HER2 IHC 0 with membrane staining; 1+, central HER2 IHC 1+; 2+, central HER2 IHC 2+; 3+, central HER2 IHC 3+. Source data

All 58 participants were evaluable for efficacy, having measurable disease at baseline and having completed at least two on-treatment disease assessments. The confirmed objective response rate (ORR) was 62.1% (36/58; 95% CI: 48.4–74.5), which did not meet the protocol-specified objective of 38/57 (66.6%) responses. One participant (1.7%) achieved a complete response and 35 participants (60.3%) achieved partial responses (Fig. 2). The unconfirmed ORR was 65.5% (38/58; 95% CI: 51.9–77.5). ORR in participants with PD-L1-positive disease and PD-L1-negative disease was 57.1% (4/7) and 65.2% (30/46), respectively. The median duration of response (DoR) was 15.2 months (95% CI: 8.44–not calculable), with 47.2% (17/36) of responses ongoing at data cutoff. The proportions of participants remaining in response at 6 and 12 months were 83.3% and 63.6%, respectively.

**Fig. 3:**
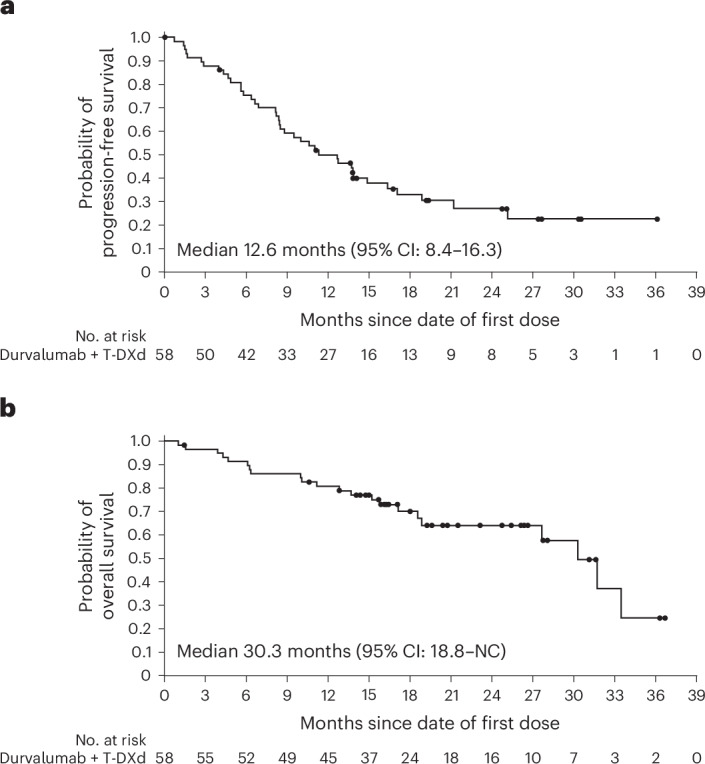
Kaplan–Meier plots for PFS and OS in the intent-to-treat population. **a**,**b**, All 58 participants who received treatment were included in the Kaplan–Meier analyses of PFS (**a**) and OS (**b**). PFS was measured from the date of first dose of study drug until the date of progression or death. OS was measured from the date of first dose of study drug until the date of death. Circles indicate censored observations. NC, not calculable. Source data

As of the data cutoff, 39 PFS events occurred (67.2%; 33 RECIST progression events and six deaths in absence of progression) and the median PFS was 12.6 months (95% CI, 8.4–16.3; Fig. 3a). The PFS rates were 75.2% (95% CI: 61.8–84.5) at 6 months, 50.1% (95% CI: 36.4–62.3) at 12 months, 33.0% (95% CI: 20.5–46.0) at 18 months and 27.0% (95% CI: 15.0–40.5) at 24 months. At the time of data cutoff, 22 participants (37.9%) had died and 36 participants (62%) were censored, among which 33 (57%) were still in survival follow-up. The median OS was 30.3 months (95% CI: 18.8–not calculable; Fig. 3b). The OS rates were 91.3% (95% CI: 80.3–96.3) at 6 months, 80.7% (95% CI: 67.8–88.8) at 12 months, 70.0% (95% CI: 55.4–80.6) at 18 months and 64.1% (95% CI: 48.5–76.1) at 24 months.

**Fig. 4:**
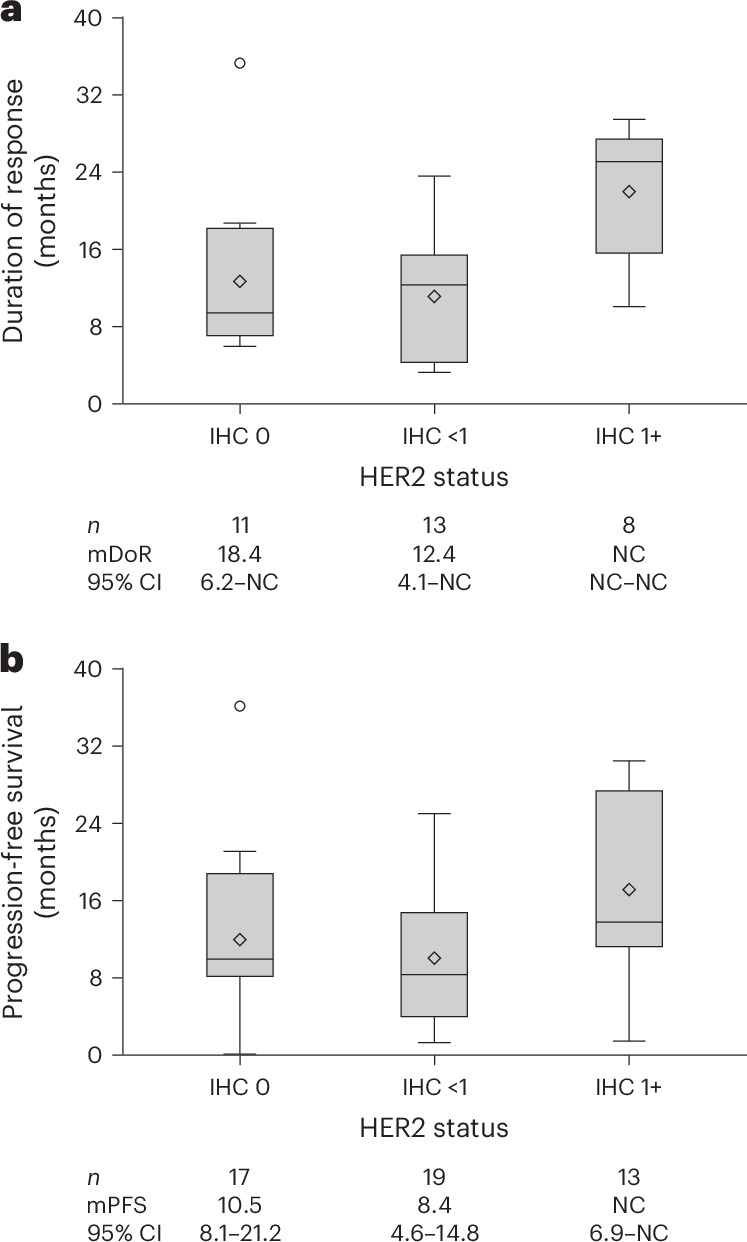
Efficacy outcomes by centrally assessed HER2 status. **a**, Analysis of DoR included the 36 participants who had an objective response. **b**, Analysis of the duration of PFS included all 58 participants who received treatment. DoR was measured from the date of first documented confirmed response to the date of progression or death. PFS was measured from the date of first dose of study drug until the date of progression or death. Participants were stratified on the basis of central HER2 testing. DoR and PFS box plots were based on descriptive analysis. Data for categories with fewer than five participants are not shown: **a**, IHC 2+ (*n* = 1), IHC 3+ (*n* = 2) and missing (*n* = 1); **b**, IHC 2+ (*n* = 3), IHC 3+ (*n* = 2) and missing (*n* = 4). The medians provided below the figures were calculated using the Kaplan–Meier method. The center horizontal lines represent the median (calculated from descriptive statistics in each group), the box represents the 25th percentile to the 75th percentile values and the diamonds represent the mean. Circles represent outlier data points. The whiskers show the most extreme observation (maxima and minima) within 1× the interquartile range from the nearest quartile, whereby all outliers >1× the interquartile range are individually displayed. Here, *n* is the number of participants included in the analysis. HER2 IHC 0, central HER2 IHC 0 absent membrane staining; HER2 IHC <1, central HER2 IHC 0 with membrane staining (ultralow); mDoR, median DoR; mPFS, median PFS. Source data

**Extended Data Fig. 1:**
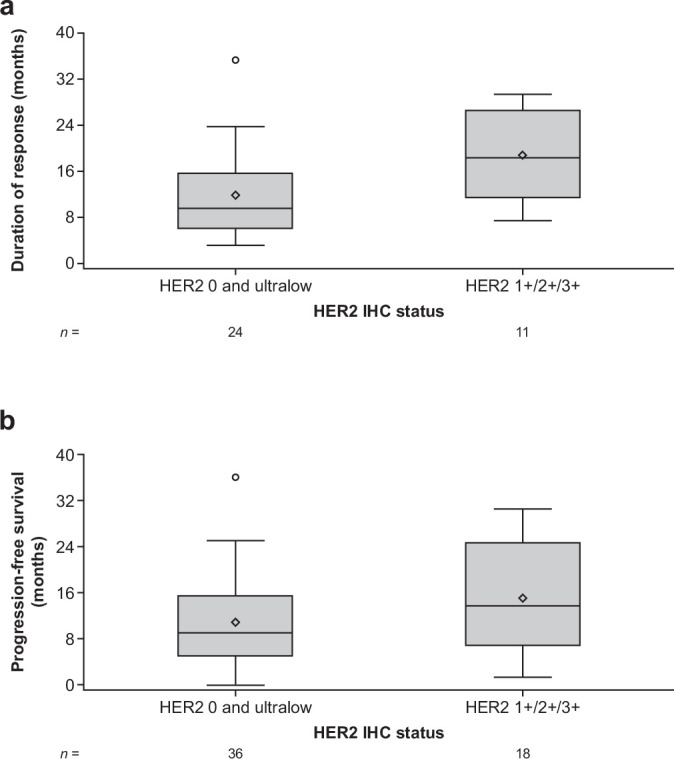
Efficacy outcomes by pooled centrally-assessed HER2 status. **a**, Median duration of response. **b**, Median progression-free survival. The center horizontal lines represent the median (calculated from descriptive statistics in each group), the box represents the 25th percentile to the 75th percentile values, the diamonds represent the mean, and the circles represent outlier data. The whiskers show the most extreme observation (minima and maxima) within 1× the interquartile range from the nearest quartile, so that all outliers >1× the interquartile range are individually displayed. HER2 0, central HER2 IHC 0 absent membrane staining. *n* is the number of patients included in the analysis. The medians provided below the figures were calculated using the Kaplan-Meier method. HER2 ultralow, central HER2 IHC 0 with membrane staining. HER2, human epidermal growth factor receptor 2; IHC, immunohistochemistry. Source data

While participant entry into the durvalumab plus T-DXd study arm was based on locally assessed HER2-low status (HER2 IHC score of 1+ or 2+ and ISH^−^ or untested), baseline tumor samples were also assessed centrally for HER2 expression in an exploratory analysis. Central HER2 IHC 0 absent membrane staining was reported in 17 participants (29.3%) and central HER2 IHC 0 with membrane staining (HER2-ultralow) was reported in 19 participants (32.8%). Central HER2 IHC 1+, 2+ and 3+ scores were obtained from 13 (22.4%), 3 (5.2%) and 2 (3.4%) participants, respectively. Central HER2 IHC testing was not possible for four participants. Responses were observed regardless of central HER2 IHC score, including HER2-ultralow and IHC 0 absent membrane staining (Fig. 2). Median DoR and median PFS for central HER2 IHC score groups are shown in Fig. 4a,b, respectively. These outcomes by pooled scores for HER2-ultralow and IHC 0 absent membrane staining versus HER2 IHC 1+, 2+ and 3+ groups are shown in Extended Data Fig. 1. The ORR, median DoR, median PFS and median OS per local HER2 IHC score are shown in Supplementary Table 1.

### Safety

All 58 participants were included in the safety analysis set. During the safety run-in, there were no dose-limiting toxicities. During the overall study, 98.3% of participants experienced adverse events (AEs), with AEs of a maximum grade of 3 or 4 reported in 48.3% of participants (Table 2). The most common AEs of any grade were nausea (79.3%), fatigue (53.4%), constipation (34.5%), neutropenia (32.8%) and vomiting (31.0%) (Table 3). The most common grade 3 or 4 AEs were neutropenia (22.4%), anemia (10.3%) and thrombocytopenia (6.9%). No cases of febrile neutropenia were reported.

**Table 2:** Summary of AEs

Category, *n* (%)	Durvalumab + T-DXd (*N* = 58)
**Any AE**	57 (98.3)
Any AE possibly related to treatment	56 (96.6)
Any AE possibly related to durvalumab only	29 (50.0)
Any AE possibly related to T-DXd only	47 (81.0)
**Any AE of maximum CTCAE grade 3 or 4**	28 (48.3)
**Any AE leading to death**	2 (3.4)
**Any serious AE**^**a**^	16 (27.6)
**Any AE leading to discontinuation of durvalumab**	9 (15.5)
**Any AE leading to discontinuation of T-DXd**	15 (25.9)
**Any AE leading to dose interruption (either treatment)**	35 (60.3)
**Any AE leading to dose reduction of T-DXd**	8 (13.8)
**Any immune-mediated AE**^**b**^	20 (34.5)
**Any immune-mediated AE**^**b**^ **of maximum CTCAE grade 3 or 4**	3 (5.2)

^a^Including events leading to death. ^b^Immune-mediated AEs were defined as AEs of special interest consistent with an immune-mediated mechanism of action with no clear alternate etiology and that required the use of systemic corticosteroids, other immunosuppressants or endocrine therapy to manage the AE.

CTCAE, Common Terminology Criteria for AEs.

**Table 3:** Most common AEs (≥20%)

	Durvalumab + T-DXd (*N* = 58)	
AE by preferred term, *n* (%)	Any grade	Maximum grade 3 or 4
**Nausea**	46 (79.3)	1 (1.7)
**Fatigue**	31 (53.4)	2 (3.4)
**Constipation**	20 (34.5)	0
**Neutropenia**	19 (32.8)	13 (22.4)
**Vomiting**	18 (31.0)	1 (1.7)
**Anemia**	16 (27.6)	6 (10.3)
**Alopecia**	16 (27.6)	0
**Hypothyroidism**	15 (25.9)	0
**Decreased appetite**	15 (25.9)	1 (1.7)
**Diarrhea**	13 (22.4)	0
**COVID-19**	13 (22.4)	2 (3.4)
**ALT increased**	13 (22.4)	0
**AST increased**	12 (20.7)	0
**Asthenia**	12 (20.7)	2 (3.4)

AEs were reported per the National Cancer Institute Common Terminology Criteria for AEs grading scale.

Participants with multiple AEs are counted once for each AE preferred term. Reported AEs include AEs with an onset date on or after date of first dose, AEs with onset date before dosing that worsened after dosing and AEs occurring up to 90 days following date of last dose or up to first subsequent therapy.

ALT, alanine aminotransferase; AST, aspartate aminotransferase.

AEs possibly related (as per the investigator) to any study treatment were reported in 96.6% of participants (Table 2). AEs of any grade related to durvalumab were reported in 84.5% of participants, the most common of which were fatigue (27.6%), nausea (24.1%) and hypothyroidism (20.7%) (Supplementary Table 2). AEs of any grade related to T-DXd occurred in 96.6% of participants, the most common of which were nausea (70.7%), fatigue (48.3%) and neutropenia (32.8%) (Supplementary Table 3).

A total of 16 participants (27.6%) reported a serious AE (Table 2). Serious AEs possibly related to study drug were observed in 11 participants (19.0%; related to durvalumab: *n* = 10, related to T-DXd: *n* = 9). AEs leading to discontinuation of durvalumab were reported in 15.5% of participants and for discontinuation of T-DXd in 25.9% (Table 2). Pneumonitis was the most common AE that led to discontinuation of durvalumab (8.6%; *n* = 5) and T-DXd (10.3%; *n* = 6), with discontinuation of both study drugs occurring in four participants (6.8%).

AEs of special interest to durvalumab were those known to be associated with immune checkpoint inhibitors and infusion or hypersensitivity reactions^23^. The most common AEs of special interest to durvalumab were hypothyroidism (25.9%; *n* = 15), diarrhea or colitis (22.4%; *n* = 13) and dermatitis or rash (19.0%; *n* = 11). Immune-mediated AEs of any grade were reported in 34.5% of participants and those with a maximum grade of 3 or 4 were reported in 5.2% of participants (Table 2). Individual immune-mediated AEs were hypothyroid events (20.7%; *n* = 12), pneumonitis (17.2%; *n* = 10), hyperthyroid events (5.2%; *n* = 3), hypophysitis and pancreatic events (1.7% each; *n* = 1). There were no fatal immune-mediated AEs. Infusion or hypersensitivity reactions were reported in three participants; two had urticaria and one had an infusion-related reaction.

AEs of special interest to T-DXd were interstitial lung disease (ILD) or pneumonitis and events of left-ventricular dysfunction. All cases of investigator-assessed ILD, pneumonitis and other related AEs were assessed by an independent adjudication committee (Methods). Cases confirmed by the adjudication committee as treatment-related ILD or pneumonitis occurred in 12 participants (20.7%). Most cases of adjudicated drug-related ILD events were of low grade (grade 1, *n* = 1, 1.7%; grade 2, *n* = 10, 17.2%), with no grade 3 or 4 events. There was one grade 5 adjudicated drug-related ILD event (1.7%), reported by the investigator as pneumonitis related to COVID-19 that occurred following a grade 3 COVID-19 infection. Left-ventricular dysfunction events were reported in four participants (6.9%): decreased ejection fraction in two participants (3.4%, both grade 2), increased troponin in two participants (3.4%, both grade 2) and one case of grade 5 cardiac failure (1.7%).

Two participants (3.4%) experienced an AE leading to death; one case was cardiac failure caused by myocardial infarction, not related to either study drug, whereas the second was the aforementioned grade 5 event of pneumonitis related to COVID-19 per the investigator, which was attributed to both study treatments.

## Discussion

The durvalumab and T-DXd combination from arm 6 of BEGONIA conferred clinically relevant efficacy with no unexpected toxicities observed in participants with locally advanced unresectable or metastatic, HR-negative, HER2-low breast cancer. At a median duration of follow-up of 20.6 months, we report an ORR of 62.1%, median DoR of 15.2 months, median PFS of 12.6 months and median OS of 30.3 months in a predominantly PD-L1-negative participant population. The OS analysis remains immature, with 62% of participants being censored. Responses were observed in both PD-L1-positive and PD-L1-negative tumors. While the study did not meet the protocol-defined threshold for adequate efficacy signal (ORR of 66.6%), the overall efficacy outcome remains clinically relevant.

Although cross-trial comparisons may be confounded, our results are consistent with the class effect of immune checkpoint inhibitors observed in phase 3 studies in advanced or metastatic TNBC^6–8^. For example, in the immune checkpoint inhibitor plus chemotherapy arms from KEYNOTE-355 and IMpassion130, the respective efficacy results in the intent-to-treat populations were an ORR of 40.8% and 56.0%, median PFS of 7.5 and 7.2 months and median OS of 17.2 and 21.0 months, respectively^6,7,24,25^. For both KEYNOTE-355 and IMpassion130, the benefit of immunotherapy plus chemotherapy was greater in the PD-L1-positive population, with an ORR of 52.7% and 58.9%, median PFS of 9.7 and 7.5 months and median OS of 23.0 and 25.0 months, respectively^6,7^. In the predominantly PD-L1-negative population of BEGONIA arm 6, responses to durvalumab plus T-DXd were not related to PD-L1 status. While the prevalence of PD-L1-positive disease was lower in our study than other trials of advanced breast cancer^6–8^, differences may be related to the availability of immune checkpoint inhibitors plus chemotherapy as treatment for persons with PD-L1-positive disease, thus impacting recruitment into this BEGONIA arm. Differences could also be derived from the use of different PD-L1 testing assays used in BEGONIA compared to KEYNOTE-355 and IMpassion130 (refs. ^6,7^). Our small sample size may also have contributed to the low prevalence of PD-L1-positive disease in this study.

Participants in BEGONIA arm 6 were enrolled on the basis of HER2-low status from local IHC testing. However, subsequent central testing of HER2 expression from tumor samples identified a substantial discrepancy in HER2 status, with 62% of participants classified centrally as HER2 IHC 0 (with or absent membrane staining) despite being classed as local HER2 IHC 1+ to 2+. There are several plausible reasons for this. First, it is possible that some participants had tumors with intratumoral HER2 heterogeneity, defined as the coexistence of at least two distinct cell clones with varying HER2 statuses within the same tumor—a phenomenon observed in up to 40% of HER2-positive breast cancers^26^. Furthermore, some differences could be related to interobserver variability. Studies using the American Society of Clinical Oncology and College of American Pathologists guidelines have reported an HER2 IHC interobserver variability of 20–30% for HER2 IHC 0 versus 1+ scoring^27–29^. In our study, the assay used for local testing was not restricted; therefore, interassay variability could also have contributed.

On the basis of central HER2 analysis, our study found that responses were observed across HER2 expression levels, even in participants with central HER2 IHC status of ultralow and 0 absent membrane staining. However, results were inconsistent in the HER2 IHC 2+ subgroup, likely because of the small number of participants. DoR and PFS were also not clearly associated with central HER2 status, although a possible trend was observed for longer DoR and PFS in HER2 IHC 1+ versus 0 absent membrane staining and ultralow groups. Other clinical trials have shown activity of T-DXd monotherapy across the HER2 spectrum^30,31^. The phase 2 DAISY trial investigated T-DXd monotherapy in participants with metastatic breast cancer with variable HER2 expression levels but did not investigate HER2 IHC 0 status by membrane staining^30^. While antitumor efficacy was highest in participants with HER2 IHC 3+ tumors, modest efficacy was also observed in the small cohort of 37 participants with HER2 IHC 0 tumors. The phase 3 DESTINY-Breast06 trial recruited participants with HER2-low and HER2-ultralow, HR-positive metastatic breast cancer following one or more lines of endocrine therapy^31^. T-DXd showed a statistically significant and clinically meaningful PFS benefit versus chemotherapy in participants with HER2-low disease (13.2 versus 8.1 months; HR = 0.62; 95% CI: 0.52–0.75, *P* < 0.001). Consistent results were observed in the smaller HER2-ultralow subgroup (13.2 versus 8.3 months; HR = 0.78, 95% CI: 0.50–1.21). Together, these results suggest that persons may still benefit from T-DXd therapy with an HER2-ultralow or IHC 0 absent membrane staining status. It is possible that heterogeneity in tumor HER2 expression may contribute to efficacy of T-DXd in persons whose samples were classed as HER2 IHC 0 absent membrane staining. Development of digital pathology and artificial-intelligence-directed solutions to interobserver variance and heterogenic HER2 expression may aid pathologists in more accurately assessing HER2-low status, thereby identifying persons who would derive greater benefit from T-DXd^32,33^. In the setting of HER2-ultralow breast cancer, while T-DXd therapy has been approved in HR-positive disease, data are still limited in HR-negative disease, with no current approval in this setting.

In general, the safety profile for durvalumab plus T-DXd was consistent with that of the individual agents in breast cancer for T-DXd and across indications for durvalumab^17,23^. Hematologic AEs were the most common grade 3 or 4 toxicities and thyroid disorders were the most common immune-mediated AE. Most cases of adjudicated drug-related ILD or pneumonitis were of low grade and the overall incidence of 20.7% was slightly higher compared to previous T-DXd monotherapy studies, which reported incidences of 10–15% (refs. ^17,18,31,34,35^). The rate of grade 3 or higher adjudicated drug-related ILD or pneumonitis was not increased, with 1.7% reported in our study compared to 0.8–2.7% reported in previous T-DXd monotherapy studies^12,17,31,34,35^. ILD or pneumonitis is an overlapping toxicity for both T-DXd and durvalumab; therefore, a slight increase was expected. Immune-mediated pneumonitis was reported at a rate of 2.5% overall and up to 5% in lung studies in a pooled durvalumab monotherapy dataset^23^. These results support the need for continued monitoring and prompt management of suspected ILD and pneumonitis. Guidelines for proactive surveillance of symptoms, as well as imaging and management of ILD and pneumonitis, were provided in the trial protocol and included guidelines for dose interruptions, reductions or discontinuations and early initiation of glucocorticoid treatment to manage the risk and minimize serious outcomes. The fatal adjudicated drug-related ILD event was reported by the investigator as pneumonitis related to COVID-19 and it occurred following grade 3 COVID-19 infection. Notably, the study was conducted during the COVID-19 pandemic, with 13 cases (22.4%) of COVID-19 infection recorded and it is possible that this confounded the safety results.

Limitations of the analysis include a relatively small participant population and inability to assess the contribution of each therapy. Additionally, the predominance of White participants and the low representation of Black or African American participants may limit the diversity of the study population and reduce the generalizability of the findings. Moreover, the inclusion of participants who received subsequent therapy before progression in the efficacy analyses may have influenced the interpretation of survival and DoR outcomes. As these participants were not censored at the time of subsequent treatment, the results should be interpreted with caution. The exploratory biomarker analysis was further limited by the small number of participants and the majority of the population being PD-L1-negative.

Other studies are currently investigating immune checkpoint inhibitor and antibody–drug conjugates targeting TROP-2 combinations for TNBC treatment. The combination of durvalumab with datopotamab deruxtecan was investigated in BEGONIA arms 7 and 8 (ref. ^36^) and in phase 3 trials in various TNBC settings^36–38^. The ongoing phase 3 ASCENT-04/KEYNOTE-D19 trial is investigating sacituzumab govitecan plus pembrolizumab versus chemotherapy plus pembrolizumab in first-line PD-L1-positive metastatic TNBC. The primary endpoint, PFS, was significantly improved with sacituzumab govitecan plus pembrolizumab compared to chemotherapy plus pembrolizumab^39^. Lastly, the phase 3 TroFuse-010 study investigating sacituzumab tirumotecan alone and with pembrolizumab versus chemotherapy in participants with unresectable locally advanced or metastatic, HR-positive, HER2-negative breast cancer initiated recruitment in 2024 (ref. ^40^).

In conclusion, we report clinically relevant efficacy of first-line durvalumab plus T-DXd combination from the phase 1b/2 BEGONIA study in participants with locally advanced unresectable or metastatic, HR-negative, HER2-low breast cancer. AEs were consistent with the known safety profiles of durvalumab and T-DXd, with no unexpected toxicities observed. These data demonstrate clinical activity with an immune checkpoint inhibitor and antibody–drug conjugate combination in a PD-L1-unselected, first-line advanced or metastatic, HR-negative, HER2-low breast cancer population. Further evaluation of this approach is warranted.

## Methods

The study was conducted at multiple centers in the United States, Canada, Poland, United Kingdom, South Korea and Taiwan under local laws and regulations and in accordance with ethical principles set forth in the Declaration of Helsinki, the Council for International Organizations of Medical Sciences guidelines and International Council for Harmonisation Good Clinical Practice guidelines. The trial protocol, all amendments and other relevant documents were approved by the institutional review board or independent ethics committee at each study site. All participants provided written informed consent before enrollment. CONSORT guidelines^41^ were followed in the reporting of this study and the CONSORT checklist is available in the Supplementary Information. Further information on research design is available in the Nature Portfolio Reporting Summary linked to this article.

### Participants

Eligible participants for BEGONIA were females aged ≥18 years with untreated, unresectable, locally advanced or metastatic stage IV TNBC. Participants enrolled into arm 6 were specifically required to have documented HER2-low tumor expression (IHC 1+/ISH^−^, IHC 2+/ISH^−^ or IHC 1+/ISH untested) as determined by local testing. Other eligibility criteria included at least one unirradiated Response Evaluation Criteria in Solid Tumors (RECIST) version 1.1 lesion, Eastern Cooperative Oncology Group performance status of 0 or 1 and adequate organ and marrow function. TNBC was defined as ER-negative, PR-negative and HER2-negative according to the American Society of Clinical Oncology and College of American Pathologists testing guidelines^42,43^.

Prior treatment for stage I–III TNBC was permitted if, at the time of screening, ≥6 months had elapsed from the completion of treatment to the first documented distant recurrence. Taxane-based therapy within the previous 12 months from the date of screening and any prior exposure to immune-mediated therapy were prohibited. Participants were required to discontinue ongoing hormonal therapy for previous ER^+^ or PR^+^ breast cancer, with a 28-day washout period before study randomization. Exclusion criteria included untreated metastases of the central nervous system, active or prior autoimmune or inflammatory disorders, history of another primary malignancy, leptomeningeal carcinomatosis or active primary immunodeficiency, prior allogeneic organ transplant, uncontrolled intercurrent illness, active tuberculosis, hepatitis B virus, hepatitis C virus or HIV infections and known allergy to study therapies or their components.

Specifically for arm 6, participants were excluded if they were previously diagnosed with HER2+ breast cancer or had received HER2-targeted therapy, had a history of noninfectious pulmonary disorders, had any lung-specific intercurrent clinically important illnesses or received prior treatment with an antibody–drug conjugate that included an exatecan derivative.

### Study design

**Extended Data Fig. 2:**
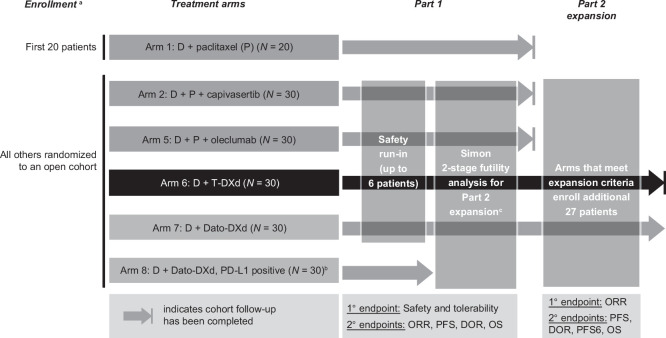
BEGONIA study design. Data from arm 6, the trastuzumab deruxtecan + durvalumab cohort, is reported in this manuscript. ^a^Cohorts were initiated at different times from December 27, 2018, through June 15, 2023. ^b^A safety run-in will not occur for this arm as Dato-DXd + D was already evaluated and found to be tolerable with no dose-limiting toxicities reported in arm 7. ^c^Novel treatment combinations may enter part 2 expansion if confirmed ORR is at least 57%. D, durvalumab; Dato-DXd, datopotamab deruxtecan; DoR, duration of response; ORR, objective response rate; OS, overall survival; PFS, progression-free survival; T-DXd, trastuzumab deruxtecan.

BEGONIA (NCT03742102) is a multicenter, multiarm, open-label, phase 1b/2 platform trial initiated to concurrently evaluate first-line novel treatment combinations for locally advanced unresectable or metastatic TNBC using a Simon’s two-stage design (Extended Data Fig. 2). The primary objective of part 1 was to assess the safety and tolerability. The primary objective of part 2 was to assess the efficacy of durvalumab combinations. Tumor biomarker analysis was an exploratory objective. The BEGONIA study used a Randomization and Trial Supply Management System (Interactive Response Technology) to centrally assign eligible participants to one of the open treatment arms. Participants were allocated into arm 6 on the basis of locally assessed HER2 tumor expression and, therefore, were not randomized. Participants with tumors classed as HER2-negative according to local HER2 expression were eligible for assignment to any open treatment arm other than arm 6.

For the arm 6 durvalumab plus T-DXd treatment combination cohort, the first six participants were monitored for dose-limiting toxicities during a run-in period, with additional participants enrolled if treatment was tolerated (around 30 evaluable participants in total for part 1). To determine whether expansion to part 2 of the Simon two-stage design was warranted for a given novel treatment combination, an assessment of confirmed ORR of participants from part 1 was performed after enrollment was complete. All participants had the opportunity to complete ≥2 on-treatment response evaluations or discontinue treatment. The arm was permitted to proceed to part 2 expansion (with approximately 27 additional evaluable participants enrolled) if confirmed ORR was at least 57% in part 1.

No compensation was offered to participants for trial participation. The study protocol is included in the Supplementary Information.

### Treatment

Participants received intravenous durvalumab (1,120 mg) every 3 weeks (Q3W) and intravenous T-DXd 5.4 mg kg^−1^ Q3W. Dose reductions were not permitted for durvalumab. For T-DXd, dose modifications including dose reductions were permitted on the basis of dose-limiting toxicity or toxicity management guidelines as applicable. The T-DXd dose could be reduced to a maximum of two dose levels, with one at 4.4 mg kg^−1^ Q3W and the other at 3.2 mg kg^−1^ Q3W. All participants were treated until disease progression or other discontinuation criteria were met; however, treatment beyond progression was permitted at the discretion of the investigator and participant.

### Assessments

Safety was assessed through physical examinations, vital signs, clinical laboratory tests, electrocardiograms and echocardiogram or multiple-gated acquisition. AEs were reported per the National Cancer Institute Common Terminology Criteria for AEs grading scale version 4.03. Immune-mediated AEs were defined as AEs of special interest consistent with an immune-mediated mechanism of action with no clear alternate etiology and that required the use of systemic corticosteroids, other immunosuppressants or endocrine therapy to manage the AE. All potential cases of ILD or pneumonitis in BEGONIA arm 6 were evaluated by an independent adjudication committee triggered by prespecified ILD or pneumonitis Medical Dictionary for Regulatory Activities Terminology (MedDRA) preferred terms, regardless of event grade. ILD and pneumonitis are AEs of special interest for T-DXd and included the preferred terms of acute respiratory failure, bronchiolitis, ILD, lung opacity, organizing pneumonia and pneumonitis. Pneumonitis as an AE of special interest for durvalumab included the preferred terms ILD and pneumonitis.

Tumors were assessed by the investigator using computed tomography or magnetic resonance imaging at baseline, then every 6 weeks for the durvalumab plus T-DXd arm for a total of 48 weeks and then every 12 weeks thereafter per RECIST version 1.1.

### Biomarker and translational analysis

The analysis of blood and tumor biomarkers was an exploratory outcome. HER2 expression was assessed locally by IHC and ISH for study enrollment. HER2-low tumor expression was defined as IHC 1+/ISH^−^, IHC 2+/ISH^−^ or IHC 1+/ISH untested. ISH was determined by fluorescence or dual ISH methods. HER2 expression was assessed centrally and retrospectively on archival tumor tissue samples by IHC using the VENTANA/PATHWAY anti-HER2/neu (4B5) assay (rabbit monoclonal primary antibody; clone 4B5, 0599957000, Roche Diagnostics). This assay is indicated for identifying persons with breast cancer who are eligible for treatment with HER2-targeted therapies. The antibody comes prediluted and was used according to manufacturer’s instructions. Tumor expression was defined as IHC 3+, 2+, 1+, ultralow (IHC 0 with membrane staining) or 0 absent membrane staining.

PD-L1 expression was assessed centrally and retrospectively on archival tumor tissue samples by IHC using the VENTANA PD-L1 (SP263) assay (rabbit monoclonal primary antibody; clone SP263, 07419821001, Roche Diagnostics). The antibody was used according to manufacturer’s instructions. Expression was defined as the percentage of the tumor area populated by tumor cells with membranous PD-L1 staining or immune cells with membranous, cytoplasmic or punctate PD-L1 staining at any intensity (TAP score). A sample was considered PD-L1-positive if the TAP score was ≥10% and PD-L1-negative if the TAP score was <10%.

### Statistical analysis

The study was sized to allow the use of a Simon two-stage design for each treatment arm according to the targeted ORR improvement from 55% to 75% with 94% power and 5% α. The treatment arm required 57 response-evaluable participants (30 in part 1 and 27 in part 2). If at least 17 of 30 participants achieved response in part 1, then the treatment arm could continue to part 2; otherwise, further recruitment into the treatment arm would be stopped. If there were at least 38 of 57 evaluable participants achieving response in a treatment arm, then the data for that cohort would be considered as having an adequate efficacy signal.

Descriptive statistics were used for baseline participant and disease characteristics. The safety analyses were performed in participants who received any amount of study treatment (safety analyses set). For the durvalumab and T-DXd arm, safety data were pooled from part 1 and part 2. Efficacy data were pooled from part 1 and part 2 of the study.

ORR was a key secondary endpoint for part 1 and the primary endpoint for part 2 and was defined as the percentage of participants with at least one confirmed complete or partial response of all treated participants with measurable disease at baseline who had the opportunity to complete at least two on-treatment disease assessments (response-evaluable analysis set). DoR, PFS, PFS at 6 months and OS were secondary endpoints in this study and were assessed for all participants who were assigned to treatment and received any amount of study treatment (intent-to-treat population). DoR was measured from the date of first documented confirmed response to the date of progression or death. PFS was measured from the date of first dose of study drug until the date of progression or death. OS was measured from the date of first dose of study drug until the date of death. All time-to-event endpoints were calculated using the Kaplan–Meier method. Data collection and analysis were not performed blind to the conditions of the experiments. Statistical analyses were performed with SAS software (version 9.4).

### Reporting summary

Further information on research design is available in the Nature Portfolio Reporting Summary linked to this article.

## Supplementary information

Reporting Summary

Supplementary Information REMARK checklist.

Supplementary Information CONSORT checklist.

Supplementary Information Study protocol.

Supplementary Tables 1–3 Supplementary Tables 1–3.

## Source data

Source Data Figs. 2–4 and Extended Data Fig. 1 Source data.

## Data Availability

Data underlying the findings described in this manuscript may be obtained in accordance with AstraZeneca’s data sharing policy (https://astrazenecagrouptrials.pharmacm.com/ST/Submission/Disclosure). Data for studies directly listed on Vivli can be requested through Vivli (www.vivli.org). Data for studies not listed on Vivli can be requested through Vivli (https://vivli.org/members/enquiries-about-studies-not-listed-on-the-vivli-platform/). The AstraZeneca Vivli member page is also available outlining further details (https://vivli.org/ourmember/astrazeneca/). Source data are provided with this paper.
