# Supplementary material for: First-line durvalumab in combination with trastuzumab deruxtecan in women with locally advanced unresectable or metastatic, hormone-receptor-negative, HER2-low breast cancer: multicenter, open-label, phase 1b/2 BEGONIA platform trial
Source: Nat Cancer. 2026 Jun 8;7(6):983–92. doi: 10.1038/s43018-026-01181-8 (PMC13309285; doi:10.1038/s43018-026-01181-8)
Supplement: Supplementary file 1 — Reporting Summary [file 43018_2026_1181_MOESM1_ESM.pdf]

nature portfolio

Corresponding author(s): Peter Schmid

Last updated by author(s): Feb 4, 2026

Reporting Summary

Nature Portfolio wishes to improve the reproducibility of the work that we publish. This form provides structure for consistency and transparency in reporting. For further information on Nature Portfolio policies, see our Editorial Policies and the Editorial Policy Checklist.

Statistics

For all statistical analyses, confirm that the following items are present in the figure legend, table legend, main text, or Methods section.

n/a	Confirmed	
☐	☒	The exact sample size (n) for each experimental group/condition, given as a discrete number and unit of measurement
☒	☐	A statement on whether measurements were taken from distinct samples or whether the same sample was measured repeatedly
☒	☐	The statistical test(s) used AND whether they are one- or two-sided<br>*Only common tests should be described solely by name; describe more complex techniques in the Methods section.*
☒	☐	A description of all covariates tested
☒	☐	A description of any assumptions or corrections, such as tests of normality and adjustment for multiple comparisons
☐	☒	A full description of the statistical parameters including central tendency (e.g. means) or other basic estimates (e.g. regression coefficient) AND variation (e.g. standard deviation) or associated estimates of uncertainty (e.g. confidence intervals)
☒	☐	For null hypothesis testing, the test statistic (e.g. F, t, r) with confidence intervals, effect sizes, degrees of freedom and P value noted<br>*Give P values as exact values whenever suitable.*
☒	☐	For Bayesian analysis, information on the choice of priors and Markov chain Monte Carlo settings
☒	☐	For hierarchical and complex designs, identification of the appropriate level for tests and full reporting of outcomes
☒	☐	Estimates of effect sizes (e.g. Cohen's d, Pearson's r), indicating how they were calculated

Our web collection on statistics for biologists contains articles on many of the points above.

Software and code

Policy information about availability of computer code

Data collection *Provide a description of all commercial, open source and custom code used to collect the data in this study, specifying the version used OR state that no software was used.*

Data analysis Statistical analyses were done with SAS software (version 9.4).

For manuscripts utilizing custom algorithms or software that are central to the research but not yet described in published literature, software must be made available to editors and reviewers. We strongly encourage code deposition in a community repository (e.g. GitHub). See the Nature Portfolio guidelines for submitting code & software for further information.

Data

Policy information about availability of data

All manuscripts must include a data availability statement. This statement should provide the following information, where applicable:

- Accession codes, unique identifiers, or web links for publicly available datasets
- A description of any restrictions on data availability
- For clinical datasets or third party data, please ensure that the statement adheres to our policy

Data underlying the findings described in this manuscript may be obtained in accordance with AstraZeneca's data sharing policy described at https://astrazenecagrouptrials.pharmacm.com/ST/Submission/Disclosure
Data for studies directly listed on Vivli can be requested through Vivli at www.vivli.org. Data for studies not listed on Vivli could be requested through Vivli at https://

vivli.org/members/enquiries-about-studies-not-listed-on-the-vivli-platform/. The AstraZeneca Vivli member page is also available outlining further details: https://vivli.org/ourmember/astrazeneca/

Research involving human participants, their data, or biological material

Policy information about studies with human participants or human data. See also policy information about sex, gender (identity/presentation), and sexual orientation and race, ethnicity and racism.

Reporting on sex and gender	The BEGONIA trial enrolled adult patients of female sex only. Gender was not considered in BEGONIA.
Reporting on race, ethnicity, or other socially relevant groupings	Race is reported in Table 1 of the manuscript. There were no race, ethnicity, or other socially relevant analyses made in this manuscript. Race was self reported.
Population characteristics	Patients presented with locally advanced unresectable or metastatic HR-negative, HER2-low breast cancer. The median age of patients was 54 years, and 67.2% of patients were White. Around one-quarter of patients had not received any prior treatment for breast cancer (all de novo metastatic). Most patients (79.3%) had a PD-L1–negative tumor status (tumor area positivity score <10%) as assessed centrally using the VENTANA PD-L1 (SP263) Assay. All were assessed to have HER2-low disease according to local testing.
Recruitment	58 patients meeting the clinical trial eligibility criteria were enrolled into Arm 6 of BEGONIA between 28 May 2020 and 28 March 2022. Recruitment into arm 6 was based on local HER2 testing results. The availability of immune checkpoint inhibitors plus chemotherapy as treatment for patients with PD-L1–positive disease may have limited recruitment of patients with PD-L1–positive disease into BEGONIA, resulting in the majority of patients in BEGONIA arm 6 being PD-L1–negative. Furthermore, the study was conducted during the COVID-19 pandemic, with 13 cases (22.4%) of COVID-19 infection recorded and it is possible that this confounded the safety results.
Ethics oversight	The study was conducted at multiple centers in the United States, Canada, Poland, United Kingdom, South Korea and Taiwan under local laws and regulations and in accordance with ethical principles set forth in the Declaration of Helsinki, the Council for International Organizations of Medical Sciences guidelines and ICH Good Clinical Practice guidelines. The trial protocol, all amendments and other relevant documents were approved by the institutional review board or independent ethics committee at each study site (list of boards/committees is available in the Supplementary Information). All patients provided written informed consent before enrollment.

Note that full information on the approval of the study protocol must also be provided in the manuscript.

Field-specific reporting

Please select the one below that is the best fit for your research. If you are not sure, read the appropriate sections before making your selection.

☒ Life sciences ☐ Behavioural & social sciences ☐ Ecological, evolutionary & environmental sciences

For a reference copy of the document with all sections, see nature.com/documents/nr-reporting-summary-flat.pdf

Life sciences study design

All studies must disclose on these points even when the disclosure is negative.

Sample size	The study was sized to allow the use of a Simon two-stage design for each treatment arm according to the targeted ORR improvement from 55% to 75% with 94% power and 5% alpha. The treatment arm required 57 response-evaluable patients (30 in part 1 and 27 in part 2). If at least 17 of 30 patients achieved response in part 1, then the treatment arm could continue to part 2; otherwise, further recruitment into the treatment arm would be stopped.<br><br>Sample sizes were only determined for the primary endpoint of ORR.
Data exclusions	All 58 patients enrolled into arm 6 received study treatment. No data were excluded.
Replication	Replication is not possible since this was a clinical trial and patients could only receive first-line treatment once, and endpoints have been assessed.
Randomization	The BEGONIA study used a Randomization and Trial Supply Management System (Interactive Response Technology) to centrally assign eligible patients to one of the open treatment arms. Patients were allocated into arm 6 based on locally assessed HER2 tumor expression, and, therefore, were not randomized. Patients with tumors classed as HER2-negative based on local HER2 expression were eligible for assignment to any open treatment arm other than Arm 6.
Blinding	BEGONIA is an open-label study. Blinding was not relevant for this study.

Reporting for specific materials, systems and methods

We require information from authors about some types of materials, experimental systems and methods used in many studies. Here, indicate whether each material, system or method listed is relevant to your study. If you are not sure if a list item applies to your research, read the appropriate section before selecting a response.

Materials & experimental systems

n/a	Involved in the study
☐	☒ Antibodies
☒	☐ Eukaryotic cell lines
☒	☐ Palaeontology and archaeology
☒	☐ Animals and other organisms
☐	☒ Clinical data
☒	☐ Dual use research of concern
☒	☐ Plants

Methods

n/a	Involved in the study
☒	☐ ChIP-seq
☒	☐ Flow cytometry
☒	☐ MRI-based neuroimaging

Antibodies

Antibodies used

Central HER2 staining: Rabbit monoclonal primary antibody (Roche), clone 4B5, reference 0599957000. Antibody comes prediluted.
Central PD-L1 staining: Rabbit monoclonal primary antibody (Roche), clone SP263, reference 07419821001.
Details of local testing methods was not captured.

Validation

Clone 4B5 has been validated as a diagnostic in breast cancer by the manufacturer and is indicated for identifying patients with breast cancer who are eligible for treatment with HER2-targeted therapies. VENTANA® anti-HER2/neu (4B5) Rabbit Monoclonal Primary Antibody. Roche Available at: https://diagnostics.roche.com/global/en/products/lab/her2-neu-4b5-ventana-rtd001197.html

Clone SP263 has not been validated as a diagnostic in breast cancer, but is validated by the manufacturer for research use in breast cancer and has been extensively used in research studies in triple negative breast cancer. Ivanova M et al. Human Pathology/ 2024;144:22-27 available at: https://www.sciencedirect.com/science/article/abs/pii/S004681772400008X. Further information can be found on the manufacturer's website: https://diagnostics.roche.com/global/en/products/lab/pd-l1-sp263-assay-ventana-rtd001235.html

Both assays were used according to manufacturers' instructions.

Clinical data

Policy information about clinical studies
All manuscripts should comply with the ICMJE guidelines for publication of clinical research and a completed CONSORT checklist must be included with all submissions.

Clinical trial registration

NCT03732677

Study protocol

Available as a supplement

Data collection

Between 28 May 2020 and 28 March 2022, 58 patients were enrolled into arm 6. Data were captured by clinical research assistants in the centers that participated in the trial using an eCRF. The study was conducted at multiple centers in the United States, Canada, Poland, United Kingdom, South Korea and Taiwan.

Outcomes

Primary endpoints were objective response rate (ORR) and safety.
Safety was assessed through physical examinations, vital signs, clinical laboratory tests, electrocardiograms and echocardiogram, or multiple-gated acquisition. Adverse events were reported per the National Cancer Institute Common Terminology Criteria for Adverse Events grading scale version 4.03. Immune-mediated adverse events were defined as adverse events of special interest consistent with an immune-mediated mechanism of action with no clear alternate etiology, and which required the use of systemic corticosteroids, other immunosuppressants or endocrine therapy to manage the adverse event. All potential cases of ILD or pneumonitis in BEGONIA arm 6 were evaluated by an independent adjudication committee triggered by prespecified ILD/ pneumonitis Medical Dictionary for Regulatory Activities Terminology (MedDRA) preferred terms, regardless of event grade.

Tumors were assessed by the investigator using computed tomography or magnetic resonance imaging at baseline, then every 6 weeks for the durvalumab + T-DXd arm, for a total of 48 weeks, then every 12 weeks thereafter per RECIST v1.1. ORR was was defined as the percentage of patients with at least one confirmed complete or partial response of all treated patients with measurable disease at baseline who had the opportunity to complete at least two on-treatment disease assessments (Response Evaluable Analysis set). DoR, PFS, PFS at 6 months and OS were secondary endpoints in this study and were assessed for all patients who were assigned to treatment and received any amount of study treatment (intention to treat population). DoR was measured from the date of first documented confirmed response to the date of progression or death. PFS was measured from the date of first dose of study drug until the date of progression or death. OS was measured from the date of first dose of study drug until the date of death. All time-to-event endpoints were calculated using the Kaplan–Meier method.

Plants

Seed stocks	*Report on the source of all seed stocks or other plant material used. If applicable, state the seed stock centre and catalogue number. If plant specimens were collected from the field, describe the collection location, date and sampling procedures.*
Novel plant genotypes	*Describe the methods by which all novel plant genotypes were produced. This includes those generated by transgenic approaches, gene editing, chemical/radiation-based mutagenesis and hybridization. For transgenic lines, describe the transformation method, the number of independent lines analyzed and the generation upon which experiments were performed. For gene-edited lines, describe the editor used, the endogenous sequence targeted for editing, the targeting guide RNA sequence (if applicable) and how the editor was applied.*
Authentication	*Describe any authentication procedures for each seed stock used or novel genotype generated. Describe any experiments used to assess the effect of a mutation and, where applicable, how potential secondary effects (e.g. second site T-DNA insertions, mosiacism, off-target gene editing) were examined.*
