# Supplementary material for: First-line durvalumab in combination with trastuzumab deruxtecan in women with locally advanced unresectable or metastatic, hormone-receptor-negative, HER2-low breast cancer: multicenter, open-label, phase 1b/2 BEGONIA platform trial
Source: Nat Cancer. 2026 Jun 8;7(6):983–92. doi: 10.1038/s43018-026-01181-8 (PMC13309285; doi:10.1038/s43018-026-01181-8)
Supplement: Supplementary file 4 — Study protocol. [file 43018_2026_1181_MOESM4_ESM.pdf]

Clinical Study Protocol

Drug Substance	Durvalumab (MEDI4736), paclitaxel, and novel oncology therapies
Study Code	D933LC00001
Version	11.0
Date	07 November 2024

A Phase IB/II, 2-Stage, Open-label, Multicenter Study to Determine the Efficacy and Safety of Durvalumab (MEDI4736) + Paclitaxel and Durvalumab (MEDI4736) in Combination With Novel Oncology Therapies With or Without Paclitaxel for First-line Metastatic Triple Negative Breast Cancer

Sponsor: AstraZeneca AB, 151 85 Södertälje, Sweden

Regulatory Agency Identifying Number(s):

IND number:	139640
EudraCT number:	2018-000764-29
EU CT Number:	2024-511646-40-00
Clinicaltrials.gov identifier:	NCT03742102

Redacted for Public Disclosure

VERSION HISTORY

DOCUMENT HISTORY	
**Document**	**Approval Date**
Version 11.0	07 November 2024
Version 10.0	23 March 2023
Version 9.0	17 January 2023
Version 8.0	25 April 2022
Version 7.0	30 August 2021
Version 6.0	12 January 2021
Version 5.0	24 July 2020
Version 4.0	20 December 2019
Version 3.0	25 April 2019
Version 2.0	4 February 2019
Original Protocol	25 July 2018

Version 11.0, 07 November 2024

This amendment is considered to be substantial based on the criteria set forth in Article 10(a) of Directive 2001/20/EC of the European Parliament and the Council of the EU and in the EU CTR Article 2, 2 (13).

Overall Rational for the Modification

The key changes implemented in this protocol amendment are to align with updates to the Dato-DXd IB edition 8.0 regarding the current safety profile of Dato-DXd, and current program-level information for durvalumab and Dato-DXd.

- Keratitis added as important identified risk for Dato-DXd. Other identified risks added were dry mouth, diarrhoea, lacrimation increased, conjunctivitis (including conjunctival disorder, conjunctival hyperaemia, and conjunctival irritation), photophobia, blepharitis, meibomian gland dysfunction, vision blurred, and visual impairment/visual acuity reduced, skin hyperpigmentation (including pigmentation disorder and skin discolouration), pruritus, dry skin/xerosis, and madarosis. Mucosal inflammation other than oral mucositis/stomatitis was updated as a potential risk for Dato-DXd in Section 2.3.5.2 based on the Dato-DXd IB.
- IRR removed as an AESI for Dato-DXd in Section 2.3.5.2. based on Dato-DXd programme standards.

- The risks were updates to durvalumab are as following: abdominal pain, cough/productive cough, thyroiditis, dysphonia, dysuria, pruritus, night sweats, anaphylaxis, pyrexia, oedema peripheral, upper respiratory tract infections, pneumonia, oral candidiasis, dental and oral soft tissue infections, influenza, myalgia, cytokine release syndrome, immune thrombocytopenia, encephalitis and immune-mediated arthritis in Section 2.3.1.1.2 to align with durvalumab IB Edition 20.
- Guidelines for overlapping toxicities of CCI [redacted] for patients on Arms 7 and 8 regardless of causality assessment for dose modification were updated in Section 6.5.2.6. based on Dato-DXd programme standards.
- IRR including anaphylaxis was removed as AESI for Dato-DXd in Section 8.3.14.5 based on latest Dato-DXd programme standards.
- Ocular Surface Toxicity' AESI updated to 'Ocular Surface Events'; and keratitis updated as an important identified risk in Section 8.3.14.5 based on the Dato-DXd programme standards.
- The guidance for Dato-DXd was updated for AE possibly causally related to Dato-DXd that have confirmed etiology other than ILD/pneumonitis to follow the management guidance outlined in "Other Non-Laboratory Adverse Events" dose modification section of Dato-DXd TMGs in Section 8.4.6.6 based on Dato-DXd programme standards.
- The IRR management guidance for Dato-DXd previous text was deleted to updated to refer to the management guidance outlined in Dato-DXd TMGs in Section 8.4.6.6 based on latest Dato-DXd programme standards.

The following amendment changes were made due to revisions of the AstraZeneca protocol template revisions:

- Addition of EU CT Number on the title page.
- Updated definition of end of study and patient considered to have completed the study in Section 4.4 based on new TransCelerate – CSP template.
- Removed the sentence "patients will not be permitted to continue immunotherapy if progression occurs after confirmed response to immunotherapy treatment in the TLs" in Section 6.1.3.
- Updated reporting requirement following final analysis. "No further data collection is required, except for reporting of SAEs and AESIs" was updated to "No further data collection is required, except for reporting of SAEs and AEs" in Section 6.6 based on latest AstraZeneca SOP.
- Updates were made in Appendix A-1 (Regulatory and ethical considerations) and added regulatory reporting requirements for SAEs to include the EU mandatory text and the latest CSP template.
- Updates were made in the Appendix A-3 (Informed consent process) based on the latest CSP template.
- Retention of records and documents by the Investigator was updated to include "Records and documents, including signed ICFs, pertaining to the conduct of this

study must be retained by the Investigator for a minimum of 25 years after study archiving or as required by local regulations, according to the AstraZeneca Global retention and Disposal (GRAD) Schedule" in Section Appendix A-7 (Data Quality Assurance) based on latest CSP template.

The following amendments changes were made to the CSP:

- Oleclumab dosage formulation [redacted] mg was added along with [redacted] mg in Table 13, Section 6.1.1.5.
- The storage of oleclumab vial information [redacted] was updated in Section 6.1.1.5 to provide clarity on the vials available for [redacted] mg and [redacted] mg dosage.
- Patients' retention time post Dato-DXd infusion was updated to [redacted] min in Section 6.1.1.7.
- IRR at any cycle during Dato-DXd infusion was updated to follow TMGs instead of the subsequent infusion for [redacted] min in Section 6.1.1.7.
- The duration between Dato-DXd and durvalumab infusion was updated to [redacted] min from [redacted] min in Section 6.1.1.7.
- The [redacted] was updated to [redacted] in Section 6.1.1.7 [redacted]
- Reporting of deaths resulting from PD to Study Clinical Lead was removed in Section 8.3.13.
- Safety data collected following the final data cut-off of the study was updated to reflect that "all AEs and SAEs will be collected and reported on a paper-based report forms or electronically as defined as AstraZeneca" in Section 8.4.2 based on the latest SOP.
- Reporting of AESIs in patients still receiving study medication after final DCO was removed in Section 8.4.2.
- The word "within timelines detailed in Section 8.4.1" was updated in reporting of AEs and SAEs in Section 8.4.2 for clarity.
- The ILD/pneumonitis management guidance was updated for DS-8201a (trastuzumab deruxtecan) in Section 8.4.6.5 based on the DS-8201a programme standards.
- General considerations for Pulmonary toxicity under toxicity management guidelines for DS-8201a table in Appendix L was updated based on the DS-8201a programme standards.
- The maximum delay in resuming the treatment for DS-8201a- and Dato-DXd was updated to [redacted] weeks for DS-8201a and [redacted] days for the Dato-DXd only if the adverse events were causally related to study treatment and not applicable to isolated COVID-19 infections in Appendix O.

This Clinical Study Protocol has been subject to a peer review according to AstraZeneca standard procedures. The Clinical Study Protocol is publicly registered, and the results are disclosed and/or published according to the AstraZeneca Global Policy on Bioethics and in compliance with prevailing laws and regulations.

TABLE OF CONTENTS

VERSION HISTORY 2
TABLE OF CONTENTS 6
LIST OF TABLES 14
LIST OF FIGURES 16
LIST OF APPENDICES 17
1 PROTOCOL SUMMARY 18
1.1 Schedule of activities 18
1.2 Synopsis 61
1.3 Schema 71
2 INTRODUCTION 72
2.1 Study rationale 72
2.2 Background 72
2.2.1 Triple negative breast cancer 72
2.2.1.1 BRCA mutations in TNBC 74
2.2.2 Immunotherapy in breast cancer 74
2.2.3 Immunotherapies 75
2.2.4 Durvalumab 77
2.2.5 Durvalumab in combination with chemotherapy and rationale for novel treatment combinations 77
2.2.6 Capivasertib (AZD5363) 82
2.2.6.1 Clinical experience of capivasertib 83
2.2.7 Oleclumab 84
2.2.8 DS-8201a (trastuzumab deruxtecan) 85
2.2.9 Dato-DXd (datopotamab deruxtecan; DS-1062a) 86
2.3 Benefit/risk assessment 88
2.3.1 Durvalumab 88
2.3.1.1 Potential benefits of durvalumab 88
2.3.1.2 Potential risks of durvalumab 88
2.3.2 Capivasertib (AZD5363) 89
2.3.2.1 Potential benefits of capivasertib 89
2.3.2.2 Potential risks identified from non-clinical studies of capivasertib 90
2.3.2.3 Potential risks of capivasertib from early clinical studies 91
2.3.2.4 Potential risks of capivasertib in combination with paclitaxel 91
2.3.3 Oleclumab (MEDI9447) 92
2.3.3.1 Potential benefits of oleclumab 92
2.3.3.2 Potential risks of oleclumab 93
2.3.3.3 Potential risks of durvalumab + oleclumab 93
2.3.4 DS-8201a (trastuzumab deruxtecan) 94
2.3.4.1 Potential benefits of DS-8201a 94
2.3.4.2 Potential risks of DS-8201a 94
2.3.4.3 Potential risks of durvalumab + DS-8201a 96

2.3.5 Dato-DXd (datopotamab deruxtecan; DS-1062a) 96
2.3.5.1 Potential benefits of Dato-DXd 96
2.3.5.2 Potential risks of Dato-DXd 97
2.3.5.3 Potential risks of durvalumab + Dato-DXd 97
2.3.6 Overall benefit/risk 98

3 OBJECTIVES AND ENDPOINTS 99

4 STUDY DESIGN 101
4.1 Overall design 101
4.1.1 Study conduct mitigation during study disruptions due to cases of civil crisis, natural disaster, or public health crisis 102
4.1.2 Part 1 study design overview 103
4.1.3 Part 2 study design overview 104
4.1.4 Part 1 safety run-in period and combination dose finding 104
4.1.4.1 Overview 104
4.1.4.2 Definitions 105
4.1.4.3 Safety Review Committee 107
4.2 Scientific rationale for study design 107
4.2.1 Rationale for efficacy study endpoints 107
4.2.2 Rationale for other study endpoints 109
4.2.3 Rationale for treatment duration 109
4.2.4 Rationale for exclusion of male patients from study 109
4.3 Justification for dose 109
4.3.1 Durvalumab dose rationale, including rationale for fixed dosing 109
4.3.2 Paclitaxel dose rationale 110
4.3.3 Capivasertib dose rationale 110
4.3.4 Oleclumab dose rationale 111
4.3.5 DS-8201a (trastuzumab deruxtecan) dose rationale 112
4.3.6 Dato-DXd (datopotamab deruxtecan; DS-1062a) dose rationale 112
4.4 End of study definition 113
4.5 Study termination 113
4.6 Site closure 114

5 STUDY POPULATION 115
5.1 Inclusion criteria 115
5.2 Exclusion criteria 117
5.3 Lifestyle restrictions 123
5.4 Screen failures 125

6 STUDY TREATMENTS 125
6.1 Treatments administered 125
6.1.1 Investigational products 125
6.1.1.1 Guidelines applicable to all treatments administered IV 129
6.1.1.2 Durvalumab (MEDI4736) 129
6.1.1.3 Paclitaxel 131

6.1.1.4 Capivasertib (AZD5363) 131
6.1.1.5 Oleclumab (MEDI9447) 133
6.1.1.6 DS-8201a (trastuzumab deruxtecan) 135
6.1.1.7 Dato-DXd (datopotamab deruxtecan; DS-1062a) 137
6.1.2 Dose and treatment regimens 140
6.1.2.1 Durvalumab and paclitaxel combination therapy (Arm 1) 141
6.1.2.2 Durvalumab + paclitaxel + novel treatment combination arms (Arms 2 and 5): overview 142
6.1.2.3 Durvalumab + paclitaxel + capivasertib combination therapy arm (Arm 2) 143
6.1.2.4 Durvalumab + paclitaxel + oleclumab combination therapy arm (Arm 5) 144
6.1.2.5 Durvalumab + DS-8201a (trastuzumab deruxtecan) combination therapy arm (Arm 6) 144
6.1.2.6 Durvalumab + Dato-DXd combination therapy arm (Arms 7 and 8) 145
6.1.3 Duration of treatment and criteria for treatment through progression 145
6.1.4 Storage 146
6.2 Measures to minimize bias: randomization and blinding 146
6.2.1 Patient enrollment and randomization 146
6.2.1.1 Enrollment procedures 148
6.2.2 Procedures for handling incorrectly enrolled or randomized patients 148
6.2.3 Methods for assigning treatment groups 149
6.2.4 Methods for ensuring blinding 149
6.3 Treatment compliance 149
6.3.1 Treatment compliance for IV study drugs 150
6.3.2 Treatment compliance for capivasertib 150
6.4 Concomitant therapy 150
6.4.1 Other concomitant treatment 153
6.4.2 Ancillary treatment for management of immuno-oncology related toxicities 154
6.5 Dose modification 154
6.5.1 Dose modifications at the cohort level during the safety run-in period of Part 1154
6.5.2 Individual patient dose modifications during all study parts 155
6.5.2.1 Durvalumab 155
6.5.2.2 Paclitaxel 155
6.5.2.3 Capivasertib 156
6.5.2.4 Oleclumab 157
6.5.2.5 DS-8201a (trastuzumab deruxtecan) 158
6.5.2.6 Dato-DXd (datopotamab deruxtecan; DS-1062a) 159
6.6 Treatment after the end of the study 162
7 DISCONTINUATION OF TREATMENT AND PATIENT WITHDRAWAL 163
7.1 Discontinuation of study treatment 163
7.1.1 Procedures for discontinuation of study treatment 164
7.2 Lost to follow-up 165
7.3 Withdrawal from the study 165
8 STUDY ASSESSMENTS AND PROCEDURES 167
8.1 Efficacy assessments 167

8.1.1 Survival assessments ... 168
8.1.2 Central Reading of Scans ... 168
8.2 Safety assessments ... 168
8.2.1 Clinical safety laboratory assessments ... 168
8.2.2 Physical examinations ... 171
8.2.3 Vital signs ... 172
8.2.4 Electrocardiograms ... 173
8.2.5 Echocardiograms/multiple gated acquisition scans ... 173
8.2.6 Early patient review for safety ... 173
8.2.7 [redacted] ... 174
8.2.8 WHO/ECOG performance status ... 174
8.2.9 Other safety assessments ... 174
8.2.9.1 Pneumonitis/ILD investigation ... 174
8.2.9.2 Safety assessments for durvalumab + DS-8201a (Arm 6) and durvalumab + Dato-DXd (Arms 7 and 8) ... 175
8.2.9.3 [redacted] ... 176
8.3 Collection of adverse events ... 176
8.3.1 Method of detecting AEs and SAEs ... 176
8.3.2 Time period and frequency for collecting AE and SAE information ... 177
8.3.3 Follow-up of AEs and SAEs ... 177
8.3.4 Adverse event data collection ... 177
8.3.5 Causality collection ... 178
8.3.6 Adverse events based on signs and symptoms ... 179
8.3.7 Adverse events based on examinations and tests ... 179
8.3.8 Hy's law ... 179
8.3.9 Pneumonitis/ILD Cases ... 180
8.3.10 Disease under study ... 180
8.3.11 Disease progression ... 180
8.3.12 New cancers ... 180
8.3.13 Deaths ... 180
8.3.14 Adverse events of special interest ... 181
8.3.14.1 Durvalumab ... 181
8.3.14.2 Capivasertib ... 182
8.3.14.3 Oleclumab ... 182
8.3.14.4 DS-8201a (trastuzumab deruxtecan) ... 183
8.3.14.5 Dato-DXd (datopotamab deruxtecan; DS-1062a) ... 184
8.4 Safety reporting and medical management ... 185
8.4.1 Reporting of serious adverse events ... 185
8.4.2 Safety Data to be Collected Following the Final Data Cutoff of the Study ... 186
8.4.3 Pregnancy ... 186
8.4.3.1 Maternal exposure ... 186
8.4.3.2 Paternal exposure ... 187
8.4.4 Overdose ... 187
8.4.5 Medication error, drug abuse, and drug misuse ... 188
8.4.5.1 Timelines ... 188
8.4.5.2 Medication error ... 188

8.4.5.3 Drug abuse 188
8.4.5.4 Drug misuse 188
8.4.6 Management of IP-related toxicities 189
8.4.6.1 Specific toxicity management and dose modification information – durvalumab and durvalumab in combination with other therapies 189
8.4.6.2 Specific toxicity management and dose modification information - paclitaxel . 190
8.4.6.3 Specific toxicity management and dose modification information - capivasertib 190
8.4.6.4 Specific toxicity management and dose modification information - oleclumab 191
8.4.6.5 Specific toxicity management and dose modification information - DS-8201a (trastuzumab deruxtecan) 191
8.4.6.6 Specific toxicity management and dose modification information - Dato-DXd 193
8.5 Pharmacokinetics and Immunogenicity 196
8.5.1 Collection of samples 196
8.5.1.1 Collection of samples to measure serum or plasma concentrations 196
8.5.1.2 Collection of samples to measure for the presence of ADAs 196
8.5.2 Storage and destruction of pharmacokinetic and ADA samples 196
8.6 Pharmacodynamics 197
8.7 [redacted] 197
8.7.1 [redacted] 197
8.7.2 [redacted] 197
8.8 [redacted] 198
8.8.1 Collection of patient tumor samples 198
8.8.2 Exploratory [redacted] 199
8.8.3 Storage, re-use, and destruction of biomarker samples 201
8.8.4 Labeling and shipment of biological samples 201
8.8.5 Chain of custody of biological samples 202
8.8.6 Withdrawal of informed consent for donated biological samples 202
9 STATISTICAL CONSIDERATIONS 203
9.1 Sample size determination 203
9.2 Populations for analyses 204
9.2.1 Full Analysis Set 204
9.2.2 Response Evaluable Analysis Set 205
9.2.3 Safety Analysis Set 205
9.2.4 PK analysis set (Part 1 only in Arms 1 to 6 and Part 2 in Arms 7 and 8) 205
9.3 Outcome measures for analyses 205
9.3.1 Calculation or derivation of efficacy variables 205
9.3.1.1 RECIST 1.1-based endpoints 205
9.3.1.2 Objective response rate 206
9.3.1.3 PFS 206
9.3.1.4 Duration of response 207
9.3.1.5 PFS 6 months 207
9.3.1.6 Overall survival 207
9.3.1.7 Change in tumor size 208
9.3.2 Calculation or derivation of safety variables 208

9.3.2.1 Adverse events 208
9.3.2.2 Safety assessments 208
9.3.3 Calculation or derivation of pharmacokinetic variables (Part 1 only) 209
9.3.3.1 Population pharmacokinetics and exposure-response/safety analysis 209
9.3.3.2 Pharmacokinetic analysis 209
9.3.4 Immunogenicity analysis (Part 1 only) 210
9.3.5 Calculation or derivation of biomarker variables 210
9.3.6 Calculation or derivation of pharmacogenetic variables 210
9.4 Statistical analyses 210
9.4.1 Safety analyses 211
9.4.1.1 Safety analyses for Part 1 211
9.4.1.2 Safety analyses for Part 2 211
9.4.2 Efficacy analyses 211
9.4.2.1 Planned Analyses 211
9.4.2.2 Exploratory analyses to be conducted if deemed necessary 213
9.4.3 Pharmacokinetic data 213
9.4.4 Pharmacokinetic/pharmacodynamic relationships 214
9.4.5 Biomarker data 214
9.4.6 Methods for multiplicity control 214
9.5 Interim analyses 214
10 REFERENCES 215
11 SUPPORTING DOCUMENTATION AND OPERATIONAL CONSIDERATIONS 226
Appendix A Regulatory, ethical and study oversight considerations 227
A-1 Regulatory and ethical considerations 227
A-2 Financial disclosure 229
A-3 Informed consent process 229
A-4 Data protection 230
A-5 Committees` structure 230
A-6 Dissemination of clinical study data 231
A-7 Data quality assurance 231
A-8 Source documents 232
A-9 Study and Site Closure 232
A-10 Publication policy 233
Appendix B Adverse event definitions and additional safety information 234
B-1 Definition of adverse events 234
B-2 Definitions of serious adverse event 234
B-3 Life threatening 235
B-4 Hospitalization 235
B-5 Important medical event or medical treatment 235
B-6 CTCAE grade 236

B-7 A guide to interpreting the causality question 236
B-8 Medication error, drug abuse, and drug misuse 237
Appendix C Handling of human biological samples 240
C-1 Chain of custody of biological samples 240
C-2 Withdrawal of Informed Consent for donated biological samples 240
C-3 International Airline Transportation Association (IATA) 6.2 guidance document 241
Appendix D [redacted] 243
[redacted] 243
..... 243
Appendix E Actions required in cases of increases in liver biochemistry and evaluation of Hy's law 247
E-1 Follow-up 247
E-2 Introduction 247
E-3 Definitions 247
E-4 Identification of potential Hy's law cases 248
E-4.1 Potential Hy's law criteria not met 248
E-4.2 Potential Hy's law criteria met 248
E-5 Review and assessment of potential Hy's law cases 249
E-6 Actions required when potential Hy's law criteria are met before and after starting study treatment 250
E-7 Actions required for repeat episodes of potential Hy's law 251
Appendix F Guidelines for evaluation of objective tumor response using RECIST 1.1 Criteria (Response Evaluation Criteria in Solid Tumors) 252
Appendix G International Airline Transportation Association (IATA) 6.2 guidance document 264
Appendix H National Institute of Allergy and Infectious Disease and Food Allergy and Anaphylaxis Network guidance for anaphylaxis diagnosis 265
Appendix I Guidance for management of specific adverse events in studies of capivasertib was removed to an Annex 266
Appendix J Guidance for management of specific adverse events in studies of selumetinib was removed 267
Appendix K Guidance regarding potential interactions of capivasertib with concomitant medications 268
Appendix L Guidance for management of specific adverse events in studies of DS-8201a (trastuzumab deruxtecan) 272
Appendix M Guidance for management of specific adverse events of Dato-DXd (datopotamab deruxtecan) was removed to an Annex 281
Appendix N Durvalumab weight-based dose calculation 282
Appendix O Instructions Related to COVID-19 (For Arms 6, 7, and 8 Only) 283

Appendix P Changes related to mitigation of study disruptions due to Cases of civil crisis, natural disaster, or public health crisis 286
P-1 Reconsent of study patients during study interruptions 286
P-2 Telemedicine visit to replace on-site visit (where applicable) 286
P-3 Data capture during telemedicine or remote visits 286
Appendix Q CCI [redacted] 287
Appendix R CCI [redacted] 288
Appendix S Abbreviations 289
Appendix T Protocol Version History 296

LIST OF TABLES

Table 1 Arm 1 (durvalumab + paclitaxel): Schedule of activities for screening and treatment periods (applies to Part 1 of the study) 19
Table 2 Arm 2 (durvalumab + paclitaxel + capivasertib): Schedule of activities for screening and treatment periods (applies to Parts 1 and 2 of the study) 24
Table 3 Arm 5 (durvalumab + paclitaxel + oleclumab): Schedule of activities for screening and treatment periods (applies to Parts 1 and 2 of the study) 30
Table 4 Arm 6 (durvalumab + DS-8201a; T-DXd [trastuzumab deruxtecan]): Schedule of activities for screening and treatment periods (applies to Parts 1 and 2 of the study) 35
Table 5 Arm 7 (durvalumab + Dato-DXd [datopotamab deruxtecan; DS-1062a]): Schedule of activities for screening and treatment periods (applies to Parts 1 and 2 of the study) 43
Table 6 Arm 8 (durvalumab + Dato-DXd [datopotamab deruxtecan; DS-1062a]): Schedule of activities for screening and treatment periods (applies to Parts 1 and 2 of the study) 51
Table 7 Schedule of assessments for patients who have completed/discontinued treatment (applies to Parts 1 and **2**) 58
Table 8 Treatment arms 64
Table 9 Treatments and treatment duration 67
Table 10 Durvalumab and nab-paclitaxel TEAEs of special interest 78
Table 11 Study objectives 99
Table 13 Study treatments 126
Table 14 Capivasertib dispensation schedule in safety run-in period 133
Table 15 Treatment arms 141
Table 16 Prohibited concomitant medications and foods 151
Table 17 Supportive medications 153
Table 18 Dose level modification in cohorts with dose reduction during the safety run-in period 155
Table 19 Dose level reductions for individual patients receiving capivasertib 156
Table 20 Dose modifications for general capivasertib-related toxicities 157
Table 21 Dose level modification for individual patients receiving DS-8201a 159
Table 22 Dose level modification for individual patients receiving Dato-DXd 161
Table 23 Clinical chemistry 170
Table 24 Hematology 170

Table 25 Coagulation 171
Table 27 Summary of outcome variables and analysis populations 204
Table 28 Pre-planned statistical and sensitivity analyses to be conducted 212
Table 29 Summary of imaging modalities for tumor assessment 252
Table 30 RECIST 1.1 evaluation of target lesions 260
Table 31 RECIST 1.1 evaluation of non-target lesions 261
Table 32 RECIST 1.1 overall visit response 262
Table 33 CYP3A4-interacting medication that should be avoided or used with caution 268
Table 34 CYP3A4, CYP2D6, or CYP2C9 substrates that should be avoided or used with caution 269
Table 35 MATE1 and/or OCT2 transporter substrates that should be used with caution 271
Table 36 Toxicity management guidelines for DS-8201a 273

LIST OF FIGURES

Figure 1 Study design 71

Figure 2 Overall survival and progression free survival in patients treated with sacituzumab govitecan versus treatment of physician's choice in patients with previously treated metastatic triple-negative breast cancer in the ASCENT study (NCT02574455) 81

Figure 3 Dosing schedule for durvalumab + paclitaxel combination therapy 142

Figure 4 Dosing schedule for durvalumab + paclitaxel + capivasertib combination therapy 143

Figure 5 Dosing schedule for durvalumab + paclitaxel + oleclumab combination therapy 144

Figure 6 Dosing schedule for durvalumab + DS-8201a (trastuzumab deruxtecan) combination therapy 144

Figure 7 Dosing schedule for durvalumab + Dato-DXd (datopotamab deruxtecan; DS-1062a) combination therapy 145

Figure 8 Dato-DXd Dose Delay Guidelines 160

LIST OF APPENDICES

Appendix A Regulatory, ethical and study oversight considerations 227
Appendix B Adverse event definitions and additional safety information 234
Appendix C Handling of human biological samples 240
Appendix D Genetics 243
Appendix E Actions required in cases of increases in liver biochemistry and evaluation of Hy's law 247
Appendix F Guidelines for evaluation of objective tumor response using RECIST 1.1 Criteria (Response Evaluation Criteria in Solid Tumors) 252
Appendix G International Airline Transportation Association (IATA) 6.2 guidance document 264
Appendix H National Institute of Allergy and Infectious Disease and Food Allergy and Anaphylaxis Network guidance for anaphylaxis diagnosis 265
Appendix I Guidance for management of specific adverse events in studies of capivasertib was removed to an Annex 266
Appendix J Guidance for management of specific adverse events in studies of selumetinib was removed 267
Appendix K Guidance regarding potential interactions of capivasertib with concomitant medications 268
Appendix L Guidance for management of specific adverse events in studies of DS-8201a (trastuzumab deruxtecan) 272
Appendix M Guidance for management of specific adverse events of Dato-DXd (datopotamab deruxtecan) was removed to an Annex 281
Appendix N Durvalumab weight-based dose calculation 282
Appendix O Instructions Related to COVID-19 (For Arms 6, 7, and 8 Only) 283
Appendix P Changes related to mitigation of study disruptions due to Cases of civil crisis, natural disaster, or public health crisis 286
Appendix Q [redacted] 287
Appendix R [redacted] 288
Appendix S Abbreviations 289
Appendix T Protocol Version History 296

1 PROTOCOL SUMMARY

1.1 Schedule of activities

The procedures for the screening and treatment periods of this study are presented in Table 1 through Table 6. The procedures for the follow-up period are presented in Table 7. The study is designed using a 2-stage approach (see Section 4.1). Study activities apply to both study parts, unless otherwise noted.

A pre-screen ICF is to be signed by patients in Arm 8 who do not have a prior determination of positive PD-L1 status, to permit determination of PD-L1 expression status by local testing prior to the 28-day screening window. At the time of signing the pre-screen ICF, Investigators should ensure that there is a reasonable possibility that the patient would be a candidate for this study based on available information. When a pre-screen ICF is signed, the main ICF should not be signed until PD-L1 positive expression status has been established.

Whenever vital signs and blood draws are scheduled for the same nominal time, the assessments should occur in the following order: vital signs and then blood draws. The timing of the vital signs assessments should be such that it allows the blood draw (eg, pharmacokinetic [redacted] sample) to occur at the timepoints indicated in the schedules of activities (SoAs). Whenever electrocardiograms (ECGs), vital signs, and blood draws are scheduled for the same nominal time, the assessments should occur in the following order: ECG, vital signs, and then blood draws. The timing of the first 2 assessments should be such that it allows the blood draw (eg, PK blood sample) to occur at the timepoints indicated in the SoAs.

For all arms, patients may delay durvalumab dosing under certain circumstances.

- Dosing may be delayed per the dosing modification and toxicity management guidelines due to either an immune or a non-immune-related adverse event (AE).
- If dosing must be delayed for reasons other than treatment-related toxicity, dosing will resume as soon as feasible.
- Dosing intervals of subsequent durvalumab cycles may be shortened as clinically feasible in order to gradually align treatment cycles with the schedule of tumor efficacy assessments (Response Evaluation Criteria in Solid Tumors, version 1.1 [RECIST 1.1]). Based on the half-life of durvalumab, subsequent time between [redacted] consecutive doses cannot be less than [redacted] days for Arms [redacted] and cannot be less than [redacted] days for Arms [redacted] (see the current Investigator's Brochure for durvalumab).

Information on dose modification (including dose reductions, as applicable) for all investigational products (IPs) is provided in Section 6.5.

Following the final DCO date by study arm, patients will be managed per standard of care (SoC) assessments at Investigator discretion.

Table 1 Arm 1 (durvalumab + paclitaxel): Schedule of activities for screening and treatment periods (applies to Part 1 of the study)

	**Screening**	**C1**		**C2**	**C3**	**C4**	**C5 to PD**	**Final visit**	**For details, see Section**
**Week**	**-4 to -1**	**0**	**2**	**Visits are driven by the treatment dosing schedule: q4w ±3 days, unless dosing needs to be held for toxicity reasons.**				**EOT visit**	
**Day**	**-28 to -1**	**1[a]**	**15**	**q28days ±3 days, unless dosing needs to be held for toxicity reasons**				**EOT visit**	
**Informed consent**									
Informed consent: study procedures[b]	X								5.1
Informed consent: [redacted] collection and analysis (optional)	[redacted]								5.1
**Study procedures**									
Physical examination (full)[c]	X								8.2.2
Targeted physical examination (based on symptoms)		X		X	X	X	X	X	8.2.2
Vital signs[c]	X	X	X	X	X	X	X	X	8.2.3
12-lead ECG (triplicate)[d,e]	X	As clinically indicated							8.2.4
MUGA scan or ECHO (for LVEF evaluation)[f]	X	As clinically indicated							8.2.5
Concomitant medications	<-->								6.4
Demography, including baseline characteristics	X								5.1
Eligibility criteria	X								5.1, 5.2
**Laboratory assessments**									
Clinical chemistry[g]	X	X[h]	X	X	X	X	X	X	Table 23
HbA1c	X								Table 23
Hematology[g,i]	X	X[h]	X	X	X	X	X	X	Table 24
Coagulation[g]	X	X[h]	As clinically indicated						Table 25

	**Screening**	**C1**		**C2**	**C3**	**C4**	**C5 to PD**	**Final visit**	**For details, see Section**
**Week**	**-4 to -1**	**0**	**2**	**Visits are driven by the treatment dosing schedule: q4w ±3 days, unless dosing needs to be held for toxicity reasons.**				**EOT visit**	
**Day**	**-28 to -1**	**1[a]**	**15**	**q28days ±3 days, unless dosing needs to be held for toxicity reasons**				**EOT visit**	
TSH (reflex free T_3 or free T_4[j])	X	X[k]		X	X	X	X	X	Table 23
Urinalysis[e,l]	X	As clinically indicated							Table 26
Hepatitis B and C and HIV	X								8.2.1
Pregnancy test[m]	X	X		X	X	X	X	X	8.2.1
**Pharmacokinetics (Part 1 only)**									
CCI [redacted]	CCI [redacted]								8.5
**Monitoring**									
WHO/ECOG performance status	X	X		X	X	X	X	X	8.2.8
AE/SAE assessment[p]	<--->								8.3
Patient follow-up contact/patient review for safety			X[q]	D15 of C2 and C3[q]					8.2.6
**IP administration**									
Durvalumab[r,s]		X		X	X	X	q4w		6.1.1.1, 6.1.1.2, 6.1.2.1
Paclitaxel[r,t]		4-week cycles: 3 weeks once weekly (D1, D8, and D15) and 1 week off					4-week cycles: 3 weeks once weekly (D1, D8, and D15) and 1 week off		6.1.1.1, 6.1.1.3, 6.1.2

	Screening	C1		C2	C3	C4	C5 to PD	Final visit	For details, see Section
**Week**	**-4 to -1**	**0**	**2**	**Visits are driven by the treatment dosing schedule: q4w ±3 days, unless dosing needs to be held for toxicity reasons.**				**EOT visit**	
**Day**	**-28 to -1**	**1[a]**	**15**	**q28days ±3 days, unless dosing needs to be held for toxicity reasons**				**EOT visit**	
**Other assessments and assays**									
CCI [redacted]									8.5
FFPE tumor sample (mandatory; a recently acquired sample is preferred if available per site's routine clinical practice; otherwise, archival sample ≤3 months can be provided)[b]	X								8.8
CCI [redacted]									8.8
									8.8
									8.8
									8.8
									8.7, 8.8
CCI [redacted]									8.8
									8.8

	Screening	C1		C2	C3	C4	C5 to PD	Final visit	For details, see Section
**Week**	**-4 to -1**	**0**	**2**	**Visits are driven by the treatment dosing schedule: q4w ±3 days, unless dosing needs to be held for toxicity reasons.**				**EOT visit**	
**Day**	**-28 to -1**	**1[a]**	**15**	**q28days ±3 days, unless dosing needs to be held for toxicity reasons**				**EOT visit**	
**Efficacy evaluations**									
Tumor assessments (CT or MRI) (RECIST 1.1)[y]	X	On-study tumor assessments occur q8w ±1 week for the first 48 weeks (relative to the date of treatment assignment for Part 1) and then q12w ±1 week thereafter until RECIST 1.1-defined radiological progression or until the patient has been taken off-study (end of study, death, or patient withdrawal of consent). The on-study schedule of q8w ±1 week for the first 48 weeks and then q12w ±1 week thereafter until progression MUST be followed regardless of any delays in dosing. For clinically stable patients receiving study treatment after RECIST 1.1-defined PD, tumor assessments MUST continue for the duration of study treatment.							8.1, Appendix F

[a] Every effort should be made to minimize the time between treatment assignment (Part 1) and starting treatment (ie, within 3 days thereafter).

[b] Written informed consent and any locally required privacy act document authorization must be obtained prior to performing any protocol-specific procedures, including screening/baseline evaluations. All patients will be required to provide consent to supply a sample of their tumor (archived [≤3 months old] or recently acquired biopsy) for entry into this study. This consent is included in the main patient ICF. Informed consent of study procedures and tumor biopsy sample may be obtained prior to the 28-day screening window, if necessary. The collection of additional biopsies upon progression is strongly encouraged. If laboratory or imaging procedures were performed for alternate reasons prior to signing consent, these can be used for screening purposes with consent of the patient. However, all screening laboratory and imaging results must have been obtained within 28 days of treatment assignment (Part 1).

[c] Body weight is recorded at each visit along with vital signs. Height will be measured at screening only. Blood pressure and pulse should be performed before, during and after the first infusion; subsequent assessments should be performed before each infusion.

[d] ECGs will be performed in triplicate at screening only and in triplicate thereafter, only if any clinically significant abnormalities are detected.

[e] Individual sites are required to indicate in the unscheduled visit eCRF if an ECG or a urinalysis was performed during study treatment.

[f] A MUGA scan or ECHO to assess LVEF will be conducted at screening and then only if clinically indicated thereafter. The modality of the cardiac function assessments must be consistent within patient (ie, if ECHO is used for the screening assessment, then ECHO should also be used for subsequent scans if required). The patients should also be examined using the same machine and operator whenever possible.

[g] Serum or plasma clinical chemistry (including LFT monitoring), hematology, and coagulation assessments may be performed more frequently if clinically indicated. At screening, serum chemistry will include fasting plasma glucose. Subsequent measurements of glucose can be either fasting or non-fasting plasma glucose measurement.

[h] If screening clinical chemistry, hematology, and coagulation assessments are performed within 3 days prior to D1 (first infusion day), they do not need to be repeated at D1.

[i] Complete blood count with differential must be checked each week when paclitaxel is administered, either 1 day prior to or on the day that paclitaxel is administered.

[j] Free T3 or free T4 will only be measured if TSH is abnormal or if there is clinical suspicion of an AE related to the endocrine system.

[k] If TSH is measured within 14 days prior to D1 (first infusion day), it does not need to be repeated at D1.

[l] The site will specify the type of test used and will provide appropriate laboratory reference range. The preferred method is urine dipstick.

[m] For women of childbearing potential only. A **urine or serum pregnancy test is acceptable.** Women of childbearing potential are required to have a serum pregnancy test within 7 days prior to the first dose of study drug and then a urine or serum pregnancy test every cycle (q4w). Pregnancy test may occur on D1, but results must be available and reviewed by the treating physician or Investigator prior to commencing an infusion.

[n] Durvalumab PK assessments will occur only in Part 1 of the study. PK sampling from C1 through C4 will be pre-dose (may not exceed 6 hours prior to start of infusion) and post-infusion (within 1 hour after the end of infusion and before paclitaxel administration).

o [redacted]

p For AEs/SAEs reported during screening, additional information such as medical history and concomitant medications may be needed.

q A study visit will occur on D15 of C1 and will include the indicated assessments (vital signs and clinical chemistry and hematology laboratories). On D15 of C2 and C3, the form of contact and procedures conducted will be at the Investigator's discretion.

r In Arm 1, durvalumab will be administered first, followed by paclitaxel.

s Results for LFTs, electrolytes, complete blood count, and creatinine must be available before commencing an infusion (within 3 days) and reviewed by the treating physician or Investigator prior to dosing.

t Paclitaxel doses can have a ±1-day dosing window (except C1D1).

u [redacted]

v [redacted]

w This blood sample is only taken [redacted] and **not** at the beginning of each cycle from C5 onward.

x [redacted]

y RECIST 1.1 assessments will be performed on images from CT (preferred) or MRI, each preferably with IV contrast, of the chest and abdomen (including the entire liver and both adrenal glands). Additional anatomy (eg, pelvis) should be imaged based on signs and symptoms of individual patients at baseline and follow-up. Digital copies of all scans (scheduled and unscheduled) must be stored at the investigative site as source documents. For clinically stable patients receiving study treatment after RECIST 1.1-defined PD, a follow-up scan is collected no earlier than 4 weeks after and no later than the next regularly scheduled imaging visit after the prior RECIST 1.1-defined PD, and this scan is evaluated using the criteria for evaluation of scans collected after a RECIST 1.1-defined PD outlined in Appendix F. If an unscheduled assessment was performed and the patient has not progressed, every attempt should be made to perform the subsequent assessments at their next regularly scheduled visit. See Section 6.1.3, Section 8.1, and Appendix F for additional details relevant to tumor assessments.

Note: All assessments on treatment days are to be performed prior to infusion, unless otherwise indicated.

ADA Anti-drug antibodies; AE Adverse event; C Cycle; CT Computed tomography; [redacted] D Day; [redacted] ECG Electrocardiogram; ECHO Echocardiogram; ECOG Eastern Cooperative Oncology Group; eCRF Electronic case report form; EOT End of treatment; FFPE Formalin-fixed and paraffin-embedded; HbA1c Glycosylated hemoglobin; HIV Human immunodeficiency virus; ICF Informed consent form; IV Intravenous; IP Investigational product; LFT Liver function test; LVEF Left ventricular ejection fraction; MRI Magnetic resonance imaging; MUGA Multiple gated acquisition; NA Not applicable; [redacted] PK Pharmacokinetic; q28days Every 28 days; q2w Every 2 weeks; q4w Every 4 weeks; q8w Every 8 weeks; q12w Every 12 weeks; RECIST Response Evaluation Criteria In Solid Tumors; [redacted] SAE Serious adverse event; T3 Triiodothyronine; T4 Thyroxine; TSH Thyroid-stimulating hormone; WHO World Health Organization.

Table 2 Arm 2 (durvalumab + paclitaxel + capivasertib): Schedule of activities for screening and treatment periods (applies to Parts 1 and 2 of the study)

	Screening	C1		C2	C3	C4	C5 to PD	Final visit	For details, see Section
**Week**	**-4 to -1**	**0**	**2**	**Visits are driven by the treatment dosing schedule: q4w ±3 days, unless dosing needs to be held for toxicity reasons.**				**EOT visit**	
**Day**	**-28 to -1**	**1[a]**	**15**	**q28 days ±3 days, unless dosing needs to be held for toxicity reasons**				**EOT visit**	
**Informed consent**									
Informed consent: study procedures[b]	X								5.1
Informed consent: [redacted] collection and analysis (optional)	[redacted]								5.1
**Study procedures**									
Physical examination (full)[c]	X								8.2.2
Targeted physical examination (based on symptoms)		X		X	X	X	X	X	8.2.2
Vital signs[c]	X	X	X	X	X	X	X	X	8.2.3
12-lead ECG (triplicate)[d,e]	X	X		X (C2D1)	q12w and as clinically indicated				8.2.4
MUGA scan or ECHO (for LVEF evaluation)[f]	X	C4D1 (±1 week); as clinically indicated thereafter							8.2.5
Concomitant medications	<--->								6.4
Demography, including baseline characteristics	X								5.1
Eligibility criteria	X								5.1, 5.2
**Laboratory assessments**									
Clinical chemistry[g]	X	X[h]	X	X	X	X	X	X	Table 23
Post-dose glucose[g]		X		X					Table 23

	**Screening**	**C1**		**C2**	**C3**	**C4**	**C5 to PD**	**Final visit**	**For details, see Section**
**Week**	**-4 to -1**	**0**	**2**	**Visits are driven by the treatment dosing schedule: q4w ±3 days, unless dosing needs to be held for toxicity reasons.**				**EOT visit**	
**Day**	**-28 to -1**	**1[a]**	**15**	**q28 days ±3 days, unless dosing needs to be held for toxicity reasons**				**EOT visit**	
HbA1c	X					C4D1 and q12w thereafter		X	Table 23
Hematology[g,i]	X	X[h]	X	X	X	X	X	X	Table 24
Coagulation[g]	X	X[h]	As clinically indicated						Table 25
TSH (reflex free T3 or free T4[j])	X	X[k]		X	X	X	X	X	Table 23
Urinalysis[e,l]	X	As clinically indicated							Table 26
Hepatitis B and C and HIV	X								8.2.1
Pregnancy test[m]	X	X		X	X	X	X	X	8.2.1
**Pharmacokinetics (Part 1 only)**									
[redacted]									8.5
									8.5
**Monitoring**									
WHO/ECOG performance status	X	X		X	X	X	X	X	8.2.8
AE/SAE assessment[p]	<-->								8.3
Patient follow-up contact/patient review for safety			X[q]	D15 of C2 and C3[q]					8.2.6
Patient dosing diary	To be completed each time capivasertib is taken								8.2.7
**IP administration**									
Capivasertib (AZD5363)[r]		4-week cycles; 3 weeks bid on (D2, D3, D4, and D5) and 1 week off					4-week cycles; 3 weeks bid on (D2, D3, D4, and D5) and 1 week off		6.1.1.4, 6.1.2.2, 6.1.2.3

	Screening	C1		C2	C3	C4	C5 to PD	Final visit	For details, see Section
**Week**	**-4 to -1**	**0**	**2**	**Visits are driven by the treatment dosing schedule: q4w ±3 days, unless dosing needs to be held for toxicity reasons.**				**EOT visit**	
**Day**	**-28 to -1**	**1[a]**	**15**	**q28 days ±3 days, unless dosing needs to be held for toxicity reasons**				**EOT visit**	
Durvalumab[r,s]		X		X	X	X	q4w		6.1.1.1, 6.1.1.2, 6.1.2.2
Paclitaxel[r,t]		4-week cycles: 3 weeks once weekly (D1, D8, and D15) and 1 week off					4-week cycles: 3 weeks once weekly (D1, D8, and D15) and 1 week off		6.1.1.1, 6.1.1.3, 6.1.2.2
**Other assessments and assays**									
CCI [redacted]									8.5
FFPE tumor sample (mandatory; a recently acquired sample is preferred if available per site's routine clinical practice; otherwise, archival sample ≤3 months old can be provided)[b]	X								8.8
CCI [redacted]									8.8
CCI [redacted]									8.8
									8.8
									8.8

	Screening	C1		C2	C3	C4	C5 to PD	Final visit	For details, see Section
**Week**	**-4 to -1**	**0**	**2**	**Visits are driven by the treatment dosing schedule: q4w ±3 days, unless dosing needs to be held for toxicity reasons.**				**EOT visit**	
**Day**	**-28 to -1**	**1[a]**	**15**	**q28 days ±3 days, unless dosing needs to be held for toxicity reasons**				**EOT visit**	
CCI [redacted]									8.7, 8.8
[redacted]									8.8
[redacted]									8.8
**Efficacy evaluations**									
Tumor assessments (CT or MRI) (RECIST 1.1)[y]	X	On-study tumor assessments occur q8w ±1 week for the first 48 weeks (relative to the date of treatment assignment) and then q12w ±1 week thereafter until RECIST 1.1-defined radiological progression or until the patient has been taken off-study (end of study, death, or patient withdrawal of consent). The on-study schedule of q8w ±1 week for the first 48 weeks and then q12w ±1 week thereafter until progression MUST be followed regardless of any delays in dosing. For clinically stable patients receiving study treatment after RECIST 1.1-defined PD, tumor assessments MUST continue for the duration of study treatment.							8.1, Appendix F

a Every effort should be made to minimize the time between treatment assignment and starting treatment (within 3 days thereafter).

b Written informed consent and any locally required privacy act document authorization must be obtained prior to performing any protocol-specific procedures, including screening/baseline evaluations. All patients will be required to provide consent to supply a sample of their tumor (archived [≤3 months old] or recently acquired biopsy) for entry into this study. This consent is included in the main patient ICF. Informed consent of study procedures and tumor biopsy sample may be obtained prior to the 28-day screening window, if necessary. The collection of additional biopsies upon progression is strongly encouraged. If laboratory or imaging procedures were performed for alternate reasons prior to signing consent, these can be used for screening purposes with consent of the patient. However, all screening laboratory and imaging results must have been obtained within 28 days of treatment assignment.

c Body weight is recorded at each visit along with vital signs. Height will be measured at screening only. Blood pressure and pulse should be performed before, during and after the first infusion; subsequent assessments should be performed before each infusion.

d Pre-dose ECGs will be performed in triplicate when indicated and if clinically significant abnormalities are detected.

e Individual sites are required to indicate in the unscheduled visit eCRF if an ECG or a urinalysis was performed during study treatment.

f A MUGA scan or ECHO to assess LVEF will be conducted at screening, on C4D1 (12 weeks ±1 week) at any time of the day, and then only if clinically indicated thereafter. A further assessment will be required at IP discontinuation. The modality of the cardiac function assessments must be consistent within patient (ie, if ECHO is used for the screening assessment, then ECHO should also be used for subsequent scans if required). The patients should also be examined using the same machine and operator whenever possible.

g Serum or plasma clinical chemistry (including LFT monitoring), hematology, and coagulation assessments may be performed more frequently if clinically indicated. Serum chemistry for Arm 2 includes fasting plasma glucose at screening, fasting plasma glucose at baseline (pre-dose) and fasting or non-fasting 4 hours post-capivasertib dose on C1D2 and C2D2. From C3 onwards fasting plasma glucose should be taken pre-dose. Further monitoring may be performed at the Investigator's discretion based on patient characteristics. 'Fasting' is defined as no caloric intake for ≥4 hours before sampling.

h If screening clinical chemistry, hematology, and coagulation assessments are performed within 3 days prior to D1 (first infusion day), they do not need to be repeated at D1.

i Complete blood count with differential must be checked each week when paclitaxel is administered, either 1 day prior to or on the day that paclitaxel is administered.

j Free T3 or free T4 will only be measured if TSH is abnormal or if there is clinical suspicion of an AE related to the endocrine system.

k If TSH is measured within 14 days prior to D1 (first infusion day), it does not need to be repeated at D1.

l The site will specify the type of test used and will provide appropriate laboratory reference range. The preferred method is urine dipstick.

m For women of childbearing potential only. **A urine or serum pregnancy test is acceptable.** Women of childbearing potential are required to have a serum pregnancy test within 7 days prior to the first dose of study drug and then a urine or serum pregnancy test every cycle (q4w). Pregnancy test may occur on D1, but results must be available and reviewed by the treating physician or Investigator prior to commencing an infusion.

n [redacted]

o [redacted]

p For AEs/SAEs reported during screening, additional information such as medical history and concomitant medications may be needed.

q A study visit will occur on D15 of C1 and will include the indicated assessments (vital signs and clinical chemistry and hematology laboratories). On D15 of C2 and C3, the form of contact and procedures conducted will be at the Investigator's discretion.

r For Arm 2, durvalumab will be administered first, followed by paclitaxel; capivasertib is administered on a different day. Capivasertib should not be taken on any week that paclitaxel infusion is not administered, either due to a scheduled off-drug week or unscheduled omission or delay. On Cycle 1 Days 2 through 5, prednisone 10 mg or equivalent should be administered.

s Results for LFTs, electrolytes, complete blood count, fasting glucose, and creatinine must be available before commencing an infusion (within 3 days) and reviewed by the treating physician or Investigator prior to dosing.

t Paclitaxel doses can have a ±1-day dosing window (except C1D1). Paclitaxel must not be administered the day after last dose of capivasertib.

u [redacted]

v [redacted]

w This blood sample is only taken [redacted] and **not** at the beginning of each cycle from C5 onward.

x [redacted].

y RECIST 1.1 assessments will be performed on images from CT (preferred) or MRI, each preferably with IV contrast, of the chest and abdomen (including the entire liver and both adrenal glands). Additional anatomy (eg, pelvis) should be imaged based on signs and symptoms of individual patients at baseline and follow-up. Digital copies of all scans (scheduled and unscheduled) must be stored at the investigative site as source documents. For clinically stable patients receiving study treatment after RECIST 1.1-defined PD, a follow-up scan is collected no earlier than 4 weeks after and no later than the next regularly scheduled imaging visit after the prior RECIST 1.1-defined PD, and this scan is evaluated using the criteria for evaluation of scans collected after a RECIST 1.1-defined PD outlined in Appendix F. If an unscheduled assessment was performed and the patient has not progressed, every attempt should be made to perform the subsequent assessments at their next regularly scheduled visit. See Section 6.1.3, Section 8.1, and Appendix F for additional details relevant to tumor assessments.

Note: All assessments on treatment days are to be performed prior to infusion, unless otherwise indicated.

ADA Anti-drug antibodies; AE Adverse event; C Cycle; CT Computed tomography; [redacted]; D Day; [redacted]; ECG Electrocardiogram; ECHO Echocardiogram; ECOG Eastern Cooperative Oncology Group; eCRF Electronic case report form; EOT End of treatment; FFPE Formalin-fixed and paraffin-embedded; HbA1c Glycosylated hemoglobin; HIV Human immunodeficiency virus; ICF Informed consent form; IV Intravenous; IP Investigational product; LFT Liver function test; LVEF Left ventricular ejection fraction; MRI Magnetic resonance imaging; MUGA Multiple gated acquisition; PD Progression of disease; PK Pharmacokinetic; q28days Every 28 days; q2w Every 2 weeks; q4w Every 4 weeks; q8w Every 8 weeks; q12w Every 12 weeks; RECIST Response Evaluation Criteria In Solid Tumors; [redacted]; SAE Serious adverse event; T3 Triiodothyronine; T4 Thyroxine; TSH Thyroid-stimulating hormone; WHO World Health Organization.

<<Arm 3 (durvalumab + paclitaxel + selumetinib) was removed with Protocol Version 3>>

<<Arm 4 (durvalumab + paclitaxel + danvatirsen) was removed with Protocol Version 4>>

Table 3 Arm 5 (durvalumab + paclitaxel + oleclumab): Schedule of activities for screening and treatment periods (applies to Parts 1 and 2 of the study)

	Screening	C1		C2		C3	C4	C5 to PD	Final visit	For details, see Section
**Week**	**-4 to -1**	**0**	**2**	**4**	**6**	**Visits are driven by the treatment dosing schedule: q4w ±3 days, unless dosing needs to be held for toxicity reasons.**			**EOT visit**	
**Day**	**-28 to -1**	**1[a]**	**15**	**29**	**43**	**q28days ±3 days, unless dosing needs to be held for toxicity reasons**			**EOT visit**	
**Informed consent**										
Informed consent: study procedures[b]	X									5.1
Informed consent: [redacted] collection and analysis (optional)	[redacted]									5.1
**Study procedures**										
Physical examination (full)[c]	X									8.2.2
Targeted physical examination (based on symptoms)		X		X		X	X	X	X	8.2.2
Vital signs[c]	X	X	X	X	X	X	X	X	X	8.2.3
12-lead ECG (triplicate)[d,e]	X	As clinically indicated								8.2.4
MUGA scan or ECHO (for LVEF evaluation)[f]	X	As clinically indicated								8.2.5
Concomitant medications	<--->									6.4
Demography, including baseline characteristics	X									5.1
Eligibility criteria	X									5.1, 5.2
**Laboratory assessments**										
Clinical chemistry[g]	X	X[h]	X	X	X	X	X	X	X	Table 23
HbA1c	X									Table 23
Hematology[g,i]	X	X[h]	X	X	X	X	X	X	X	Table 24

	Screening	C1		C2		C3	C4	C5 to PD	Final visit	For details, see Section
**Week**	**-4 to -1**	**0**	**2**	**4**	**6**	**Visits are driven by the treatment dosing schedule: q4w ±3 days, unless dosing needs to be held for toxicity reasons.**			**EOT visit**	
**Day**	**-28 to -1**	**1[a]**	**15**	**29**	**43**	**q28days ±3 days, unless dosing needs to be held for toxicity reasons**			**EOT visit**	
Coagulation[g]	X	X[h]	As clinically indicated							
TSH (reflex free T3 or free T4[j])	X	X[k]		X		X	X	X	X	Table 23
Urinalysis[e,l]	X	As clinically indicated								Table 26
Hepatitis B and C and HIV	X									8.2.1
Pregnancy test[m]	X	X		X		X	X	X	X	8.2.1
**Pharmacokinetics (Part 1 only)**										
CCI [redacted]										8.5
										8.5
**Monitoring**										
WHO/ECOG performance status	X	X		X		X	X	X	X	8.2.8
AE/SAE assessment[p]	<-->									8.3
Patient follow-up contact/patient review for safety			X[r]		X	D15 of C3[q]				8.2.6
**IP administration**										
Oleclumab[r,s]		q2w for the first 2 cycles (D1 and D15 of C1 and C2)				q4w starting at C3 (D1)				6.1.1.5, 6.1.2.2, 6.1.2.4
Durvalumab[r,t]		X		X		X	X	q4w		6.1.1.1, 6.1.1.2, 6.1.2.2

	Screening	C1		C2		C3	C4	C5 to PD	Final visit	For details, see Section
**Week**	**-4 to -1**	**0**	**2**	**4**	**6**	**Visits are driven by the treatment dosing schedule: q4w ±3 days, unless dosing needs to be held for toxicity reasons.**			**EOT visit**	
**Day**	**-28 to -1**	**1[a]**	**15**	**29**	**43**	**q28days ±3 days, unless dosing needs to be held for toxicity reasons**			**EOT visit**	
Paclitaxel[r,u]		4-week cycles: 3 weeks once weekly (D1, D8, and D15) and 1 week off						4-week cycles: 3 weeks once weekly (D1, D8, and D15) and 1 week off		6.1.1.1, 6.1.1.3, 6.1.2.2
**Other assessments and assays**										
[redacted]										8.5
										8.5
FFPE tumor sample (mandatory; a recently acquired sample is preferred if available per site's routine clinical practice; otherwise, archival sample ≤3 months old can be provided)[b]	X									8.8
[redacted]										8.8
										8.8
										8.8

	Screening	C1		C2		C3	C4	C5 to PD	Final visit	For details, see Section
**Week**	**-4 to -1**	**0**	**2**	**4**	**6**	**Visits are driven by the treatment dosing schedule: q4w ±3 days, unless dosing needs to be held for toxicity reasons.**			**EOT visit**	
**Day**	**-28 to -1**	**1[a]**	**15**	**29**	**43**	**q28days ±3 days, unless dosing needs to be held for toxicity reasons**			**EOT visit**	
CCI [redacted]										8.7, 8.8
										8.8
										8.8
**Efficacy evaluations**										
Tumor assessments (CT or MRI) (RECIST 1.1)[z]	X	On-study tumor assessments occur q8w ±1 week for the first 48 weeks (relative to the date of treatment assignment) and then q12w ±1 week thereafter until RECIST 1.1-defined radiological progression or until the patient has been taken off-study (end of study, death, or patient withdrawal of consent). The on-study schedule of q8w ±1 week for the first 48 weeks and then q12w ±1 week thereafter until progression MUST be followed regardless of any delays in dosing. For clinically stable patients receiving study treatment after RECIST 1.1-defined PD, tumor assessments MUST continue for the duration of study treatment.								8.1, Appendix F

a Every effort should be made to minimize the time between treatment assignment and starting treatment (within 3 days thereafter).

b Written informed consent and any locally required privacy act document authorization must be obtained prior to performing any protocol-specific procedures, including screening/baseline evaluations. All patients will be required to provide consent to supply a sample of their tumor (archived [≤3 months old] or recently acquired biopsy) for entry into this study. This consent is included in the main patient ICF. Informed consent of study procedures and tumor biopsy sample may be obtained prior to the 28-day screening window, if necessary. The collection of additional biopsies upon progression is strongly encouraged. If laboratory or imaging procedures were performed for alternate reasons prior to signing consent, these can be used for screening purposes with consent of the patient. However, all screening laboratory and imaging results must have been obtained within 28 days of treatment assignment.

c Body weight is recorded at each visit along with vital signs. Height will be measured at screening only. Blood pressure and pulse should be performed before, during and after the first infusion; subsequent assessments should be performed before each infusion.

d ECGs will be performed in triplicate at screening only and in triplicate thereafter, only if any clinically significant abnormalities are detected.

e Individual sites are required to indicate in the unscheduled visit eCRF if an ECG or a urinalysis was performed during study treatment.

f A MUGA scan or ECHO to assess LVEF will be conducted at screening and then only if clinically indicated thereafter. The modality of the cardiac function assessments must be consistent within patient (ie, if ECHO is used for the screening assessment, then ECHO should also be used for subsequent scans if required). The patients should also be examined using the same machine and operator whenever possible.

g Serum or plasma clinical chemistry (including LFT monitoring), hematology, and coagulation assessments may be performed more frequently if clinically indicated. At screening, serum chemistry will include fasting plasma glucose. Subsequent measurements of glucose can be either fasting or non-fasting plasma glucose measurement.

h If screening clinical chemistry, hematology, and coagulation assessments are performed within 3 days prior to D1 (first infusion day), they do not need to be repeated at D1.
i Complete blood count with differential must be checked weekly each week when paclitaxel is administered, either 1 day prior to or on the day that paclitaxel is administered.
j Free T3 or free T4 will only be measured if TSH is abnormal or if there is clinical suspicion of an AE related to the endocrine system.
k If TSH is measured within 14 days prior to D1 (first infusion day), it does not need to be repeated at D1.
l The site will specify the type of test used and will provide appropriate laboratory reference range. The preferred method is urine dipstick.
m For women of childbearing potential only. **A urine or serum pregnancy test is acceptable**. Women of childbearing potential are required to have a serum pregnancy test within 7 days prior to the first dose of study drug and then a urine or serum pregnancy test every cycle (q4w). Pregnancy test may occur on D1, but the results must be available and reviewed by the treating physician or Investigator prior to commencing an infusion.
n [redacted]
o [redacted]
p For AEs/SAEs reported during screening, additional information such as medical history and concomitant medications may be needed.
q Study visits will occur on D15 of C1 and C2 and will include the indicated assessments (vital signs and clinical chemistry and hematology laboratories). On D15 of C3, the form of contact and procedures conducted will be at the Investigator's discretion.
r Oleclumab will be administered first, followed by durvalumab, then followed by paclitaxel. During the combination portion of treatment, the durvalumab infusion will start approximately [redacted] after the end of the infusion of oleclumab. If there are no clinically significant infusion reactions within the first cycle, and at the discretion of the Investigator, then for all other cycles, the durvalumab can be given no less than [redacted] after the infusion of oleclumab has finished.
s Oleclumab dose can have a ±1-day dosing window for the first 2 cycles followed by a ±2-day dosing window starting with Cycle 3.
t Results for LFTs, electrolytes, complete blood count, and creatinine must be available before commencing an infusion (within 3 days) and reviewed by the treating physician or Investigator prior to dosing.
u Paclitaxel doses can have a ±1-day dosing window (except C1D1).
v [redacted]
w [redacted]
x This blood sample is only taken [redacted] and **not** at the beginning of each cycle from C5 onward.
y [redacted]
z RECIST 1.1 assessments will be performed on images from CT (preferred) or MRI, each preferably with IV contrast, of the chest and abdomen (including the entire liver and both adrenal glands). Additional anatomy (eg, pelvis) should be imaged based on signs and symptoms of individual patients at baseline and follow-up. Digital copies of all scans (scheduled and unscheduled) must be stored at the investigative site as source documents. For clinically stable patients receiving study treatment after RECIST 1.1-defined PD, a follow-up scan is collected no earlier than 4 weeks after and no later than the next regularly scheduled imaging visit after the prior RECIST 1.1-defined PD, and this scan is evaluated using the criteria for evaluation of scans collected after a RECIST 1.1-defined PD outlined in Appendix F. If an unscheduled assessment was performed and the patient has not progressed, every attempt should be made to perform the subsequent assessments at their next regularly scheduled visit. See Section 6.1.3, Section 8.1, and Appendix F for additional details relevant to tumor assessments.

Note: All assessments on treatment days are to be performed prior to infusion, unless otherwise indicated.
ADA Anti-drug antibodies; AE Adverse event; C Cycle; CT Computed tomography; [redacted] D Day; [redacted]; ECG Electrocardiogram; ECHO Echocardiogram; ECOG Eastern Cooperative Oncology Group; eCRF Electronic case report form; EOT End of treatment; FFPE Formalin-fixed and paraffin-embedded; HbA1c Glycosylated hemoglobin; HIV Human immunodeficiency virus; ICF Informed consent form; IV Intravenous; IP Investigational product; LFT Liver function test; LVEF Left ventricular ejection fraction; MRI Magnetic resonance imaging; MUGA Multiple gated acquisition; PD Progression of disease; PK Pharmacokinetic; q28days Every 28 days; q2w Every 2 weeks; q4w Every 4 weeks; q8w Every 8 weeks; q12w Every 12 weeks; RECIST Response Evaluation Criteria In Solid Tumors; [redacted]; SAE Serious adverse event; T3 Triiodothyronine; T4 Thyroxine; TSH Thyroid-stimulating hormone; WHO World Health Organization.

Table 4 Arm 6 (durvalumab + DS-8201a; T-DXd [trastuzumab deruxtecan]): Schedule of activities for screening and treatment periods (applies to Parts 1 and 2 of the study)

	Screening	C1			C2			C3	C4	C5	C6 to PD	Final visit	For details, see Section
**Week**	**-4 to -1**	**0**	**1**	**2**	**3**	**4**	**5**	**Visits are driven by the treatment dosing schedule: q3w, unless dosing needs to be held for toxicity reasons.**				**EOT visit**	
**Cycle Day**	**-28 to -1**	**1[a]**	**8**	**15**	**1**	**8**	**15**	**q21 days, unless dosing needs to be held for toxicity reasons**					
**Window (days)**		**0[a]**	**±2**	**±2**	**±2**	**±2**	**±2**	**±2**	**±2**	**±2**	**±2**	**±7**	
**Informed consent**													
Informed consent: study procedures[b]	X												5.1
Informed consent: [redacted] collection and analysis (optional)	[redacted]												5.1
**Study procedures**													
Eligibility criteria	X												5.1, 5.2
Demography, including baseline characteristics	X												5.1
Physical examination (full)[c]	X												8.2.2
Targeted physical examination (based on symptoms)		X	X		X			X	X	X	X	X	8.2.2
Vital signs[c]	X	X	X	X	X			X	X	X	X	X	8.2.3
12-lead ECG (triplicate)[d,e]	X	X							C5 and every 4 cycles thereafter				8.2.4
MUGA scan or ECHO (for LVEF evaluation)[f]	X									C5 every 4 cycles (±7 days) thereafter			8.2.5

	Screening	C1			C2			C3	C4	C5	C6 to PD	Final visit	For details, see Section
**Week**	**-4 to -1**	**0**	**1**	**2**	**3**	**4**	**5**	**Visits are driven by the treatment dosing schedule: q3w, unless dosing needs to be held for toxicity reasons.**				**EOT visit**	
**Cycle Day**	**-28 to -1**	**1[a]**	**8**	**15**	**1**	**8**	**15**	**q21 days, unless dosing needs to be held for toxicity reasons**					
**Window (days)**		**0[a]**	**±2**	**±2**	**±2**	**±2**	**±2**	**±2**	**±2**	**±2**	**±2**	**±7**	
[redacted]													8.2.9
PFT[h]	X												8.2.9
Pulmonary HRCT (or CT if HRCT is contraindicated) as per institutional guidelines	X												8.2.9
[redacted]													8.2.9
Concomitant medications	<-->												6.4
**Laboratory assessments**													
Clinical chemistry[j]	X	X[k]	X	X	X	X	X	X	X	X	X	X	Table 23
HbA1c	X												Table 23
Hematology[j]	X	X[k]	X	X	X	X	X	X	X	X	X	X	Table 24
Coagulation[j]	X	X[k]	As clinically indicated									X	Table 25
TSH (reflex free T3 or free T4[l])	X	X[m]			X			X	X	X	X	X	Table 23
Urinalysis[e,n]	X	As clinically indicated											Table 26
Hepatitis B and C and HIV	X												8.2.1
Troponin T[o]	X											X	8.4.6.5
Pregnancy test[p]	X	X			X			X	X	X	X	X	8.2.1

	Screening	C1			C2			C3	C4	C5	C6 to PD	Final visit	For details, see Section
**Week**	**-4 to -1**	**0**	**1**	**2**	**3**	**4**	**5**	**Visits are driven by the treatment dosing schedule: q3w, unless dosing needs to be held for toxicity reasons.**				**EOT visit**	
**Cycle Day**	**-28 to -1**	**1[a]**	**8**	**15**	**1**	**8**	**15**	**q21 days, unless dosing needs to be held for toxicity reasons**					
**Window (days)**		**0[a]**	**±2**	**±2**	**±2**	**±2**	**±2**	**±2**	**±2**	**±2**	**±2**	**±7**	
**Pharmacokinetics (Part 1 only)**													
[redacted]													8.5
													8.5
**Monitoring**													
WHO/ECOG performance status	X	X			X				X		X	X	8.2.8
AE/SAE assessment[u]	<-->												8.3
Patient follow-up contact/patient review for safety				X[v]			X[v]						8.2.6
**IP administration**													
DS-8201a[w,x]		X			X			X	X	X	X		6.1.1.1, 6.1.2.5
Durvalumab[w,y]		X			X			X	X	X	X		6.1.1.1, 6.1.1.2, 6.1.2.5

	Screening	C1			C2			C3	C4	C5	C6 to PD	Final visit	For details, see Section
**Week**	**-4 to -1**	**0**	**1**	**2**	**3**	**4**	**5**	**Visits are driven by the treatment dosing schedule: q3w, unless dosing needs to be held for toxicity reasons.**				**EOT visit**	
**Cycle Day**	**-28 to -1**	**1[a]**	**8**	**15**	**1**	**8**	**15**	**q21 days, unless dosing needs to be held for toxicity reasons**					
**Window (days)**		**0[a]**	**±2**	**±2**	**±2**	**±2**	**±2**	**±2**	**±2**	**±2**	**±2**	**±7**	
**Other assessments and assays**													
CCI [redacted]													8.5
													8.5
FFPE tumor sample (mandatory; a recently acquired sample is preferred if available per site's routine clinical practice; otherwise, archival sample ≤3 months old can be provided)[b]	X												8.8
CCI [redacted]													8.8
													8.8

	Screening	C1			C2			C3	C4	C5	C6 to PD	Final visit	For details, see Section
**Week**	**-4 to -1**	**0**	**1**	**2**	**3**	**4**	**5**	**Visits are driven by the treatment dosing schedule: q3w, unless dosing needs to be held for toxicity reasons.**				**EOT visit**	
**Cycle Day**	**-28 to -1**	**1[a]**	**8**	**15**	**1**	**8**	**15**	**q21 days, unless dosing needs to be held for toxicity reasons**					
**Window (days)**		**0[a]**	**±2**	**±2**	**±2**	**±2**	**±2**	**±2**	**±2**	**±2**	**±2**	**±7**	
CCI [redacted]													8.8
[redacted]													8.8
[redacted]													8.8
[redacted]													8.8.2
[redacted]													8.8
[redacted]													8.7

	Screening	C1			C2			C3	C4	C5	C6 to PD	Final visit	For details, see Section
**Week**	**-4 to -1**	**0**	**1**	**2**	**3**	**4**	**5**	**Visits are driven by the treatment dosing schedule: q3w, unless dosing needs to be held for toxicity reasons.**				**EOT visit**	
**Cycle Day**	**-28 to -1**	**1[a]**	**8**	**15**	**1**	**8**	**15**	**q21 days, unless dosing needs to be held for toxicity reasons**					
**Window (days)**		**0[a]**	**±2**	**±2**	**±2**	**±2**	**±2**	**±2**	**±2**	**±2**	**±2**	**±7**	
**Efficacy evaluations**													
Tumor assessments (CT or MRI) (RECIST 1.1)[dd]	X	On-study tumor assessments occur q6w ±1 week for the first 48 weeks (relative to the date of treatment assignment) and then q12w ±1 week thereafter until RECIST 1.1-defined radiological progression or until the patient has been taken off-study (end of study, death, or patient withdrawal of consent). The on-study schedule of q6w ±1 week for the first 48 weeks and then q12w ±1 week thereafter until progression MUST be followed regardless of any delays in dosing. For clinically stable patients receiving study treatment after RECIST 1.1-defined PD, tumor assessments MUST continue for the duration of study treatment.											8.1, Appendix F

[a] Every effort should be made to minimize the time between treatment assignment and starting treatment (within 3 days thereafter).

[b] Written informed consent and any locally required privacy act document authorization must be obtained prior to performing any protocol-specific procedures, including screening/baseline evaluations. All patients will be required to provide consent to supply a sample of their tumor (archived [≤3 months old] or recently acquired biopsy) for entry into this study. This consent is included in the main patient ICF. Informed consent of study procedures and tumor biopsy sample may be obtained prior to the 28-day screening window, if necessary. The collection of additional biopsies upon progression is strongly encouraged. If laboratory or imaging procedures were performed for alternate reasons prior to signing consent, these can be used for screening purposes with consent of the patient. However, all screening laboratory and imaging results must have been obtained within 28 days of treatment assignment.

[c] Body weight is recorded at each visit along with vital signs. Height will be measured at screening only. Blood pressure and pulse should be performed before, during and after the first infusion; subsequent assessments should be performed before each infusion.

[d] ECGs will be performed at screening, prior to administration of IMP at C1D1, and every fourth cycle (once) thereafter (eg, C5, C9, C13). Triplicate ECGs will be performed at screening. Subsequent ECGs will be performed in triplicate only if abnormalities are noted. When done in triplicate, ECGs will be taken in close succession, while in a supine/semi-recumbent position. If 12-lead ECG is abnormal, follow institutional guidelines.

[e] Individual sites are required to indicate in the unscheduled visit eCRF if an ECG or a urinalysis was performed during study treatment.

[f] A MUGA scan or ECHO to assess LVEF will be conducted at screening and before treatment on D1 of C5 and then every 4 cycles (±7 days) (eg, Cycle 5, 9, 13…). The modality of the cardiac function assessments must be consistent within patient (ie, if ECHO is used for the screening assessment, then ECHO should also be used for subsequent scans if required). The patients should also be examined using the same machine and operator whenever possible.

[g] [redacted] should be obtained within [redacted] days prior to Cycle 1 Day 1. [redacted] should be obtained before and after each infusion. [redacted] hould be evaluated by PI or the delegate physician prior to the administration of IP at each visit.

[h] PFT as a minimum should include spirometry. Minimum requirement of: FVC (L), FVC% predicted, FEV1 (L), FEV1% predicted, FEV1/FVC%. Optional components to include PEF, FEV6, TLC, DLCO. DLCO will be performed (when feasible), but for patients with prior severe and/or clinically significant pulmonary disorders, DLCO is a requirement.

[i] [redacted].

j Serum or plasma clinical chemistry (including LFT monitoring), hematology, and coagulation assessments may be performed more frequently if clinically indicated and can be done up to 2 days prior to treatment administration. At screening, serum chemistry will include fasting plasma glucose. Subsequent measurements of glucose can be either fasting or non-fasting plasma glucose measurement.

k If screening clinical chemistry, hematology, and coagulation assessments are performed within 3 days prior to D1 of C1 (first infusion day), they do not need to be repeated at D1C1.

l Free T3 or free T4 will only be measured if TSH is abnormal or if there is clinical suspicion of an AE related to the endocrine system.

m If TSH is measured within 14 days prior to D1 (first infusion day), it does not need to be repeated at D1.

n The site will specify the type of test used and will provide appropriate laboratory reference range. The preferred method is urine dipstick.

o Collect blood samples for troponin (preferably high-sensitivity troponin-T) at the timepoints indicated and if at any time a patient reports signs or symptoms suggesting congestive heart failure, myocardial infarction, or other causes of myocyte necrosis. Samples collected on infusion days should be collected at the end of infusion.

p For women of childbearing potential only. A urine or serum pregnancy test is acceptable. Women of childbearing potential are required to have a serum pregnancy test within 7 days prior to the first dose of study drug and then a urine or serum pregnancy test every cycle (q3w). Pregnancy test may occur on D1, but the results must be available and reviewed by the treating physician or Investigator prior to commencing an infusion.

q [redacted]

r [redacted]

s [redacted]

u All AEs and SAEs [redacted] will be collected from the time of signature of the ICF throughout the treatment period and including the safety follow-up (which is [redacted] after the discontinuation of all IPs or until initiation of another therapy). [redacted] If an event that starts post the defined safety follow up period noted above is considered to be due to a late onset toxicity to study treatment, then it should be reported as an AE or SAE as applicable.

v Study visits will occur on D15 of C1 and C2 and will include the indicated assessments (vital signs and clinical chemistry and hematology laboratories). On D15 of C3, the form of contact and procedures conducted will be at the Investigator's discretion.

w DS-8201a will be administered first, followed by durvalumab. It is recommended that patients receive prophylactic anti-emetic agents prior to infusion of DS-8201a and on subsequent days (See 6.1.1.6 for details). During the combination portion of treatment, the durvalumab infusion will start approximately [redacted] after the end of the infusion of DS-8201a. If there are no clinically significant infusion reactions within the first cycle, and at the discretion of the Investigator, then for all other cycles, durvalumab can be administered after at least [redacted] after the infusion of DS-8201a has finished.

x DS-8201a doses can have a ±2-day dosing window (except C1D1).

y Results for LFTs, electrolytes, complete blood count, and creatinine must be available before commencing an infusion (within 2 days C2 onwards) and reviewed by the treating physician or Investigator prior to dosing.

z [redacted]

aa [redacted]

bb This blood sample is only taken [redacted] and **not** at the beginning of each cycle from C6 onward.

cc [redacted]

dd RECIST 1.1 assessments will be performed on images from CT (preferred) or MRI, each preferably with IV contrast, of the chest and abdomen (including the entire liver and both adrenal glands). Additional anatomy (eg, pelvis) should be imaged based on signs and symptoms of individual patients at baseline and follow-up. Digital copies of all

scans (scheduled and unscheduled) must be stored at the investigative site as source documents. For clinically stable patients receiving study treatment after RECIST 1.1-defined PD, a follow-up scan is collected no earlier than 4 weeks after and no later than the next regularly scheduled imaging visit after the prior RECIST 1.1-defined PD, and this scan is evaluated using the criteria for evaluation of scans collected after a RECIST 1.1-defined PD outlined in Appendix F. If an unscheduled assessment was performed and the patient has not progressed, every attempt should be made to perform the subsequent assessments at their next regularly scheduled visit. See Section 6.1.3, Section 8.1, and Appendix F for additional details relevant to tumor assessments.

Note: All assessments on treatment days are to be performed prior to infusion, unless otherwise indicated.

ADA Anti-drug antibodies; AE Adverse event; C Cycle; CT Computed tomography; [redacted] D Day; DLCO Diffusion capacity of the lungs for carbon monoxide; [redacted] DS-8201a Trastuzumab deruxtecan; ECG Electrocardiogram; ECHO Echocardiogram; ECOG Eastern Cooperative Oncology Group; eCRF Electronic case report form; EOT End of treatment; FEV Forced expiratory volume; FFPE Formalin-fixed and paraffin-embedded; FVC Forced vital capacity; HbA1c Glycosylated hemoglobin; HIV Human immunodeficiency virus; HRCT High-resolution computed tomography; ICF Informed consent form; IV Intravenous; IMP Investigational medicinal product; LFT Liver function test; LVEF Left ventricular ejection fraction; MRI Magnetic resonance imaging; MUGA Multiple gated acquisition; PD Progression of disease; PEF Peak expiratory flow; PFT Pulmonary function test; PK Pharmacokinetic; q3w Every 3 weeks; q12w Every 12 weeks; RECIST Response Evaluation Criteria In Solid Tumors; [redacted]; SAE Serious adverse event; T3 Triiodothyronine; T4 Thyroxine; T-DXd Trastuzumab deruxtecan; TLC Total lung capacity; TSH Thyroid-stimulating hormone; WHO World Health Organization.

Table 5 Arm 7 (durvalumab + Dato-DXd [datopotamab deruxtecan; DS-1062a]): Schedule of activities for screening and treatment periods (applies to Parts 1 and 2 of the study)

	Screening	C1			C2			C3		C4 to PD	Final visit	For details, see Section
**Week**	**-4 to -1**	**0**		**1**	**3**		**4**	**6**		**q3w, unless dosing needs to be held for toxicity reasons**	**EOT visit**	
**Cycle Day**	**-28 to -1**	**1[a]**		**8**	**1**		**8**	**1**		**1**		
**Infusion**	**-28 to -1**	**BI**	**EOI**		**BI**	**EOI**		**BI**	**EOI**			
**Window (days)**				±3	±3		±3	±3		±3	±7	
**Informed consent**												
Informed consent: study procedures[b]	X											5.1
Informed consent: [redacted] collection and analysis (optional)	[redacted]											5.1
**Study procedures**												
Eligibility criteria	X											5.1, 5.2
Demography, including baseline characteristics	X											5.1
Physical examination (full)[c]	X											8.2.2
[redacted]												8.2.9.3
Targeted physical examination (based on symptoms)		X			X			X		X	X	8.2.2
Vital signs[c]	X	X	X	X	X	X	X	X	X	X	X	8.2.3
12-lead ECG[d,e]	X	As clinically indicated									X	8.2.4

	Screening	C1			C2			C3		C4 to PD	Final visit	For details, see Section
**Week**	**-4 to -1**	**0**		**1**	**3**		**4**	**6**		**q3w, unless dosing needs to be held for toxicity reasons**	**EOT visit**	
**Cycle Day**	**-28 to -1**	**1[a]**		**8**	**1**		**8**	**1**		**1**		
**Infusion**	**-28 to -1**	**BI**	**EOI**		**BI**	**EOI**		**BI**	**EOI**			
**Window (days)**				±3	±3		±3	±3		±3	±7	
MUGA scan or ECHO (for LVEF evaluation)[f]	X	As clinically indicated									X	8.2.5
PFT[g]	X											8.2.9
Pulmonary HRCT (or CT if HRCT is contraindicated) as per institutional guidelines	X											8.2.9
[redacted]												8.2.9
[redacted]												8.2.9
												6.1.1.7
Concomitant medications	<-->											6.4
**Laboratory assessments**												
Clinical chemistry[k]	X	X[l]		X	X		X	X		X	X	Table 23
Hematology[k]	X	X[l]		X	X		X	X		X	X	Table 24
Coagulation[k]	X	X[l]		As clinically indicated							X	Table 25
TSH (reflex free T3 or free T4)[m]	X	X[n]			X			X		X	X	Table 23
Urinalysis[e,o]	X	As clinically indicated										Table 26
Hepatitis B and C and HIV	X											8.2.1

	Screening	C1			C2			C3		C4 to PD	Final visit	For details, see Section
**Week**	**-4 to -1**	**0**		**1**	**3**		**4**	**6**		**q3w, unless dosing needs to be held for toxicity reasons**	**EOT visit**	
**Cycle Day**	**-28 to -1**	**1[a]**		**8**	**1**		**8**	**1**		**1**		
**Infusion**	**-28 to -1**	**BI**	**EOI**		**BI**	**EOI**		**BI**	**EOI**			
**Window (days)**				±3	±3		±3	±3		±3	±7	
Pregnancy test[p]	X	X			X			X		X	X	8.2.1
**Pharmacokinetics (Durvalumab Part 1 only; Dato-DXd Parts 1 and 2)**												
CCI [redacted]												8.5
												8.5
**Monitoring**												
WHO/ECOG performance status	X	X			X			X		X	X	8.2.8
AE/SAE assessment[u]	<--->											8.3
Patient follow-up contact/patient review for safety		On D15 of C1, C2, and C3[v]										8.2.6
**IP administration**												
Dato-DXd[w,x]		X			X			X		X		6.1.1.7<br>6.1.2.6
Durvalumab [w,y]		X			X			X		X		6.1.1.1, 6.1.1.2, 6.1.2.6

	**Screening**	**C1**			**C2**			**C3**		**C4 to PD**	**Final visit**	**For details, see Section**
**Week**	**-4 to -1**	**0**		**1**	**3**		**4**	**6**		**q3w, unless dosing needs to be held for toxicity reasons**	**EOT visit**	
**Cycle Day**	**-28 to -1**	**1[a]**		**8**	**1**		**8**	**1**		**1**		
**Infusion**	**-28 to -1**	**BI**	**EOI**		**BI**	**EOI**		**BI**	**EOI**			
**Window (days)**				**±3**	**±3**		**±3**	**±3**		**±3**	**±7**	
**Other assessments and assays**												
[redacted]												8.5
[redacted]												8.5
FFPE tumor sample (mandatory; a recently acquired sample is preferred if available per site's routine clinical practice; otherwise, archival sample ≤3 months old can be provided)[b]	X											8.8
[redacted]												8.8

	Screening	C1			C2			C3		C4 to PD	Final visit	For details, see Section
**Week**	-4 to -1	0		1	3		4	6		q3w, unless dosing needs to be held for toxicity reasons	EOT visit	
**Cycle Day**	-28 to -1	1[a]		8	1		8	1		1		
**Infusion**	-28 to -1	BI	EOI		BI	EOI		BI	EOI			
**Window (days)**				±3	±3		±3	±3		±3	±7	
CCI [redacted]												8.8
[redacted]												8.8
[redacted]												8.8
[redacted]												8.8
[redacted]												8.8.2
[redacted]												8.8
[redacted]												8.7

	Screening	C1			C2			C3		C4 to PD	Final visit	For details, see Section
**Week**	**-4 to -1**	**0**		**1**	**3**		**4**	**6**		**q3w, unless dosing needs to be held for toxicity reasons**	**EOT visit**	
**Cycle Day**	**-28 to -1**	**1[a]**		**8**	**1**		**8**	**1**		**1**		
**Infusion**	**-28 to -1**	**BI**	**EOI**		**BI**	**EOI**		**BI**	**EOI**			
**Window (days)**				±3	±3		±3	±3		±3	±7	
**Efficacy evaluations**												
Tumor assessments (CT or MRI) (RECIST 1.1)[dd]	X	On-study tumor assessments occur q6w ±1 week for the first 48 weeks (relative to the date of treatment assignment) and then q12w ±1 week thereafter until RECIST 1.1-defined radiological progression plus an additional follow-up scan or until the patient has been taken off-study (end of study, death, or patient withdrawal of consent). The on-study schedule of q6w ±1 week for the first 48 weeks and then q12w ±1 week thereafter until progression MUST be followed regardless of any delays in dosing. For clinically stable patients receiving study treatment after RECIST 1.1-defined PD, tumor assessments MUST continue for the duration of study treatment.										8.1, Appendix F

a Every effort should be made to minimize the time between treatment assignment and starting treatment (within 3 days thereafter).

b Written informed consent and any locally required privacy act document authorization must be obtained prior to performing any protocol-specific procedures, including screening/baseline evaluations. All patients will be required to provide consent to supply a sample of their tumor (archived [≤3 months old] or recently acquired biopsy) for entry into this study. This consent is included in the main patient ICF. Informed consent of study procedures and tumor biopsy sample may be obtained prior to the 28-day screening window, if necessary. The collection of additional biopsies upon progression is strongly encouraged. If laboratory or imaging procedures were performed for alternate reasons prior to signing consent, these can be used for screening purposes with consent of the patient. However, all screening laboratory and imaging results must have been obtained within 28 days of treatment assignment.

c Body weight is recorded at each visit along with vital signs. Height will be measured at screening only.
Blood pressure and pulse are to be performed before, during and after the first infusion; before and after subsequent infusions at Day 1 and EOT.

d ECG will be taken in triplicate at screening and if an abnormality is noted. ECGs will be taken in close succession, while in a supine/semi-recumbent position. Single ECG will be performed at EOT and as clinically indicated during treatment.

e Individual sites are required to indicate in the unscheduled visit eCRF if an ECG or a urinalysis was performed during study treatment.

f A MUGA scan or ECHO to assess LVEF will be conducted at screening, as clinically indicated during treatment, and at EOT. The modality of the cardiac function assessments must be consistent within patient (ie, if ECHO is used for the screening assessment, then ECHO should also be used for subsequent scans if required). The patients should also be examined using the same machine and operator whenever possible.

g PFT as a minimum should include spirometry. Minimum requirement of FVC (L), FVC% predicted, FEV1 (L), FEV1% predicted, and FEV1/FVC%. Optional components to include PEF, FEV6, TLC, and DLCO. DLCO will be performed (when feasible), but for patients with prior severe and/or clinically significant pulmonary disorders, DLCO is a requirement.

h [redacted] should be obtained within [redacted] prior to C1D1. [redacted] should be obtained before and after each Dato-DXd infusion, at D1 for C1 to C3, prior to infusion from C4 onwards (within [redacted] before infusion), and at EOT. [redacted] should be evaluated by the PI or the delegate physician prior to the administration of IMP at each visit, and at EOT. [redacted] should be evaluated by the PI or the delegate physician prior to the administration of IMP at each visit.

i [redacted]

j [redacted]

k Serum or plasma clinical chemistry (including LFT monitoring), hematology, and coagulation assessments may be performed more frequently if clinically indicated and can be done up to 2 days prior to treatment administration. At screening, serum chemistry will include fasting plasma glucose. Subsequent measurements of glucose can be either fasting or non-fasting plasma glucose measurement.

l If screening clinical chemistry, hematology, and coagulation assessments are performed within 3 days prior to D1 of C1 (first infusion day), they do not need to be repeated at D1C1.

m Free T3 or free T4 will only be measured if TSH is abnormal or if there is clinical suspicion of an AE related to the endocrine system.

n If TSH is measured within 14 days prior to D1 (first infusion day), it does not need to be repeated at D1.

o The site will specify the type of test used and will provide appropriate laboratory reference range. The preferred method is urine dipstick.

p For women of childbearing potential only. Women of childbearing potential are required to have a negative serum pregnancy test within 72 hours before randomization. A positive urine pregnancy test must immediately be confirmed using a serum test. Repeat pregnancy tests (urine or serum test per institutional guideline) will be performed within 72 hours before infusion at each cycle of study intervention, at EOT, and the safety follow-up visit.

q [redacted]

r [redacted]

s [redacted]

t [redacted]

u All AEs and SAEs [redacted] will be collected from the time of signature of the ICF throughout the treatment period and including the safety follow-up (which is [redacted] after the discontinuation of all IPs or until initiation of another therapy). For [redacted] If an event that starts post the defined safety follow-up period noted above is considered to be due to a late onset toxicity to study treatment, then it should be reported as an AE or SAE as applicable. [redacted].

v Study visits will occur on D8 of C1 and C2 and will include the indicated assessments (vital signs and clinical chemistry and hematology laboratories). On D15 of C1 through C3, the form of contact and procedures conducted will be at the Investigator's discretion.

w Dato-DXd will be administered first, followed by durvalumab. It is recommended that patients receive [redacted] prior to infusion of Dato-DXd and on subsequent days (See 6.1.1.7 for details). During the combination portion of treatment, the durvalumab infusion will start approximately [redacted] after the end of the infusion of Dato-DXd. If there are no clinically significant [redacted] within the first cycle, and at the discretion of the Investigator, then for all other cycles, durvalumab can be administered after at least [redacted] after the infusion of Dato-DXd has finished.

x Dato-DXd doses can have a ±2-day dosing window (except C1D1).

y Results for LFTs, electrolytes, complete blood count, and creatinine must be available before commencing an infusion (within 2 days C2 onwards) and reviewed by the treating physician or Investigator prior to dosing.

z [redacted]

aa [redacted]

bb This blood sample is only taken [redacted] and **not** at the beginning of each cycle from C4 onwards.

cc [redacted]

dd RECIST 1.1 assessments will be performed on images from CT (preferred) or MRI, each preferably with IV contrast, of the chest and abdomen (including the entire liver and both adrenal glands). Additional anatomy (eg, pelvis) should be imaged based on signs and symptoms of individual patients at baseline and follow-up. Digital copies of all scans (scheduled and unscheduled) must be stored at the investigative site as source documents. For clinically stable patients receiving study treatment after RECIST 1.1-defined PD, a follow-up scan is collected no earlier than 4 weeks after and no later than the next regularly scheduled imaging visit after the prior RECIST 1.1-defined PD, and this scan is evaluated using the criteria for evaluation of scans collected after a RECIST 1.1-defined PD outlined in Appendix F. If an unscheduled assessment was performed and the patient has not progressed, every attempt should be made to perform the subsequent assessments at their next regularly scheduled visit. See Section 6.1.3, Section 8.1, and Appendix F for additional details relevant to tumor assessments.

Note: All assessments on treatment days are to be performed prior to infusion, unless otherwise indicated.

ADA Anti-drug antibodies; AE Adverse event; BI Before infusion; C Cycle; CT Computed tomography; [redacted] COVID-19 Coronavirus disease 2019-nCoV; D Day; Dato-DXd Datopotamab deruxtecan; DS-1062a Datopotamab deruxtecan; ECG Electrocardiogram; ECHO Echocardiogram; ECOG Eastern Cooperative Oncology Group; eCRF Electronic case report form; EOI End of infusion; EOT End of treatment; FFPE Formalin-fixed and paraffin-embedded; HIV Human immunodeficiency virus; HRCT High-resolution computed tomography; ICF Informed consent form; ILD Interstitial lung disease; IMP Investigational medicinal product; IP Investigational product; IV Intravenous; LFT Liver function test; LVEF Left ventricular ejection fraction; MRI Magnetic resonance imaging; MUGA Multiple gated acquisition; PD Progression of disease; PFT Pulmonary function test; PI Principal investigator; PK Pharmacokinetic; q3w Every 3 weeks; q12w Every 12 weeks; q21 Every 21; RECIST Response Evaluation Criteria In Solid Tumors; [redacted] SAE Serious adverse event; [redacted] [redacted]; T3 Triiodothyronine; T4 Thyroxine; TSH Thyroid-stimulating hormone; WHO World Health Organization.

Table 6 Arm 8 (durvalumab + Dato-DXd [datopotamab deruxtecan; DS-1062a]): Schedule of activities for screening and treatment periods (applies to Parts 1 and 2 of the study)

	Screening	C1			C2			C3		C4 to PD	Final visit	For details, see Section
**Week**	**-4 to -1**	**0**		**1**	**3**		**4**	**6**		**q3w, unless dosing needs to be held for toxicity reasons**	**EOT visit**	
**Cycle Day**	**-28 to -1**	**1[a]**		**8**	**1**		**8**	**1**		**1**		
**Infusion**	**-28 to -1**	**BI**	**EOI**		**BI**	**EOI**		**BI**	**EOI**			
**Window (days)**				±3	±3		±3	±3		±3	±7	
**Informed consent**												
Informed consent: main study procedures[b]	X											5.1
Informed consent: [redacted] collection and analysis (optional)	[redacted]											5.1
**Study procedures**												
Eligibility criteria	X											5.1, 5.2
Demography, including baseline characteristics	X											5.1
Physical examination (full)[c]	X											8.2.2
[redacted]												8.2.9.3
Targeted physical examination (based on symptoms)		X			X			X		X	X	8.2.2
Vital signs[c]	X	X	X	X	X	X	X	X	X	X	X	8.2.3
12-lead ECG[d,e]	X	As clinically indicated									X	8.2.4
MUGA scan or ECHO (for LVEF evaluation)[f]	X	As clinically indicated									X	8.2.5
PFT[g]	X											8.2.9

	Screening	C1			C2			C3		C4 to PD	Final visit	For details, see Section
**Week**	**-4 to -1**	**0**		**1**	**3**		**4**	**6**		**q3w, unless dosing needs to be held for toxicity reasons**	**EOT visit**	
**Cycle Day**	**-28 to -1**	**1[a]**		**8**	**1**		**8**	**1**		**1**		
**Infusion**	**-28 to -1**	**BI**	**EOI**		**BI**	**EOI**		**BI**	**EOI**			
**Window (days)**				**±3**	**±3**		**±3**	**±3**		**±3**	**±7**	
Pulmonary HRCT (or CT if HRCT is contraindicated) as per institutional guidelines	X											8.2.9
[redacted]												8.2.9
[redacted]												8.2.9
												6.1.1.7
Concomitant medications	<-->											6.4
**Laboratory assessments**												
Clinical chemistry[k]	X	X[l]		X	X		X	X		X	X	Table 23
Hematology[k]	X	X[l]		X	X		X	X		X	X	Table 24
Coagulation[k]	X	X[l]		As clinically indicated							X	Table 25
TSH (reflex free T3 or free T4)[m]	X	X[n]			X			X		X	X	Table 23
Urinalysis[e,o]	X	As clinically indicated										Table 26
Hepatitis B, C, and D and HIV	X											8.2.1
Pregnancy test[p]	X	X			X			X		X	X	8.2.1
**Pharmacokinetics (Durvalumab Part 1 only; Dato-DXd Parts 1 and 2)**												
[redacted]												8.5

	Screening	C1			C2			C3		C4 to PD	Final visit	For details, see Section
**Week**	**-4 to -1**	**0**		**1**	**3**		**4**	**6**		**q3w, unless dosing needs to be held for toxicity reasons**	**EOT visit**	
**Cycle Day**	**-28 to -1**	**1[a]**		**8**	**1**		**8**	**1**		**1**		
**Infusion**	**-28 to -1**	**BI**	**EOI**		**BI**	**EOI**		**BI**	**EOI**			
**Window (days)**				**±3**	**±3**		**±3**	**±3**		**±3**	**±7**	
CCI [redacted]												8.5
**Monitoring**												
WHO/ECOG performance status	X	X			X			X		X	X	8.2.8
AE/SAE assessment[u]	<--->											8.3
Patient follow-up contact/patient review for safety		On D15 of C1, C2, and C3[v]										8.2.6
**IP administration**												
Dato-DXd[w,x]		X			X			X		X		6.1.1.7<br>6.1.2.6
Durvalumab [w,y]		X			X			X		X		6.1.1.1, 6.1.1.2, 6.1.2.6
**Other assessments and assays**												
CCI [redacted]												8.5

	Screening	C1			C2			C3		C4 to PD	Final visit	For details, see Section
**Week**	**-4 to -1**	**0**		**1**	**3**		**4**	**6**		**q3w, unless dosing needs to be held for toxicity reasons**	**EOT visit**	
**Cycle Day**	**-28 to -1**	**1[a]**		**8**	**1**		**8**	**1**		**1**		
**Infusion**	**-28 to -1**	**BI**	**EOI**		**BI**	**EOI**		**BI**	**EOI**			
**Window (days)**				**±3**	**±3**		**±3**	**±3**		**±3**	**±7**	
CCI [redacted]												8.5
FFPE tumor sample (mandatory; a recently acquired sample is preferred if available per site's routine clinical practice; otherwise, archival sample ≤3 months old can be provided)[b]	X											8.8
CCI [redacted]												8.8
												8.8
												8.8
												8.8
												8.8
												8.8.2

	Screening	C1			C2			C3		C4 to PD	Final visit	For details, see Section
**Week**	**-4 to -1**	**0**		**1**	**3**		**4**	**6**		**q3w, unless dosing needs to be held for toxicity reasons**	**EOT visit**	
**Cycle Day**	**-28 to -1**	**1[a]**		**8**	**1**		**8**	**1**		**1**		
**Infusion**	**-28 to -1**	**BI**	**EOI**		**BI**	**EOI**		**BI**	**EOI**			
**Window (days)**				**±3**	**±3**		**±3**	**±3**		**±3**	**±7**	
CCI [redacted]												8.8
												8.7
**Efficacy evaluations**												
Tumor assessments (CT or MRI) (RECIST 1.1)[dd]	X	On-study tumor assessments occur q6w ±1 week for the first 48 weeks (relative to the date of treatment assignment) and then q12w ±1 week thereafter until RECIST 1.1-defined radiological progression plus an additional follow-up scan or until the patient has been taken off-study (end of study, death, or patient withdrawal of consent). The on-study schedule of q6w ±1 week for the first 48 weeks and then q12w ±1 week thereafter until progression MUST be followed regardless of any delays in dosing. For clinically stable patients receiving study treatment after RECIST 1.1-defined PD, tumor assessments MUST continue for the duration of study treatment.										8.1, Appendix F

a Every effort should be made to minimize the time between treatment assignment and starting treatment (within 3 days thereafter).

b Written informed consent and any locally required privacy act document authorization must be obtained prior to performing any protocol-specific procedures, including screening/baseline evaluations. All patients will be required to provide consent to supply a sample of their tumor (archived [≤3 months old] or recently acquired biopsy) for entry into this study. This consent is included in the main patient ICF. Informed consent of study procedures and tumor biopsy sample may be obtained prior to the 28-day screening window, if necessary. The collection of additional biopsies upon progression is strongly encouraged. If laboratory or imaging procedures were performed for alternate reasons prior to signing consent, these can be used for screening purposes with consent of the patient. However, all screening laboratory and imaging results must have been obtained within 28 days of treatment assignment.
A pre-screen ICF is to be signed by patients in Arm 8 who do not have a prior determination of positive PD-L1 status, to permit determination of PD-L1 expression status by local testing prior to the 28-day screening window. At the time of signing the pre-screen ICF, Investigators should ensure that there is a reasonable possibility that the patient would be a candidate for this study based on available information. When a pre-screen ICF is signed, the main ICF should not be signed until PD-L1 positive expression status has been established. Patients whose tumor is not found to express PD L1 will discontinue from the study.

c Body weight is recorded at each visit along with vital signs. Height will be measured at screening only.
Blood pressure and pulse are to be performed before, during and after the first infusion; before and after subsequent infusions at Day 1 and EOT.

d ECG will be taken in triplicate at screening and if an abnormality is noted. ECGs will be taken in close succession, while in a supine/semi-recumbent position. Single ECG will be performed at EOT and as clinically indicated during treatment.

e Individual sites are required to indicate in the unscheduled visit eCRF if an ECG or a urinalysis was performed during study treatment.

f A MUGA scan or ECHO to assess LVEF will be conducted at screening, as clinically indicated during treatment, and at EOT. The modality of the cardiac function assessments must be consistent within patient (ie, if ECHO is used for the screening assessment, then ECHO should also be used for subsequent scans if required). The patients should also be examined using the same machine and operator whenever possible.

g PFT as a minimum should include spirometry. Minimum requirement of FVC (L), FVC% predicted, FEV1 (L), FEV1% predicted, and FEV1/FVC%. Optional components to include PEF, FEV6, TLC, and DLCO. DLCO will be performed (when feasible), but for patients with prior severe and/or clinically significant pulmonary disorders, DLCO is a requirement.

h [redacted] should be obtained within [redacted] prior to C1D1. [redacted] should be obtained before and after each Dato-DXd infusion, at D1 for C1 to C3, prior to infusion from C4 onwards (within [redacted] before infusion), and at EOT. [redacted] should be evaluated by the PI or the delegate physician prior to the administration of IMP at each visit, and at EOT.

i [redacted]

j [redacted]

k Serum or plasma clinical chemistry (including LFT monitoring), hematology, and coagulation assessments may be performed more frequently if clinically indicated and can be done up to 2 days prior to treatment administration. At screening, serum chemistry will include fasting plasma glucose. Subsequent measurements of glucose can be either fasting or non-fasting plasma glucose measurement.

l If screening clinical chemistry, hematology, and coagulation assessments are performed within 3 days prior to D1 of C1 (first infusion day), they do not need to be repeated at D1C1.

m Free T3 or free T4 will only be measured if TSH is abnormal or if there is clinical suspicion of an AE related to the endocrine system.

n If TSH is measured within 14 days prior to D1 (first infusion day), it does not need to be repeated at D1.

o The site will specify the type of test used and will provide appropriate laboratory reference range. The preferred method is urine dipstick.

p For women of childbearing potential only. Women of childbearing potential are required to have a negative serum pregnancy test within 72 hours before randomization. A positive urine pregnancy test must immediately be confirmed using a serum test. Repeat pregnancy tests (urine or serum test per institutional guideline) will be performed within 72 hours before infusion at each cycle of study intervention, at EOT, and the safety follow-up visit.

q [redacted]

r

s

t

u For patients who sign the pre-screening ICF, only SAEs will be reported during the pre-screening period. All AEs and SAEs [redacted] will be collected from the time of signature of the main ICF throughout the treatment period and including the safety follow-up (which is [redacted] after the discontinuation of all IPs or until initiation of another therapy). [redacted]. If an event that starts post the defined safety follow-up period noted above is considered to be due to a late onset toxicity to study treatment, then it should be reported as an AE or SAE as applicable. [redacted]

v Study visits will occur on D8 of C1 and C2 and will include the indicated assessments (vital signs and clinical chemistry and hematology laboratories). On D15 of C1 through C3, the form of contact and procedures conducted will be at the Investigator's discretion.

[w] Dato-DXd will be administered first, followed by durvalumab. It is recommended that patients receive [redacted] prior to infusion of Dato-DXd and on subsequent days (See 6.1.1.7 for details). During the combination portion of treatment, the durvalumab infusion will start approximately [redacted] after the end of the infusion of Dato-DXd. If there are no clinically significant [redacted] within the first cycle, and at the discretion of the Investigator, then for all other cycles, durvalumab can be administered after at least [redacted] after the infusion of Dato-DXd has finished.

[x] Dato-DXd doses can have a ±2-day dosing window (except C1D1).

[y] Results for LFTs, electrolytes, complete blood count, and creatinine must be available before commencing an infusion (within 2 days C2 onwards) and reviewed by the treating physician or Investigator prior to dosing.

[z] [redacted]

[aa] [redacted]

[bb] This blood sample is only taken [redacted] and **not** at the beginning of each cycle from C4 onwards.

[cc] [redacted]

[dd] RECIST 1.1 assessments will be performed on images from CT (preferred) or MRI, each preferably with IV contrast, of the chest and abdomen (including the entire liver and both adrenal glands). Additional anatomy (eg, pelvis) should be imaged based on signs and symptoms of individual patients at baseline and follow-up. Digital copies of all scans (scheduled and unscheduled) must be stored at the investigative site as source documents. For clinically stable patients receiving study treatment after RECIST 1.1-defined PD, a follow-up scan is collected no earlier than 4 weeks after and no later than the next regularly scheduled imaging visit after the prior RECIST 1.1-defined PD, and this scan is evaluated using the criteria for evaluation of scans collected after a RECIST 1.1-defined PD outlined in Appendix F. If an unscheduled assessment was performed and the patient has not progressed, every attempt should be made to perform the subsequent assessments at their next regularly scheduled visit. See Section 6.1.3, Section 8.1, and Appendix F for additional details relevant to tumor assessments.

Note: All assessments on treatment days are to be performed prior to infusion, unless otherwise indicated.

ADA Anti-drug antibodies; AE Adverse event; BI Before infusion; C Cycle; CT Computed tomography; [redacted]; COVID-19 Coronavirus disease 2019-nCoV; D Day; Dato-DXd Datopotamab deruxtecan; DS-1062a Datopotamab deruxtecan; ECG Electrocardiogram; ECHO Echocardiogram; ECOG Eastern Cooperative Oncology Group; eCRF Electronic case report form; EOI End of infusion; EOT End of treatment; FFPE Formalin-fixed and paraffin-embedded; HIV Human immunodeficiency virus; HRCT High-resolution computed tomography; ICF Informed consent form; ILD Interstitial lung disease; IMP Investigational medicinal product; IP Investigational product; IV Intravenous; LFT Liver function test; LVEF Left ventricular ejection fraction; MRI Magnetic resonance imaging; MUGA Multiple gated acquisition; PD Progression of disease; PFT Pulmonary function test; PI Principal investigator; PK Pharmacokinetic; q3w Every 3 weeks; q12w Every 12 weeks; q21 Every 21; RECIST Response Evaluation Criteria In Solid Tumors; [redacted] SAE Serious adverse event; [redacted] T3 Triiodothyronine; T4 Thyroxine; TSH Thyroid-stimulating hormone; WHO World Health Organization.

Table 7 Schedule of assessments for patients who have completed/discontinued treatment (applies to Parts 1 and 2)

	Time since last dose of IP						
**Evaluations**	**Day (±3)**	**Months (±1 week)**				**12 months and every 3 months (±2 weeks)**	**For details, see Section**
	**30**	**2**	**3**	**6**	**9**		
Physical examination (full)	X						8.2.2
Vital signs (temperature, respiratory rate, blood pressure, and pulse)	X						8.2.3
MUGA scan or ECHO (for LVEF evaluation) [a]	X						8.2.5
Weight	X	X	X				8.2.3
Pregnancy test[b]	X	As clinically indicated					8.2.1
AE/SAE assessment	X	X	X				8.3
Concomitant medications	X	X	X				6.4
WHO/ECOG performance status	At timepoints consistent with tumor assessments; at [redacted], [redacted] [redacted] days; and then at initiation of subsequent anticancer therapy[c]						8.2.8
Subsequent anticancer therapy[d]	<-->						8.1
Survival status[e]			X	X	X	X	8.1.1
Hematology	X	X	X				Table 24
Clinical chemistry	X	X	X				Table 23
TSH (reflex free T3 or free T4[f])	X	X	X				Table 23
[redacted]							8.5
							8.5
							8.5

	**Time since last dose of IP**						
**Evaluations**	**Day (±3)**	**Months (±1 week)**				**12 months and every 3 months (±2 weeks)**	**For details, see Section**
	**30**	**2**	**3**	**6**	**9**		
CCI [redacted]							8.5
[redacted]							8.5
Tumor assessment (CT or MRI) (RECIST 1.1) [p]	On-study tumor assessments occur q8w (or q6w for DS-8201a and Dato-DXd) ±1 week for the first 48 weeks (relative to the date of treatment assignment) and then q12w ±1 week thereafter until RECIST 1.1 defined radiological progression (plus an additional follow up scan for patients on Arms 7 and 8) or until the patient has been taken off-study (end of study, death, or patient withdrawal of consent). The on-study schedule of q8w (or q6w for DS-8201a and Dato-DXd) ±1 week (relative to the date of treatment assignment) for the first 48 weeks and then q12w ±1 week thereafter MUST be followed regardless of any delays in dosing. Additional scans to be completed per standard practice post-progression.						8.1, Appendix F

[a] The modality of the cardiac function assessments must be consistent within patient (ie, if ECHO is used for the screening assessment, then ECHO should also be used for the follow-up scan). The patients should also be examined using the same machine and operator whenever possible.

[b] For women of childbearing potential only. A urine or serum pregnancy test is acceptable.

[c] WHO/ECOG performance status should also be collected at other site visits that the patient attends, if appropriate site staff are available to collect such information. In addition, WHO/ECOG performance status should be provided when information on subsequent anticancer therapy is provided, where possible.

[d] Details of any treatment for TNBC (including surgery) post the last dose of IP must be recorded in the eCRF. At minimum, collect the start date and description of the subsequent anticancer therapy.

[e] Patients may be contacted in the week following data cutoffs to confirm survival status.

[f] Free T3 or free T4 will only be measured if TSH is abnormal or if there is clinical suspicion of an AE related to the endocrine system.

[g] CCI [redacted]

[l] Immunogenicity assessments will occur only in Part 1 of the study. Immunogenicity samples are collected 90 days (3 months) (±7 days) after the last dose of durvalumab.

[m] Immunogenicity assessments will occur only in Part 1 of the study for Arm 5 only. Immunogenicity samples are collected 90 days (3 months) (±7 days) after the last dose of oleclumab. In addition, a final immunogenicity sample is taken 6 months (±7 days) after treatment ends.

[n] Immunogenicity assessments will occur only in Part 1 of the study for Arm 6 only. Immunogenicity samples are collected 90 days (3 months) (±7 days) after the last dose of DS-8201a. In addition, a final immunogenicity sample is taken 6 months (±7 days) after treatment ends.

[o] Immunogenicity assessments will occur in Parts 1 and 2 of the study for Arms 7 and 8 only. Immunogenicity samples are collected 90 days (3 months) (±7 days) after the last dose of Dato-DXd.

[p] RECIST 1.1 assessments will be performed on images from CT (preferred) or MRI, each preferably with IV contrast, of the chest and abdomen (including the entire liver and both adrenal glands). Additional anatomy (eg, pelvis) should be imaged based on signs and symptoms of individual patients at baseline and follow-up. Digital copies of all scans (scheduled and unscheduled) must be stored at the investigative site as source documents. If an unscheduled assessment was performed and the patient has not progressed, every attempt should be made to perform the subsequent assessments at their next regularly scheduled visit. See Section 8.1, and Appendix F for additional details relevant to tumor assessments.

ADA Anti-drug antibodies; AE Adverse event; CT Computed tomography; Dato-DXd Datopotamab deruxtecan; DS-8201a Trastuzumab deruxtecan; ECHO Echocardiogram; ECOG Eastern Cooperative Oncology Group; eCRF Electronic case report form; IP Investigational product; LVEF Left ventricular ejection fraction; MRI Magnetic resonance imaging; MUGA Multiple gated acquisition; PD Progression of disease; PK Pharmacokinetics; q8w Every 8 weeks; q12w Every 12 weeks; RECIST Response Evaluation Criteria in Solid Tumors; SAE Serious adverse event; T3 Triiodothyronine; T4 Thyroxine; TSH Thyroid-stimulating hormone; TNBC Triple negative breast cancer; WHO World Health Organization.

1.2 Synopsis

International Co-ordinating Investigator

Peter Schmid, MD, PhD
Barts Cancer Institute
Barts and The London School of Medicine and Dentistry, Queen Mary University of London
Charterhouse Square, London EC1M 6BQ
p.schmid@qmul.ac.uk

Protocol Title: A Phase IB/II, 2-stage, open-label, multicenter study to determine the efficacy and safety of durvalumab (MEDI4736) in combination with novel oncology therapies with or without paclitaxel and durvalumab (MEDI4736) + paclitaxel for first-line metastatic triple negative breast cancer

Short Title: Phase IB/II, 2-stage study of oncology combinations in first-line Stage IV TNBC

Rationale:

No targeted therapies currently exist for triple negative breast cancer (TNBC), the subtype of breast cancer (BC) characterized by a lack of tumor expression of estrogen receptor, progesterone receptor, and human epidermal growth factor receptor 2. Among all early-stage BC subtypes, TNBC has the highest probability of relapse and portends the poorest overall survival (OS). Metastatic TNBC has an aggressive clinical course, marked by more frequent tumor progression than for early-stage TNBC as well as shorter progression-free survival (PFS) and OS. Affected patients are managed by serial administration of single-agent chemotherapeutics, generally with AEs that impact quality of life and poor clinical outcomes. Patients with TNBC have a poor prognosis and limited treatment options and thus represent an unmet medical need.

Given the broad success of immune checkpoint inhibitors in providing durable clinical response in multiple cancer types, the utility of programmed cell death 1 (PD-1)/programmed cell death ligand 1 (PD-L1) blockade in BC is under active investigation. Encouraging responses have been seen in patients with high PD-L1 tumor expression (PD-L1[+] patients), particularly as a first-line treatment, but this population represents only a subset of all patients with metastatic TNBC. There is a strong rationale for adding PD-1/PD-L1 blockade to chemotherapy. In particular, paclitaxel monotherapy has been shown to increase the proportion of tumor-infiltrating lymphocytes (TILs) when given to patients with BC, thus promoting a more immunogenic environment. Studies to date that combine checkpoint inhibitors and chemotherapy have shown improved objective response rate (ORR) compared with checkpoint inhibitor monotherapy. However, for the substantial proportion of patients who do not derive benefit from the combination of chemotherapy and PD-1/PD-L1 inhibition, developing a novel treatment combination with other immunotherapies or targeted therapies is of particular interest. In this Phase IB/II study, novel oncology therapies designed for immune

modulation are being tested in combination with durvalumab (PD-L1 blocker) and paclitaxel (chemotherapy); a durvalumab + paclitaxel arm, a durvalumab + DS-8201a (trastuzumab deruxtecan, also referred to as T-DXd) combination arm, and a durvalumab + Dato-DXd (datopotamab deruxtecan, also referred to as DS-1062a) combination arm will also be evaluated. Durvalumab + Dato-DXd will also be evaluated in PD-L1 positive patients. Modalities with promise for BC and therapies with available clinical safety and combination data with durvalumab will be prioritized for study.

Objectives and endpoints - Part 1	
**Primary objective:**	**Endpoint/variable:**
To assess the safety and tolerability profile of durvalumab + novel oncology therapies with or without paclitaxel and durvalumab + paclitaxel	AEs, exposure, physical examinations, laboratory findings, [redacted] and vital signs
**Secondary objectives:**	**Endpoint/variable:**
To assess the efficacy of durvalumab + novel oncology therapies with or without paclitaxel and durvalumab + paclitaxel in terms of ORR, PFS, DoR, and OS	Endpoints based on Investigator assessment according to RECIST 1.1:<br>• ORR: The percentage of evaluable patients with a confirmed Investigator-assessed visit response of CR or PR<br>• PFS: Time from date of first dose until the date of objective radiological disease progression using RECIST 1.1 or death (by any cause in the absence of progression)<br>• DoR: Time from date of first detection of objective response (which is subsequently confirmed) until the date of objective radiological disease progression<br>• OS: Time from date of first dose until the date of death by any cause
To assess the PK of durvalumab and novel oncology therapies (ie, oleclumab, DS-8201a, and Dato-DXd) in all treatment arms	Serum concentration of durvalumab and serum or plasma concentration of novel oncology therapies
To investigate the immunogenicity of durvalumab and applicable novel oncology therapies (ie, oleclumab, DS-8201a, and Dato-DXd) in all applicable treatment arms	Presence of ADAs for durvalumab and applicable novel oncology therapies
**Exploratory objectives:**	**Endpoint/variable:**
To collect blood and tissue samples to evaluate molecular/biological responses and/or identify candidate markers that may correlate with the likelihood of clinical benefit	Blood [redacted], including but not limited to the following, and their association with treatment benefit:<br>• TILs detected by H&E<br>• Protein expression detected by IHC<br>• [redacted]<br>• mRNA expression
To further assess the efficacy of durvalumab + novel oncology therapies with or without paclitaxel and durvalumab + paclitaxel in terms of change in tumor size	Change in tumor size

Objectives and endpoints - Part 2[a]	
**Primary objective:**	**Endpoint/variable:**
To assess the efficacy of durvalumab + novel oncology therapies with or without paclitaxel in terms of ORR	Endpoint based on Investigator assessment according to RECIST 1.1:<br>• ORR: The percentage of evaluable patients with an Investigator-assessed visit response of CR or PR. Part 1 and Part 2 data will be pooled for efficacy analysis.
**Secondary objectives:**	**Endpoint/variable:**
To further assess the efficacy of durvalumab + novel oncology therapies with or without paclitaxel in terms of PFS, DoR, OS, and PFS6[b]	Endpoints based on Investigator assessment according to RECIST 1.1:<br>• PFS: Time from date of first dose until the date of objective radiological disease progression using RECIST 1.1 or death (by any cause in the absence of progression)<br>• DoR: Time from date of first detection of objective response until the date of objective radiological disease progression according to RECIST 1.1<br>• PFS6: PFS at 6 months following date of first dose<br>• OS: Time from date of first dose until the date of death by any cause
To assess the safety and tolerability profile of durvalumab + novel oncology therapies with or without paclitaxel	AEs, exposure, physical examinations, laboratory findings, [redacted], and vital signs
**Exploratory objective:**	**Endpoint/variable:**
To collect blood and tissue samples to evaluate molecular/biological responses and/or identify candidate markers that may correlate with the likelihood of clinical benefit	Blood [redacted], including but not limited to the following, and their association with treatment benefit:<br>• TILs detected by H&E<br>• Protein expression detected by IHC<br>• [redacted]<br>• mRNA expression

[a] All mentioned endpoints for Part 2 will be analyzed in the dataset of Part 1 plus Part 2 patients in each cohort and additionally may be analyzed for each part separately.

[b] Progression-free survival at 6 months following date of first dose (PFS6) is equivalent to the proportion of patients alive and progression free at 6 months following date of first dose (APF6).

ADA Anti-drug antibodies; AE Adverse event; CR Complete response; [redacted]; Dato-DXd Datopotamab deruxtecan; DoR Duration of response; H&E Hematoxylin and eosin stain; IHC Immunohistochemistry; mRNA Messenger ribonucleic acid; ORR Objective response rate; OS Overall survival; PFS Progression-free survival; PK Pharmacokinetics; PR Partial response; RECIST 1.1 Response Evaluation Criteria in Solid Tumors, version 1.1; TIL Tumor-infiltrating lymphocyte.

Overall design:

This is a Phase IB/II, 2-stage, open-label, multicenter study to determine the efficacy and safety of durvalumab in combination with novel oncology therapies (ie, capivasertib, oleclumab, DS-8201a [trastuzumab deruxtecan], and Dato-DXd [datopotamab deruxtecan]) with or without paclitaxel and durvalumab + paclitaxel as first-line treatment in patients with metastatic (Stage IV) TNBC. The study is designed to concurrently evaluate potential novel treatment combinations with clinical promise using a 2-stage approach. The study will use a [redacted] design to evaluate which cohorts may proceed to expansion.

The treatment arms are defined in Table 8. Part 1 is a Phase IB study of safety, initial efficacy, and PK/ADA. Part 2 will expand patient enrollment in a particular arm if an adequate efficacy signal is observed for that arm in Part 1. The treatment regimens evaluated in Part 2 will depend on the evaluation of safety and efficacy outcomes in Part 1.

Table 8 Treatment arms

Study part	Arm	Study treatment
1	1	durvalumab + paclitaxel
1	2	durvalumab + paclitaxel + capivasertib
1	3	Removed (Protocol Version 3)
1	4	Removed (Protocol Version 4)
1	5	durvalumab + paclitaxel + oleclumab
1	6	durvalumab + DS-8201a (trastuzumab deruxtecan)
1	7	durvalumab + Dato-DXd (datopotamab deruxtecan)
1	8	durvalumab + Dato-DXd (datopotamab deruxtecan) [a]
2		Treatment arms that meet pre-defined endpoints from Part 1 may be expanded in Part 2[b]

[a] Patients with PD-L1 positive status.
[b] Refer to Section 4.1.3 (Part 2 study design overview).

The primary objective of Part 1 will be safety. The safety and tolerability of each combination will be assessed. Additionally, an efficacy analysis for futility (based on ORR) will be assessed using a [redacted] design to determine enrollment for Part 2. Part 1 of the study will enroll the first 20 patients to the durvalumab + paclitaxel arm and monitor for toxicity; additional patients may be enrolled in order to have 20 evaluable patients. Patient assignment to one of the novel treatment combination arms that is open for enrollment will start after completion of recruitment to the durvalumab + paclitaxel arm. Each subsequent cohort will undergo a safety run-in period, except for Arm 8, given that the durvalumab + Dato-DXd combination was already evaluated in Arm 7 and found to be tolerable with no DLTs. In the

safety run-ins, 6 patients are planned to be enrolled to initially inform the safety profile and determine the combination dose; more or fewer patients may be enrolled, depending on how many patients are evaluable for dose-limiting toxicities (DLTs) and how many patients have DLTs. The novel treatment combination cohorts meeting the safety evaluation criteria (ie, no more than 1 of 6 DLT-evaluable patients experience DLTs during the first treatment cycle) will be expanded to 30 patients. Additional cohorts may be opened at prespecified lower dose levels if the initial doses are not tolerated. Any decision to introduce additional cohorts in order to investigate an alternative dosing regimen will be at the discretion of AstraZeneca after discussion with the Safety Review Committee (SRC). Efficacy assessments based on ORR (as determined by the Investigator according to RECIST 1.1) will be made when each cohort has completed enrollment and all patients have either had the opportunity to complete at least 2 on-treatment response evaluations or have discontinued treatment.

The primary objective of Part 2 will be efficacy, with a primary endpoint of ORR. Efficacy of the durvalumab + novel oncology therapy combinations with or without paclitaxel will be evaluated. A given treatment arm from Part 1 will be analyzed before an arm with the same treatment may be expanded into Part 2, and no patients from Part 1 may enroll in Part 2. Within each study part, there will be no crossover between treatment arms. Initiation of Part 2 will be considered if the safety profile and ORR observed in at least 1 of the novel treatment combination arms in Part 1 of the study meet a threshold criterion for ORR. Any impact on paclitaxel exposure (delays and discontinuations) observed during Part 1 will be considered in the decision.

In both study parts, the imaging schedule will be as follows: response evaluation will be performed using computed tomography (CT) or magnetic resonance imaging (MRI) at screening (as baseline), with follow-up imaging every 8 weeks ±1 week (for Arms 1 through 5 [ie, arms with 4-week cycles]) or every 6 weeks ±1 week (for Arms 6, 7, and 8 [ie, arm with 3-week cycles]) for 48 weeks and every 12 weeks ±1 week thereafter until RECIST 1.1-defined radiological progression or until the patient has been taken off study (end of study, death, or patient withdrawal of consent).

All tumor assessments should be reported on the electronic case report form until enrollment is complete and all patients have had the opportunity to complete at least 2 on-treatment response evaluations; AstraZeneca will inform sites once this milestone has been reached. After data cutoff (DCO) for any treatment arm in Part 1 for ORR, patients must continue to follow the schedules of activities, and all safety assessments will be collected per Section 1.1. Following the final DCO date by study arm, participants will be managed per SoC assessments at Investigator discretion.

Study period:

In both study parts, patients will receive treatment until any discontinuation criteria are met (see Section 7.1), including clinical progression or radiological progression (refer to Appendix F) and Investigator determination that the patient is no longer benefiting from treatment with IP.

Number of patients:

This study will enroll approximately 57 response-evaluable patients (ie, patients eligible to be included in the Response Evaluable Analysis Set) per arm (ie, 30 patients in Part 1 and 27 patients in Part 2 per treatment arm, with the exception of Arm 1; Arm 1 will enroll 20 patients in Part 1 and will not be included in Part 2). The study is sized to allow the use of a CCI [redacted] design within each treatment arm, with the exception of Arm 1.

Part 1: At least 20 patients will be enrolled in the durvalumab + paclitaxel arm and at least 30 patients in each of the other novel treatment combination arms, for a total of approximately 170 patients (durvalumab + paclitaxel arm and 4 novel treatment combination arms). Additional patients may be enrolled in order to have at least 20 or 30 evaluable patients per durvalumab + paclitaxel arm or novel treatment combination arm, respectively.

Part 2: Approximately 27 patients will be assigned to each treatment arm, for an anticipated total of 57 response-evaluable patients (ie, patients eligible to be included in the Response Evaluable Analysis Set) per arm (ie, 30 patients in Part 1 and 27 patients in Part 2 per treatment arm, with the exception of Arm 1, which will enroll 20 patients in Part 1 and will not be included in Part 2). Arm expansion from 30 patients in Part 1 to an additional 27 patients from Part 2 will be determined based on a futility analysis utilizing a CCI [redacted] design, and Sponsor decision.

Target patient population:

Patients with locally assessed and locally confirmed advanced/unresectable or metastatic TNBC, as defined by the most recent American Society of Clinical Oncology/College of American Pathologists guidelines, who are ≥18 years of age with Stage IV breast adenocarcinoma, had no prior systemic treatment for metastatic disease, have a tumor evaluable by RECIST 1.1, and are immunotherapy naïve will be eligible for this study. Patients must provide a tumor sample (archived or per routine clinical care) for determination of PD-L1 expression; however, the results of this assessment will not be used for inclusion/exclusion or stratification purposes. For enrollment into Arm 6 (durvalumab + DS-8201a), patients must provide documentation of locally determined advanced/unresectable or metastatic TNBC with HER2-low tumor expression (IHC 2+/ISH–, IHC 1+/ISH–, or IHC 1+/ISH untested). (Note: ISH may be determined by either fluorescence in situ hybridization [FISH] or dual in situ hybridization [DISH]). For enrollment into Arm 8 (durvalumab + Dato-DXd), patients must provide documentation of locally determined

advanced/unresectable or metastatic TNBC that is PD-L1 positive as determined by a pre-existing test or by local testing during pre-screening.

Treatments and treatment duration:

Durvalumab will be administered according to 28-day cycles (Arms 1 through 5) or 21-day cycle (Arms 6, 7, and 8). The dosage and mode of administration for durvalumab, paclitaxel, and each novel oncology therapy to be evaluated in a novel treatment combination arm are summarized in Table 9.

Please note that if a patient's weight falls to ≤30 kg after initiation of study treatment, then the patient should receive weight-based dosing equivalent to 20 mg/kg (Arms 1 through 5 only) of durvalumab every 4 weeks [q4w] after consultation between the Investigator and the Study Clinical Lead, until the weight improves to >30 kg, at which point the patient should start receiving the fixed dosing of durvalumab 1500 mg every cycle. Arms 6, 7, and 8 would not require weight-based dosing because the weight restriction is due to endotoxin limits per dose and is not applicable for doses <1500 mg.

Table 9 Treatments and treatment duration

Target	Compound	Dose	Route	Schedule
PD-L1	Durvalumab (MEDI4736)	1500 mg (Arms 1 through 5)<br>1120 mg (Arms 6, 7, and 8)	IV	q4w (Arms 1 through 5)<br>q3w (Arms 6, 7 and 8)
NA	Paclitaxel	90 mg/m^2 (Arms 1 and 5)<br>80 mg/m^2 (Arm 2)[a]	IV	4-week cycles: 3 weeks once weekly (D1, D8, and D15) and 1 week off
AKT	Capivasertib (AZD5363)	400 mg	oral	bid in a 4-week cycle; 3 weeks on (D2, D3, D4, and D5) and 1 week off[b]
CD73	Oleclumab (MEDI9447)	3000 mg	IV	q2w for the first 2 cycles (D1 and D15 of C1 and C2); q4w starting at C3 (D1)
HER2	DS-8201a (trastuzumab deruxtecan)	5.4 mg/kg	IV	q3w
TROP2	Dato-DXd (datopotamab deruxtecan)	6.0 mg/kg	IV	q3w

[a] For patients on Arm 2 who were treated prior to Protocol Version 4, dosing may continue unchanged (ie, if the patient was treated at starting dose 90 mg/m^2 without dose modifications, they may continue at this dose, as clinically indicated).

[b] Capivasertib should not be taken during any week that the paclitaxel infusion is not administered – either due to a scheduled off-drug-week or an unscheduled omission or delay.

AKT protein kinase B; bid Twice daily; C Cycle; CD Cluster of differentiation; D Day; IV Intravenous; NA Not applicable; PD-L1 Programmed cell death ligand 1; q1w Every 1 week; q2w Every 2 weeks; q3w Every 3 weeks; q4w Every 4 weeks.

Duration of treatment

Unless specific treatment discontinuation criteria are met, all patients will continue therapy until clinical progression or radiological progression (defined in Appendix F).

Progression during treatment

Patients who are clinically stable at an initial RECIST 1.1-defined progression of disease (PD) [redacted] to receive [redacted] at the [redacted]. [redacted] with [redacted] who [redacted] [redacted] can receive [redacted], and [redacted] should [redacted] for the duration of [redacted]

Follow-up of patients post discontinuation of study drug

Patients who have discontinued treatment due to toxicity, symptomatic deterioration, or clinical progression during treatment or who have commenced subsequent anticancer therapy will be followed up with tumor assessments until RECIST 1.1-defined PD plus an additional optional follow-up scan or until death (whichever comes first) and followed for survival. These patients are not eligible for re-treatment at any time.

Survival

All patients assigned or randomized to treatment should be followed for survival.

Safety Review Committee

A SRC will review the initial safety profile for each of the novel treatment combination arms in Part 1 after 6 DLT-evaluable patients have completed the first treatment cycle or had a DLT during the first treatment cycle for each treatment arm and will review safety and tolerability data on an ongoing basis. In Arm 8, a review of the initial safety profile will not be undertaken, given that Arm 8 does not have a safety run-in; however, the SRC review safety and tolerability data in Arm 8 on an ongoing basis. Additional details on the use of the SRC during the Part 1 safety run-in period are provided in Section 4.1.4.3.

An SRC will continue to be used throughout Part 1 and in Part 2 of the study, as appropriate.

Sample size

Part 1 of this study enrolled 20 patients in Arm 1 (now closed to enrollment) and will enroll 30 patients in all other treatment arms. Part 2 will enroll a further 27 patients in each treatment arm that meets the [redacted] [redacted] noted below.

The primary objective for Part 1 is safety; 20 patients in the durvalumab + paclitaxel arm and 30 patients per novel treatment combination arm are considered appropriate to characterize the AE profile of each combination. Twenty patients in the durvalumab + paclitaxel arm (Arm 1)

is sufficient to evaluate safety based on data for durvalumab + chemotherapy from other studies. Additional patients (n=30) for novel treatment combination arms is warranted because this study will be the only one to provide data on these novel combinations.

The study is sized to allow the use of a [redacted] for each treatment arm according to the [redacted] Each treatment arm (with the exception of Arm 1) requires 57 response-evaluable patients (30 in Part 1 and 27 in Part 2). If at least 17 out of 30 patients achieve response, then the treatment arm may continue to Part 2; otherwise, further recruitment into this treatment arm will be stopped. If there are at least 38 out of 57 evaluable patients achieving response in a treatment arm, then the data for that cohort will be considered as having an adequate efficacy signal. Additional information is provided in the statistical analysis plan (SAP).

Additional novel therapy arms may be added at the discretion of the Sponsor.

Statistical methods

Efficacy will be assessed in terms of ORR, progression-free survival (PFS), duration of response (DoR), and OS based on patients pooled from Part 1 and Part 2 in each treatment arm. Efficacy data will also be explored in terms of PD-L1 status. Results of ORR statistical analysis will be presented using 95% exact Clopper-Pearson confidence interval (CI).

Kaplan-Meier plots and median DoR, PFS, and OS will be presented. Also, summaries (ie, number of patients [%]) of PFS, death events, will be provided.

The analysis of ORR, DoR, and PFS will be based on site Investigator assessments according to RECIST 1.1 using all scans regardless of whether they were scheduled or not.

<u>Methods for statistical analysis</u>

Four analysis sets will be defined as follows:

- Safety Analysis Set: All patients who received at least 1 dose of study treatment (at least 1 IP [durvalumab, paclitaxel, or novel oncology therapy]).
- Full Analysis Set (FAS): All patients who received at least 1 dose of study treatment in either Part 1 or Part 2. This is the same as the Safety Analysis Set.
- Response Evaluable Analysis Set: All subjects who received study treatment in either Part 1 or Part 2 and who have measurable disease at baseline.
- PK Analysis Set (Part 1 only): All patients who received at least 1 dose of durvalumab or novel oncology therapy per protocol and had at least 1 post-dose evaluable PK data point for durvalumab or the novel oncology therapy.

Safety

The Safety Analysis Set will be used to summarize all safety data according to the treatment received. Data from all cycles of treatment will be combined in the presentation of safety data. The AEs (both in terms of Medical Dictionary for Regulatory Activities [MedDRA] preferred terms and Common Terminology Criteria for Adverse Event [CTCAE] grade) will be listed individually by patient. The number of patients experiencing each AE will be summarized by treatment arm and CTCAE grade. Other safety data will be assessed in terms of clinical chemistry, hematology, and vital signs and summarized using appropriate summary statistics by treatment arm.

Efficacy

Efficacy will be assessed in terms of ORR, PFS, DoR, OS, and PFS6 following assignment of study treatment based on patients pooled from Part 1 and Part 2 in treatment arms 2 to 8 (Arm 1 will not be included in Part 2). A secondary objective in Part 1 and the primary objective of Part 2 is to assess the efficacy of durvalumab + novel oncology therapies with or without paclitaxel, and durvalumab + paclitaxel in terms of confirmed ORR as determined by the Investigator according to RECIST 1.1. The number and percentage of patients who achieve confirmed response (CR or PR) as determined by the Investigator according to RECIST 1.1 in each treatment arm will be summarized, and the 95% Clopper-Pearson CIs will be calculated. To meet additional secondary efficacy objectives, PFS, DoR, OS, and PFS6 will be summarized by treatment arm and Kaplan-Meier plots will be presented for PFS, DoR, and OS. The FAS will be used to summarize efficacy data according to the assigned treatment. Efficacy data will also be explored in terms of PD-L1 status based on the FAS.

Pharmacokinetics and antidrug antibodies

PK and antidrug antibodies (ADAs) will be summarized using the PK Analysis Set and ADA-evaluable set, respectively. The details of the PK and ADA analysis will be provided in the Statistical Analysis Plan.

1.3 Schema

The general study design is summarized in Figure 1.

Figure 1 Study design

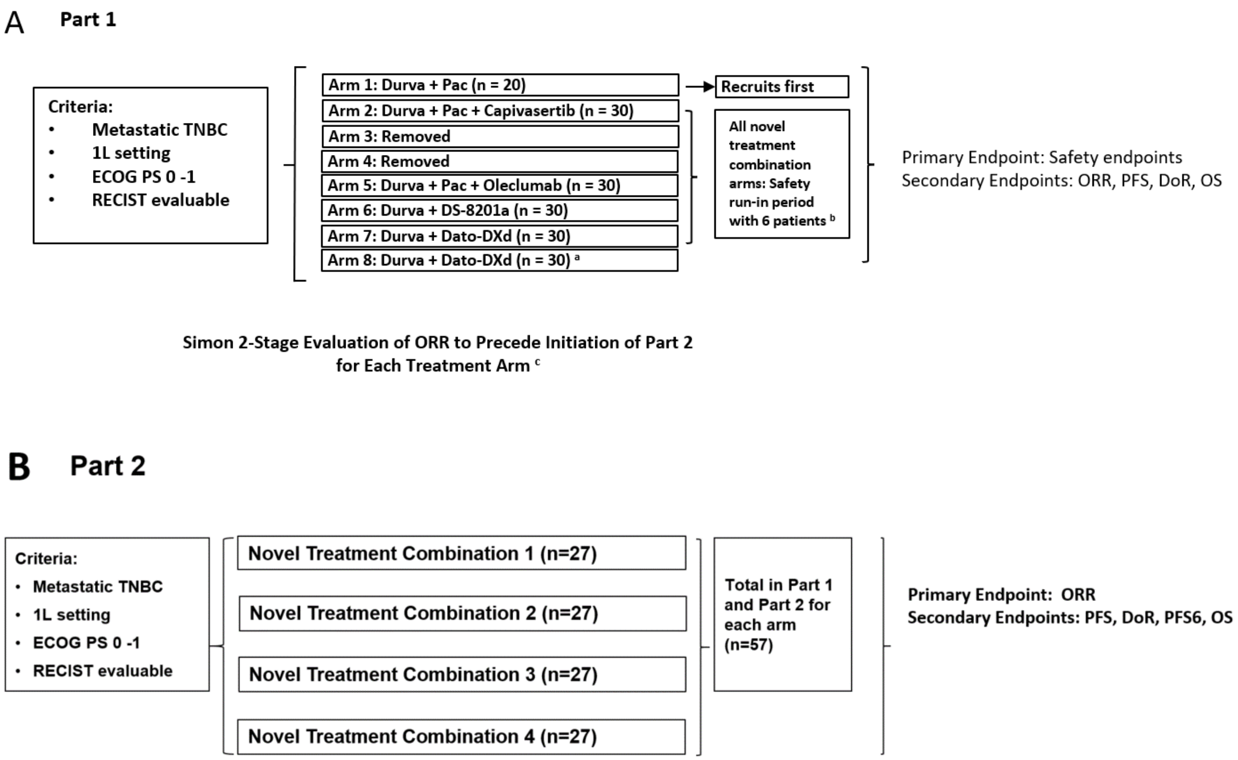

a In patients with PD-L1 positive status.

b In Part 1, the novel treatment combination arms will include a safety run-in period with a planned enrollment of 6 patients. Fewer than 6 patients may be enrolled if 2 patients have DLTs before 6 patients are enrolled; more than 6 patients may be enrolled in order to have 6 DLT-evaluable patients. No safety run-in will occur in Arm 8 given that the durvalumab + Dato-DXd combination was found to be tolerable with no DLTs in Arm 7.

c See Section 9.1 for additional details.

1L First-line; DLT Dose-limiting toxicity; DoR Duration of response; Durva Durvalumab; ECOG Eastern Cooperative Oncology Group; ORR Objective response rate; OS Overall survival; Pac Paclitaxel; Dato-DXd Datopotamab deruxtecan; DS-8201a Trastuzumab deruxtecan; PFS Progression-free survival; PFS6 Progression-free survival 6 months following date of first dose; PS Performance status; RECIST Response Evaluation Criteria in Solid Tumors; TNBC Triple negative breast cancer.

2 INTRODUCTION

2.1 Study rationale

This is a Phase IB/II, 2-stage, open-label, multicenter study to determine the efficacy and safety of durvalumab in combination with novel oncology therapies with or without paclitaxel and durvalumab with paclitaxel for the treatment of first-line metastatic triple negative breast cancer (TNBC). Clinical studies to date have revealed a substantial proportion of patients with TNBC who do not derive benefit from the combination of taxanes and immune checkpoint inhibitors (programmed cell death 1 [PD-1])/programmed cell death ligand 1 [PD-L1]), despite the encouraging responses of these therapies in a limited patient population. Modalities with promise for breast cancer (BC) and therapies with available clinical safety and combination data with durvalumab will be prioritized for study towards developing a novel treatment combination.

2.2 Background

A detailed description of the chemistry, pharmacology, efficacy, and safety of durvalumab and each novel oncology therapy is provided in the respective Investigator's Brochure for each molecule.

2.2.1 Triple negative breast cancer

Globally, BC is the most diagnosed cancer among women (GLOBOCAN 2012), the second most common cancer overall, and the fifth leading cause of cancer deaths. BC is the leading cause of cancer death among European women, responsible for an estimated 91585 deaths in 2012 (GLOBOCAN 2012) and is projected to kill 40610 women in the United States (US) in 2017 (Desantis et al 2017, Noone et al 2017). Despite significant improvements in early detection and treatment, most women who develop metastatic disease will eventually succumb to the disease.

BC subtypes are based on the tumor expression of the estrogen receptor (ER), progesterone receptor (PgR), and human epidermal growth factor receptor 2 (HER2). Hormone receptor-positive tumors (ER+, PgR+) are treated with agents that interrupt hormonal pathways, and HER2+ tumors are treated with agents that down-regulate HER2 activity. However, no targeted therapies currently exist for TNBC, the subtype of BC characterized by a lack of tumor expression of ER, PgR, or HER2.

TNBC accounts for 15% to 20% of newly diagnosed BC (Hudis and Gianni 2011). Among all early-stage (Stages I to III) BC subtypes, TNBC has the highest probability of relapse and portends the poorest OS. Metastatic (Stage IV) TNBC has an aggressive clinical course and shorter PFS and OS than early-stage TNBC. Affected patients are managed by serial administration of single-agent chemotherapeutics, generally with adverse events (AEs) that

impact quality of life and poor clinical outcomes. TNBC patients have a poor prognosis and limited treatment options and thus present an unmet medical need.

The current standard of care (SoC) for early-stage TNBC is a taxane-based regimen, with further addition of an anthracycline in the neoadjuvant setting. The standard clinical practice for early-stage TNBC following completion of definitive local and systemic chemotherapy treatment is observation. Cytotoxic chemotherapy is the main systemic therapy for patients presenting with advanced TNBC. Platinum salts, taxanes, and antibody drug conjugates with topoisomerase I inhibitors have been evaluated in patients with metastatic TNBC, with the following results for monotherapies and combination therapies:

- In Study TBCR009, patients received cisplatin or carboplatin as first-line or second-line therapy. The objective response rate (ORR) was 25.6% overall (32.6% with cisplatin and 18.7% with carboplatin), with a median PFS of 2.9 months and an OS of 11 months (Isakoff et al 2015).
- In the TNT study, patients with metastatic or locally advanced TNBC were treated with either carboplatin or docetaxel in the first-line setting. Response rates of 31.4% for carboplatin and 35.6% for docetaxel were not significantly different. Patients bearing breast cancer susceptibility gene (BRCA) 1/2 germline mutations responded better to carboplatin than to docetaxel, with an ORR of 68.0% versus 33.3%, respectively (Ganpenrieder et al 2017).
- CBCSG006, a randomized Phase III study, explored cisplatin + gemcitabine compared with paclitaxel + gemcitabine as first-line therapy for metastatic TNBC (Hu et al 2015). With 120 patients per group, the ORR and median PFS were 64% and 7.7 months in the cisplatin + gemcitabine group and 49% and 6.5 months in the paclitaxel + gemcitabine group. Of note, the OS results for both combination cohorts were similar (19.37 months for cisplatin + gemcitabine and 18.07 months for paclitaxel + gemcitabine).
- In Study DS8201-A-J101, a Phase I study enrolling patients with HER2 low-expressing breast tumors (HER2 IHC1-2+), patients were treated with DS-8201a (trastuzumab deruxtecan) monotherapy. Overall response rates of 37% (20/54) were reported in extensively pretreated patients, including a subset of TNBC patients (DS-8201 Investigator's Brochure Version 6). Evaluation of patients with HER2 low-expressing breast tumors in this study is ongoing. The response rates of DS-8201a, which delivers a topoisomerase I inhibitor to the tumor, were comparable to overall response rates with docetaxel in earlier lines of therapy (Ganpenrieder et al 2017).
- In the ASCENT study, a randomized Phase III study in patients with previously treated metastatic TNBC, the trophoblast cell surface protein 2 (TROP2) antibody-drug conjugate (ADC), sacituzumab govitecan, was compared with treatment of physician's choice. Of the 529 patients enrolled, median PFS for sacituzumab govitecan was 5.6 months versus 1.7 months for SoC chemotherapy (hazard ratio 0.41; $P<0.0001$), median OS was 12.1 months for sacituzumab govitecan versus 6.7 months for SoC chemotherapy (hazard ratio 0.48; $P<0.0001$) and overall response

rate was 35% versus 5% (Bardia et al 2020).DS1062-A-J101 is an ongoing Phase 1, 2-part, multicenter, nonrandomized, open-label, multiple dose, first-in-human study of Dato-DXd. The TNBC cohort enrolled 24 patients (22 patients on 6.0 mg/kg and 2 patients on 8.0 mg/kg) who were heavily pretreated (21 patients with ≥ 2 prior lines of therapy). A total of 6 patients (25%) discontinued the study due to disease progression. No patients discontinued the study due to an AE. Dose reductions due to AEs occurred in 6 patients (25%) and were mostly due to stomatitis (3 patients [13%]) and mucosal inflammation (2 patients [8%]). Most patients (67%) experienced mild to moderate AEs that were nonhematologic. There were no cases of Grade 3 diarrhoea or neutropenia and no cases of adjudicated drug-related interstitial lung disease (ILD). Antitumor response as measured by ORR from a blinded independent central review (BICR) was 43% and the disease control rate (DCR) was 95% (Bardia 2021).

- Overall, serial monotherapy of chemotherapeutics is the primary SoC, with promising ORR and PFS; however, OS still remains poor.

2.2.1.1 BRCA mutations in TNBC

Germline BRCA1/2 mutation is a known risk factor for breast cancer, with current National Comprehensive Cancer Network (NCCN) guidelines recommending screening for all women diagnosed with TNBC who are younger than 60 years old (NCCN Guidelines). BRCA1 and BRCA2 defective tumors are intrinsically sensitive to poly (ADP-ribose) polymerase inhibitors. The OlympiaD trial evaluated patients with a BRCA1/2 mutation and randomized previously treated patients with breast cancer who received either olaparib or standard of care. The patients who received olaparib had an improved PFS of 7.0 months compared to 4.2 months (HR 0.58) (Robson et al 2017). It is currently unknown whether BRCA1/2 mutations are predictive of benefit with the current proposed treatment regimens.

2.2.2 Immunotherapy in breast cancer

Given the broad success of immune checkpoint inhibitors in providing durable clinical response in multiple cancer types (see Section 2.2.3), the utility of PD-1/PD-L1 blockade in BC is under active investigation. Although checkpoint inhibitors have been successfully approved in melanoma, non-small cell lung cancer (NSCLC), and bladder cancer, to date, none have been approved in BC at the time of writing this protocol. In clinical studies, the ability to modulate the immune system in BC has been demonstrated with antibodies that target PD-1 (pembrolizumab) and PD-L1(durvalumab, atezolizumab, and avelumab).

In JAVELIN, 168 patients (BC subtypes: 42.9% ER+/PgR+, 34.5% TNBC, 15.5% HER2+, and 7.1% unknown) received avelumab monotherapy and had an ORR of 4.8% (Heery et al 2017). Although responses were seen in all BC subtypes, they appeared to be higher in TNBC (8.6%) or in patients with high PD-L1 expression (defined by ≥10% PD-L1[+] immune cells). In 9 PD-L1(+) and 39 PD-L1(-) TNBC patients (48 patients total), the ORRs were 44.4% and 2.6%, respectively. In an atezolizumab study, PD-L1 status was prospectively assessed

(positive criteria: >5% PD-L1-staining immune cells), and ORR was 13% in PD-L1(+) and 5% in PD-L1(-) patients, of 112 evaluable TNBC patients in total; the ORR was much higher (26%) for patients receiving first-line treatment (Emens et al 2015). In KEYNOTE-012 and KEYNOTE-086, patients with TNBC were treated with pembrolizumab, and PD-L1 expression was assessed (positive criteria: >1% of tumor or immune cells stained for antibody 22C3) (Nanda et al 2016). In KEYNOTE-012, an ORR of 18.5% was observed in 27 PD-L1(+) patients. KEYNOTE-086 included both salvage therapy and first-line cohorts for metastatic PD-L1(+) TNBC and reported ORRs of 4.8% and 23.1%, respectively. Although encouraging responses to immunotherapies have been seen in patients with high PD-L1 tumor expression (PD-L1[+] patients), particularly as a first-line treatment, this population represents only a subset of TNBC patients who could thus derive clinical benefit.

Several studies have evaluated the addition of immunotherapy to standard chemotherapy, with the goal of stimulating or "priming" the immune system. As was observed in the immunotherapy-alone studies, there was a trend toward higher response rates (ORR of 46%) in patients treated first line. The IMPassion 130 study evaluated 902 patients with untreated metastatic TNBC. Patients were randomly assigned to atezolizumab or placebo in combination with nab-paclitaxel to assess PFS and OS. The changes in PFS data were significant in both the intention-to-treat (ITT) analysis group (the median PFS was 7.2 months with atezolizumab plus nab-paclitaxel compared to 5.5 months with placebo plus nab-paclitaxel [p=0.002]) and the PD-L1-positive tumor analysis subgroup (the median PFS was 7.5 months with atezolizumab plus nab-paclitaxel compared to 5.0 months with placebo plus nab-paclitaxel [p<0.001]) (Schmid et al 2018b). The IMPassion 131 study, in which paclitaxel is evaluated instead of nab-paclitaxel, is ongoing at the time of this protocol amendment. Pembrolizumab and eribulin combinations were explored in 95 previously treated metastatic TNBC patients of any PD-L1 status; the ORR for patients who had seen 2 or 3 prior lines of therapy or were treatment naïve was 27.3% and 41.2%, respectively, and did not appear to be dependent on PD-L1 status (Tolaney et al 2016). These promising results are being followed up in KEYNOTE-355, an ongoing Phase III study of first-line pembrolizumab with chemotherapy in metastatic TNBC.

2.2.3 Immunotherapies

It is increasingly understood that cancers are recognized by the immune system and that, under some circumstances, the immune system may control or even eliminate tumors (Dunn et al 2004).

PD-L1 is part of a complex system of receptors and ligands that are involved in controlling T cell activation. The PD-1 receptor (cluster of differentiation [CD]279) is expressed on the surface of activated T cells (Keir et al 2008). It has 2 known ligands: PD-L1 (B7-H1; CD274) and programmed cell death ligand-2 (PD-L2; B7-DC; CD273) (Okazaki and Honjo 2007). PD-1 and PD-L1/PD-L2 belong to a family of immune checkpoint proteins that act as

coinhibitory factors, which can halt or limit the development of T cell response. When PD-L1 binds to PD-1, an inhibitory signal is transmitted into the T cell, which reduces cytokine production and suppresses T cell proliferation. Tumor cells exploit this immune checkpoint pathway as a mechanism to evade detection and inhibit immune response.

PD-L1 is constitutively expressed by B-cells, dendritic cells, and macrophages (Qin et al 2016). Importantly, PD-L1 is commonly over-expressed on tumor cells or on non-transformed cells in the tumor microenvironment (Pardoll 2012). PD-L1 expressed on the tumor cells binds to PD-1 receptors on the activated T cells, leading to the inhibition of cytotoxic T cells. These deactivated T cells remain inhibited in the tumor microenvironment. The PD-1/PD-L1 pathway represents an adaptive immune resistance mechanism that is exerted by tumor cells in response to endogenous antitumor activity.

The inhibitory mechanism described above is co-opted by tumors that express PD-L1 as a way of evading immune detection and elimination. The binding of an anti-PD-L1 agent to the PD-L1 receptor inhibits the interaction of PD-L1 with the PD-1 and CD80 receptors expressed on immune cells. This activity overcomes PD-L1-mediated inhibition of antitumor immunity. While functional blockade of PD-L1 results in T cell reactivation, this mechanism of action (MOA) is different from direct agonism of a stimulatory receptor such as CD28.

PD-L1 is expressed in a broad range of cancers. Based on these findings, an anti-PD-L1 antibody could be used therapeutically to enhance antitumor immune responses in patients with cancer. Results of pre-clinical and clinical studies of monoclonal antibodies (mAbs) targeting the PD-L1/PD-1 pathway have shown evidence of clinical activity and a manageable safety profile, supporting the hypothesis that an anti-PD-L1 antibody could be used to therapeutically enhance antitumor immune response in cancer patients (Brahmer et al 2012, Hirano et al 2005, Iwai et al 2002, Okudaira et al 2009, Topalian et al 2012, Zhang et al 2008) with responses that tend to be more pronounced in patients with tumors that express PD-L1 (Powles et al 2014, Rizvi et al 2015, Segal et al 2015). In addition, high mutational burden (eg, in bladder carcinoma; Alexandrov et al 2013) may contribute to the responses seen with immune therapy.

Clinical data has now been added to a wealth of pre-clinical data showing that blockade of negative regulatory signals to T cells such as PD-L1 has promising clinical activity. Nivolumab and pembrolizumab, 2 anti-PD-1 agents, and atezolizumab and durvalumab (see Section 2.2.4), 2 anti-PD-L1 agents, have been granted approvals by agencies for the treatment of a number of malignancies, including metastatic melanoma, squamous and non-squamous cell NSCLC, squamous cell carcinoma of the head and neck and urothelial carcinoma. In addition, there are data from agents in the anti-PD-1/PD-L1 class showing clinical activity in a wide range of tumor types.

2.2.4 Durvalumab

Durvalumab is a human mAb of the immunoglobulin G (IgG) 1 kappa subclass that blocks the interaction of PD-L1 (but not PD-L2) with PD-1 on T cells and CD80 (B7.1) on immune cells. It is being developed by AstraZeneca/MedImmune for use in the treatment of cancer. (MedImmune is a wholly owned subsidiary of AstraZeneca; AstraZeneca/MedImmune will be referred to as AstraZeneca throughout this document.). The proposed MOA for durvalumab is interference in the interaction of PD-L1 with PD-1 and CD80 (B7.1). The blockade of PD-L1/PD-1 and PD-L1/CD80 interactions releases the inhibition of immune responses, including those that may result in tumor elimination. In vitro studies demonstrate that durvalumab antagonizes the inhibitory effect of PD-L1 on primary human T cells, resulting in restored interferon-gamma (IFN-γ)-induced proliferation (Stewart et al 2015). In vivo studies have shown that durvalumab inhibits tumor growth in xenograft models via a T cell-dependent mechanism (Stewart et al 2015). Based on these data, durvalumab is expected to stimulate the patient's antitumor immune response by binding to PD-L1 and shifting the balance toward an antitumor response. Durvalumab has been engineered to reduce antibody-dependent cellular cytotoxicity and complement-dependent cytotoxicity.

To date, durvalumab has been given to an estimated 14260 patients as part of ongoing studies either as monotherapy or in combination with other anticancer agents. Details on the safety profile of durvalumab are summarized in Section 8.3.14. Refer to the current durvalumab Investigator's Brochure for a complete summary of pre-clinical and clinical information, including safety, efficacy, and pharmacokinetics (PK).

2.2.5 Durvalumab in combination with chemotherapy and rationale for novel treatment combinations

There is a strong rationale for adding PD-1/PD-L1 blockade to chemotherapy. In fact, there are several reports describing the prognostic value of immune signature and tumor-infiltrating lymphocytes (TILs) for outcome to chemotherapy, including the following:

- Large retrospective analysis of patient samples demonstrating that patients with increased TILs have a robust correlation with increased response to chemotherapy (Savas et al 2016).
- Paclitaxel monotherapy when given to patients with BC increases the percentage of TILs, promoting a more immunogenic environment (Demaria et al 2001).
- Chemotherapy is thought to synergize with PD-1/PD-L1 blockade by making the tumor "immunogenic" through initiating cell death, promoting phagocytosis of tumor cells, and ultimately leading to reactivation of immune-mediated tumor surveillance.
- RNA sequencing analysis of breast tumors demonstrated a strong link between expression of immune markers and the percentage of TIL embedded in the lesion. BC cases with the highest TIL percentage were generally TNBC. In addition,

large-scale gene expression analysis demonstrated strong associations between immunomodulatory genes and outcome to chemotherapy (Savas et al 2016).

As discussed in Section 2.2.2, a substantial proportion of patients do not derive benefit from the combination of chemotherapy and PD-1/PD-L1 inhibition; therefore, developing a novel treatment combination with other immunotherapies or targeted therapies is of particular interest.

Durvalumab has previously been combined with taxane therapy and was found to be well tolerated in an ongoing study (Study NCT02250326). Seventy-eight patients with recurrent metastatic NSCLC were treated with nab-paclitaxel and durvalumab. The median number of treatment cycles was 7.0 (range: 1 to 14 cycles), with a median treatment duration of 24.4 weeks (range: 1.4 to 41.1 weeks). Treatment-emergent adverse events (TEAEs) of special interest for durvalumab + nab-paclitaxel are summarized in Table 10. Overall, the treatment combination was well tolerated.

Table 10 Durvalumab and nab-paclitaxel TEAEs of special interest

TEAE of special interest, n (%)	Durvalumab + nab-paclitaxel (N = 78)	
	**All Grades**	**Grades 3 and 4**
**Non-hematologic**		
Peripheral sensory neuropathy	19 (24.4)	3 (3.8)
Dyspnea	16 (20.5)	4 (5.1)
**Hematologic**		
Neutropenia	13 (16.7)	5 (6.4)
Febrile neutropenia	0	0
Anemia	22 (28.2)	3 (3.8)

TEAE Treatment-emergent adverse event.

The combination of topoisomerase I inhibition and PD-L1 blockade has been shown to result in an additive antitumor response in pre-clinical models. In the FM3A murine breast tumor model, there was an increase in the proliferation and number of tumor-infiltrating CD8+ T cells in response to combination therapy with the topoisomerase I inhibitor, irinotecan, and the anti-PD-L1 antibody. Irinotecan alone was also found to decrease the number of regulatory T cells in lymph nodes and tumors, augment major histocompatibility complex (MHC) class 1 expression on tumor cells, and also increase PD-L1 expression on tumor cells and tumor-infiltrating immune cells (Iwai et al 2018).

In pre-clinical studies, DS-8201a has been combined with an anti-programmed cell death antibody (PD-1) to determine whether there is synergy with topoisomerase I inhibition and immunotherapy. The combination of DS-8201a and the anti-PD-1 antibody was more effective than either monotherapy in a syngeneic mouse model, using the murine colon cancer cell lines CT26.WT that stably expresses human HER2 (DS-8201a Investigator's Brochure). In addition, the combination of DS-8201a and an anti-PD-1 antibody is currently being tested in breast cancer patients (Study DS-8201-A-U105; NCT03523572 [completed]; DS8201-A-U106; NCT04042701).

Overexpression of TROP2 is associated with more aggressive disease, poorer OS, and worse disease-free survival in patients with solid tumors (Goldenberg et al 2018). In breast cancer, increased TROP2 mRNA is a strong predictor of lymph node involvement, distant metastasis, and poor OS (Lin et al 2013, Zhao et al 2018). Overexpression of TROP2 is observed across all breast cancer subtypes, including TNBC where 80% of patients show TROP2 overexpression (Son et al 2018, Huang et al 2005). In direct assessment of TROP2 activity, overexpression of TROP2 mRNA and protein stimulated cell growth with tumor progression that was abrogated with a TROP2 knockdown (Trerotola et al 2013). Additionally, in evaluation of 702 consecutive breast cancer cases, membrane localization of TROP-2 had an unfavorable prognostic factor for OS (HR 1.63; p = 0.04) (Ambrogi et al 2014).

It is expected that Dato-DXd will demonstrate efficacy in cancers that have high expression of TROP2. In vitro studies indicate that Dato-DXd exhibits TROP2 expression-dependent inhibition of cell growth through induction of DNA damage and apoptosis, and in vivo studies in human cancer-bearing mouse models have shown administration of Dato-DXd results in stronger antitumor activity in TROP-2 high tumors compared to TROP2-low or negative tumors. In the TROP2 positive pancreatic CFPAC-1 xenograft mouse mode, Dato-DXd induced dose dependent tumor growth inhibition and tumor regression (Okajima et al 2018). The biodistribution and antitumor activity of the ADC are expected to depend on the expression level of the target antigen in tumor tissue.

The combination of TROP2 and PD-L1 inhibition has been explored with a bi-specific TROP2/PD-L1 chimeric antigen receptor (CAR)-T cell. In in vitro studies, the bi-specific TROP2/PD-L1 CAR-T cells showed specific cytotoxicity against TROP2- and PD-L1-positive expressing gastric cancer cells, which was higher than that of mono-specific CAR-T cells, and also produced IFN-γ and interleukin (IL)-2 in response to the overexpression of TROP2 and PD-L1 in gastric cancer cells. The bi-specific TROP2/PD-L1 CAR-T cells significantly reduced the tumor growth of a xenograft model bearing human gastric tumors (Zhao et al 2019). These studies together indicate that treatment of TROP2 expressing cells with Dato-DXd may result in DNA damage and apoptosis of target cells, and the combination with durvalumab may lead to additive responses driven by enhanced T cell activation through reduction of regulatory T cells, augmented MHC class-1 antigen presentation, and inhibition

of PD-L1 signaling. In addition to directly targeting TROP-2 expressing cells, the hydrolysable linker of Dato-DXd also enables the topoisomerase I inhibitor, DXd, to be released into the tumor microenvironment. Thus adjacent tumor cells may also be killed by DXd released extracellularly via a bystander effect (Kalinsky et al 2020, Ogitani et al 2016). This has been demonstrated in NSCLC patients where responses to Dato-DXd were seen irrespective of TROP2 expression (Lisberg et al 2020).

The potential for TROP2 ADCs in the treatment of breast cancer has been demonstrated with the April 2020 FDA accelerated approval of sacituzumab govitecan (TRODELVY; IMMU-132; Immunomedics, Inc.) for patients with metastatic TNBC who have received at least 2 prior therapies. This ADC is a conjugate of a humanized TROP2 monoclonal antibody linked with SN-38, the active metabolite of irinotecan, enabling the selective delivery of SN-38 to TROP2 expressing tumors (Bardia et al 2017). Pre-clinical studies have shown sacituzumab govitecan mediated antitumor responses in different tumor types with varying levels of TROP2 expression, including a squamous cell lung carcinoma tumor line having low TROP2 surface expression and a TNBC tumor cell line, HCC1806, with moderate TROP2 levels (Goldenberg et al 2015., Cardillo et al 2015, Cardillo et al 2020). These studies support the hypothesis that high surface TROP2 expression may be predictive of a positive clinical outcome for sacituzumab govitecan. In mice bearing the high TROP2 expressing MDA-MB-468 TNBC tumor model, this ADC could induce significant tumor regression compared to saline or an irinotecan control and mediate the activation of the intrinsic apoptotic pathway (Goldenberg et al 2015.). In IMMU-132-01 (NCT01631552), a Phase 1/2 single arm study of sacituzumab govitecan monotherapy in patients with epithelial cancers, 108 patients with metastatic TNBC, who had received at least 2 prior treatments for metastatic disease, were treated with 10 mg/kg sacituzumab govitecan. The most common adverse events (AEs) were nausea, diarrhea, fatigue, and myelosuppression, and discontinuation rates due to AEs were low (2.8%). The overall response rate was 33.3% (36/108) and the median duration of response (DoR) was 7.7 months (Bardia et al 2019). Among patients with TROP2 assessment (n=48), there was a trend toward higher PFS in patients with moderate to strong TROP2 expression (88%) versus those with weak TROP2 expression or no staining (12%) (Bardia et al 2017). Furthermore, in the ASCENT study, a randomized Phase 3 study of sacituzumab govitecan versus treatment of physician's choice in patients with previously treated metastatic TNBC, sacituzumab govitecan demonstrated significant PFS and OS improvement over SoC chemotherapy. Of the 529 patients enrolled, median PFS for sacituzumab govitecan was 5.6 months versus 1.7 months for SoC chemotherapy (hazard ratio 0.41; $P<0.0001$), median OS was 12.1 months for sacituzumab govitecan versus 6.7 months for SoC chemotherapy (hazard ratio 0.48; $P<0.0001$) and overall response rate was 35% versus 5% (see Figure 2). In the safety population (n=224), key treatment-related grade ≥3 AEs with sacituzumab govitecan versus SoC chemotherapy were neutropenia (51% versus 33%), diarrhea (10.5% versus <1%), anemia (8% versus 5%), and febrile neutropenia (6% versus 2%). No grade >3 neuropathy or ILD, and no treatment-related deaths were reported (Bardia et al 2020).

Sacituzumab govitecan is also being investigated in combination with the anti-PD-1 antibody, pembrolizumab, in patients with PD-L1-negative metastatic TNBC (NCT04468061).

Figure 2 Overall survival and progression free survival in patients treated with sacituzumab govitecan versus treatment of physician's choice in patients with previously treated metastatic triple-negative breast cancer in the ASCENT study (NCT02574455)

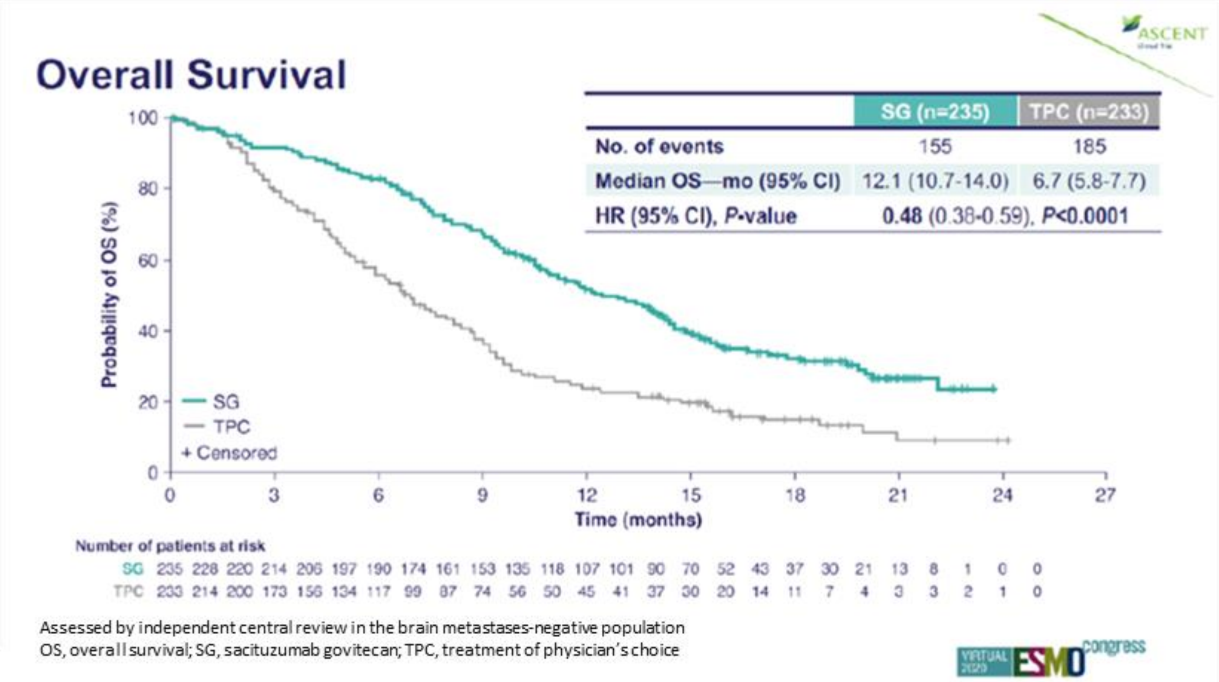

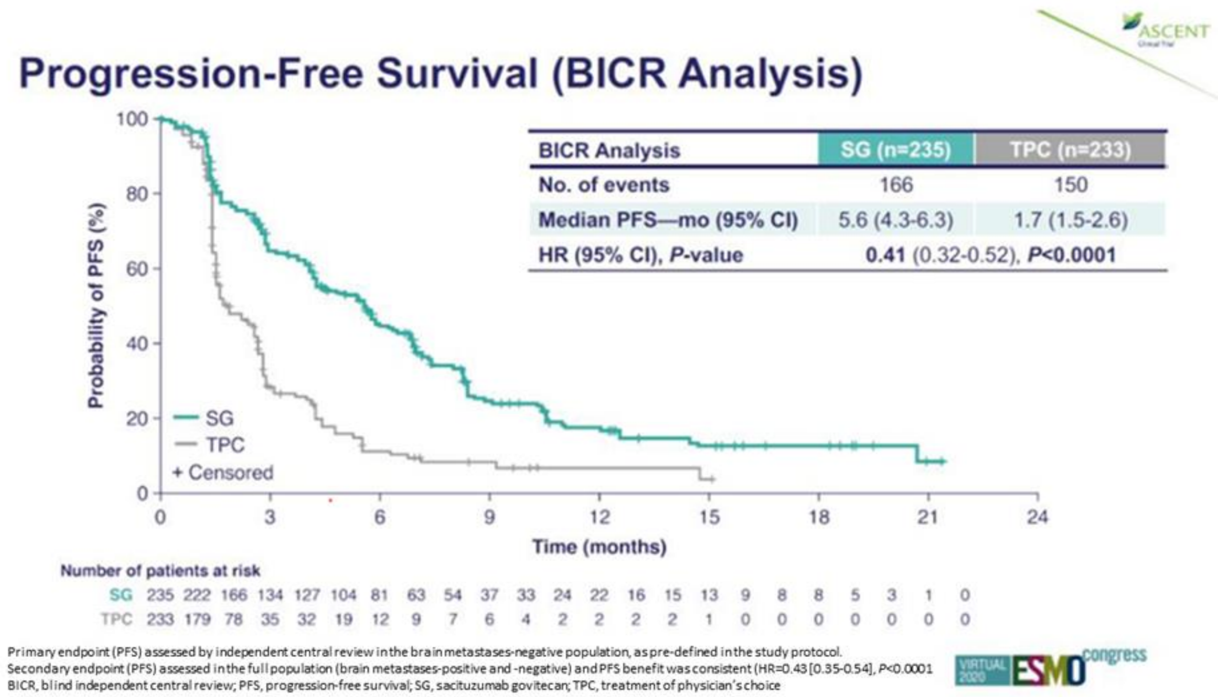

In this Phase IB/II study, novel oncology therapies designed for immune modulation by different mechanisms are being tested in combination with durvalumab (PD-L1 blocker) with and without paclitaxel (chemotherapy). Modalities with promise for BC and therapies with available clinical safety and combination data with durvalumab will be prioritized for study.

Background on the novel oncology therapies is provided in Section 2.2.6 through Section 2.2.9.

2.2.6 Capivasertib (AZD5363)

Capivasertib (previously known as AZD5363) is a potent, selective inhibitor of the kinase activity of the serine/threonine-specific protein kinase AKT (protein kinase B [PKB]) (Davies et al 2012). Capivasertib is being developed as a potential treatment for solid and hematological malignancies.

AKT is a node of multiple signaling pathways promoting tumorigenesis, inhibiting apoptosis, impacting on the cell cycle, and promoting invasion and migration. All 3 AKT isoforms are activated in different tumor types, including breast, prostate, ovarian, pancreatic, and gastric cancers, and this activation is often associated with resistance to established cancer therapies as well as advanced disease and/or poor prognosis (Altomare et al 2005). AKT activation in tumors is largely due to input from other signaling pathways upstream of AKT (eg, mutation of oncogenes such as Ras or Bcr-abl, mutation of receptor tyrosine kinases such as epidermal growth factor receptor, amplification of HER2, loss of phosphatase and tensin homolog [PTEN] function, or mutations of phosphoinositide-3-kinase [PI3K]).

The PI3K/AKT signaling pathway is often activated in TNBC through activating mutations in PIK3CA or AKT1 and/or inactivating alterations in PTEN (Cancer Genome Atlas Network 2012, Curtis et al 2012, Shah et al 2012). Deficient expression of PTEN is a common finding in TNBC and has been associated with a higher degree of AKT pathway activation (LoRusso 2016, Millis et al 2015). The prevalence of TNBC patients with tumors harboring PIK3CA/AKT1/PTEN alterations is reported to be in the range of 23% to 41% (Ciriello et al 2015, Curtis et al 2012, Kim et al 2017, Pereira et al 2016, Wilson et al 2019).

Inhibitors of AKT are anticipated to have efficacy when combined with cytotoxic chemotherapies or when combined with targeted or antihormonal agents. Capivasertib inhibits all 3 AKT isoforms (AKT1, AKT2, and AKT3) and therefore has the potential to provide clinical benefit over a range of therapeutic indications. A significant relationship has been found in pre-clinical studies between the presence of a PI3K, catalytic, alpha polypeptide (PIK3CA) mutation or PTEN mutations and sensitivity to monotherapy with capivasertib in a panel of 182 cancer cell lines (p=0.0059; t-test). Capivasertib inhibits the growth of several human breast cancer xenograft models, including the TNBC xenograft model HCC1187 and the HER2+, PIK3CA mutant xenograft models BT474 and HCC1954. Combination studies in

breast cancer xenograft models, including the TNBC xenograft model HCC1187, demonstrate significantly increased efficacy when capivasertib and docetaxel are combined, compared to treatment with taxane or capivasertib alone. The HCC1187 xenograft model does not carry mutations of PIK3CA, AKT1 or PTEN, confirming that activity in combination studies is not restricted to models with intrinsic, aberrant PI3K pathway activation (Davies et al 2012).

2.2.6.1 Clinical experience of capivasertib

As of the final data cut-off (DCO) date of 04 October 2017, approximately 456 patients have been exposed to capivasertib in AstraZeneca-sponsored studies, including 297 patients treated with capivasertib monotherapy, 92 patients treated with capivasertib in combination dosing with paclitaxel, and 67 patients treated with capivasertib in combination dosing with fulvestrant. Of the 5 studies in the clinical development program, 2 are ongoing (Studies D3610C00001 and D3610C00002), 2 were completed (Studies D3610C00004 and D3610C00007), and 1 (Study D3610C00003) was terminated early following an unscheduled interim analysis indicating that capivasertib was unlikely to generate a positive efficacy signal in this setting.

In addition, 18 Investigator-sponsored studies of capivasertib are planned or ongoing.

Tumor response data available from 2 Phase I studies—D3610C00001 (Parts A and B) (Western population) and D3610C00004 (Japan)—showed that 3 of the 131 patients had Response Evaluation Criteria in Solid Tumors (RECIST) partial responses (PRs): 1 patient whose tumor was positive for PIK3CA mutation and 2 patients with tumors that tested positive for AKT1 mutation. These Phase I studies recruited patients from advanced cancer populations who had been heavily pre-treated and showed resistance to a number of prior therapies. The expansion parts of Study D3610C00001 (Parts C and D) recruited an advanced cancer population selected for patients with proven PIK3CA (Part C) or AKT1 (Part D) mutations. In patients with ER+ BC, 1 patient had a confirmed RECIST response of PR in Part C (PIK3CA mutation). In patients with gynecological cancer, 2 patients with PIK3CA-mutant cancer had a confirmed RECIST response of PR. In patients with AKT1 E17K-mutant tumors (n=52) and a median of 5 lines of prior therapy, the median PFS was 5.5 months (95% CI: 2.9 to 6.9 months) in patients with ER+ BC, 6.6 months (95% CI: 1.5 to 8.3 months) in patients with gynecologic cancer, and 4.2 months (95% CI: 2.1 to 12.8 months) in patients with other solid tumors (Banerji et al 2017).

Of note, while the addition of capivasertib to first-line weekly paclitaxel in ER+HER- advanced BC (Study D3610C00002 [BEECH]) did not improve PFS in the overall or the PIK3CA-mutation positive population (Turner et al 2017), recently reported data on the externally sponsored randomized, double-blind, placebo-controlled, Phase II study PAKT showed that the same combination was highly effective in metastatic TNBC patients as first-line therapy, substantially improving PFS and OS in the overall population. The benefits were

more pronounced in patients with *PIK3CA/AKT1/PTEN*-altered tumors detected by next-generation sequencing (NGS) (Schmid et al 2018a). Capivasertib in combination with paclitaxel was well tolerated overall and had a manageable safety profile, with no marked impact on the tolerability and dose intensity of paclitaxel. In response to the positive results from the PAKT study, a Phase III study (CAPItello-290, NCT03997123) is being conducted with the aim to provide confirmatory evidence of the efficacy and safety of capivasertib in combination with paclitaxel versus placebo + paclitaxel as first-line treatment for patients with locally advanced (not amenable to resection with curative intent) or metastatic TNBC.

Details on the safety and risk profile of capivasertib alone and in combination therapy are provided in Sections 2.3.2.2, 2.3.2.3, and 2.3.2.4. Further details regarding capivasertib are provided in the capivasertib Investigator's Brochure.

2.2.7 Oleclumab

Oleclumab (MEDI9447) is a human mAb of the IgG1 lambda (λ) subtype that selectively binds to and inhibits the ectonucleotidase activity of CD73. The triple mutation, L234F/L235E/P331S (according to the European Union [EU] numbering convention), is encoded in the heavy chain constant region to significantly reduce IgG effector function. Oleclumab inhibits the catalysis of adenosine monophosphate (AMP) to adenosine and organic phosphate by CD73. Extracellular adenosine is believed to mediate the immunosuppressive effects of both MDSCs and regulatory T cells, among others (Antonioli et al 2013). The enzymatic blockade of CD73 caused by binding of oleclumab to CD73 may therefore lead to increased antitumor immunity.

CD73 inhibition has been studied in several in vivo models. In mouse tumor models, targeting cancer cell CD73 using RNA interference inhibited tumor growth by direct inhibition of tumor cell migration and metastasis (Zhi et al 2007) and by freeing antitumor T cells from the suppressive effects of extracellular adenosine, restoring efficacy of adoptive T cell therapy (Jin et al 2010). In a variety of mouse tumor models, CD73 antagonist antibodies may rescue endogenous antitumor responses, especially in tumor types with high levels of expression of CD73, such as TNBC (Stagg et al 2010). Ablation of CD73 in gene-targeted mice resulted in the suppression of growth of colon cancer, lymphoma, mammary tumors, and melanoma (Stagg et al 2011). Furthermore, in murine models of mammary carcinoma and melanoma, dual inhibition of CD73 and the adenosine A2A receptor resulted in reduced tumor growth, increased immune infiltrates, and improvement in OS as compared to either placebo or each treatment as monotherapy (Young et al 2016), suggesting that dual inhibition of the adenosinergic pathway may be an effective therapeutic approach.

In 122 samples from patients with TNBC, high CD73 protein expression was inversely related to patient disease-free survival and OS (Buisseret et al 2017). Notably, CD73 gene expression is significantly associated with a worse prognosis in TNBC patients but not in patients with

luminal or HER2+ BC (Loi et al 2013). It is postulated that in TNBC, CD73 overexpression may confer chemoresistance to doxorubicin, or other anthracycline therapies, by suppressing adaptive antitumor immune responses via activation of A2A adenosine receptors. In pre-clinical models, targeted blockade of CD73 in TNBC reversed the effect and enhanced doxorubicin-mediated antitumor immune responses, significantly prolonging survival (Loi et al 2013). Chemotherapy, including paclitaxel, has also been reported to induce the transcription of PD-L1 and CD73 in TNBC (Samanta et al 2018), creating an environment that is highly susceptible to a paclitaxel, durvalumab, and oleclumab combination.

Details on the potential benefits of oleclumab based on clinical experience are provided in Section 2.3.3.1.

Details on the safety and risk profile of oleclumab alone and in combination therapy are provided in Sections 2.3.3.2 and 2.3.3.3.

2.2.8 DS-8201a (trastuzumab deruxtecan)

DS-8201a (T-DXd) is an ADC that targets HER2. The molecule consists of an antibody component (MAAL-9001), which is a humanized immunoglobulin G1 (IgG1) monoclonal antibody with reference to the amino acid sequence of trastuzumab. The antibody is covalently conjugated via a maleimide tetrapeptide linker to the drug component, an exatecan derivative that is a topoisomerase I inhibitor (DXd). This ADC has a high drug-to-antibody ratio (DAR) (7-8:1) with homogenous conjugation with DXd. DS-8201a is cleaved by lysosomal enzymes and releases DXd in the cytoplasm after it binds to HER2 receptor and gets internalized in tumor cells. DS-8201a is expected to inhibit tumor growth on the basis of the following reasons: it exhibits antibody dependent cellular cytotoxicity similar to those of trastuzumab when it binds to HER2; the DXd that is released from DS-8201a after the internalization induces apoptosis by inhibiting topoisomerase I.

As of 08 June 2019, 1036 patients received at least 1 dose of DS-8201a. In the ongoing DS8201-A-J101 study, the overall efficacy results in patients with HER2-positive breast cancer at 5.4 mg/kg or 6.4 mg/kg demonstrated a confirmed ORR of 52.5%. In patients with HER2-low expressing tumors (HER2 IHC1+ or 2+), the overall response rates of 37% (20/54) were reported in extensively pretreated patients. A subset of the patients with HER2 low tumors in the study were diagnosed with TNBC. The overall efficacy results in patients with other cancers demonstrated a confirmed ORR of 29.5%.

Details on the safety and risk profile of DS-8201a alone and in combination therapy are provided in Sections 2.3.3.2 and 2.3.3.3. Further details regarding DS-8201a are provided in the DS-8201a Investigator’s Brochure.

2.2.9 Dato-DXd (datopotamab deruxtecan; DS-1062a)

Dato-DXd is an ADC that targets tumor-associated calcium signal transducer 2, TROP2, a transmembrane protein that is highly expressed in various epithelial tumors including esophageal squamous cell carcinoma, breast cancer, and non-small cell lung cancer. The molecule consists of a recombinant humanized anti-TROP2 IgG1 monoclonal antibody, MAAP-9001a, which is covalently conjugated to a drug linker, MAAA-1162a, via thioether bonds. Upon binding to TROP2, Dato-DXd is internalized, and after enzymatic processing, the topoisomerase I inhibitor, DXd, is released, leading to inhibition of tumor growth and apoptosis of target cells. In non-clinical toxicology studies, lung toxicity, corneal toxicity, skin toxicity, intestinal toxicity, lymphatic/hematopoietic system toxicity, reproductive and accessory organ toxicities, hepatic toxicity, renal toxicity, and joint cartilage toxicity were found in association with the administration of Dato-DXd. As with any therapeutic antibodies, there is a possibility of infusion-related reactions and immune responses causing allergic or anaphylactic reactions to Dato-DXd.

DS1062-A-J101 is an ongoing Phase I first-in-human study evaluating escalating doses of Dato-DXd in advanced solid tumors, including TNBC. Clinical data are available evaluating escalating doses of DS 1062a (0.27 to 10.0 mg/kg) in unselected patients with advanced NSCLC or TNBC who have relapsed or are refractory to standard of care therapy (Lisberg et al 2020).

The Phase 1 study had a dose escalation phase and a dose expansion phase. A total of 208 patients were enrolled as of the Investigator's Brochure DCO, 04 September 2020. The median duration of treatment was 2.76 months (range: 0.7 to 20.0 months). The patients treated include pathologically documented unresectable advanced NSCLC or TNBC who have been refractory to or relapsed on standard treatment. In the escalation phase, dose-limiting toxicities occurred in 3 subjects: 2 subjects at 10 mg/kg (1 with Grade 3 stomatitis and 1 with Grade 3 mucosal inflammation) and 1 subject at 6.0 mg/kg (Grade 3 maculo-papular rash). The maximum tolerated dose was determined at 8.0 mg/kg during the expansion phase.

As of the DCO, 189 patients were evaluable for a response assessment (defined as having at least 1 dose of Dato-DXd and at least 1 pre- and post-baseline tumor assessment or discontinued treatment). The ORR by BICR was 15.4% (6 PRs in 39 patients) in the 6.0 mg/kg dose group and 23.8% (19 PRs in 80 patients) in the 8.0 mg/kg dose group. The DCR was 66.7% (6 PRs and 18 stable disease [SDs] in 39 patients) in the 6.0 mg/kg dose group and 80.0% (19 PRs and 43 SDs in 80 patients) in the 8.0 mg/kg dose group. Duration of response was not sufficiently mature as of the DCO, but preliminary results supported the durability of the response with median DoR by BICR of 5.5 months at 8.0 mg/kg and not yet reached at 4.0 and 6.0 mg/kg.

As of the DCO, TEAEs were reported in 95.7% of 208 patients; TEAEs ≥ Grade 3 were reported in 42.8 % and serious TEAEs occurred in 34.1% of patients. The most frequent (≥ 20% of patients) TEAEs regardless of causality were nausea (46.6%), stomatitis (38.9%), fatigue (34.1%), alopecia (33.7%), decreased appetite (24.5%), vomiting (23.1%), and constipation (20.7%). A serious adverse event (SAE) of infusion-related reaction (IRR) occurred in 3 patients (1 on 4 mg/kg, 1 on 6 mg/kg, and 1 on 8 mg/kg) and 1 patient with a history of allergy had an SAE of anaphylaxis (4 mg/kg). Apart from 2 patients with a Grade 3 IRR (1 on 4 mg/kg and 1 on 6 mg/kg), IRR cases were mild or moderate, and manageable by close observation and dose modification.

Twenty patients (9.6%) had a TEAE associated with discontinuation of study drug. Twenty-eight patients (13.5%) and 35 patients (16.8%) had TEAEs that were associated with dose interruption or dose reduction, respectively. There were 14 patients (6.7%) with an ILD event that was independently adjudicated as treatment related, including 12 patients (14.6%) on 8.0 mg/kg, 1 patient (2.2%) on 6.0 mg/kg, and 1 patient (2.0%) on 4.0 mg/kg. National Cancer Institute - Common Terminology Criteria for Adverse Events (NCI CTCAE) grades for these 14 events were as follows: three Grade 1, six Grade 2, two Grade 3, and three Grade 5 events. The patients with an adjudicated drug related Grade 5 ILD were on 8.0 mg/kg. Interstitial lung disease/pneumonitis is considered an important identified risk for Dato-DXd. Established treatment guidelines are in place to manage toxicities associated with Dato-DXd.

At the DCO, 46 patients were dosed at 6.0 mg/kg, the planned dose level for Dato-DXd to be combined with durvalumab in BEGONIA. The median duration of treatment was 2.07 months (range: 0.7 to 19.7 months) at the 6.0 mg/kg dose. At the 6.0 mg/kg dose, dose interruptions, reductions, and discontinuations due to AEs occurred in 9 (19.6%), 4 (8.7%), and 4 (8.7%) patients, respectively. Of the 39 response-evaluable patients at the 6.0 mg/kg dose level (defined as having at least 1 dose of Dato-DXd and at least 1 post-baseline tumor assessment or discontinued treatment), 6 (15.4%) patients had a confirmed PR, and 2 of these patients discontinued due to PD. Forty-two (91.3%) patients at the 6.0 mg/kg dose experienced a TEAE, with 7 (15.2%) patients having treatment-related TEAEs ≥ Grade 3 and with 4 (8.7%) patients having serious treatment-related TEAEs. There was 1 (2.2%) patient with an ILD event that was independently adjudicated as treatment related. This ILD event was Grade 2 according to NCI CTCAE. There was 1 patient with a Grade 5 TEAE in this dose cohort, which was not related to study drug. Overall, Dato-DXd was noted to have an acceptable and manageable safety profile.

An oral presentation at the European Society for Medical Oncology, Breast Cancer Congress 2021 presented results on the TNBC cohort in Dato-Dxd DS1062-A-J101 ongoing Phase 1 study through 08 January 2021. The Dato-DXd TNBC Cohort enrolled 24 patients (22 patients on 6.0 mg/kg and 2 patients on 8.0 mg/kg) who were heavily pretreated (21 patients with ≥ 2 prior lines of therapy). A total of 6 patients (25%) discontinued the study due to

disease progression. No patients discontinued the study due to an AE. Dose reductions due to AEs occurred in 6 patients (25%) and were mostly due to stomatitis (3 patients [13%] and mucosal inflammation (2 patients [8%]). Most patients (67%) experienced mild to moderate AEs that were nonhematologic. There were no cases of Grade 3 diarrhoea or neutropenia and no cases of adjudicated drug-related ILD. Antitumor response as measured by ORR from a BICR was 43% and the DCR was 95% (Bardia 2021).

Details on the safety and risk profile of Dato-DXd alone and in combination therapy are provided in Sections 2.3.5.2 and 2.3.5.3. Further details regarding Dato-DXd are provided in the Dato-DXd Investigator's Brochure.

2.3 Benefit/risk assessment

More detailed information about the known and expected benefits and risks and reasonably expected AEs of durvalumab and each novel oncology therapy may be found in the respective Investigator's Brochure for each molecule.

2.3.1 Durvalumab

2.3.1.1 Potential benefits of durvalumab

The majority of the safety and efficacy data currently available for durvalumab (MEDI4736) are based on the first-in-human, single-agent study (Study 1108) in patients with advanced solid tumors. Overall, as of 07 May 2015, 456 of 694 patients treated with durvalumab 10 mg/kg every 2 weeks (q2w) were evaluable for response. Evaluable patients were defined as having ≥24 weeks of follow-up, measurable disease at baseline, and ≥1 follow-up scan or discontinued because of PDs or death without any follow-up scan. In PD-L1 unselected patients, the ORR in TNBC was <10%, with a DCR of 4.2% at 24 weeks. A Phase I/II study of durvalumab in combination with olaparib (a poly-adenosine-diphosphate-ribose polymerase inhibitor) is ongoing in patients with advanced solid tumors, including TNBC.

2.3.1.2 Potential risks of durvalumab

2.3.1.2.1 OVERALL IMMUNOTHERAPY RISKS

Monoclonal antibodies directed against immune checkpoint proteins, such as PD-L1 as well as those directed against PD-1 or cytotoxic T-lymphocyte–associated antigen 4 (CTLA-4), aim to boost endogenous immune responses directed against tumor cells. By stimulating the immune system, however, there is the potential for adverse effects on normal tissues.

Most adverse drug reactions seen with the immune checkpoint inhibitor class of agents are thought to be due to the effects of inflammatory cells on specific tissues. These risks are generally events with a potential inflammatory or immune-mediated mechanism and may require more frequent monitoring and/or unique interventions such as immunosuppressants and/or endocrine therapy. These immune-mediated effects can occur in nearly any organ

system and are most commonly seen as gastrointestinal AEs such as colitis and diarrhea, pneumonitis/ILD, hepatic AEs such as liver enzyme elevations, skin events such as rash and dermatitis, and endocrinopathies including hypothyroidism and hyperthyroidism.

2.3.1.2.2 DURVALUMAB RISKS

Risks with durvalumab include, but are not limited to, diarrhea/colitis, abdominal pain, cough/productive cough, pneumonitis/ILD, endocrinopathies (ie, events of hypophysitis/hypopituitarism, adrenal insufficiency, hyper- and hypo-thyroidism, thyroiditis, type I diabetes mellitus [which may present with diabetic ketoacidosis] and diabetes insipidus), dysphonia, hepatitis/increases in transaminases (ALT and AST increase), nephritis/increases in creatinine, dysuria, rash/dermatitis, pruritus, night sweats, IRRs, hypersensitivity reactions and anaphylaxis, pancreatitis, serious infections, pyrexia, oedema peripheral, upper respiratory tract infections, pneumonia, oral candidiasis, dental and oral soft tissue infections, influenza, myalgia, cytokine release syndrome, and other rare or less frequent inflammatory events including neuromuscular toxicities (eg, Guillain-Barré syndrome, myasthenia gravis, myocarditis, myositis/polymyositis, immune thrombocytopenia, and encephalitis), pericarditis, sarcoidosis, uveitis, and other events involving the eye (eg, keratitis and optic neuritis), skin (eg, scleroderma, vitiligo and pemphigoid), rheumatological events (polymyalgia rheumatic and immune-mediated arthritis), vasculitis, non-infectious meningitis, non-infectious encephalitis, and psoriasis.

For information on all identified and potential risks with durvalumab, please always refer to the current version of the durvalumab Investigator’s Brochure.

In monotherapy clinical studies, AEs at an incidence of $\geq$20% include events such as fatigue, cough, decreased appetite, dyspnea and nausea. Approximately 10% of patients discontinued the drug due to an AE. Please see the current version of the Investigator’s Brochure for a detailed summary of the monotherapy data including AEs, SAEs, and CTC Grade 3 to 5 events reported across the durvalumab program.

The majority of treatment-related AEs were manageable with dose delays, symptomatic treatment, and, in the case of events suspected to have an immune basis, the use of established treatment guidelines for immune-mediated toxicity (see Section 8.4.6.1).

A detailed summary of durvalumab monotherapy AE data can be found in the current version of the durvalumab Investigator’s Brochure.

2.3.2 Capivasertib (AZD5363)

2.3.2.1 Potential benefits of capivasertib

The PI3K/AKT/PTEN pathway is frequently deregulated in TNBC and drives tumor growth and cell survival. PI3K pathway activation is frequently associated with resistance to

chemotherapy, and multiple lines of pre-clinical investigation have demonstrated that the addition of AKT- or PI3K-pathway inhibitors can significantly increase the activity of chemotherapy and overcome resistance.

Capivasertib is a potent inhibitor of AKT1, AKT2, and AKT3 (half-maximal inhibitory concentration [IC_{50}] <10 nM). Non-clinical in vitro and in vivo assays have demonstrated the inhibition of phosphorylation of the AKT substrates GSK3β and PRAS40 and of tumor growth in xenograft models. Furthermore, pre-clinical studies have demonstrated strong synergistic antitumor activity when capivasertib and taxane chemotherapy are used in combination.

In the randomized, placebo-controlled Phase II study (PAKT) of 140 patients with newly diagnosed metastatic TNBC, the median OS was 19.1 months in patients treated with capivasertib in combination with paclitaxel compared to 12.6 months for placebo with paclitaxel ([HR: 0.61; 95% CI: 0.37 to 0.99; 1-sided p=0.02; 2-sided p=0.04). The median PFS was 5.9 months for active treatment compared to 4.2 months for placebo (HR: 0.74; 95% CI: 0.50 to 1.08), with a greater effect observed in a biomarker-selected population (patients with tumors harboring *PIK3CA/AKT1/PTEN* mutations by NGS).

2.3.2.2 Potential risks identified from non-clinical studies of capivasertib

This section is based on non-clinical toxicology and safety pharmacology studies with capivasertib. The toxicological profile of capivasertib has been evaluated in rats and dogs in studies of up to 9 months in duration. The key risks identified from non-clinical assessments to date are considered to be effects on glucose homeostasis and insulin signaling, cardiac function, and renal function. Other tissues affected in non-clinical studies have included the reproductive organs, liver, hypothalamic-pituitary axis, and hematopoietic system. In addition, genotoxicity and phototoxicity have been assessed.

Glucose homeostasis and insulin signaling: Marked increases in blood insulin and glucose levels were noted following dosing in the dog and rat studies. Levels of insulin and glucose had generally returned to normal 24 hours post-dose. AKT is known to be involved in the regulation of glucose metabolism (Chang et al 2004); therefore, these changes are likely to be related to the primary pharmacology of capivasertib.

CCI [redacted]

[redacted]

Renal function: Polyuria, glucosuria, proteinuria, increased water consumption, and decreased tubular epithelial cell size have been observed non-clinically, but there was no associated histopathology in the kidney. These effects are consistent with the pharmacological activity of AKT in terms of a role in proximal tubular glucose and phosphate transport (Kempe et al 2010).

Reproductive toxicity: Studies in rats and dogs showed degenerative changes in the testes and epididymides in males, as well as decreased prostate weights. Findings in the female reproductive organs (uterus and ovaries) were noted in the rat 1-month toxicity study but were limited to a reversible reduction in organ weight, with no histopathological findings. Capivasertib had an adverse effect on embryonic survival as well as early postnatal growth when administered to pregnant and lactating rats. Exposure to capivasertib was confirmed in suckling pups, which may indicate the potential for excretion of capivasertib in milk.

Additional information is provided in the current version of the Investigator's Brochure for capivasertib.

2.3.2.3 Potential risks of capivasertib from early clinical studies

As of the DCO date of 04 October 2017, 5 clinical studies sponsored by AstraZeneca using capivasertib have been initiated. A total of 297 patients have been treated with capivasertib monotherapy, 92 patients with capivasertib in combination dosing with paclitaxel, and 67 patients with capivasertib in combination dosing with fulvestrant (see also Section 2.2.6.1).

Expected adverse events of special interest (AESIs) for capivasertib are diarrhea, hyperglycemia, rash, stomatitis, infection/lower respiratory tract infection, and [redacted] Most cases can be managed by treatment with other drugs (eg, topical steroids, anti-diarrheal drugs) and/or by dose adjustment of capivasertib, per AE toxicity management guidelines provided in Section 8.4.6.3. Additionally, the AE of fatigue is commonly reported for capivasertib, affecting >30% of patients in the pooled monotherapy intermittent group, irrespective of causality.

Further information on specific AESIs can be found in Section 6.5.2 of the capivasertib Investigator's Brochure.

2.3.2.4 Potential risks of capivasertib in combination with paclitaxel

Capivasertib was administered in combination with weekly paclitaxel in 2 Phase II, randomized, placebo-controlled studies. The AE profile was consistent with the individual AE profiles of each drug, with no additional observations in relation to safety and tolerability.

In the externally sponsored Phase II study, PAKT, of capivasertib in combination with paclitaxel compared to placebo with paclitaxel in women with metastatic TNBC, the most common Grade ≥3 AEs, were diarrhea (13.2% of capivasertib-treated patients versus 1.4% of placebo-treated patients), infection (4.4% versus 1.4%), and neutropenia (2.9% versus 2.9%). In the AstraZeneca-sponsored Phase II study, BEECH, of capivasertib in combination with paclitaxel compared to placebo with paclitaxel in women with metastatic ER+HER- BC, the most common Grade ≥3 AEs, irrespective of causality, were diarrhea (24.1% of capivasertib-treated patients versus 1.8% of placebo-treated patients), hyperglycemia (13% versus 0), maculo-papular rash (9.3% versus 0), peripheral neuropathy (5.6% versus 1.8), alanine aminotransferase (ALT) increased (3.7% versus 3.6%), and stomatitis (3.7% versus 0). In both PAKT and BEECH studies, capivasertib in combination with paclitaxel was well tolerated overall and had a manageable safety profile, with no marked impact on the tolerability and dose intensity of paclitaxel.

The study design aims to minimize potential risks of the novel treatment combination. Careful blood glucose monitoring to evaluate for hyperglycemia will be done throughout the study. Toxicity management guidelines are shown in Section 8.4.6.3, and further guidance for Investigators is available in the capivasertib Investigator’s Brochure. Secondary prophylaxis and loperamide can be used to reduce the risk of capivasertib-induced diarrhea. There is overlap between the toxicity profiles for capivasertib and durvalumab (see Section 2.3.1.2), notably with diarrhea and rash, which are to be managed with loperamide for prevention and the toxicity management guidelines as noted in Section 8.4.6.3.

2.3.3 Oleclumab (MEDI9447)

A detailed summary of oleclumab (MEDI9447) monotherapy and combination data can be found in the current version of the oleclumab Investigator’s Brochure.

2.3.3.1 Potential benefits of oleclumab

Oleclumab (MEDI9447) is a human IgG1λ mAb that selectively binds to and inhibits the ectonucleotidase activity of CD73. Oleclumab inhibits the catalysis of AMP to adenosine and organic phosphate by CD73. The enzymatic blockade of CD73 caused by the binding of oleclumab to CD73 may lead to increased antitumor immunity.

Preliminary data from Study D6070C00001 demonstrate that treatment with oleclumab is well tolerated and leads to a reduction in CD73 expression both in the tumor and on circulating immune cells. In addition, oleclumab has been demonstrated to inhibit CD73 enzymatic activity in patient biopsies after treatment.

The overall ORR (confirmed and unconfirmed) in the oleclumab 40 mg/kg + durvalumab 10 mg/kg dose expansion phase was 7.1% (95% CI: 1.5%, 19.5%). The ORR (confirmed and unconfirmed) in the CRC and pancreatic cancer cohorts was 4.8% (95% CI: 0.1%, 23.8%) and

10.0% (95% CI: 1.2%, 31.7%), respectively. The overall DCR in the dose expansion phase was 16.7% (95% CI: 7.0%, 31.4%). The DCR in the CRC and pancreatic cancer cohorts was 14.3% (95% CI: 3.0%, 36.3%) and 20.0% (95% CI: 5.7%, 43.7%), respectively.

2.3.3.2 Potential risks of oleclumab

In Study D6070C00001, 42 patients enrolled to receive oleclumab monotherapy, and 74 patients received oleclumab + durvalumab combination therapy. In the monotherapy cohort, 39 (92.9%) patients experienced ≥1 TEAE, and 23 (54.8%) patients experienced a treatment-related TEAE. The most frequently reported TEAEs (in ≥20% of the total patients) were fatigue (38.1%) and anemia, abdominal pain, and dyspnea (21.4% each). There were no clinically meaningful trends in the incidence of TEAEs across dose groups. The most frequently reported treatment-related TEAEs (≥5% of the total patients) were fatigue (16.7%) and nausea and anemia (9.5% each). In the monotherapy group, 15 (35.7%) patients experienced treatment-emergent serious adverse events (TESAEs). The only SAE reported by more than 1 patient was ascites, reported by 3 (7.1%) patients. None of the SAEs were treatment related. In oleclumab + durvalumab cohort, 33 (44.6%) patients experienced at least 1 TESAE, and 4 (5.4%) patients experienced treatment-related SAEs. The most frequently reported TESAEs (>2 patients) were abdominal pain and ascites (4.1% each). The treatment-related TESAEs of anemia, thrombocytopenia, and abdominal pain were reported by 1 patient (1.4%) each. The treatment-related SAE of hepatitis was reported by 2 (2.7%) patients.

Three (7.1%) patients discontinued study treatment due to AE in the oleclumab monotherapy dose-escalation phase. Two patients in the oleclumab 10 mg/kg treatment group experienced SAEs (a Grade 3 event of ascites and a Grade 4 event of metastases to central nervous system [CNS]) that led to discontinuation, and 1 patient in the oleclumab 20 mg/kg group experienced Grade 4 pulmonary embolism that led to discontinuation. None of these events were treatment related.

2.3.3.3 Potential risks of durvalumab + oleclumab

Among the 74 patients treated with oleclumab + durvalumab combination therapy, 68 (91.9%) experienced at least 1 TEAE. Thirty-five (47.3%) patients experienced at least 1 treatment-related TEAE. The most frequently reported TEAEs (in ≥15% of the total patients) were fatigue (25.7%) and abdominal pain, constipation, and vomiting (16.2% each). There were no clinically meaningful patterns in the incidence of AEs across all combination therapy dose groups. The most frequently reported treatment-related TEAEs (in ≥5% of the total patients) were fatigue (13.5%); aspartate aminotransferase (AST) increased, diarrhea, and pyrexia (8.1% each); alkaline phosphatase (ALP) increased (6.8%); and ALT increased, decreased appetite, and vomiting (5.4% each). Eight (10.8%) patients experienced TEAEs leading to discontinuation of treatment with oleclumab and durvalumab combination therapy. Three (4.1%) patients experienced treatment-related TEAEs that led to treatment

discontinuation (1 patient experienced treatment-related nausea and vomiting, 1 patient experienced treatment-related AST increased and blood bilirubin increased, and 1 patient experienced treatment-related hepatitis).

2.3.4 DS-8201a (trastuzumab deruxtecan)

2.3.4.1 Potential benefits of DS-8201a

DS-8201a is under development for the treatment of HER2-expressing cancers and HER2-mutant tumors. Based on the preliminary clinical observations in the Phase I study (Study DS8201-A-J101), DS-8201a demonstrates antitumor activity in HER2-expressing cancers, including HER2-positive and HER2-low breast cancer (see Section 2.2.8).

2.3.4.2 Potential risks of DS-8201a

As of 08 June 2021, 3462 patients have been treated with at least one dose of DS-8201a either alone or in combination. Based on data from clinical trials, toxicities considered to be associated with administration of DS-8201a include the important identified risks of ILD/pneumonitis and neutropenia (including febrile neutropenia). Other identified risks for DS-8201a are IRRs, hematological toxicity (thrombocytopenia, anemia, leukopenia, and lymphopenia), pulmonary/respiratory AEs (cough, dyspnea, and upper respiratory tract infection, epistaxis), gastrointestinal AEs (abdominal pain, constipation, decreased appetite, diarrhea, dyspepsia, nausea, stomatitis, and vomiting), hepatic adverse events (hepatic function abnormality, ALT increased, AST increased, ALP increased, and blood bilirubin increased), skin adverse events (alopecia, rash, and pruritis), pneumonia, hypokalemia, dizziness, fatigue, headache, peripheral edema, pyrexia, dry eye, and dehydration.

Based on the available non-clinical data, review of the cumulative literature, and reported toxicities for the same class of agents, the important potential risks for DS-8201a are left ventricular ejection fraction (LVEF) decrease and embryo-fetal toxicity. Keratitis is considered a potential risk for DS-8201a.

ILD/pneumonitis and LVEF decrease are considered to be AESIs.

HER2-targeted agents

Several agents that target HER2 and prevent its activation or heterodimerization have been developed and marketed for the treatment of HER2-positive cancers. These include the monoclonal antibodies trastuzumab (Herceptin®) and pertuzumab (Perjeta®), the antibody-drug conjugate T-DM1 (Kadcyla®), and HER1- and 2-associated tyrosine kinase inhibitor, lapatinib (Tykerb®), and neratinib (Nerlynx®). The safety profile of these HER2-targeted agents has been well described. The main safety risks identified in patients receiving HER2-targeted products are described below; these could potentially be expected to occur in patients receiving DS-8201a.

- **Cardiotoxicity:** Patients treated with trastuzumab are at increased risk for developing congestive heart failure (New York Heart Association [NYHA] class II to IV) or asymptomatic cardiac dysfunction, including LVEF decrease. Cardiac dysfunction, mainly asymptomatic LVEF decrease, has also been observed with pertuzumab in combination with trastuzumab. Similarly, cardiac dysfunction has been observed in patients receiving T-DM1, at a lower incidence than in patients receiving trastuzumab. Most cases have been asymptomatic decreases in LVEF. Cardiac dysfunction with lapatinib has occurred mainly in patients receiving the combination of trastuzumab and lapatinib and has consisted of predominantly asymptomatic LVEF decrease.
- **Pulmonary toxicity:** Cases of pulmonary toxicity, including ILD and pneumonitis, have been observed in patients receiving trastuzumab, T-DM1, and lapatinib. Occasionally, these cases have been severe in nature and have resulted in fatal outcomes. Risk factors associated with ILD/pneumonitis include prior or concomitant therapy with other antineoplastic therapies known to be associated with it such as taxanes, gemcitabine, vinorelbine, and radiation therapy.
- **Hypersensitivity/infusion-related reactions:** The administration of therapeutic proteins is associated with a risk of hypersensitivity and/or infusion reactions. Hypersensitivity/IRRs have been reported with trastuzumab, pertuzumab and T-DM1. These can range from mild reactions to severe anaphylactic shock with fatal outcome, as has been the case for trastuzumab.
- **Hepatic toxicity:** Cases of hepatic toxicity have occurred with T-DM1, lapatinib, and trastuzumab. In patients receiving T-DM1, hepatic toxicity has manifested mainly as transient asymptomatic liver transaminase elevations, although serious cases of drug-induced liver failure and nodular regenerative hyperplasia have also been reported. Lapatinib has also been associated with serious cases of drug-induced liver injury.
- **Hematological toxicity:** Hematological toxicity has been observed with all HER2-targeted therapies. Neutropenia, febrile neutropenia, leukopenia, and anemia have occurred commonly with trastuzumab, pertuzumab, and T-DM1. Thrombocytopenia, including Grade 3 and 4, is a common occurrence in T-DM1-treated patients. Although rare, serious hemorrhagic events have been reported in the setting of thrombocytopenia. Lower rates of thrombocytopenia have also occurred with trastuzumab and pertuzumab when used, in combination with chemotherapy.

Topoisomerase I inhibitors

DXd is a derivative of exatecan (DX-8951f), a topoisomerase I inhibitor. Other products of the same class include irinotecan and topotecan. Exatecan is a campothecin derivative, which has previously been developed by the former Daiichi Pharmaceuticals Co., Ltd. as an anticancer therapy.

- The main risks associated with the use of topoisomerase I inhibitors include hematological toxicities and gastrointestinal toxicities. Hematological toxicities, manifesting as neutropenia, febrile neutropenia, anemia, thrombocytopenia, and

pancytopenia, are commonly observed. An increased risk of infections, including neutropenic colitis and neutropenic sepsis, has been reported with these agents.

- Diarrhea and delayed onset diarrhea, which can be severe and lead to dehydration, have been associated with topoisomerase I inhibitors. Other significant risks include ILD/pneumonitis, liver impairment, immune system disorders, and alopecia. Acute cholinergic syndrome, manifesting as diarrhea and other cholinergic symptoms, has been reported with irinotecan.
- The safety profile of exatecan is broadly similar to the safety profile of other topoisomerase I inhibitors, with hematological toxicities and gastrointestinal toxicities being the most significant groups of events.

Given the data available on the efficacy and safety of DS-8201a, the overall benefit/risk is positive.

2.3.4.3 Potential risks of durvalumab + DS-8201a

At this time, there are limited safety data regarding the combination of durvalumab and DS-8201a. Given the differing mechanisms of action of durvalumab and DS-8201a, the potential for potentiation of toxicities is thought to be limited. Some known overlapping toxicities, for example, CCI [redacted], may on theoretical grounds be potentiated in the combination arm and particular attention should be paid to these.

The study design aims to minimize potential risks of the novel treatment combination. Careful monitoring of pulmonary function to evaluate for ILD/pneumonitis will be done throughout the study. Monitoring toxicity management guidelines are shown in Section 8.4.6.5 and Appendix L, with further guidance for Investigators available in the DS-8201 Investigator's Brochure.

Monoclonal antibodies are not metabolized through classical hepatic enzyme pathways; therefore, no PK interaction is anticipated within this study.

2.3.5 Dato-DXd (datopotamab deruxtecan; DS-1062a)

2.3.5.1 Potential benefits of Dato-DXd

Data from the ongoing DS1062-A-J101 Phase I study show efficacy across dose groups with tumor responses observed at starting doses of 4.0-, 6.0-, and 8.0-mg/kg and an acceptable and manageable toxicity profile across doses of Dato-DXd. As of the DCO date of 04 September 2020, Dato-DXd has demonstrated response rates across these 3 dose cohorts with confirmed and durable responses in a study population that includes a significant proportion of subjects who were heavily pretreated.

The proof of concept of targeting TROP2 has been delivered by the TROP2 ADC (sacituzumab govitecan), which demonstrates a favorable benefit/risk assessment in patients with metastatic TNBC that have received 2 or more lines of therapy (Section 2.2.9).

2.3.5.2 Potential risks of Dato-DXd

Based on the cumulative review of the safety data, the following are the AESIs to be monitored in Dato-DXd studies. ILD/pneumonitis and keratitis are considered important identified risks associated with administration of Dato-DXd. Other identified risks for Dato-DXd are fatigue (including asthenia, lethargy, and malaise), anemia, dry mouth, stomatitis/oral mucositis (including oropharyngeal pain, mouth ulcerations, odynophagia, oral pain, glossitis, pharyngeal inflammation, and aphthous ulcer), diarrhoea, constipation, nausea, decreased appetite, alopecia, vomiting, dry eye, lacrimation increased, conjunctivitis (including conjunctival disorder, conjunctival hyperaemia, and conjunctival irritation), photophobia, blepharitis, meibomian gland dysfunction, vision blurred, and visual impairment/visual acuity reduced, IRR, rash (including rash maculopapular, rash pruritic, and erythematous rash), skin hyperpigmentation (including pigmentation disorder and skin discolouration), pruritus, dry skin/xerosis, and madarosis. Embryo-fetal toxicity is considered an important potential risk. Mucosal inflammation other than oral mucositis/stomatitis is considered a potential risk for Dato-DXd. Dato-DXd has not been studied in participants with renal or hepatic impairment.

The identified/potential risks have been generally manageable through dose modification and routine clinical practice. As with any therapeutic antibodies, there is a possibility of IRRs and immune responses causing allergic or anaphylactic reactions of Dato-DXd.

In the Dato-DXd clinical program, inclusion/exclusion criteria and monitoring/management guidelines are currently built into the study protocols to mitigate all identified and potential risks of Dato-DXd, including the important identified risk of ILD/pneumonitis.

Potential ILD cases are monitored closely for signs/symptoms of ILD and reviewed [redacted] established for the DS-8201a and Dato-DXd program. The study protocols include detailed dose modification and supportive care guidelines for the proactive management of ILD. In the ongoing DS1062-A-J101 Phase I study, the majority of the drug-induced ILD occurred at 8.0 mg/kg.

Ongoing review of emerging Phase 1 study data has allowed a closer evaluation of benefit: risk by dose. Based on the most recent review of safety and efficacy data, Dato-DXd at the 6.0 mg/kg dose has a more favorable benefit: risk profile compared with that of the 8.0 mg/kg and 4.0 mg/kg doses, respectively. Subject to continual review of emerging data, current evaluation of benefit: risk supports the selection of 6.0 mg/kg as the optimal dose for further development of Dato-DXd in patients with advanced/unresectable or metastatic TNBC.

2.3.5.3 Potential risks of durvalumab + Dato-DXd

At this time, there are no reported safety data regarding the combination of durvalumab and Dato-DXd. A Phase IB/II study in advanced or metastatic NSCLC (U104, NCT04612751) evaluating durvalumab with Dato-DXd is currently ongoing and will evaluate safety and

tolerability of this combination. Given the differing mechanisms of action of durvalumab and Dato-DXd, the potential for potentiation of toxicities is thought to be limited. Some known overlapping toxicities, [redacted], may on theoretical grounds be potentiated in the combination arm and particular attention should be paid to these.

The study design aims to minimize potential risks of the novel treatment combination. Careful monitoring of pulmonary function to evaluate for ILD will be done throughout the study. Monitoring toxicity management guidelines are shown in Section 8.4.6.6 and the Annex document to this CSP with further guidance for Investigators available in the Dato-DXd Investigator's Brochure. Where there is potential for overlapping toxicities between agents, consideration should be given to using the guidance from the agent with the most conservative TMGs.

Monoclonal antibodies are not metabolized through classical hepatic enzyme pathways; therefore, no PK interaction is anticipated within this study.

Given the data available on the efficacy and safety of Dato-DXd, the anticipated overall benefit/risk is positive.

2.3.6 Overall benefit/risk

As described in Section 2.2.1, the prognosis of metastatic TNBC is poor. There is a significant unmet medical need for improving treatment outcome and providing more treatment options to patients with metastatic TNBC.

The study design aims to minimize potential risks in several ways. First, via the study design and 2-stage recruitment process, the same comparator arm with durvalumab + paclitaxel is used to evaluate safety and preliminary efficacy for novel treatment combination regimens with or without paclitaxel, in a small number of patients, minimizing patient exposure. Second, the protocol includes safety monitoring in excess of SoC monitoring, with the intent of protecting patients involved in the study. Furthermore, there is specific guidance for Investigators for each therapy to support optimal management of those risks deemed to be most likely or serious. The monitoring and management of potential risks are discussed in Section 8.4.6. For more information on the potential benefits of each therapy and an assessment of the potential and known risks, refer to the respective Investigator's Brochure.

Potential benefits of each combination in patients with TNBC are unknown at this time; however, non-clinical and clinical data to date have shown acceptable safety profile and antitumor activity for the agents proposed in this study.

Thus, the benefit/risk assessment for this Phase IB/II study is acceptable.

3 OBJECTIVES AND ENDPOINTS

Any exploratory analysis within the study related to tumor size may be reported with the primary analysis Clinical Study Report (CSR); all other exploratory analyses may be reported separately. Table 11 outlines the objectives of the study.

Table 11 Study objectives

**Objectives and endpoints - Part 1**	
**Primary objective:**	**Endpoint/variable:**
To assess the safety and tolerability profile of durvalumab + novel oncology therapies with or without paclitaxel and durvalumab + paclitaxel	AEs, exposure, physical examinations, laboratory findings, [redacted] and vital signs
**Secondary objectives:**	**Endpoint/variable:**
To assess the efficacy of durvalumab + novel oncology therapies with or without paclitaxel and durvalumab + paclitaxel in terms of ORR, PFS, DoR, and OS	Endpoints based on Investigator assessment of RECIST 1.1:<br>• ORR: The percentage of evaluable patients with a confirmed Investigator-assessed visit response of CR or PR<br>• PFS: Time from date of first dose until the date of objective radiological disease progression using RECIST 1.1 or death (by any cause in the absence of progression)<br>• DoR: Time from date of first detection of objective response (which is subsequently confirmed) until the date of objective radiological disease progression<br>• OS: Time from date of first dose until the date of death by any cause
To assess the PK of durvalumab and novel oncology therapies (ie, oleclumab, DS-8201a, and Dato-DXd) in all treatment arms	Serum concentration of durvalumab and serum or plasma concentration of novel oncology therapies
To investigate the immunogenicity of durvalumab and applicable novel oncology therapies (ie, oleclumab, DS-8201a, and Dato-DXd) in all applicable treatment arms	Presence of ADAs for durvalumab and applicable novel oncology therapies
**Exploratory objectives:**	**Endpoint/variable:**
To collect blood and tissue samples to evaluate molecular/biological responses and/or identify candidate markers that may correlate with the likelihood of clinical benefit	Blood [redacted], including but not limited to the following, and their association with treatment benefit:<br>• TILs detected by H&E<br>• Protein expression detected by IHC<br>• [redacted]<br>• mRNA expression
To further assess the efficacy of durvalumab + novel oncology therapies with or without paclitaxel and durvalumab + paclitaxel in terms of change in tumor size	Change in tumor size

**Objectives and endpoints - Part 2[a]**	
**Primary objective:**	**Endpoint/variable:**
To assess the efficacy of durvalumab + novel oncology therapies with or without paclitaxel in terms of ORR	Endpoint based on Investigator assessment of RECIST 1.1:<br>• ORR: The percentage of evaluable patients with an Investigator-assessed visit response of CR or PR. Part 1 and Part 2 data will be pooled for efficacy analysis.
**Secondary objectives:**	**Endpoint/variable:**
To further assess the efficacy of durvalumab + novel oncology therapies with or without paclitaxel in terms of PFS, DoR, OS, and PFS6[b]	Endpoints based on Investigator assessment of RECIST 1.1:<br>• PFS: Time from date of first dose until the date of objective radiological disease progression using RECIST 1.1 or death (by any cause in the absence of progression)<br>• DoR: Time from date of first detection of objective response until the date of objective radiological disease progression according to RECIST 1.1<br>• PFS6: PFS at 6 months following date of first dose<br>• OS: Time from date of first dose until the date of death by any cause
To assess the safety and tolerability profile of durvalumab + novel oncology therapies with or without paclitaxel	AEs, exposure, physical examinations, laboratory findings, [redacted] and vital signs
**Exploratory objective:**	**Endpoint/variable:**
To collect blood and tissue samples to evaluate molecular/biological responses and/or identify candidate markers that may correlate with the likelihood of clinical benefit	Blood [redacted], including but not limited to the following, and their association with treatment benefit:<br>• TILs detected by H&E<br>• Protein expression detected by IHC<br>• [redacted]<br>• mRNA expression

a All mentioned endpoints for Part 2 will be analyzed in the data set of Part 1 plus Part 2 patients in each cohort and additionally may be analyzed for each part separately.

b Progression-free survival at 6 months following date of first dose (PFS6) is equivalent to the proportion of patients alive and progression free at 6 months following date of first dose (APF6).

ADA Anti-drug antibody; AE Adverse event; CR Complete response; [redacted]; Dato-DXd Datopotamab deruxtecan; DoR Duration of response; H&E Hematoxylin and eosin stain; IHC Immunohistochemistry; mRNA Messenger ribonucleic acid; ORR Objective response rate; OS Overall survival; PFS Progression-free survival; PK Pharmacokinetics; PR Partial response; RECIST 1.1 Response Evaluation Criteria in Solid Tumors version 1.1; TIL Tumor-infiltrating lymphocyte.

4 STUDY DESIGN

4.1 Overall design

This is a Phase IB/II, 2-stage, open-label, multicenter study to determine the efficacy and safety of durvalumab + novel oncology therapies (ie, capivasertib, oleclumab, DS-8201a [trastuzumab deruxtecan], and Dato-DXd [datopotamab deruxtecan]) with or without paclitaxel and durvalumab + paclitaxel alone as first-line treatment in patients with metastatic (Stage IV) TNBC. The efficacy and safety of durvalumab + Dato-DXd in patients with PD-L1 positive TNBC will also be evaluated in Arm 8 in order to further assess safety and efficacy in patients with PD-L1 positive tumors. The study is designed to concurrently evaluate potential novel treatment combinations with clinical promise using a 2-stage approach. The study will use a CCI [redacted] test to evaluate which cohorts may proceed to expansion.

For an overview of the study design, refer to Section 1.3 (see Figure 1). Details on treatments given during the study are also presented (see Section 6.1). In Part 1 (Phase IB; safety, initial efficacy, and PK/ADA), at least 20 patients will be enrolled into the durvalumab + paclitaxel arm and at least 30 patients in each of the novel treatment combination arms; additional patients may be enrolled in order to have at least 20 or 30 evaluable patients per arm, as appropriate. The safety of durvalumab + paclitaxel has been previously defined (see Section 2.2.5). For novel treatment combinations, the novel oncology therapies will be dosed in combination with durvalumab with or without paclitaxel using a rolling 6-patient design to evaluate for toxicity, where doses for novel oncology therapies will be based on RP2Ds defined in previous combination studies (where available). A total of at least 20 patients will be enrolled in the durvalumab + paclitaxel arm and at least 30 patients in the novel treatment combination arms during Part 1.

The enrollment criteria and screening phase procedures are the same for both study parts. After obtaining informed consent, core biopsy tumor samples from the patient will be confirmed for TNBC status by a local laboratory (details are provided in Section 5.1). Patients who fulfill all of the inclusion criteria and none of the exclusion criteria will be included in the study. In Arm 8, a pre-screening phase will also be conducted to identify patients with a PD-L1 positive status in patients whose PD-L1 expression status has not been determined prior to entry in the study.

In both study parts, patients will receive treatment until any discontinuation criteria are met (see Section 7.1), including clinical progression or radiological progression (refer to Appendix F) and Investigator determination that the patient is no longer benefiting from treatment with IP.

In both study parts, the imaging schedule will be as follows: response evaluation will be performed using computed tomography (CT) or magnetic resonance imaging (MRI) at

screening (as baseline), with follow-up imaging every 8 weeks (q8w) ±1 week (Arms 1 through 5 [ie, arms with 4-week cycles]) and every 6 weeks (q6w) ±1 week (Arms 6, 7, and 8 [ie, arm with 3-week cycles]) for 48 weeks and then every 12 weeks (q12w) ±1 week until RECIST-1.1-defined radiological progression or until the patient has been taken off study (end of study, death, or patient withdrawal of consent). The timing of follow-up imaging will be relative to the date of treatment assignment.

All patients must provide a formalin-fixed and paraffin-embedded (FFPE) tumor sample (archived [≤3 months old] or per routine clinical care but not collected specifically for this study) for determination of [redacted] expression by immunohistochemistry (IHC); however, the results of this assessment will not be used for inclusion/exclusion or stratification purposes. Tumor tissue can be from either the primary tumor or metastatic biopsy. Collected tissue samples will not be limited to PD-L1 testing [redacted] For enrollment onto Arm 6 (durvalumab + DS-8201a), patients must provide pathologically documented advanced/unresectable or metastatic breast cancer with HER2 low expression (as defined in Section 5.1). Patients in Arm 8 must have a PD-L1 positive status, as determined by a pre-existing test or by local testing during pre-screening. Additional details on tumor sample collection and assessments are provided in Section 8.8.

For details on what is included in the efficacy and safety endpoints, see Section 3.

4.1.1 Study conduct mitigation during study disruptions due to cases of civil crisis, natural disaster, or public health crisis

The guidance given below supersedes instructions provided elsewhere in this protocol and should be implemented only during cases of civil crisis, natural disaster, or public health crisis (eg, during quarantines and resulting site closures, regional travel restrictions, and considerations if site personnel or study patients become infected with SARS-CoV-2 or a similar pandemic infection) that would prevent the conduct of study-related activities at study sites, thereby compromising the study site staff or the patient’s ability to conduct the study. The Investigator or designee should contact the study Sponsor to discuss whether the mitigation plans below should be implemented.

To ensure continuity of the clinical study during a civil crisis, natural disaster, or public health crisis, changes may be implemented to ensure the safety of study patients, maintain compliance with Good Clinical Practice, and minimize risks to study integrity.

Where allowable by local health authorities, ethics committees, healthcare provider guidelines, (eg, hospital policies) or local government, these changes may include the following options:

- Obtaining consent/reconsent for the mitigation procedures
- Telemedicine visit: Remote contact with the patients using telecommunications technology, including phone calls, virtual or video visits, and mobile health devices.

For further details on study conduct during a civil crisis, natural disaster, or public health crisis, refer to Appendix P.

4.1.2 Part 1 study design overview

The primary objective of Part 1 will be safety. The safety and tolerability of each treatment arm will be assessed. Additionally, an efficacy analysis for futility (based on ORR) will be assessed using a [redacted] design to determine enrollment for Part 2. Part 1 of the study will enroll the first 20 patients to the durvalumab + paclitaxel arm (Arm 1) and monitor for toxicity; additional patients may be enrolled in order to have 20 evaluable patients. Completion of recruitment to the durvalumab + paclitaxel arm will trigger randomized patient assignment (see Section 6.2.1.1) to each novel treatment combination arm that is open for enrollment. For enrollment onto Arm 6 (durvalumab + DS-8201a), patients must provide pathologically-confirmed documentation of advanced/unresectable or metastatic breast cancer with HER2 low expression (as defined in Section 5.1). Patients with no HER2 tumor expression will be eligible for assignment to any open treatment arm other than Arm 6. For enrollment into Arm 8, patients must provide pathologically-confirmed documentation of advanced/unresectable or metastatic TNBC with PD-L1 positive status, as determined by a pre-existing test or by local testing during pre-screening.

Each subsequent cohort, apart from Arm 8, will undergo a run-in period, in which 6 patients are planned to be enrolled to initially inform the safety profile and determine the RP2D; more or fewer patients may be enrolled depending on how many patients are evaluable for dose-limiting toxicities (DLTs) and how many patients have DLTs. Additional details on the safety run-in period are provided in Section 4.1.4. Briefly, a Safety Review Committee (SRC) will evaluate the safety, tolerability, and available PK of each novel treatment combination arm in Part 1 and may confirm the RP2D or recommend dose modification based on the first 6 DLT-evaluable patients who have completed the first treatment cycle or had a DLT during the first treatment cycle for each treatment arm. A combination dose will be considered not tolerable if 2 or more of up to 6 DLT-evaluable patients experience a DLT at the combination dose level.

The novel treatment combination arms meeting the safety evaluation criteria (ie, no more than 1 of 6 DLT-evaluable patients experience DLTs during the first treatment cycle at a given dose level) will be expanded to at least 30 patients (enrollment sufficient to have 30 evaluable patients).

Efficacy will be evaluated as a secondary objective. Assessments based on confirmed ORR (as determined by the Investigator according to RECIST 1.1) will be made when each cohort has completed enrollment and all patients have either had the opportunity to complete at least 2 on-treatment response evaluations or have discontinued treatment. Each cohort is independent, and any signal observed in 1 arm will not have an impact on other arms.

4.1.3 Part 2 study design overview

Part 2 is stage 2 of the [redacted] design. The assessment ORR on the first 30 subjects recruited in Part 1 (Section 9.1) is applied, and if it is decided to continue recruitment, an additional 27 subjects will be recruited at stage 2, for a total of 57 subjects.

The primary objective of Part 2 will be efficacy, with a primary endpoint of ORR. Efficacy of the novel treatment combination arms will be assessed.

4.1.4 Part 1 safety run-in period and combination dose finding

4.1.4.1 Overview

Each novel treatment combination arm will undergo a run-in period, in which 6 patients are planned to be enrolled to initially inform the safety profile and determine the RP2D. Safety run-in will not occur in Arm 8 as the treatment combination was already evaluated in Arm 7 and found to be tolerable with no DLTs reported. Fewer than 6 patients may be enrolled if 2 patients have DLTs before 6 patients are enrolled; more than 6 patients may be enrolled in order to have 6 DLT-evaluable patients. If patients in the safety run-in phase tolerate a given novel treatment combination, the corresponding novel treatment combination arm will directly enter into the expansion phase and enroll a total of at least 30 patients (enrollment sufficient to have at least 30 evaluable patients per treatment arm).

An SRC will review the emerging data from the study and will continue to monitor safety data on an ongoing basis. The SRC will evaluate the safety, tolerability, and available PK of each combination to either confirm the RP2D or to recommend dose modification (Table 18).

Six DLT-evaluable patients are required to determine the combination recommended dose for further clinical evaluation. Patients participating in the safety-run in period who are not evaluable for DLTs may be replaced. In the event that recruitment is paused for Arms 2 through 7 concurrently while they undergo their DLT review period, patients in screening will have the opportunity to be assigned to Arm 1 to avoid delay in receiving treatment.

A combination dose will be considered not tolerable if [redacted] 6 DLT-evaluable patients experience a DLT at the combination dose level. No further patients will be enrolled at that dose level; however, any patient who did not experience a DLT will continue at the initial assigned dose level until the criteria for dose reduction or treatment discontinuation are met. In the event that the initial dose level is not tolerated, a new cohort with up to 6 new

DLT-evaluable patients will be opened at a lower dose level (see Table 18); this process may be repeated until the lowest dose level is reached. In the event that the lowest dose is not tolerated, no additional patients will be enrolled to evaluate that novel treatment combination.

If no more than [redacted] DLT-evaluable patients experience DLTs [redacted], the SRC will determine an RP2D for the novel treatment combination arm. Once the RP2D is identified, additional patients will be enrolled so that a total of at least 30 patients are enrolled at the RP2D. Additional patients may be enrolled to have 30 evaluable patients.

Once a given patient is assigned a dose level, that patient will not be switched to another dose level during the safety run-in period, unless the dose is reduced for toxicity (see dose reduction and toxicity management guidelines in Sections 6.5.1 and 8.4.6, respectively) in that individual (ie, a patient may not participate in more than 1 cohort).

For Arm 2 (durvalumab + paclitaxel + capivasertib), a new group of 6 DLT-evaluable patients will be reviewed following the adjustments specified in protocol amendment 4, in particular the decrease in paclitaxel dose (see Section 4.3.2). If this regimen is tolerated, additional patients will be enrolled for a total of at least 30 patients across Arm 2.

No new dose level cohorts will be opened once the RP2D is determined. A total of 30 patients will be treated with the RP2D of the novel treatment combination arm, including the 6 DLT-evaluable patients who were enrolled in the safety run-in phase. Patients who are enrolled after the DLT period will be considered evaluable for safety and efficacy if they have received at least 1 dose of any IP in the novel treatment combination arms. As such, patients who received at least 1 dose of any IP during the initial RP2D determination, but who were not DLT evaluable and did not count toward the 6 required patients for RP2D evaluation, will be counted as part of the 30 patient safety and efficacy cohort (ie, as part of the at least 30 evaluable patients).

4.1.4.2 Definitions

4.1.4.2.1 DLT-EVALUABLE PATIENT

For making decisions on the dose, an evaluable patient for the assessment of DLTs in the safety run-in period ("DLT-evaluable" patient) is defined as a patient who has received the full prescribed dose of durvalumab and who has received:

- Arms 2 through 5: [redacted] of the prescribed number of doses of paclitaxel and of the novel oncology therapy during Cycle 1 (Weeks 0 through 3), and either has completed the first 28-day cycle or has a DLT during the first 28-day cycle.
- Arms 6 and 7: must have received the prescribed dose of DS-8201a or Dato-DXd therapy, respectively, during Cycle 1 (Weeks 0 through 2), and either has completed the first 21-day cycle or has a DLT during the first 21-day cycle.

If a patient experiences a DLT that causes them to receive less than the prescribed number of doses stated above in their first cycle, then the patient will still be considered DLT-evaluable. Patients who are not DLT-evaluable by this definition will be replaced.

4.1.4.2.2 DOSE-LIMITING TOXICITY

A DLT will be defined as the occurrence of a [redacted] included on the list below that is at least possibly related to durvalumab and/or the novel oncology therapy. AEs will be graded according to the NCI CTCAE version 4.03. Any clinically insignificant [redacted] that resolves within [redacted] with appropriate medical management will not be considered a DLT.

- [redacted]
- Any [redacted], excluding the following:
  - [redacted] of any duration, unless associated with [redacted]
  - [redacted] or [redacted] lasting less than [redacted] days
  - [redacted] lasting less than [redacted] days
  - [redacted] lasting less than [redacted] days
- [redacted] × upper limit of normal (ULN) with concurrent increase in [redacted] × ULN without evidence of [redacted]
- Any [redacted] toxicity, excluding the following:
  - [redacted] mmol/L) for less than 1 week
  - [redacted] that is considered clinically insignificant by the Investigator and resolves within [redacted] hours with appropriate medical management
  - [redacted] lasting less than [redacted] hours
  - [redacted] lasting less than [redacted] hours
  - [redacted] lasting less than [redacted] days
  - [redacted], unless [redacted] is present
  - [redacted] (first occurrence and in the absence of [redacted] that does not resolve within [redacted] with appropriate clinical management)

For patients treated in Arm 6, the DLT criteria also includes the following:

- [redacted] or symptomatic with a [redacted]

For patients treated in Arm 7 the DLT criteria also includes the following:

- [redacted]
- [redacted]
- [redacted] if accompanied by [redacted]
- In subjects without [redacted] metastases, [redacted] × ULN lasting [redacted] days
- In subjects with [redacted] metastases, [redacted] × ULN lasting [redacted] days, if the baseline level was [redacted] × ULN
- In subjects with [redacted] metastases, [redacted] × ULN lasting [redacted] days, if the baseline level was [redacted] × ULN

4.1.4.3 Safety Review Committee

The SRC will consist of the following:

- The Sponsor Study Clinical Lead or delegate
- The Principal Investigator or delegate sub-investigator from every active site with patients to be discussed during the call

In addition, other physicians from the following may be invited:

- Global Safety Physician or delegate

The Clinical Pharmacology Scientist, Study Statistician, Patient Safety Scientist, Associate Director Study Management, and other delegates may also be invited as appropriate. The SRC Charter for this study will define the exact membership and who should be present for decisions to be made.

Further internal or external experts may be consulted by the SRC, as necessary. The Global Safety Physician or delegate should always be present at the SRC if there are safety issues for discussion.

4.2 Scientific rationale for study design

4.2.1 Rationale for efficacy study endpoints

The primary aim of Part 1 of this study is to determine the safety and tolerability, with a key secondary endpoint of efficacy, of durvalumab + novel oncology therapies with or without paclitaxel and durvalumab + paclitaxel alone as first-line treatment in patients with metastatic TNBC. This will be accomplished by identifying the RP2D for all combinations for which a confirmed safe dose is established. The occurrence of DLTs will be used to establish the RP2D; thus, the standard safety endpoints, such as AEs, SAEs, clinically meaningful changes from baseline in laboratory parameters, and vital signs, will be included in the evaluation. In

addition, PK parameters may be used in determining the RP2D along with safety and efficacy. Changes in paclitaxel dosing, including dose delays, reductions, and discontinuations, will be considered in the evaluation of safety of the durvalumab + paclitaxel and other novel treatment combinations.

Efficacy is not a primary objective of Part 1. Secondary and exploratory efficacy assessments in Part 1 will be based on Investigator assessment according to RECIST 1.1 guidelines. The endpoints for assessment of antitumor activity are those routinely included in oncology studies and will include ORR, PFS, and DoR (based on RECIST 1.1), as well as OS. ORR will be the key secondary endpoint used in Part 1, as it will afford the earliest understanding of which combinations have potential for a robust antitumor response. Additional recruitment of subjects into Part 2 will be considered based on the ORR assessments specified in Section 9.1. Safety of the combination will be continually evaluated throughout enrollment of the trial and will be factored in to determine whether a combination moves into Part 2 of this study.

The primary aim of Part 2 of this study is to determine the efficacy of durvalumab + novel oncology therapies in terms of Investigator-assessed ORR in TNBC patients.

Parts 1 and 2 combined are sized to detect evidence of improved efficacy. Conventionally, ORR, PFS, and OS are used as validated measures of clinical benefit. ORR does not incorporate survival measures as in OS and PFS; however, it has historically been widely used in early phase trials. Simon describes Phase II trials intended to “determine whether [a therapy] has sufficient biologic activity… to warrant more extensive development,” and these are precisely the aims of this study. OS is generally regarded as the most reliable cancer endpoint and is preferred for studies that can be conducted to adequately assess survival. PFS may serve as a surrogate endpoint for OS when differences between treatment arms are of sufficient magnitude and clinical importance (FDA Guidance 2011, Pazdur 2008). ORR improvement in study patients may result in PFS benefits. A PFS secondary endpoint affords an earlier understanding of treatment effect than OS. Furthermore, PFS, unlike OS, will not be confounded by a crossover effect of subsequent treatments, hence the need to include PFS and OS as secondary endpoints.

The secondary endpoints in Parts 1 and 2, duration of response (DoR), PFS at 6 months following date of first dose (PFS6), and OS will be examined to further evaluate the antitumor effect of durvalumab + novel oncology therapies with or without paclitaxel versus durvalumab + paclitaxel combination therapy.

In Part 2, antitumor activity will be based on RECIST 1.1 guidelines.

Changes in paclitaxel dosing, including dose delays, reductions, and discontinuations, will be considered in the evaluation of safety of the durvalumab + paclitaxel arm and durvalumab + novel oncology therapies with or without paclitaxel.

4.2.2 Rationale for other study endpoints

Biological samples will be used to explore potential biomarkers in tumor, plasma, and/or serum, which may influence or may be associated with the progression of cancer (and associated clinical characteristics) and/or response.

In Part 1 only, blood samples will be taken to allow for research into the PK of durvalumab and the novel oncology therapies and immunogenicity of durvalumab and the novel oncology therapies. Arms 7 and 8 (Dato-DXd) will also include plasma PK samples for Part 2 of the study.

4.2.3 Rationale for treatment duration

Treatment in this study will continue until any discontinuation criteria are met (see Section 7.1), including clinical progression or radiological progression (refer to Appendix F) and Investigator determination that the patient is no longer benefiting from treatment with IP.

4.2.4 Rationale for exclusion of male patients from study

Male breast cancer is rare and biologically heterogeneous. These rare patients will be excluded from this study, as safety results may be difficult to interpret.

4.3 Justification for dose

For information on dose modifications for each study treatment, see Section 6.5.

4.3.1 Durvalumab dose rationale, including rationale for fixed dosing

Based on available PK and safety data for weight-based doses of 10 mg/kg q2w and 20 mg/kg q4w and simulated PK data for a fixed dose of 1500 mg q4w, the fixed-dosing regimen was predicted to provide comparable exposure to the body weight-based dosing regimens. Durvalumab has a flat exposure-response (efficacy and safety) relationship. Based on all the observed and simulated data, a fixed-dosing regimen of 1500 mg q4w was predicted to provide similar efficacy and safety profiles as the body weight-based dosing regimens. Therefore, a fixed-dosing regimen of 1500 mg q4w was selected for Arms 1 through 5 in this study. In Arms 6 (durvalumab + DS-8201a) 7 and 8 (durvalumab + Dato-DXd), a fixed dose of 1120 mg durvalumab q3w is included in the current study. This dose is used in multiple studies across the durvalumab clinical program and is equivalent to the dose intensity being used in all other study arms. A dose of 1120 mg rather than 1125 mg is being used for practical reasons (ie, due to the vial sizes available).

For additional details on the pre-clinical and clinical data that informed durvalumab dose selection, see the durvalumab Investigator's Brochure.

For information on dose modifications for durvalumab (dose delays only), see Section 6.5.2.

4.3.2 Paclitaxel dose rationale

In a meta-analysis of randomized, controlled studies in advanced breast cancer (5 studies, 1471 patients), weekly administration of paclitaxel was compared to administration of every 3 weeks. The analysis concluded that there was an improvement in OS with weekly paclitaxel (pooled HR, 0.78; 95% CI: 0.67 to 0.89; p=0.001). Additionally, the incidence of neutropenia, neutropenic fever, peripheral neuropathy, and other SAEs was significantly lower with weekly paclitaxel compared to paclitaxel every 3 weeks (Mauri et al 2010). Based on this meta-analysis, paclitaxel in a 4-week cycle (3 weeks once weekly and 1 week off) was chosen.

Paclitaxel will be administered at an SoC dose level of 90 mg/m^2 in 4-week cycles as follows: 3 weeks once weekly (Days 1, 8, and 15) and 1 week off.

For Arm 2 (durvalumab + paclitaxel + capivasertib), the standard dose of weekly paclitaxel (for 3 weeks of 4-weekly cycles) has been amended (in Version 4 of the protocol) to 80 mg/m^2 to align with the capivasertib Phase III trial CAPItello-290 (NCT 03997123) (a Phase III randomized study assessing the efficacy and safety of capivasertib and paclitaxel versus placebo and paclitaxel as first-line treatment for patients with histologically confirmed, locally advanced (inoperable) or metastatic TNBC), which was initiated when the current study was already ongoing. The choice of such paclitaxel dose in the global CAPItello-290 study was determined based on the fact that, while both 80 mg/m^2 and 90 mg/m^2 doses are commonly used in clinical trials and both are considered as standard doses for paclitaxel in metastatic TNBC worldwide, 80 mg/m^2 is more frequently used in clinical practice (NCCN Guidelines V3.2019) (especially in Asian countries); with the intent to minimize toxicity and the discontinuation rate for AEs with no expected detriment on efficacy, this is considered particularly relevant in the context of a triplet combination with durvalumab + capivasertib + paclitaxel.

For information on dose modifications for paclitaxel, see Section 6.5.2.2.

4.3.3 Capivasertib dose rationale

Capivasertib will be administered orally at a dose of 400 mg bid on an intermittent weekly dosing schedule in 4 week cycles as follows: administered on Days 2 through 5 of Weeks 0, 1, and 2 (ie, 4 days on and 3 days off each week) followed by 1 week off treatment.

This dose regimen was selected as the combination dose with weekly paclitaxel for further evaluation based on the BEECH study, which evaluated 2 intermittent weekly dosing schedules (2 days on/5 days off treatment and 4 days on/3 days off treatment) of capivasertib taken bid at ascending doses 3 weeks on and 1 week off in combination with weekly paclitaxel 90 mg/m2 (Days 1, 8, and 15 of a 28-day cycle). Although this Phase IIB combination regimen failed to demonstrate efficacy in ER+ BC patients (BEECH Part B), clinical efficacy was proven in TNBC (PAKT study, which showed a significantly longer PFS

and OS with capivasertib + paclitaxel versus with paclitaxel alone). Moreover, based on these 2 randomized Phase II studies, capivasertib appeared to be well tolerated overall, with no marked impact on the tolerability and dose intensity of paclitaxel.

For additional details on the pre-clinical and clinical data on capivasertib dose selection, see the capivasertib Investigator's Brochure.

For information on dose modifications for capivasertib, see Section 6.5.2.3.

4.3.4 Oleclumab dose rationale

The oleclumab initial dose of 3000 mg q2w for the first 2 cycles, followed thereafter by q4w dosing starting at Cycle 3, was selected based on the available clinical safety, tolerability, efficacy, PK, and pharmacodynamic data from the Phase I Study D6070C00001 (NCT02503774) and from population PK modeling and simulation analysis results. In Study D6070C00001, doses of [redacted], and [redacted] mg/kg q2w were evaluated both as oleclumab monotherapy and in combination with durvalumab [redacted] mg/kg q2w. Oleclumab was well tolerated, and there were no observed DLTs either as monotherapy or in combination with durvalumab at any dose level. The oleclumab [redacted] mg/kg q2w dose was identified for evaluation with durvalumab [redacted] mg/kg q2w in the dose expansion phase of Study D6070C00001.

Population PK analysis with data from 97 patients in Study D6070C00001 indicated only a moderate impact of body weight on the PK of oleclumab (coefficient of [redacted] on clearance and volume of distribution); however, the change expected in PK parameters due to body weight was not significant at approximately [redacted] Therapy arms (monotherapy/combination) or disease type (CRC or pancreatic adenocarcinoma) did not impact the PK parameters significantly. To determine the impact of body weight-based dosing of [redacted] mg/kg q2w or fixed dosing of 3000 mg q2w on oleclumab PK exposure, simulations were conducted [redacted] and predicted steady-state concentrations (5th, median, and 95th percentiles) were compared. Simulation results demonstrated that a body weight-based dose of [redacted] mg/kg and a fixed dose of 3000 mg yield similar steady-state PK concentrations with similar overall between-patient variability. Additionally, the highest concentrations predicted following a fixed dose of 3000 mg q2w were lower than the well-tolerated highest exposure observed at [redacted] mg/kg q2w dosing of approximately [redacted] µg/mL. The predicted similarity of exposure following either dosing regimen is consistent with literature, showing that an exponent of the covariate model for body weight approximately 0.5 is expected to yield similar median PK profiles and population variability (Wang et al 2009). However, due to the ease of use and reduced potential dosing errors, a fixed-dosing approach is preferable. Based on analyses of safety, PK, and preliminary efficacy in Study D6070C00001, an RP2D of 3000 mg ([redacted] mg/kg) oleclumab was selected.

For additional details on the pre-clinical and clinical data on oleclumab dose selection, see the oleclumab Investigator's Brochure.

For information on dose modifications for oleclumab, see Section 6.5.2.4.

4.3.5 DS-8201a (trastuzumab deruxtecan) dose rationale

Based on all available information to date, a DS-8201a dose of 5.4 mg/kg q3w has been chosen for this study. Doses of 5.4 mg/kg and 6.4 mg/kg DS-8201a monotherapy have been tested in clinical studies; both showed efficacy in different tumor types and neither reached the MTD. A numerically higher incidence of ILD/pneumonitis was observed with 6.4 mg/kg compared to 5.4 mg/kg in breast cancer study populations. DS-8201a is currently being evaluated in two Phase I studies in combination with an anti-PD1 antibody (Study DS-8201-A-U105; NCT03523572 [completed]; DS8201-A-U106; NCT04042701). The recommended Phase II dose of 5.4 mg/kg for breast cancer will be evaluated with durvalumab.

For additional details on the non-clinical and clinical data on DS-8201a dose selection, see the DS-8201a Investigator's Brochure.

For information on dose modifications for DS-8201a, see Section 6.5.2.5.

4.3.6 Dato-DXd (datopotamab deruxtecan; DS-1062a) dose rationale

In the Phase 1 study (DS1062-A-J101), both 4.0- and 6.0-mg/kg doses were well tolerated and showed strong efficacy results in the NSCLC and TNBC cohorts, with a generally comparable exposure - safety relationship:

- Positive exposure-response relationships were identified between Dato-DXd exposure and efficacy endpoints (ORR and PFS).
- The preliminary exposure-ORR relationship in TNBC appears to be similar to that in NSCLC.

The 6 mg/kg dose was therefore selected for the treatment of TNBC participants in this study. Efficacy and safety results from the 6.0 mg/kg dose are presented in Section 2.2.9. These data indicate a manageable safety profile at the 6.0 mg/kg dose, and further study in breast cancer warranted.

For information on dose modifications for Dato-DXd, see Section 6.5.2.6.

4.4 End of study definition

For the purpose of Clinical Trial Transparency the definition of the end of the study differs under FDA and EU regulatory requirements:

- European Union requirements define study completion as the last visit of the last subject for any protocol related activity.
- Food and Drug Administration requirements defines 2 completion dates:
  - Primary Completion Date – the date that the final participant is examined or receives an intervention for the purposes of final collection of data for the primary outcome measure, whether the clinical study concluded according to the pre-specified protocol or was terminated. In the case of clinical studies with more than one primary outcome measure with different completion dates, this term refers to the date on which data collection is completed for all of the primary outcomes.
  - Study Completion Date – is defined as the date the final participant is examined or receives an intervention for purposes of final collection of data for the primary and secondary outcome measures and AEs (for example, last participant's last visit), whether the clinical study concludes according to the pre-specified protocol or is terminated.

The end of study is defined as the date of the last scheduled procedure shown in the schedule of activities for the last patient in the study.

A patient is considered to have completed the study if they have completed all phases of the study including the last scheduled procedure shown in the SoA (including follow up for OS).

Patients may be withdrawn from the study if the study itself is stopped. The study may be stopped if, in the judgment of AstraZeneca, study patients are placed at undue risk because of clinically significant findings.

4.5 Study termination

The study may be stopped if, in the judgement of AstraZeneca, study patients are placed at undue risk because of the following clinically significant findings:

- Meet individual stopping criteria or are otherwise considered significant
- Assessed as causally related to study treatment
- Not considered to be consistent with continuation of the study

As this study is testing novel investigational products, emerging information from this study or another study that impacts the safety or the benefit/risk profile may lead to the permanent closure of any of the study arms.

Regardless of the reason for termination, all data available for the patient at the time of discontinuation of follow-up must be recorded in the electronic case report form (eCRF).

All reasons for discontinuation of treatment must be documented.

See Appendix A-6 for guidelines for the dissemination of study results.

4.6 Site closure

The Sponsor or designee reserves the right to close the study site at any time for any reason at the sole discretion of the Sponsor. Study sites will be closed upon study completion. A study site is considered closed when all required documents and study supplies have been collected and a study-site closure monitoring visit has been performed.

The Investigator may initiate study site closure at any time, provided that there is reasonable cause and that sufficient notice is given in advance of the intended termination.

Reasons for the early closure of a study site by the Sponsor or Investigator may include, but are not limited to, the following:

- Failure of the Investigator to comply with the protocol, the requirements of the Institutional Review Board (IRB)/Independent Ethics Committee (IEC) or local health authorities, the Sponsor's procedures, or Good Clinical Practice guidelines
- Inadequate recruitment of participants by the Investigator
- Discontinuation of further study intervention development

5 STUDY POPULATION

Prospective approval of protocol deviations to recruitment and enrollment criteria, also known as protocol waivers or exemptions, is not permitted.

Each patient should meet all of the inclusion criteria and none of the exclusion criteria for this study in order to be assigned/randomized to a study intervention. Under no circumstances can there be exceptions to this rule. Patients who do not meet the entry requirements are screen failures; refer to Section 5.4.

For procedures for withdrawal of incorrectly enrolled patients see Section 6.2.2.

5.1 Inclusion criteria

Informed consent

1 Capable of giving signed informed consent, which includes compliance with the requirements and restrictions listed in the ICF and in this protocol.

2 Provision of signed and dated, written ICF prior to any mandatory study-specific procedures, sampling, and analyses

3 Prior to collection of the sample for genetic analysis, which is an optional assessment, provision of [redacted] and analysis in the appropriate section of the signed and dated written informed consent

The ICF process is described in Appendix A-3.

Age

4 Age ≥18 years at the time of screening

Type of patient and disease characteristics

5 Locally confirmed advanced/unresectable or metastatic TNBC, determined from the most recent tumor sample taken for diagnostic purposes (accompanied by an associated pathology report), defined as the following. (The local testing facility must be acceptable for Investigator determination of patient eligibility.)

- Negative for ER with <1% of tumor cells positive for ER on IHC or IHC score (Allred) of ≤2,
- Negative for PR with <1% of tumor cells positive for PR on IHC or IHC score (Allred) of ≤2 or PR unknown, and
- Negative for HER2 with 0, or 1+ intensity on IHC, or 2+ intensity on IHC and no evidence of amplification on in situ hybridization as per the American Society of Clinical Oncology-College of American Pathologists HER2 guideline (Hammond et al 2010, Wolff et al 2018)

6 No prior treatment for metastatic or locally advanced unresectable TNBC. Prior treatment with curative intent for Stage I to III TNBC (that subsequently became metastatic) is acceptable if it meets the following criteria at the time of screening:

- ≥6 months elapsed between the completion of treatment (eg, date of primary breast tumor surgery or the date of the last adjuvant chemotherapy administration, whichever occurred last) and the first documented distant disease recurrence
- ≥12 months since the last administration of taxane
- [redacted]

7 World Health Organization (WHO)/Eastern Cooperative Oncology Group (ECOG) performance status (PS) of 0 or 1 at enrollment

8 (Inclusion Criterion 8 was removed following Protocol Amendment 1.)

9 At least 1 lesion, not previously irradiated, that qualifies as a RECIST 1.1 target lesion (TL) at baseline. Tumor assessment by CT scan or MRI must be performed within 28 days prior to treatment assignment/randomization.

10 Adequate organ and marrow function as defined below:

- Hemoglobin ≥[redacted] g/dL
- Absolute neutrophil count ≥[redacted] × 10^9/L
- Platelet count ≥[redacted] × 10^9/L
- Serum bilirubin ≤[redacted] × the ULN (<[redacted] × ULN if with documented [redacted] for patients in Arms [redacted]
- ALT and AST ≤[redacted] × ULN. In Arm 8, patients with hepatitis B virus (HBV) infection, characterized by positive hepatitis B surface antigen (HBsAg) and/or anti-hepatitis B core antibodies (HBcAb) must have normal ALT and AST or ≤[redacted] × ULN if liver metastases are present and the elevation is not attributable to HBV infection
- Measured creatinine clearance [redacted] mL/min calculated by Cockcroft-Gault (using actual body weight) or by measured 24-hour urine collection

11 Provision of a FFPE tumor sample is mandatory. A recently acquired sample is preferred if available per site's routine clinical practice. Otherwise, a pre-treatment assignment/pre-randomization archival tumor sample can be provided if [redacted] A FFPE tissue block is preferred over sections. If it is not possible to provide a tissue block, freshly-cut unstained serial tumor sections [redacted] are to be provided. Specimens

with limited tumor content, fine needle aspirates and samples obtained from metastatic sites in bone are inadequate for testing and not acceptable. For additional details on sample requirements, see Section 8.8.1 and the Laboratory Manual.

Weight

12 Body weight >35 kg

Sex

13 Part 1 and Part 2: Female

Patients enrolled in Arm 6 (durvalumab + DS-8201a) must also meet the following criteria:

14 Must provide documentation of locally determined advanced/unresectable or metastatic TNBC with HER2-low tumor expression (IHC 2+/ISH–, IHC 1+/ISH–, or IHC 1+/ISH untested) (note: ISH may be determined by either fluorescence in situ hybridization [FISH] or dual in situ hybridization [DISH])

15 Serum albumin [redacted] g/dL

16 International normalized ratio (INR)/prothrombin time (PT) and either partial thromboplastin (PTT) or activated partial thromboplastin time (aPTT) [redacted] × ULN

Patients enrolled in Arms 7 and 8 (durvalumab + Dato-DXd) must also meet the following criteria in addition to criteria 1 to 13:

17 INR/PT and either PTT or aPTT [redacted] × ULN

Patients enrolled in Arm 8 (durvalumab + Dato-DXd) must also meet the following criterion in addition to criteria 1 to 13 and 17:

18 PD-L1 positive tumor as determined by an IHC based assay. Pre-existing test results are acceptable. Where no result exists a positive local test result must be obtained during pre-screening.

5.2 Exclusion criteria

Medical conditions

1 History of allogeneic organ transplantation.

2 Active or prior documented autoimmune or inflammatory disorders [redacted]:

   - [redacted]
   - [redacted]

- CCI [redacted]
- CCI [redacted]
  CCI [redacted].
- CCI [redacted].

3 Uncontrolled intercurrent illness, including, but not limited to, ongoing or active infection, symptomatic congestive heart failure, uncontrolled hypertension, unstable angina pectoris, uncontrolled cardiac arrhythmia, active ILD, serious chronic gastrointestinal conditions associated with diarrhea, chronic diverticulitis or previous complicated diverticulitis, or psychiatric illness/social situations that would limit compliance with study requirements, substantially increase risk of incurring AEs, or compromise the ability of the patient to give written informed consent

4 History of another primary malignancy, except for

- CCI [redacted]
  CCI [redacted]
- CCI [redacted]
  CCI [redacted]
- CCI [redacted]

5 History of leptomeningeal carcinomatosis.

6 History of active primary immunodeficiency.

7 Active infection including:

- Tuberculosis (clinical evaluation that includes clinical history, physical examination and radiographic findings, and tuberculosis testing in line with local practice),
- Known HBV infection:
  - CCI [redacted]
  - [redacted]

  - [redacted]

- Hepatitis C infection [redacted]
- Human immunodeficiency virus (HIV) infection:
  - [redacted]

Some medications used for these conditions have drug-drug interactions with the study treatment.

8 Any unresolved toxicity NCI CTCAE Grade ≥[redacted] from previous anticancer therapy with the exception of [redacted], and the laboratory values defined in the inclusion criteria.

  - Patients with irreversible toxicity not reasonably expected to be exacerbated by treatment with IP may be included only after consultation with the Study Clinical Lead.

9 Has untreated CNS metastases identified either on the baseline brain imaging (see Appendix F [RECIST]) for details on the imaging modality) obtained during the screening period or identified prior to signing the ICF. Patients whose brain metastases have been treated may participate, provided they show radiographic [redacted] In addition, any [redacted] as a result of either the brain metastases or their treatment [redacted] Brain metastases will not be recorded as RECIST TLs at baseline.

10 [redacted]

11 Any clinically important abnormalities in rhythm, conduction, or morphology of resting ECG [redacted]

12 Patients enrolled in Arm 2 only: Any factors that increase the risk of QTc prolongation or risk of arrhythmic events, such as heart failure, hypokalemia, potential for torsades de pointes, congenital long QT syndrome, family history of long QT syndrome or unexplained sudden death under 40 years of age, or any concomitant medication known to prolong the QT interval (see Appendix I for list of QT prolonging medications).

13 Experience of any of the following procedures or conditions in the preceding 6 months: [redacted]

14 Known allergy or hypersensitivity to any of the study drugs or any of the study drug excipients.

15 Patients enrolled in Arm 5 only: History of venous thromboembolism in the past 3 months.

Prior/concomitant therapy

16 Any concurrent chemotherapy, IP, or biologic for cancer treatment. [redacted]

17 Prior exposure to immune-mediated therapy, including but not limited to, other anti-CTLA-4, anti-PD-1, [redacted], or anti-PD-L2 antibodies, [redacted]

18 (Exclusion criterion 18 was removed following Protocol Amendment 1.)

19 Receipt of live attenuated vaccine within [redacted] days prior to the first dose of IP. Note: Patients, if enrolled, should not receive live vaccine while receiving IP and up to [redacted] days after the last dose of IP. [redacted]

20 Major surgical procedure (as defined by the Investigator) within [redacted] [redacted] prior to the first dose of IP. [redacted].

21 Current or prior use of immunosuppressive medication within [redacted] days before the first dose of IP. The following are exceptions to this criterion:

- [redacted]
- [redacted]

– [redacted]

22 "(Exclusion Criterion 22 was removed in Protocol Version 3.)"

23 Patients enrolled in Arm 2 only: Potent inhibitors or inducers or substrates of CYP3A4 or substrates of CYP2C9 or CYP2D6 within 2 weeks before the first dose of study treatment (3 weeks for St John's Wort) (see Appendix K for further guidance).

Prior/concurrent clinical study experience

24 Participation in another clinical study with an IP administered in the last [redacted] days.

25 Previous IP assignment in the present study.

26 Concurrent enrollment in another clinical study, unless it is an observational (non-interventional) clinical study or during the follow-up period of an interventional study.

27 Prior randomization or treatment in a previous durvalumab clinical study, regardless of treatment arm assignment.

Other exclusions

28 Female patients who are pregnant, breastfeeding or of reproductive potential who are not willing to employ highly effective birth control from screening to [redacted] months after the last dose of durvalumab, [redacted] months after the last dose of capivasertib, oleclumab, or paclitaxel and at least [redacted] months after the final administration of DS-8201a or Dato-DXd.

29 Judgment by the Investigator that the patient should not participate in the study if the patient is unlikely to comply with study procedures, restrictions, and requirements.

30 Patients enrolled in Arm 2 only: Prior treatment with PI3K inhibitors, AKT inhibitors, or mammalian target of rapamycin (mTOR) inhibitors.

31 Patients enrolled in Arm 2 only: Clinically significant abnormalities of glucose metabolism, as defined by any of the following:

- Patients with diabetes mellitus Type I or diabetes mellitus Type II requiring insulin treatment
- HbA1c ≥[redacted]% ([redacted] mmol/mol)

32 For patients who provide [redacted]

33 Patients with baseline peripheral neuropathy Grade >1.

Additional criteria

34 Cardiac ejection fraction outside institutional range of normal or <50% (whichever is higher) as measured by echocardiogram ([ECHO] or multiple-gated acquisition [MUGA] scan if an ECHO cannot be performed or is inconclusive).

Patients enrolled in Arm 6 (durvalumab + DS-8201a) and Arms 7 and 8 (durvalumab + Dato-DXd) must not meet any of the following criteria:

35 Patients enrolled in Arms 7 and 8 only: Clinically significant corneal disease in the opinion of the Investigator.

36 History of (non-infectious) ILD/pneumonitis that required steroids, has current ILD/pneumonitis, or where suspected ILD/pneumonitis cannot be ruled out by imaging at screening

37 [redacted]

38 (Exclusion Criterion 38 was removed in Protocol Version 5).

39 Received radiation therapy including palliative stereotactic radiation therapy to chest ≤[redacted] weeks of enrollment or palliative stereotactic radiation therapy to other areas ≤[redacted] weeks of enrollment.

40 [redacted]

41 Use of chloroquine or hydroxychloroquine in <14 days prior to Day 1 of DS-8201a (Arm 6) or Dato-DXd (Arms 7 and 8) treatment (see Appendix O).

42 Prior treatment with ADC that comprises of an exatecan derivative that is a topoisomerase I inhibitor.

43 Has a history of severe hypersensitivity reactions to either the drug substances or inactive ingredients (including but not limited to polysorbate 80) of Dato-DXd or DS-8201a or has a history of severe hypersensitivity reactions to other monoclonal antibodies.

44 Patients enrolled in Arm 6 only: Previously been diagnosed as HER2+ or received HER2-targeted therapy.

5.3 Lifestyle restrictions

The following restrictions apply while the patient is receiving IP and for the specified times before and after:

1. Female patient of child-bearing potential

Female patients of childbearing potential who are not abstinent and intend to be sexually active with a non-sterilized male partner must use at least 1 **highly** effective method of contraception (Table 12) from the time of screening throughout the total duration of the drug treatment and the drug washout period ([redacted] after the last dose of durvalumab, [redacted] after the last dose of capivasertib, oleclumab, or paclitaxel; [redacted] after the last dose of DS-8201a or Dato-DXd). Non-sterilized male partners of a female patient of childbearing potential must use a male condom plus spermicide throughout this period. Cessation of birth control after this point should be discussed with a responsible physician. Periodic abstinence, the rhythm method, and the withdrawal method are not acceptable methods of contraception. Female patients should refrain from breastfeeding throughout this period. Women must not donate, or retrieve for their own use, ova from the time of screening and throughout the study treatment period, and for at least [redacted] months after the final administration of durvalumab, capivasertib, oleclumab, or paclitaxel and at least [redacted] months after the final administration of DS-8201a or Dato-DXd.

Note: Females of childbearing potential are defined as those who are not surgically sterile (ie, bilateral salpingectomy, bilateral oophorectomy, or complete hysterectomy) or post-menopausal.

Women will be considered post-menopausal if they have been amenorrheic for 12 months without an alternative medical cause. The following age-specific requirements apply:

- Women <50 years of age would be considered post-menopausal if they have been amenorrheic for 12 months or more following cessation of exogenous hormonal treatments and if they have luteinizing hormone and follicle-stimulating hormone levels in the post-menopausal range for the institution.
- Women ≥50 years of age would be considered post-menopausal if they have been amenorrheic for 12 months or more following cessation of all exogenous hormonal treatments, had radiation-induced menopause with last menses >1 year ago, or had chemotherapy-induced menopause with last menses >1 year ago.
- Women who are surgically sterile (ie, bilateral salpingectomy, bilateral oophorectomy, or complete hysterectomy) are eligible.

Highly effective methods of contraception, defined as one that results in a low failure rate (ie, less than 1% per year) when used consistently and correctly are described in Table 12.

Note that some contraception methods are not considered highly effective (eg, male or female condom with or without spermicide; female cap, diaphragm, or sponge with or without spermicide; non-copper containing intrauterine device; progestogen-only oral hormonal contraceptive pills where inhibition of ovulation is not the primary mode of action [excluding Cerazette/desogestrel which is considered highly effective]; and triphasic combined oral contraceptive pills).

All patients should also follow the local prescribing information relating to contraception, the time limits for such precautions, and any additional restrictions for paclitaxel.

Table 12 Highly effective methods of contraception (<1% failure rate)

Non-hormonal methods	Hormonal methods
• Total sexual abstinence (evaluate in relation to the duration of the clinical study and the preferred and usual lifestyle choice of the participant)<br>• Vasectomized sexual partner (with participant assurance that partner received post-vasectomy confirmation of azoospermia)<br>• Bilateral tubal occlusion<br>• Intrauterine device (provided coils are copper-banded)	• Injection: Medroxyprogesterone injection (eg, Depo-Provera®) [a]<br>• Levonorgestrel-releasing intrauterine system (eg, Mirena®) [a]<br>• Implants: Etonogestrel-releasing implants (eg, Implanon® or Nexplanon®)<br>• Intravaginal devices: Ethinylestradiol/etonogestrel-releasing intravaginal devices (eg, NuvaRing®)<br>• Combined pill: Normal and low dose combined oral contraceptive pill<br>• Patch: Norelgestromin/ethinylestradiol-releasing transdermal system (eg, Xulane®, Ortho Evra®)<br>• Mini pill: Progesterone-based oral contraceptive pill using desogestrel: Cerazette® is currently the only highly effective progesterone-based pill

[a] Hormonal methods not prone to drug-drug interactions.

2. All patients: Patients should not donate blood or blood components while participating in this study and through [redacted] after the last dose of durvalumab, capivasertib, oleclumab, DS-8201a, Dato-DXd, or paclitaxel or until alternate anticancer therapy is started.

3. Restrictions relating to concomitant medications and food are described in Section 6.4.

4. During study intervention and for [redacted] after the last dose of study intervention, participants should be advised to avoid prolonged exposure to the sun, wear protective clothing including a hat, and seek shade from the sun as much as possible; in addition, SPF30+ sunscreen should be used. Exposure to other sources of ultraviolet light including sun beds and tanning booths, etc, should be avoided.

5. Patients enrolled in Arms 6 (durvalumab + DS-8201a) and 7 and 8 (durvalumab + Dato-DXd): use of tobacco products and e-cigarettes and vaping is strongly discouraged but not prohibited.

5.4 Screen failures

Screen failures are patients who do not fulfill the eligibility criteria for the study, and therefore must not be assigned treatment or randomized. These patients should have the reason for study withdrawal recorded as “eligibility criteria not fulfilled” (ie, patient does not meet the required inclusion/exclusion criteria). This reason for study withdrawal is only valid for screen failures (ie, not randomized patients). Patients may be rescreened a single time, but they may not be re-allocated to treatment.

A minimal set of screen failure information is required to ensure transparent reporting of screen failure patients to meet the Consolidated Standards of Reporting Trials publishing requirements and to respond to queries from regulatory authorities. Minimal information includes demography, screen failure details, eligibility criteria, and any SAEs, including patients who are identified in the pre-screening phase as not eligible.

6 STUDY TREATMENTS

Study treatment is defined as any IP(s) (including marketed product comparator and placebo) or medical device(s) intended to be administered to a study participant according to the study protocol. Study treatment in this study refers to durvalumab, paclitaxel, and any of the novel oncology therapies. In arms with an IP that is given IV (ie, oleclumab, DS-8201a, or Dato-DXd), the IP should be administered first, followed by durvalumab, and then paclitaxel (Arms 1 through 5 only).

6.1 Treatments administered

6.1.1 Investigational products

Table 13 summarizes all study IPs. Additional details are provided on the administration of all IV-administered IP in Section 6.1.1.1 and on each individual IP in Section 6.1.1.2 through Section 6.1.1.6. The treatment regimens are described in Section 6.1.2, with the individual treatment arms for Parts 1 and 2 of the study summarized in Table 15.

Table 13 Study treatments

Study treatment name	Dosage formulation	Dosing instructions	Route of administration	Packaging and labeling	Provider
Durvalumab (MEDI4736)<br>Target: PD-L1	500 mg vial solution for infusion after dilution, 50 mg/mL	**Arms 1 to 5:**<br>1500 mg q4w<br><br>**Arms 6, 7, and 8:**<br>1120 mg q3w	IV	Study treatment will be provided in 500 mg vials. Each 500 mg vial will be labeled in accordance with GMP Annex 13 and per country regulatory requirement.[a]	MedImmune
Paclitaxel	Variable	**Arms 1 and 5:**<br>90 mg/m^2<br>**Arm 2[b]:**<br>80 mg/m^2<br><br>4-week cycles: 3 weeks once weekly (D1, D8, and D15) and 1 week off	IV	Variable	Sourced locally by site[c]
Capivasertib[d,e]<br>(AZD5363)<br>Target: AKT	200 mg film-coated tablets (160 mg film-coated tablets for dose reduction)	400 mg bid 4 days on (D2, D3, D4, and D5) and 3 days off<br>4-week cycles: 3 weeks on (intermittent; see above) and 1 week off	Oral	Study treatment will be provided in bottles of 60 tablets for the 200 mg and 160 mg tablets. Each bottle will be labeled in accordance with GMP Annex 13 and per country regulatory requirement.	AstraZeneca

Table 13 Study treatments

Study treatment name	Dosage formulation	Dosing instructions	Route of administration	Packaging and labeling	Provider
Oleclumab[d] (MEDI9447) Target: CD73	[redacted], [redacted]	3000 mg q2w for first 2 cycles (D1 and D15 of C1 and C2), then q4w starting at C3 (D1)	IV	Study treatment will be provided in [redacted] mg and [redacted] mg vials. Each [redacted] mg and [redacted] mg vial will be labeled in accordance with GMP Annex 13 and per country regulatory requirement.[c]	MedImmune
DS-8201a[d] (trastuzumab deruxtecan; T-DXd) Target: HER2	Powder for concentrate for solution for infusion, [redacted] mg/vial	5.4 mg/kg q3w	IV	Study treatment will be provided in [redacted] mg vials. Each [redacted] mg vial will be labeled in accordance with GMP Annex 13 and per country regulatory requirement.[c]	Daiichi-Sankyo
Dato-DXd[d] (datopotamab deruxtecan; DS-1062a) Target: TROP2	Powder for concentrate for solution for infusion, [redacted] mg/vial	6.0 mg/kg q3w	IV	Study treatment will be provided in cartons containing 1 vial of Dato-DXd powder for injection [redacted] labeled in accordance with GMP Annex 13 and per country regulatory requirement.[c]	Daiichi-Sankyo

[a] Label text prepared for durvalumab (MEDI4736) will show the product name as "MEDI4736" or "durvalumab (MEDI4736)," depending on the agreed product name used in the approved study master label document. All naming conventions are correct during this transitional period.

[b] For patients on Arm 2 who were treated prior to Protocol Version 4, dosing may continue unchanged (ie, if the patient was treated at starting dose 90 mg/m^2 without dose modifications, they may continue at this dose, as clinically indicated).

[c] Under certain circumstances when local sourcing is not feasible, paclitaxel may be supplied centrally through AstraZeneca.

[d] Label text prepared for capivasertib (AZD5363) will show the product name as "AZD5363" or "capivasertib (AZD5363)," label text for oleclumab (MEDI9447) will show "MEDI9447" or "oleclumab (MEDI9447)," label text for DS-8201a (trastuzumab deruxtecan) will show "DS-8201a," and label text

for Dato-DXd (datopotamab deruxtecan) will show "DS-1062a"or "Dato-DXd" depending on the agreed product name used in the respective approved study master label document. All naming conventions for these compounds are correct during this transitional period.

[e] Capivasertib should not be taken during any week that the paclitaxel infusion is not administered, either due to a scheduled off-drug-week or an unscheduled omission or delay.

bid Twice daily; C Cycle; CD Cluster of differentiation; D Day; GMP Good Manufacturing Practice; IV Intravenous; MEK Mitogen-activated protein kinase/extracellular signal-regulated kinase; PD-L1 Programmed cell death ligand 1; q1w Every 1 week; q2w Every 2 weeks; q3w Every 3 weeks; q4w Every 4 weeks; STAT3 Signal transducer and activator of transcription 3.

6.1.1.1 Guidelines applicable to all treatments administered IV

IV bag contents should be equilibrated to room temperature (approximately 20°C to 25°C) to avoid IRR symptoms due to the administration of the solution at low temperatures.

A physician must be present at the site or immediately available to respond to emergencies during all administrations of IP given via IV infusion. Fully functional resuscitation facilities should be available. The IP must not be administered via IV push or bolus but as a slow IV infusion.

In the event of an IRR with any IP administered IV, the durvalumab combination therapy guidelines may be followed to manage the toxicity by reducing the infusion rate or discontinuing infusion, as applicable (see Section 8.4.6).

6.1.1.2 Durvalumab (MEDI4736)

Durvalumab (MEDI4736) will be supplied by AstraZeneca as a 500 mg vial solution for infusion after dilution. The solution contains 50 mg/mL durvalumab (MEDI4736), [redacted] mM histidine/ histidine-hydrochloride, [redacted] mM trehalose dihydrate, and [redacted] polysorbate 80; it has a pH of 6.0 and a density of 1.054 g/mL. The label-claim volume is 10 mL.

Durvalumab is a sterile, clear to opalescent, colorless to slightly yellow solution, and free from visible particles. Each vial selected for dose preparation should be inspected.

IP vials are stored at 2°C to 8°C (36°F to 46°F) and must not be frozen. Investigational product must be kept in original packaging until use to prevent prolonged light exposure. A temperature log will be used to record the temperature of the storage area. Temperature excursions outside the permissible range listed in the clinical supply packaging are to be reported to the monitor upon detection. A calibrated temperature monitoring device will be used to record the temperature conditions in the drug storage facility. Storage conditions stated in the IB may be superseded by the label storage.

The investigator or designee (eg, pharmacist) must confirm appropriate temperature conditions have been maintained during transit for all study intervention received at the site and throughout the entire study duration until authorisation is provided for on-site destruction or removal of the study intervention, reflecting completion of the study. In the event of a temperature excursion detected at any time during the study, sites will follow the reporting procedures for notifying the sponsor (or designated party); release of study intervention for clinical use can only occur once the event has been reviewed and approval is provided by the sponsor (or designated party).

Preparation and administration of durvalumab (MEDI4736) doses for IV infusion

The dose of durvalumab (MEDI4736) for administration must be prepared by the Investigator or site's designated IP manager using an aseptic technique. The total time from needle puncture of the durvalumab (MEDI4736) vial to the start of administration must not exceed:

- 24 hours at 2°C to 8°C (36°F to 46°F)
- 4 hours at room temperature
- If the final product is stored at both refrigerated and ambient temperatures, the total time must not exceed 24 hours.

A dose of 1500 mg (for patients >30 kg in weight) for Arms 1 through 5 every 4 weeks or a dose of 1120 mg for Arms 6, 7, and 8 every 3 weeks, will be administered using an IV bag containing 0.9% (w/v) saline or 5% (w/v) dextrose, with a final durvalumab (MEDI4736) concentration ranging from 1 to 15 mg/mL, and delivered through an IV administration set with a 0.2 or 0.22 µm filter. Add 30 mL (ie, 1500 mg) or 22.4 mL (ie, 1120 mg) of durvalumab (MEDI4736) to the IV bag. The IV bag size should be selected such that the final concentration is within 1 to 15 mg/mL. The bag should be mixed by gently inverting to ensure homogeneity of the dose in the bag.

If a patient's weight falls to ≤30 kg after initiation of study treatment, weight-based dosing at 20 mg/kg (Arms 1 through 5 only) will be administered using an IV bag size selected such that the final durvalumab concentration is within 1 to 15 mg/mL. Arms 6, 7, and 8 would not require weight-based dosing because the weight restriction is due to endotoxin limits per dose and is not applicable for doses <1500 mg.

The use of elastomeric pumps and use of a pneumatic tube for transport of either a vial or a final prepared product have not been studied and therefore should be avoided.

The standard infusion time is 1 hour ± 10 minutes; however, if there are interruptions, the total allowed time must not exceed 8 hours with the infusion bag maintained at room temperature, otherwise a new dose must be prepared from new vials.

Do not co-administer other drugs through the same infusion line.

The IV line will be flushed according to local practices to ensure that the full dose is administered. Infusion time does not include the final flush time.

If either preparation time or infusion time exceeds the time limits, a new dose must be prepared from new vials. Durvalumab (MEDI4736) does not contain preservatives, and any unused portion must be discarded.

For detailed dose preparation, refer to the Handling Instructions document provided by AstraZeneca.

6.1.1.3 Paclitaxel

The SoC agent, paclitaxel, will either be locally sourced or be centrally supplied by AstraZeneca and will be administered according to prescribing information or treatment guidance in general use by the investigating site. Under certain circumstances when local sourcing is not feasible, AstraZeneca will centrally supply the drug, which will be labeled with text translated in the local language in accordance with regulatory guidelines.

Administration of paclitaxel

A dose of 90 mg/m^2 will be administered IV over 1 hour for Arms 1 and 5 and a dose of 80 mg/m^2 will be administered IV over 1 hour for Arm 2. For patients on Arm 2 who were treated prior to Protocol Version 4, dosing may continue unchanged (ie, if the patient was treated at a starting dose of 90 mg/m^2 without dose modifications, they may continue at this dose, as clinically indicated).

Premedications will be administered 30 to 60 minutes prior to therapy and may include an antihistamine (such as diphenhydramine 50 mg administered IV) and an H_2 blocker (such as ranitidine 50 mg IV or cimetidine 300 mg IV). Corticosteroids (such as dexamethasone 20 mg) will be administered orally 12 and 6 hours or IV 30 to 60 minutes before paclitaxel. If no hypersensitivity reactions occur after the first paclitaxel dose, corticosteroid dose reductions are permitted at the Investigator’s discretion.

6.1.1.4 Capivasertib (AZD5363)

Capivasertib (AZD5363) will be supplied by AstraZeneca and labeled and distributed to sites by Fisher Clinical Services. Capivasertib will be supplied in bottles of 60 tablets at strengths of 200 mg and 160 mg (for dose reductions). Additional information about the IP may be found in the capivasertib Investigator’s Brochure.

Capivasertib tablets should be stored at room temperature, in their original packaging until use.

Administration of capivasertib

Capivasertib will be administered orally at a dose of 400 mg bid on an intermittent weekly dosing schedule in 4-week cycles as follows: administered on Days 2 through 5 of Weeks 0, 1, and 2 (ie, 4 days on and 3 days off each week), followed by 1 week off treatment.

For Cycle 1, prednisone 10 mg (or equivalent) should be taken on Days 2 through 5 of Week 0.

When possible, all doses of capivasertib should be taken at approximately the same time each day (and 12 hours apart in the same day) and in a fasted state (water to drink only) from at least 2 hours prior to the dose to at least 1 hour post-dose. If vomiting occurs, a replacement dose should not be taken, and the patient should take their allotted dose at the next scheduled time.

Should a patient miss a scheduled dose, the patient will be allowed to take the dose up to a maximum of 2 hours after the scheduled dose time. If more than 2 hours after the scheduled dose time have passed, the missed dose should not be taken, and the patient should take his/her allotted dose at the next scheduled time. If a patient needs to take the dose earlier for whatever reason, the patient can take the dose up to 2 hours earlier than the scheduled dose time. The patient should make every reasonable effort to take the capivasertib tablet(s) on time.

If the paclitaxel infusion is not administered, then capivasertib can be interrupted or continued based on Investigator discretion. If capivasertib is continued on a weekly schedule, there should be 3 days off between capivasertib and subsequent paclitaxel administration.

If treatment with paclitaxel is permanently discontinued, capivasertib may be continued on a weekly schedule at the Investigator's discretion. If at the Investigator's discretion the patient should continue to receive capivasertib following withdrawal from paclitaxel, any unused capivasertib must be returned to the pharmacy, and a new supply must be dispensed. Accountability must be captured in the appropriate accountability log.

Because of the known potential for allergic reactions to paclitaxel, premedication is required for paclitaxel per local practice (see Section 6.1.1.3). Routine premedication is not required for capivasertib beyond prednisone 10 mg (or equivalent) on Days 2 through 5 in Cycle 1 for general prophylaxis of rash. The following therapies are recommended during study participation, as applicable: prophylactic anti-emetic therapy before and after paclitaxel treatment should be given, as needed, on a prophylactic and treatment basis in compliance with the standards of the center; no prophylactic anti-emetic therapy is planned for capivasertib, but standard anti-emetic therapy, including a 5-hydroxytryptamine receptor (5-HT3)-antagonist, can be given, as needed, on a prophylactic and treatment basis in compliance with the standards of the center's local policy.

Administration of capivasertib during the safety run-in period

Capivasertib administration at the standard dose (dose level 1) and in the event of de-escalation (dose level -1) during the safety run-in period is summarized in Table 14.

Table 14 Capivasertib dispensation schedule in safety run-in period

Dose level	1	-1
Daily dose regimen of capivasertib	400 mg bid<br>4 days on (D2, D3, D4, and D5) and 3 days off in 4-week cycles: 3 weeks on (intermittent; see above) and 1 week off	[redacted] mg bid<br>4 days on (D2, D3, D4, and D5) and 3 days off in 4-week cycles: 3 weeks on (intermittent; see above) and 1 week off
Number of tablets per day	4 tablets 200 mg<br>2 tablets 200 mg bid	[redacted] tablets [redacted] mg<br>[redacted] tablets [redacted] mg bid
Number of tablets per bottle	60 ct bottles	60 -ct/[redacted] bottles
Number of tablets per cycle	48 tablets	48 tablets ([redacted] mg)
Number of bottles to be dispensed per cycle	One 60 ct/[redacted] bottles	One 60-ct/[redacted] bottles

bid: Twice daily; ct: Count

The dose and dose scheduling of capivasertib administered may be modified until the recommended dose for capivasertib in the combination therapy arm has been defined by the SRC. The daily dose of capivasertib, number of tablets per day, and number of capivasertib bottles to be dispensed at each dispensation visit may therefore differ from those described in Table 14.

6.1.1.5 Oleclumab (MEDI9447)

Oleclumab (MEDI9447) is supplied as a [redacted] mg and [redacted] mg vial solution for infusion after dilution. The solution contains 50 mg/mL oleclumab (MEDI9447), [redacted] mM [redacted] mM [redacted] and [redacted]% (w/v) [redacted]; it has a pH of [redacted] and a density of [redacted] g/mL. The nominal fill volume is [redacted] mL.

Oleclumab is a sterile, clear to opalescent, colorless to yellow solution that may contain a few white to off-white translucent particles.

IP vials are stored at 2°C to 8°C (36°F to 46°F) and must not be frozen. Investigational product must be kept in original packaging until use to prevent prolonged light exposure.

Preparation of oleclumab (MEDI9447) doses for administration with an IV bag

Each vial selected for dose preparation should be inspected. If the solution is not clear to opalescent or if any discoloration or particulates are observed, the site monitor should be notified immediately, and the vial(s) should be stored in **quarantine** at refrigerated (2°C to 8°C [36°F to 46°F]) temperature for drug accountability and potential future inspection.

Oleclumab (MEDI9447) should not be removed from storage at 2°C to 8°C (36°F to 46°F) until all other procedures required prior to patient dosing have been completed. After removing the required number of vials CCI [redacted] the carton container should be immediately returned to refrigerated storage.

The dose of oleclumab (MEDI9447) for administration must be prepared by the Investigator or site's designated IP manager using an aseptic technique. The total time from needle puncture of the oleclumab (MEDI9447) vial to the start of administration must not exceed the following:

- 24 hours at 2°C to 8°C (36°F to 46°F)
- 4 hours at room temperature
- If the final product is stored at both refrigerated and ambient temperatures, the total time must not exceed 24 hours.

If the preparation time exceeds the time limits, a new dose must be prepared from new vials. Oleclumab (MEDI9447) does not contain preservatives, and any unused portion must be discarded.

No incompatibilities between oleclumab (MEDI9447) and CCI [redacted] or CCI [redacted] bags have been observed.

For detailed dose preparation, refer to the Handling Instructions document provided by AstraZeneca.

Administration of oleclumab (MEDI9447)

Doses of 1500 and 3000 mg will be administered using an IV bag containing 0.9% (w/v) saline, with a final oleclumab (MEDI9447) concentration ranging from CCI [redacted] mg/mL, and delivered through an IV administration set with a 0.2 or 0.22 µm filter. Add CCI [redacted] mL (ie, 1500 mg) or CCI [redacted] mL (ie, 3000 mg) of oleclumab (MEDI9447) to the IV bag. The IV bag size should be selected such that the final concentration is within 1.5 to 30 mg/mL. The bag should be mixed gently to ensure homogeneity of the dose in the bag.

There must be no less than CCI [redacted] between the end of the infusion of oleclumab (MEDI9447) and the beginning of the infusion of durvalumab. The infusion of paclitaxel should start between 15 and 30 minutes after the durvalumab infusion ends.

The standard infusion time is 1 hour; however, if there are interruptions during infusion, the total allowed time for infusion must not exceed 4 hours at room temperature. If this duration is

met, then the remainder of the dose should be abandoned and should not be completed with a second prepared dose.

Do not co-administer other drugs through the same infusion line. The IV line will be flushed according to local practices to ensure the full dose is administered. Infusion time does not include the final flush time.

The durvalumab infusion will start approximately [redacted] after the end of the infusion of oleclumab. If there are no clinically significant infusion reactions within the first cycle, and at the discretion of the Investigator, then for all other cycles, durvalumab can be given no less than [redacted] minutes after the infusion of oleclumab has finished.

Oleclumab administration at the initial dose (dose level 1) and in the event of de-escalation (dose level -1) during the safety run-in period is summarized in Table 18.

Monitoring of oleclumab (MEDI9447) dose administration

Patients will be monitored during and after infusion of oleclumab (MEDI9447) (and subsequent infusion of durvalumab). Vital signs will be measured according to the SoA (Table 1).

Management of IP-related toxicities, including those for oleclumab (MEDI9447) and durvalumab, are described in Section 8.4.6. [redacted] and/or an [redacted] may be administered at the discretion of the Investigator. If the IRR is severe or prolonged, [redacted] [redacted] (or the equivalent) should also be administered. Investigators may administer [redacted] at their discretion as clinically indicated and per their institution's guidelines. The Study Clinical Lead should be informed if [redacted] are utilized for management of an IRR.

As with any biologic product, allergic reactions to dose administration are possible. Therefore, appropriate drugs and medical equipment to treat acute anaphylactic reactions must be immediately available, and study personnel must be trained to recognize and treat anaphylaxis.

6.1.1.6 DS-8201a (trastuzumab deruxtecan)

DS-8201a (T-DXd) will be supplied by AstraZeneca as a [redacted] mg/vial lyophilized powder for concentrate for solution for infusion. Following reconstitution with sterile water for injection, the solution contains 20 mg/mL [redacted]

The reconstituted drug product is a [redacted]

Investigational product vials are stored at 2°C to 8°C (36°F to 46°F) and must not be frozen. Investigational product must be kept in original packaging until use to prevent prolonged light exposure.

Each vial is designed for single use only and is not to be used to treat more than 1 patient.

Preparation of DS-8201a doses for administration with an IV bag

The dose of DS-8201a for administration must be prepared by the Investigator's or site's designated IP manager using aseptic technique. Total time from needle puncture of the DS-8201a vial to the start of administration must not exceed:

- 24 hours at 2°C to 8°C (36°F to 46°F)
- 4 hours at room temperature
- If the final product is stored at both refrigerated and ambient temperatures, the total time must not exceed 24 hours.

Following preparation and during administration, the prepared IV bag must be covered by light protection cover.

Procedures for proper handling and disposal of anticancer drugs should be followed in compliance with the standard operating procedures of the study site.

Administration of DS-8201a

DS-8201a is emetogenic, which includes delayed nausea and/or vomiting. Prior to each dose of DS-8201a, patients should be premedicated with a combination regimen of two or three medicinal products (e.g., dexamethasone with either a 5-HT3 receptor antagonist and/or a NK1 receptor antagonist, as well as other medicinal products as indicated) for prevention of chemotherapy-induced nausea and vomiting. DS-8201a will be administered using an IV bag containing 5% (w/v) Dextrose Injection infusion solution and delivered through an IV administration set with a 0.2- or 0.22-µm filter. The standard infusion time for DS-8201a is approximately 90 minutes ±10 minutes for the first infusion. If the first infusion is well tolerated and the patient does not experience an infusion-related reaction, then the minimum infusion time for subsequent cycles is [redacted] However, if there are interruptions during the infusion, the total time must not exceed [redacted] at room temperature.

Refer to the Pharmacy Instructions for detailed information about preparation and administration of DS-8201a.

There must be no less than [redacted] between the end of the infusion of DS-8201a and the beginning of the infusion of durvalumab.

The durvalumab infusion will start approximately [redacted] after the end of the infusion of DS-8201a. If there are no clinically significant infusion reactions within the first cycle, and at the discretion of the Investigator, then for all other cycles, durvalumab can be given no less than [redacted] after the infusion of DS-8201a has finished.

DS-8201a administration at the initial dose (dose level 1) and in the event of de-escalation (dose level -1) during the safety run-in period is summarized in Table 18.

Monitoring of DS-8201a dose administration

Patients will be monitored during and after infusion of DS-8201a (and subsequent infusion of durvalumab). Vital signs will be measured according to the SoA (Table 4).

Management of IP-related toxicities, including those for DS-8201a and durvalumab, are described in Section 8.4.6.

As with any biologic product, allergic reactions to dose administration are possible. Therefore, appropriate drugs and medical equipment to treat acute anaphylactic reactions must be immediately available, and study personnel must be trained to recognize and treat anaphylaxis.

6.1.1.7 Dato-DXd (datopotamab deruxtecan; DS-1062a)

Dato-DXd will be supplied by AstraZeneca as a [redacted] mg/vial lyophilized powder for concentrate for solution for infusion. Following reconstitution with sterile water for injection, the solution contains 20 mg/mL [redacted]

The reconstituted drug product is a [redacted]

Investigational product vials are stored at 2°C to 8°C (36°F to 46°F) and must not be frozen. Investigational product must be kept in original packaging until use to prevent prolonged light exposure.

Preparation of Dato-DXd

The dose of Dato-DXd for administration must be prepared by the Investigator’s or site’s designated IP manager using aseptic technique in compliance with local regulations and site requirements. Total time from needle puncture of the Dato-DXd vial to the **start** of administration must not exceed 24 hours at 2°C to 8°C (36°F to 46°F). Following preparation and during administration, the prepared IV bag must be covered by light protection cover.

Administration of Dato-DXd

Dato-DXd will be administered as a 6.0 mg/kg IV infusion q3w on Day 1 of each 21-day cycle. Based on the currently available clinical safety data, it is highly recommended that patients receive [redacted] agents prior to infusion of Dato-DXd and on subsequent days as needed. [redacted] such as [redacted] and [redacted] should be considered and administered in accordance with the prescribing information or institutional guidelines. [redacted] can be used, if needed. Preferentially, other [redacted] agents should be used before considering the use of [redacted] Premedication for IRRS is required prior to any dose of Dato-DXd and must include [redacted] and [redacted] with and without [redacted] Premedication with [redacted] such as [redacted] mg (or equivalent) can be given per investigator discretion. Patients should remain at the site for at least [redacted] post-infusion of every dose of Dato-DXd for close observation for possible allergic reaction.

Dato-DXd will be administered using an IV bag containing [redacted] infusion solution and delivered through an IV administration set with a 0.2- or 0.22-µm filter. The standard infusion time for Dato-DXd is approximately [redacted] for the first infusion. If the first infusion is well tolerated and the patient does not experience an infusion-related reaction, then the minimum infusion time for subsequent cycles is [redacted] [redacted]. In the case of an IRR at any cycle, follow the TMGs. However, if there are interruptions during the infusion, the total cumulative time from needle puncture of the vial to the end of administration must not exceed [redacted] [redacted] at room temperature.

The IV line will be flushed with [redacted] according to local practices to ensure the full dose is administered. Infusion time does not include the final flush time.

Refer to the Pharmacy Instructions for detailed information about preparation and administration of Dato-DXd.

The participant should remain at the site for at least [redacted] post-infusion of Dato-DXd for close observation for possible allergic reactions and IRRs at all cycles.

Durvalumab should not be co-administered with other drugs through the same infusion line.

Dato-DXd will be administered first. For Cycle 1, the durvalumab infusion will start approximately [redacted] after the end of the infusion of Dato-DXd. If there are no clinically significant infusion reactions within the first cycle, and at the discretion of the Investigator, then for all other cycles, durvalumab can be given a minimum of [redacted] [redacted] after the end of the Dato-DXd infusion.

The participant's weight at screening (baseline) will be used to calculate the initial dose. If, during the course of treatment, the participant's weight changes [redacted] the participant's

dose will be recalculated based on the participant's updated weight. After the recalculation, the updated participant's weight will be used as the new baseline weight. The site may follow local institutional policy for re-calculating dose based on weight changes less than [redacted]

Dato-DXd administration at the initial dose (dose level 1) and in the event of de-escalation (dose level -1) during the safety run-in period is summarized in Table 18.

[redacted]

[redacted] will be [redacted] as specified in the SoA. An [redacted] will be provided [redacted] and [redacted] until the [redacted] which will include a [redacted]

Participants should adhere to the following guidance:

- [redacted]
- [redacted]
- [redacted]
  - [redacted]
- [redacted]
- [redacted]

[redacted]

CCI [redacted]

Monitoring of Dato-DXd dose administration

Patients will be monitored during and after infusion of Dato-DXd (and subsequent infusion of durvalumab). Vital signs will be measured according to the SoA (Table 5).

Management of IP-related toxicities, including those for Dato-DXd and durvalumab, are described in Section 8.4.6.

Management of study intervention-related toxicities are described in the Toxicity Management Guidelines (TMGs) for Dato-DXd, (see the Annex document to this CSP). As with any biologic product, allergic reactions to dose administration are possible. Therefore, appropriate drugs and medical equipment to treat acute anaphylactic reactions must be immediately available, and study personnel must be trained to recognise and treat anaphylaxis.

6.1.2 Dose and treatment regimens

The individual treatment arms for Parts 1 and 2 of the study are summarized in Table 15. Part 1 has 8 arms. Part 2 is anticipated to have 2 or 3 arms.

Crossover within the study will not be permitted. For example, if a patient is enrolled into Arm 1 in Part 1 and experiences PD, she cannot enroll into another arm of this protocol.

The durvalumab + paclitaxel treatment regimen (Arm 1) is summarized in Section 6.1.2.1. An overview of the novel treatment combination regimens (Arms 2 and 5) is provided in Section 6.1.2.2, and individual regimens for each treatment arm are described in Section 6.1.2.3 through Section 6.1.2.4. The durvalumab + DS-8201a treatment regimen (Arm 6) is summarized in Section 6.1.2.5. The durvalumab + Dato-DXd treatment regimen (Arms 7 and 8) is summarized in Section 6.1.2.6.

Table 15 Treatment arms

Study part	Arm	Study treatment
1	1	Durvalumab + paclitaxel
1	2	Durvalumab + paclitaxel + capivasertib (AZD5363)
1	3	Removed (Protocol Version 3)
1	4	Removed (Protocol Version 4)
1	5	durvalumab + paclitaxel + oleclumab
1	6	durvalumab + DS-8201a (trastuzumab deruxtecan)
1	7	durvalumab + Dato-DXd (datopotamab deruxtecan)
1	8	durvalumab + Dato-DXd (datopotamab deruxtecan) [a]
2		Treatment arms that meet pre-defined endpoints from Part 1 may be expanded in Part 2[b].

[a] Patients with PD-L1 positive status.
[b] Refer to Section 4.1.3 (Part 2 study design overview).

6.1.2.1 Durvalumab and paclitaxel combination therapy (Arm 1)

Patients in the durvalumab + paclitaxel combination therapy arm will receive 1500 mg durvalumab q4w via IV infusion and paclitaxel 90 mg/m^2 in 4-week cycles (3 weeks once weekly and 1 week off) via IV infusion until any discontinuation criteria are met (see Section 7.1), including clinical progression or radiological progression (refer to Appendix F) and Investigator determination that the patient is no longer benefiting from treatment with IP.

Treatment with paclitaxel will be concurrent with durvalumab (ie, starting on Cycle 1 Day 1). In the event that durvalumab is discontinued or temporarily held due to treatment-related toxicity, paclitaxel may still be administered as scheduled at the Investigator's discretion.

On days when both durvalumab and paclitaxel are administered, durvalumab will be administered first, followed by paclitaxel.

The standard infusion time for durvalumab is 1 hour, and the standard time for paclitaxel is 1 hour. In the event that there are interruptions during durvalumab infusion, the total allowed time must not exceed 8 hours at room temperature. If there are no clinically significant infusion reactions within the first cycle, and at the discretion of the Investigator, then for all other cycles, paclitaxel can be given immediately after the infusion of durvalumab has finished.

Note that if a patient's weight falls to 30 kg or below (≤30 kg), after initiation of study treatment, the patient should receive weight-based dosing equivalent to 20 mg/kg (Arms 1

through 5 only) of durvalumab q4w after consultation between the Investigator and Study Clinical Lead until the weight improves to >30 kg, at which point the patient should start receiving the fixed dosing of durvalumab 1500 mg q4w. Arms 6, 7, and 8 would not require weight-based dosing because the weight restriction is due to endotoxin limits per dose and is not applicable for doses <1500 mg.

The dosing schedule for the combination of durvalumab + paclitaxel is shown in Figure 3.

Figure 3 Dosing schedule for durvalumab + paclitaxel combination therapy

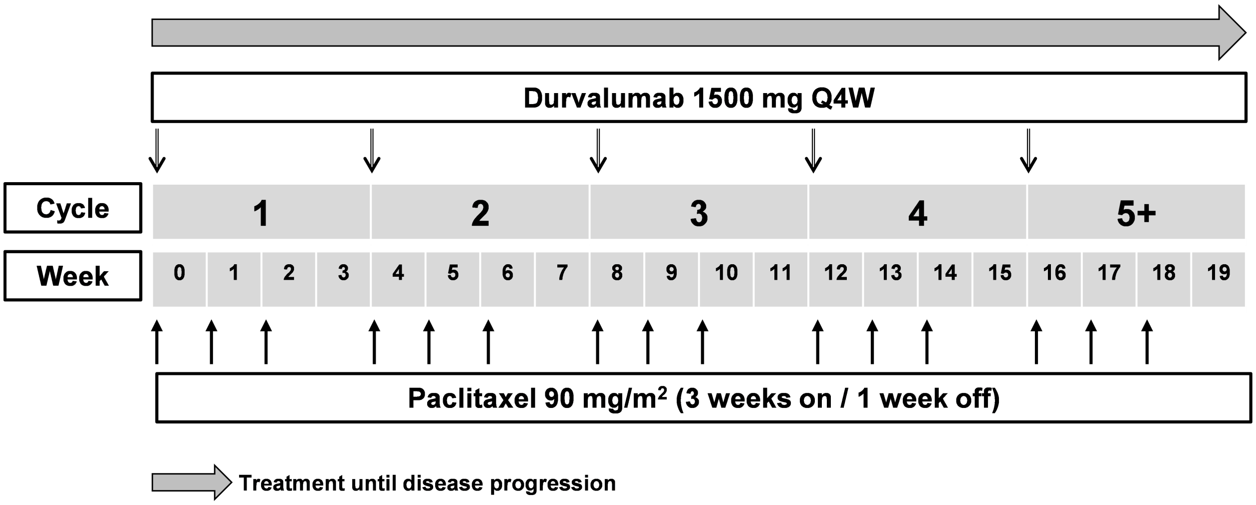

Q4W Every 4 weeks.

6.1.2.2 Durvalumab + paclitaxel + novel treatment combination arms (Arms 2 and 5): overview

Patients in each durvalumab + paclitaxel + novel treatment combination arm will receive each applicable study treatment (see Table 15) according to its dose and schedule (see Table 13) until any discontinuation criteria are met (see Section 7.1), including clinical progression or radiological progression (refer to Appendix F) and Investigator determination that the patient is no longer benefiting from treatment with IP.

Treatment with durvalumab, paclitaxel, and the novel oncology therapy will be concurrent (ie, starting on Cycle 1 Day 1). In the event that one or more of the study treatments is discontinued, refer to Section 7.1.1 for guidance on discontinuation of the other treatment(s). In the event that durvalumab is temporarily held due to treatment-related toxicity, the novel oncology therapy must also be held, with the exception of capivasertib, which may be continued in the absence of durvalumab (based on data from the PAKT study) while adhering to instructions indicated in Section 6.1.1.4; paclitaxel may still be administered as scheduled at the Investigator's discretion. In each arm, in the event that the novel oncology therapy is held due to a toxicity attributed to that agent only, durvalumab and/or paclitaxel may still be

administered as scheduled at the Investigator's discretion. In the event that paclitaxel is discontinued, durvalumab and/or the novel oncology therapy can be continued as scheduled at the Investigator's discretion.

On days when multiple IPs are administered, CCI [redacted]

Each novel treatment combination arm will undergo a safety run-in phase to confirm the RP2D before treatment of additional patients in the expansion phase (see Section 4.1.4 for details).

6.1.2.3 Durvalumab + paclitaxel + capivasertib combination therapy arm (Arm 2)

The dosing schedule for the combination of durvalumab + paclitaxel + capivasertib is shown in Figure 4.

Figure 4 Dosing schedule for durvalumab + paclitaxel + capivasertib combination therapy

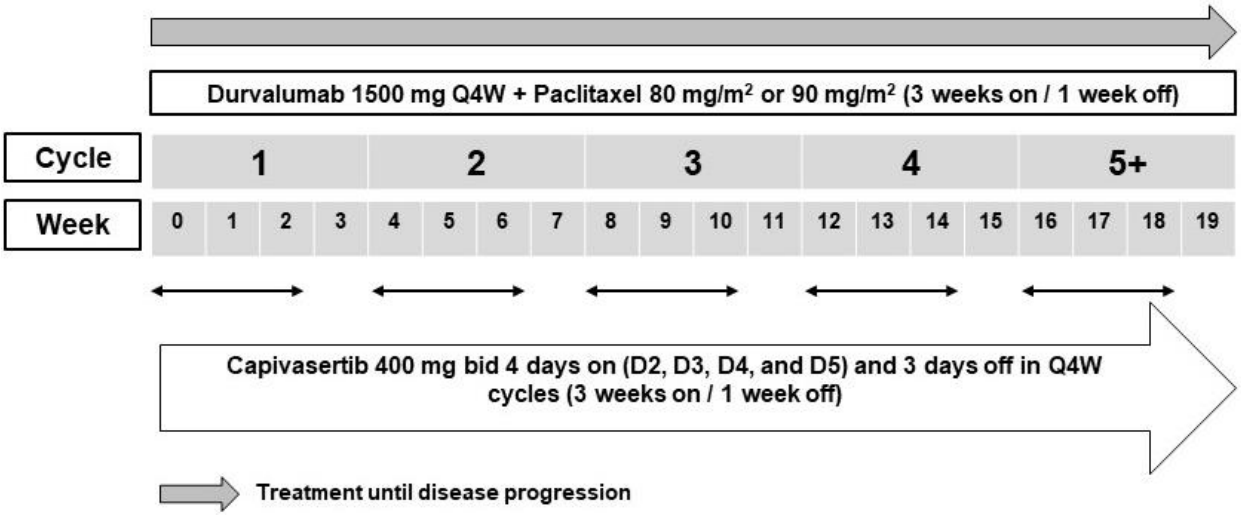

Note: Paclitaxel dose 80 mg/m^2 will be administered. For patients on Arm 2 who were treated prior to Protocol Version 4, dosing may continue unchanged (ie, if the patient was treated at 90 mg/m^2, they may continue at this dose, as clinically indicated).

bid Twice daily; Q4W Every 4 weeks.

6.1.2.4 Durvalumab + paclitaxel + oleclumab combination therapy arm (Arm 5)

The dosing schedule for the combination of durvalumab + paclitaxel + oleclumab is shown in Figure 5.

Figure 5 Dosing schedule for durvalumab + paclitaxel + oleclumab combination therapy

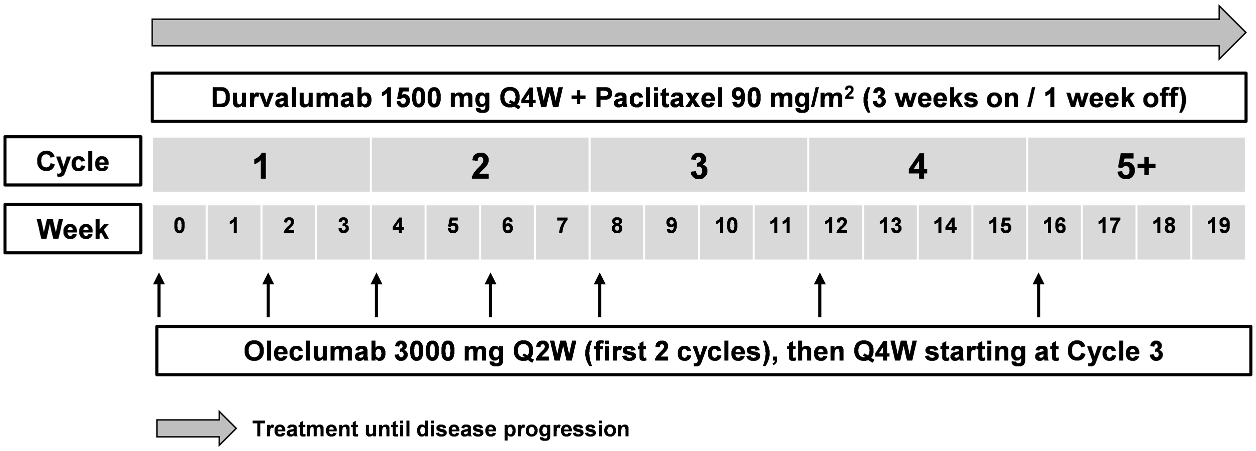

Q2W Every 2 weeks; Q4W Every 4 weeks.

6.1.2.5 Durvalumab + DS-8201a (trastuzumab deruxtecan) combination therapy arm (Arm 6)

The dosing schedule for the combination of durvalumab + DS-8201a is shown in Figure 6.

Figure 6 Dosing schedule for durvalumab + DS-8201a (trastuzumab deruxtecan) combination therapy

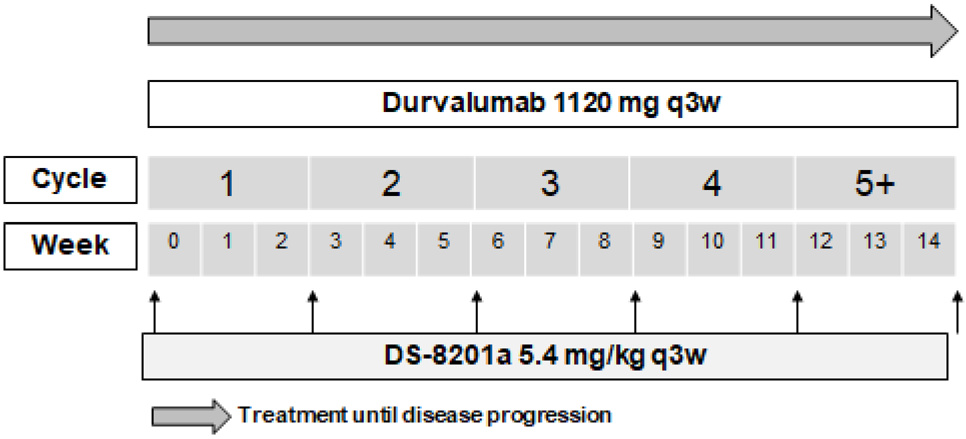

6.1.2.6 Durvalumab + Dato-DXd combination therapy arm (Arms 7 and 8)

The dosing schedule for the combination of durvalumab + Dato-DXd is shown in Figure 7.

Figure 7 Dosing schedule for durvalumab + Dato-DXd (datopotamab deruxtecan; DS-1062a) combination therapy

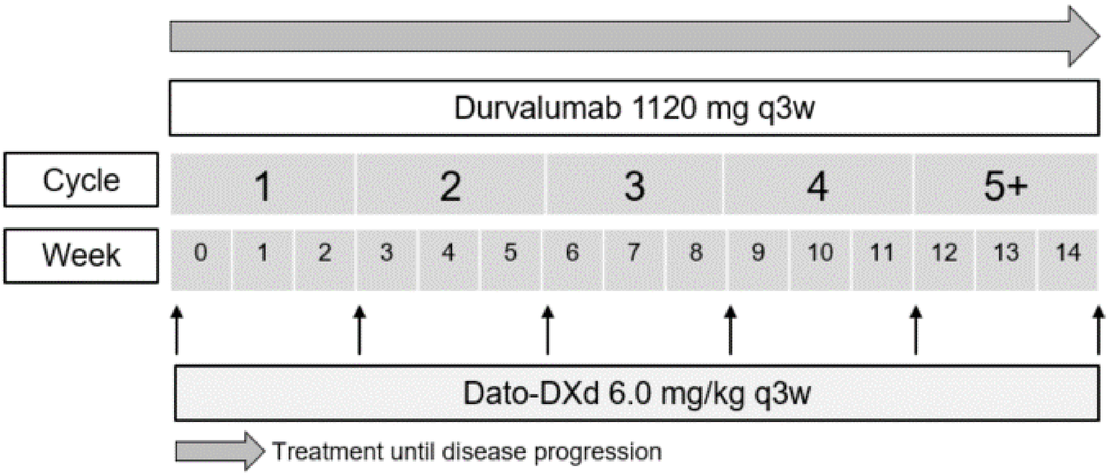

6.1.3 Duration of treatment and criteria for treatment through progression

All treatment will be administered beginning on Day 1 until any discontinuation criteria are met (see Section 7.1), including clinical progression or radiological progression (refer to Appendix F) and Investigator determination that the patient is no longer benefiting from treatment with IP.

However, patients who are clinically stable at an initial RECIST 1.1-defined PD may continue to receive study treatment at the discretion of the Investigator and patient. A follow-up scan is to be collected after the initial RECIST 1.1-defined PD no earlier than 4 weeks after and no later than the next regularly scheduled imaging visit after the prior RECIST 1.1-defined PD, and this follow-up scan is evaluated using the criteria for evaluation of scans collected after a RECIST 1.1-defined PD outlined in Appendix F. Patients who continue to receive study treatment at the discretion of the Investigator and patient[redacted] can receive treatment until they are no longer having clinical benefit, and tumor assessments should continue on their regular imaging schedule for the duration of treatment.

Patients with [redacted] or with [redacted] will not be eligible for continuing study treatment.

For all patients who are treated through progression, the Investigator should ensure the following:

- The patient does not have CCI [redacted]
- There is absence CCI [redacted]
- There is absence CCI [redacted]
- The patient still fulfills the eligibility criteria for this study (see Sections 5.1 and 5.2), with the exception CCI [redacted] CCI [redacted]
- The ICF addendum for treatment beyond progression is signed.

Patients who CCI [redacted] determine may not continue treatment after RECIST 1.1-defined PD will be followed up for survival. Patients who have discontinued treatment due to toxicity, symptomatic deterioration, or clinical progression during treatment, or who have commenced subsequent anticancer therapy, will be followed up with tumor assessments until RECIST 1.1-defined PD plus an additional optional follow-up scan or until death (whichever comes first) and followed for survival. These patients are not eligible for re-treatment at any time.

6.1.4 Storage

The Investigator, or an approved representative (eg, pharmacist), will ensure that all IP is stored in a secured area, at appropriate temperatures (refrigerated [2°C to 8°C] for IV-administered IP and as specified on the bottle label for orally administered IP [ie, capivasertib]) and in accordance with applicable regulatory requirements. A temperature log will be used to record the temperature of the storage area. Temperature excursions outside the permissible range listed in the clinical supply packaging are to be reported to the Study Clinical Lead upon detection. A calibrated temperature monitoring device will be used to record the temperature conditions in the drug storage facility. Storage conditions stated in the Investigator's Brochure may be superseded by the label storage.

Capivasertib tablets should be stored at room temperature, in their original packaging until use.

6.2 Measures to minimize bias: randomization and blinding

6.2.1 Patient enrollment and randomization

All patients will be centrally assigned to study treatment using a Randomisation and Trial Supply Management System (RTSM) (Interactive Response Technology; IRT) that will assign

them depending on open cohorts and safety run-in. Before the study is initiated, the telephone number and call-in directions and/or the log-in information and directions for the RTSM (IRT) will be provided to each site.

If a patient withdraws from the study, then his/her enrollment/randomization code cannot be reused. Withdrawn patients will not be replaced.

Investigators should keep a record (ie, the patient screening log) of patients who entered pre-screening and/or screening.

If a patient enters pre-screening, the Investigators or suitably trained delegate will do the following:

- Obtain signed pre-screening informed consent before any study-specific procedures are performed.
- Obtain a unique [redacted] enrollment number (E-code), through the RTSM (IRT) in the following format: [redacted] This number is the patient's unique identifier and is used to identify the patient on the eCRFs.

At screening/baseline (Days -28 to -1), the Investigators or suitably trained delegate will do the following:

- Obtain signed main informed consent before any study-specific screening procedures are performed. If laboratory or imaging procedures were performed for alternate reasons prior to signing consent, these can be used for screening purposes with consent of the patient. However, all screening laboratory and imaging results must have been obtained within 28 days of treatment assignment/randomization. For patients with a single TL, if screening biopsy is collected prior to screening imaging for baseline tumor assessment, allow approximately 2 weeks before imaging scans are acquired.
- If the patient did not enter pre-screening, obtain a unique [redacted] enrollment number (E-code), through the RTSM (IRT) in the following format: [redacted] This number is the patient's unique identifier and is used to identify the patient on the eCRFs.
- Determine patient eligibility (see Sections 5.1 and 5.2).
- Obtain signed informed consent for [redacted]

Procedures after a patient is confirmed to be eligible are described in Sections 6.2.1.1.

If the patient is ineligible and not assigned treatment, the RTSM (IRT) should be contacted to terminate the patient in the system.

Patients will begin treatment on Day 1. Treatment should start no more than 3 days after treatment assignment. Patients must not be assigned to treatment and must not be treated, unless all eligibility criteria have been met.

6.2.1.1 Enrollment procedures

At baseline, once the patient is confirmed to be eligible, the Investigator or suitably trained delegate will do the following:

- Obtain a unique treatment assignment number via the RTSM (IRT). The system will assign at least the first 20 eligible patients to the durvalumab + paclitaxel arm to be monitored for toxicity.
- Once recruitment is complete for the durvalumab + paclitaxel arm (Arm 1), patient assignment to the other novel treatment combination cohorts can begin. The system will PPD assign, whenever appropriate, the eligible patient to 1 of the open novel treatment combination groups. Patients with no HER2 tumor expression will be eligible for assignment to any open treatment arm other than Arm 6.
- Each subsequent novel treatment combination cohort (Arms 2 through 7) will have a 6-patient safety run-in (more or fewer patients may be enrolled depending on how many patients are evaluable for DLTs and how many patients have DLTs). The novel treatment combination arms meeting the safety evaluation criteria (ie, no more than 1 of 6 DLT-evaluable patients experience DLTs during the first cycle of dosing) will be expanded to enroll 30 patients (additional patients may be enrolled in order to have 30 evaluable patients).
- If at least 17 out of 30 patients in a given treatment arm achieve response according to the [redacted] design, then the treatment arm may continue into Part 2 and enroll 27 additional patients in order to have 57 response-evaluable patients per treatment arm for Part 2.
- For enrollment onto Arm 6 (durvalumab + DS-8201a), patients must provide pathologically documented advanced/unresectable or metastatic breast cancer with HER2 low expression (see Section 5.1 for detailed description).
- For enrollment onto Arm 8 (durvalumab + Dato-DXd), patients must have PD-L1 positive TNBC (see Section 5.1 for detailed description).

6.2.2 Procedures for handling incorrectly enrolled or randomized patients

Patients who fail to meet the eligibility criteria should not, under any circumstances, be enrolled or receive study medication. There can be no exceptions to this rule. Patients who are enrolled but are subsequently found to not meet all the eligibility criteria must not be assigned to treatment or initiated on treatment, and they must be withdrawn from the study.

When a patient does not meet all the eligibility criteria but is assigned to treatment, or incorrectly started on treatment, the Investigator should inform the AstraZeneca Study Clinical Lead immediately, and a discussion should occur between the AstraZeneca Study

Clinical Lead and the Investigator regarding whether to continue or discontinue the patient from treatment. The AstraZeneca Study Clinical Lead must ensure all decisions are appropriately documented and that the potential benefit:risk profile remains positive for the patient.

6.2.3 Methods for assigning treatment groups

The actual treatment given to patients will be determined by the randomization scheme in the RTSM (IRT) (whenever appropriate). As not all cohorts will be open at a given time, patients can only be randomized to open cohorts. If only 1 cohort is open, treatment assignment can still occur. However, the intention is to randomize patients whenever appropriate. Patients assigned to Arms 6, 7, and 8 will need to meet the additional specific inclusion and exclusion criteria for Arms 6, 7, and 8, respectively.

The randomization scheme will be produced by a computer software program that incorporates a standard procedure for generating randomization numbers.

Patients will be identified to the RTSM (IRT) per country regulations. Treatment allocation codes will be assigned as patients become eligible for treatment assignment. Drug supply after allocation is completed will be managed manually by Site Pharmacists.

6.2.4 Methods for ensuring blinding

This study is open-label and there is no blinding.

6.3 Treatment compliance

The administration of all IPs should be recorded in the appropriate sections of the eCRF.

Any change from the dosing schedule, does interruptions, dose reductions, and dose discontinuations should be recorded in the eCRF. The reason should also be documented. Note: Dose reductions are **not** permitted for durvalumab and oleclumab.

The Investigational Product Storage Manager is responsible for managing the IP from receipt by the study site until the destruction or return of all unused IP. The Investigator is responsible for ensuring that the patient has returned all unused IP.

Treatment compliance will be ensured by site reconciliation of study treatment dispensed as described in Sections 6.3.1 and 6.3.2.

Use of study treatment in doses in excess of that specified in the protocol is considered to be an overdose. Refer to Section 8.4.4 for procedures in case of overdose.

6.3.1 Treatment compliance for IV study drugs

The Investigator or pharmacist must retain records of all study drugs administered at the site (ie, those administered IV). The Study Clinical Lead will check these records to confirm the compliance with the protocol administration schedule.

6.3.2 Treatment compliance for capivasertib

Patients will be instructed to bring capivasertib [redacted] to the clinic for each study visit. Compliance will be checked by the site staff during each study visit. In case of low compliance, patients should be retrained by the site staff, and the retraining should be documented.

Patients will be instructed to bring all [redacted] to the clinic on dispensation visits. For each cycle, patients should return all unused tablets during the dispensation visit of the subsequent cycle, at which point a new set of bottles will be dispensed to the patients.

Accountability records should be maintained by the site staff and should be current and up to date.

6.4 Concomitant therapy

The Investigator must be informed as soon as possible about any medication taken from the time of screening until the end of the clinical treatment phase of the study including the [redacted] follow-up period following the last dose of study drug.

Any medication or vaccine, including over-the-counter or prescription medicines, vitamins, and/or herbal supplements, that the patient is receiving at the time of enrollment or receives during the study must be recorded along with the following:

- Reason for use
- Dates of administration, including start and end dates
- Dosage information, including dose, unit, and frequency

Patients must be instructed not to take any medications, including over-the-counter products, without first consulting with the Investigator.

Concomitant medications administered as treatment for drug-related AESIs should be recorded until either event resolution, end of study, trial termination, withdrawal of consent, or participant death.

Restricted, prohibited, and permitted concomitant medications are described in the following tables and apply to all treatment arms. Refer also to the dosing modification and toxicity

management guidelines (see Section 8.4.6). Refer to the local prescribing information for paclitaxel with regard to warnings, precautions, and contraindications.

Table 16 Prohibited concomitant medications and foods

**Prohibited medication/class of drug:**	**Usage:**
For all treatment arms	
Any investigational anticancer therapy other than those under investigation in this study	Should not be given concomitantly while the patient is on study treatment
mAbs against CTLA-4, PD-1, or PD-L1 other than those under investigation in this study	Should not be given concomitantly while the patient is on study treatment
Any concurrent chemotherapy, radiotherapy, immunotherapy, or biologic or hormonal therapy for cancer treatment other than those under investigation in this study	Should not be given concomitantly while the patient is on study treatment. (Concurrent use of hormones for non-cancer-related conditions [eg, insulin for diabetes and hormone replacement therapy] is acceptable. Local treatment of isolated lesions, excluding target lesions, for palliative intent is acceptable [eg, by local surgery or radiotherapy]).<br>For Arms 6, 7, and 8, radiotherapy to the thorax should be avoided and all radiotherapy should be reviewed with the study team.
Live attenuated vaccines	Should not be given through [redacted] after the last dose of IP
Immunosuppressive medications, including, but not limited to, systemic corticosteroids at doses exceeding [redacted] mg/day of prednisone or equivalent, methotrexate, azathioprine, and tumor necrosis factor-α blockers	Should not be given concomitantly or used for [redacted] prior to any IP infusions. The following are allowed exceptions:<br>• Use of immunosuppressive medications for the management of IP-related AEs<br>• Use as premedication<br>• Use in patients with contrast allergies<br>• Use of inhaled, topical, intranasal corticosteroids, intra-articular steroid injections, and [redacted]<br>A temporary period of steroids will be allowed if clinically indicated and considered to be essential for the management of non-IP related events experienced by the patient (eg, chronic obstructive pulmonary disease, radiation, nausea, etc).

**Prohibited medication/class of drug:**	**Usage:**
EGFR TKIs	Should not be given concomitantly.<br>Should be used with caution in the [redacted] post last dose of IP<br>Increased incidences of pneumonitis (with third generation EGFR TKIs) and increased incidence of transaminase increases (with 1st generation EGFR TKIs) has been reported when durvalumab has been given concomitantly.
Herbal and natural remedies which may have immune-modulating effects	Should not be given concomitantly unless agreed by the Sponsor
Medications or foods (eg, grapefruit juice) that may affect IP metabolism (eg, CYP1A2, CYP2C19, CYP2D6, or CYP3A4 inhibitors/inducers)	Should not change medications or add medications or foods that may affect IP metabolism (eg, CYP1A2, CYP2C19, CYP2D6, or CYP3A4 inhibitors/ inducers), unless considered clinically indicated[a]
**For [redacted] arm only**	
Medications that may be QT prolonging	The concomitant administration of drugs known to prolong the QT interval is restricted unless considered essential due to patient management, in which case, patients should be closely monitored. Information regarding drugs known to prolong the QT interval can be found in Appendix I. Standard anti-emetic therapy, including a 5-HT3-antagonist, can be given, as needed, on a prophylactic and treatment basis in compliance with the standards of the center's local policy.
Drugs or herbal supplements that are known to be potent inhibitors or inducers of CYP3A4, CYP2C9 and/or CYP2D6 metabolism inhibition and/or MATE1 or OCT2 transport, and which have a narrow therapeutic window, as they may increase the exposure and toxicity of capivasertib.	Should not be given concomitantly while the patient is on study treatment[a]
Drugs known to be sensitive to CYP3A4, CYP2C9 and/or CYP2D6 metabolism inhibition and/or have a narrow therapeutic window. If co-administration is necessary, then additional monitoring for signs of toxicity related to increased exposure to the substrates is required.	Should not be given concomitantly while the patient is on study treatment[a]
**For Arms [redacted] only**	
[redacted]	Should not be given concomitantly while the patient is on study treatment. See Appendix O

[a] Additional details on drug-drug interactions are provided in Appendix K.

5-HT3 5-hydroxytryptamine receptor; AE Adverse event; CYP cytochrome P450; EGFR Epidermal growth factor receptor; IP Investigational product; mAb Monoclonal antibody; PD-1 Programmed cell death 1; PD-L1 Programmed cell death ligand 1; TKI tyrosine kinase inhibitors.

Table 17 Supportive medications

Supportive medication/class of drug:	Usage:
Concomitant medications or treatments [redacted] deemed necessary to provide adequate prophylactic or supportive care, except for those medications identified as "prohibited," as listed in Table 16	To be administered as prescribed by the Investigator
Best supportive care (including antibiotics, nutritional support, correction of metabolic disorders, optimal symptom control, and pain management [including palliative radiotherapy to non-target lesions, etc])	Should be used, when necessary, for all patients
Inactivated viruses, such as those in the influenza vaccine	Permitted
G-CSF agents	Primary prophylaxis with neutrophil growth factors is prohibited. Patients experiencing neutropenic fever or severe neutropenia with paclitaxel monotherapy during neoadjuvant or adjuvant therapy may receive G-CSF at the discretion of the Investigator. Patients who develop neutropenia during the study may be administered growth factors according to local standards of care.

INR International normalized ratio; G-CSF Granulocyte colony-stimulation factor.

6.4.1 Other concomitant treatment

Medication other than those described above that is considered necessary for the patient's safety and wellbeing may be given at the discretion of the Investigator and recorded in the appropriate sections of the eCRF.

For Arms 7, and 8 only:

- Subjects/participants should be closely monitored when Dato-DXd is concomitantly used with drugs that inhibit CYP3A, organic anion transporting polypeptide (OATP) 1B1, OATP1B3, multidrug and toxin extrusion transporter (MATE) 2-K, P-glycoprotein, breast cancer resistance protein (BCRP), and multidrug resistance-associated protein (MRP) 1. For a list of inhibitor drugs, refer to the US Food and Drug Administration Table of Substrates, Inhibitors and Inducers (https://www.fda.gov/drugs/drug-interactions-labeling/drug-development-and-drug-interactions-table-substrates-inhibitors-and-inducers) or locally available sources.
- In patients with [redacted] with undetectable [redacted] ust be initiated, then continued for the study duration and for [redacted] months after the last dose of IP.

6.4.2 Ancillary treatment for management of immuno-oncology related toxicities

The study site will supply the required rescue medication and will be obtained locally. The following rescue medications are required to be available at the site:

1 Infliximab/infliximab biosimilar (eg, for colitis).

2 Mycophenolate (eg, for hepatitis).

Under certain circumstances when local sourcing by the study site is not feasible or local regulations prevent the use of infliximab or mycophenolate for this use (as they are considered off-label for management of immunotherapy-related toxicities), AstraZeneca will centrally supply the required rescue medications, which will be labelled and accompanied by Prescribing Information with local language translated text in accordance with regulatory guidelines. Accountability and storage requirements, as specified in Section 6.1, apply for any study intervention supplied by AstraZeneca. If required to manage an imAE, then the IVRS/IWRS RTSM (IRT) will allocate the specific medication either by a kit or another identification number, to the pharmacist for a specific participant at the time of the event.

As a result of imAEs that could potentially be experienced by participants on durvalumab, appropriate treatment (eg, steroids and specific immunosuppressant rescue medications) must be made readily available to this participant population.

6.5 Dose modification

Dose modifications at the cohort level during the Part 1 safety run-in period are described in Section 6.5.1. Dose modifications in individual patients applicable to all parts of the study are described in Section 6.5.2 through Section 6.5.2.6. See also Section 8.4.6 on toxicity management.

6.5.1 Dose modifications at the cohort level during the safety run-in period of Part 1

Part 1 of the study will have a safety run-in period for each novel treatment combination arm (Arms 2 through 7). In the event that the initial dose level of an oncology therapy is not tolerated (ie, [redacted] or more of up to 6 DLT-evaluable patients experience DLTs at the combination dose level), a new cohort with up to 6 new DLT-evaluable patients will be opened at a lower dose level (see Table 18); this process may be repeated until the lowest dose level is reached. Any decision to introduce additional cohorts in order to investigate an alternative dosing regimen will be at the discretion of AstraZeneca after discussion with the SRC. In the event that the lowest dose is not tolerated, no additional patients will be enrolled to evaluate that novel treatment combination arm.

Any dose reductions due to DLTs will be evaluated in new cohorts of new patients; patients may not switch from 1 cohort to another. A patient who develops a toxicity during the DLT period would follow the appropriate dose level modifications and toxicity management guidelines as described in Section 8.4.6. Additional details on the safety run-in period and combination dose finding approach are provided in Section 4.1.4.

Table 18 Dose level modification in cohorts with dose reduction during the safety run-in period

	Dose level		
**Study agent**	**Initial dose (level 1)**	**Dose level -1**	**Dose level -2**
Capivasertib	400 mg bid oral 4 days on (D2, D3, D4, and D5) and 3 days off in 4-week cycles: 3 weeks on (intermittent; see above) and 1 week off	CCI CCI [redacted]	CCI [redacted]
Oleclumab	3000 mg IV q2w first 2 cycles, then q4w starting at C3 (D1)	CCI [redacted]	CCI [redacted]
DS-8201a (trastuzumab deruxtecan)	5.4 mg/kg IV q3w	CCI [redacted]	CCI [redacted]
Dato-DXd (datopotamab deruxtecan)	6.0 mg/kg IV q3w	CCI [redacted]	CCI [redacted]

bid Twice daily; C Cycle; D day; IV Intravenous; q1w Every 1 week; q2w Every 2 weeks; q3w Every 3 weeks; q4w Every 4 weeks.

6.5.2 Individual patient dose modifications during all study parts

6.5.2.1 Durvalumab

Dose delays are permitted for durvalumab therapy (see dosing modification and toxicity management guidelines in Section 8.4.6). However, **dose reduction is not permitted**.

6.5.2.2 Paclitaxel

Investigators should follow their local guidelines and standard clinical practice regarding management of paclitaxel-related toxicities.

Prophylactic anti-emetic therapy prior to and after paclitaxel treatment should be given as needed on a prophylactic and treatment basis in compliance with institutional standards. Granulocyte colony stimulating factors in compliance with the standards of the center should be given as needed for secondary prophylaxis or treatment of neutropenia (see also Table 17).

6.5.2.3 Capivasertib

The combination of durvalumab, paclitaxel, and capivasertib may increase the risk or severity of overlapping toxicities. There could potentially be new, unanticipated toxicities as well. The Investigator should use clinical judgement when attributing a toxicity to 1 or more of the agents. Paclitaxel dose reductions, delays, and toxicity management guidelines are found in Section 6.5.2.2. Durvalumab delays and toxicity management guidelines are found in Section 6.5.2.1. Capivasertib dose reductions, delays, and toxicity management guidelines are found below.

The following dose modifications apply to toxicities that are attributable to capivasertib. Investigator judgement should apply for the management of toxicities possibly related to more than 1 investigational medicinal product (IMP). (Durvalumab toxicity guidelines can be found in Section 6.5.2.1 and Section 8.4.6.)

If a patient experiences a clinically significant and/or unacceptable toxicity (including a DLT related to capivasertib [ie, not attributable to the disease or disease-related processes under investigation] during the assessment period), capivasertib dosing will be interrupted or the dose reduced and supportive therapy administered as required (see Appendix I).

Table 19 Dose level reductions for individual patients receiving capivasertib

	Dose level		
**Study agent**	**Initial dose (level 1)**	**Dose level -1**	**Dose level -2**
Capivasertib	400 mg bid oral<br>4 days on (D2, D3, D4, and D5) and<br>3 days off in 4-week cycles: 3 weeks on (intermittent; see above) and 1 week off	CCI [redacted]	CCI [redacted]

bid Twice daily; C Cycle; D day.

Dose modification guidelines for general capivasertib-related toxicities are shown in Table 20. Refer to Section 8.4.6.3 and Appendix I for the management of capivasertib specific AEs: rash, hyperglycemia, and diarrhea. Appropriate and optimal treatment of the toxicity is assumed prior to considering dose modifications. The Study Clinical Lead may be consulted prior to discontinuation of study drug due to toxicities.

Table 20 Dose modifications for general capivasertib-related toxicities

NCI CTCAE v4.0 Toxicity Grade	Action
**Grade 1 or 2 clinically significant or intolerable**	Hold dosing and follow guidance below, depending on outcome
• Resolves to baseline or clinically tolerable within 21 days of onset	• Resume dosing at same dose or one reduced dose level as clinically appropriate
• Grade 1 or 2 clinically significant or intolerable does not resolved within 21 days of onset	• Discontinue study drug and observe patient until resolution
**Grade ≥3**	Hold dosing and follow guidance below, depending on outcome
• Grade ≥3 toxicity for ≤21 days and resolves to ≤Grade 2 or baseline within 21 days of onset	• Resume dosing at same dose or one reduced dose level as clinically appropriate
• Grade ≥3 toxicity for >21 days	• Discontinue study drug and observe patient until resolution

NCI CTCAE v4 National Cancer Institute Common Terminology Criteria for Adverse Events Version 4.0.

6.5.2.4 Oleclumab

The combination of durvalumab, paclitaxel, and oleclumab may increase the risk or severity of overlapping toxicities. There could potentially be new, unanticipated toxicities as well. The investigator should use clinical judgement when attributing a toxicity to 1 or more of the agents. Paclitaxel dose reductions, delays, and toxicity management guidelines are found in Section 6.5.2. Durvalumab delays and toxicity management guidelines are found in Section 6.5.2.1. Oleclumab dose reductions, delays, and toxicity management guidelines are found below.

The following dose modifications apply to toxicities that are attributable to oleclumab.

Dose delays are permitted for oleclumab therapy. However, **dose reduction is not permitted**.

Specifically, dose delay is permitted for management of non-drug-related AEs up to 7 days after the scheduled dose with prior consultation of the Study Clinical Lead. In these instances, the dosing interval for the next oleclumab cycle may be shortened as clinically feasible to gradually align with the schedule of tumor efficacy assessment and durvalumab dosing. Two consecutive doses must be administered at least 10 days apart. Any dose delayed longer than 7 days should be skipped, and the patient should receive the next scheduled dose.

For management of dose delays due to drug-related events, the toxicity management guidelines (see Section 8.4.6.4) or DLT guidelines (see Section 4.1.4) should be followed, as applicable.

In summary, if a patient experiences a clinically significant and/or unacceptable toxicity (including a DLT related to oleclumab during the assessment period), dosing will be interrupted (or discontinued in the case of a DLT) or supportive therapy administered as required.

On improvement of an AE for which oleclumab was temporarily interrupted, oleclumab may be restarted at the same dose at the discretion of the Investigator. If a further episode of the same AE subsequently requires dose interruption, or if a different AE subsequently requires dose interruption, oleclumab may be restarted at the same dose level on improvement of the AE.

6.5.2.5 DS-8201a (trastuzumab deruxtecan)

The combination of durvalumab and DS-8201a may increase the risk or severity of potential overlapping toxicities. There could potentially be new, unanticipated toxicities as well. The Investigator should use clinical judgement when attributing a toxicity to 1 or more of the agents. Durvalumab delays and toxicity management guidelines are found in Section 6.5.2.1. DS-8201a dose reductions, delays, and toxicity management guidelines are found below and in Appendix L. In the event that durvalumab is temporarily held due to treatment-related toxicity, attributed to durvalumab only, DS-8201a can continue. In the event that DS-8201a is temporarily held due to treatment-related toxicity attributed to DS-8201a only, durvalumab can continue.

All dose modifications (interruption, reduction, and/or discontinuation) should be based on the worst preceding toxicity (CTCAE version 4.03). Specific criteria for interruption, re-initiation, dose reduction, and/or discontinuation of DS-8201a are listed in Table 21 which is applicable only to TEAEs that are assessed as related to use of DS-8201a by the Investigator(s). For non-drug related TEAEs, follow standard clinical practice. Appropriate clinical experts should be consulted as deemed necessary and following the toxicity management guidelines.

Every effort should be made to limit study drug delay, however in circumstances of adverse event management or medical intervention, the study drug can be held up to [redacted] from the last DS-8201a dose. During this time scheduled CT/MRI scans should continue as per protocol, and patients should fulfil **all of** the following criteria:

- Study drug may be resumed with confirmation of continued benefit per RECIST 1.1. Scans should be performed at the frequency defined per protocol, while the drug is being held- At minimum 1 scan must be done within 6 weeks prior to restarting the study drug

- IP(s) is/are restarted within the guidance of the TMGs for DS-8201a and any combination agents, if appropriate
- No prohibited concomitant medications have been administered since the last dose of DS-8201a

For management of dose delays due to drug-related events, the toxicity management guidelines (see Section 8.4.6.4) or DLT guidelines (see Section 4.1.4) should be followed, as applicable.

In summary, if a patient experiences a clinically significant and/or unacceptable toxicity (including a DLT related to DS-8201a during the assessment period), dosing will be interrupted (or discontinued in the case of a DLT) or supportive therapy administered as required.

On improvement of an AE for which DS-8201a was temporarily interrupted, DS-8201a may be restarted at the same dose at the discretion of the Investigator. If a further episode of the same AE subsequently requires dose interruption, or if a different AE subsequently requires dose interruption, DS-8201a may be restarted at the same dose level on improvement of the AE (Appendix L).

Patients for whom DS-8201a dosing is temporarily withheld for any reason may receive future cycles scheduled based on the date of the last DS-8201a dose. The minimal interval between two consecutive doses is 19 days.

Table 21 Dose level modification for individual patients receiving DS-8201a

	Dose level		
**Study agent**	**Initial dose (level 1)**	**Dose level -1**	**Dose level -2**
DS-8201a (trastuzumab deruxtecan)	5.4 mg/kg IV q3w	[redacted] mg/kg IV q3w	[redacted] mg/kg IV q3w

IV Intravenous; q3w Every 3 weeks.

Once the dose of DS-8201a has been reduced because of toxicity, all subsequent cycles should be administered at that lower dose level unless further dose reduction is required. More than 2 dose reductions are not allowed, and the patient will be withdrawn from the study treatment if further toxicity meeting the requirement for dose reduction occurs.

6.5.2.6 Dato-DXd (datopotamab deruxtecan; DS-1062a)

The combination of durvalumab and Dato-DXd may increase the risk or severity of potential overlapping toxicities. There could potentially be new, unanticipated toxicities as well. The Investigator should use clinical judgement when attributing a toxicity to one or more of the

agents. Durvalumab delays and toxicity management guidelines are found in Section 6.5.2.1. Dato-DXd delays are found below and toxicity management guidelines are included in the Annex document to this CSP. In the event that durvalumab is temporarily held due to treatment-related toxicity, attributed to durvalumab only, Dato-DXd can continue. In the event that Dato-DXd is temporarily held due to treatment-related toxicity attributed to Dato-Dxd only, durvalumab can continue.

All dose modifications (interruption, reduction, and/or discontinuation) should be based on the worst preceding toxicity as per Dato-DXd TMG Annex document to this CSP.

Specific criteria for interruption, re-initiation, dose reduction, and/or discontinuation of Dato-DXd are listed in Table 22 that is applicable only to TEAEs that are assessed as related to use of Dato-DXd a by the Investigator(s). For [redacted], follow standard clinical practice. Appropriate clinical experts should be consulted as deemed necessary and following the toxicity management guidelines.

Dose delay guidelines:

A Dato-DXd dose delay due to treatment related toxicity can be delayed for up to [redacted] cycles [redacted] from the planned date of administration (ie, [redacted] from the last infusion date) (see Figure 8). If a patient is assessed as requiring a dose delay of longer than [redacted] cycles (up to [redacted] from the last infusion date to the planned date of administration on a Q3W schedule), the participant must discontinue study treatment.

Figure 8 Dato-DXd Dose Delay Guidelines

[redacted]

Dato-DXd dose delays for conditions [redacted] should be kept as short as possible. If a patient cannot restart Dato-DXd treatment within [redacted] for resolution of intercurrent conditions [redacted], the case should be discussed with the AstraZeneca study physician. All dose reductions and delay (including any missed doses), and the reasons for the reductions/delays are to be recorded in the eCRF.

The dosing interval for the next Dato-DXd cycle may be shortened, as clinically feasible to gradually align with the schedule of tumor efficacy assessment. Dosing of Dato-DXd should be harmonized with durvalumab dosing upon recovery from treatment-related toxicity attributed to Dato-DXd only. Two consecutive doses must be administered at least [redacted] apart.

Dose reduction guidelines:

Up to [redacted] dose reductions will be permitted for patients receiving Dato-DXd (Table 22). Once the dose of Dato-DXd is reduced, [redacted] will be permitted. After the permitted dose reductions, if further toxicity meeting the requirement for dose reduction occurs, the patient will be withdrawn from the study treatment. More than [redacted] dose reductions are not allowed, and the study intervention will be discontinued if further toxicity that meets the requirement for dose reduction occurs.

Table 22 [redacted] modification for individual patients receiving Dato-DXd

		[redacted]	
**Study agent**	**Initial dose (level 1)**	[redacted]	[redacted]
Dato-DXd (datopotamab deruxtecan)	6.0 mg/kg IV q3w	[redacted] mg/kg IV q3w	[redacted] mg/kg IV q3w

IV Intravenous; q3w Every 3 weeks.

Dose interruption and modification – toxicity management guidelines:

Dose modification criteria for patients with suspected or confirmed COVID-19, also known as severe acute respiratory syndrome coronavirus 2 (SARS-CoV-2), are presented in Appendix O.

Specific criteria for Dato-DXd delay, dose interruption, re-initiation, dose reduction, and/or discontinuation of Dato-DXd are listed in the TMG Annex document to this CSP, which is applicable only to TEAEs that are assessed as related to use of Dato-DXd by the investigator(s). [redacted], follow standard clinical practice. Appropriate clinical experts should be consulted as deemed necessary.

The investigator may consider dose delays or discontinuations of Dato-DXd based on other events not listed in the TMGs (see the Annex document to this CSP) according to the participant's condition and after discussion with the Study Clinical Lead or designee.

On improvement of an AE for which Dato-DXd was temporarily delayed, Dato-DXd may be restarted at the same dose at the discretion of the investigator. If a further episode of the same AE subsequently requires dose delay, or if a different AE subsequently requires dose delay, Dato-DXd may be restarted at a [redacted] on improvement of the AE or discontinued if the participant is receiving the [redacted].

[redacted]

[redacted]

Appropriate and optimal treatment of the toxicity should be attempted prior to considering dose modifications.

If a participant experiences a clinically significant and/or unacceptable toxicity, dosing will be delayed, interrupted, or permanently discontinued in accordance with the TMGs and supportive therapy administered as required.

6.6 Treatment after the end of the study

As described in Section 4.4, the study will remain open until all patients have discontinued study intervention and completed their last expected visit/contact.

After the final DCO in each arm of this study, AstraZeneca will continue to supply IP to patients who received durvalumab, paclitaxel and/or the novel oncology therapy until meeting any of the discontinuation criteria as defined in Section 7.1. Participants should be followed according to the institution's standard of care assessments. No further data collection is required, except for reporting of SAEs and AEs.

AstraZeneca will continue [redacted] while, in the opinion of the Investigator, the participant is benefiting.

In the event that [redacted] a point [redacted], then [redacted] will be discussed by AstraZeneca with the Investigator. AstraZeneca will work with the Investigator to [redacted], where possible.

In the event that a [redacted] is available at the time of the final DCO and database closure, patient(s) currently receiving treatment with IP may then be transitioned to such a study, and the current study may reach its end. The roll-over or extension study would ensure treatment continuation with visit assessments per its protocol, as applicable. Any patient who would be eligible [redacted] would be given a [redacted] as applicable.

7 DISCONTINUATION OF TREATMENT AND PATIENT WITHDRAWAL

7.1 Discontinuation of study treatment

An individual patient will not receive further IP (durvalumab, paclitaxel and/or the novel oncology therapy according to the guidelines in Section 7.1.1) if any of the following occurs in the patient in question:

- Withdrawal of consent from further treatment with IP. The patient is, at any time, free to discontinue treatment, without prejudice to further treatment. A patient who discontinues treatment is normally expected to continue to participate in the study (eg, for safety and survival follow-up) unless they specifically withdraw their consent to all further participation in any study procedures and assessments (see Section 7.3).
- An AE that, in the opinion of the Investigator or AstraZeneca, contraindicates further dosing
- Any AE that meets the criteria for discontinuation as defined in the dosing modification and toxicity management guidelines for durvalumab or any novel oncology therapy (see Section 8.4.6) or as defined in the local prescribing information for paclitaxel
- Pregnancy or intent to become pregnant
- Non-compliance with the study protocol that, in the opinion of the Investigator or AstraZeneca, warrants withdrawal from treatment with IP (eg, refusal to adhere to scheduled visits)
- Initiation of alternative anticancer therapy including another investigational agent
- Clinical progression or radiological progression (refer to Appendix F) and Investigator determination that the patient is no longer benefiting from treatment with IP

As this study is testing novel investigational products, emerging information from this study or another study that impacts the safety or the benefit/risk profile may lead to the permanent closure of any of the study arms.

7.1.1 Procedures for discontinuation of study treatment

In the event that durvalumab is discontinued, the novel oncology therapy must also be discontinued, with the exception of capivasertib, DS-8201a, and Dato-DXd, which may be continued in the absence of durvalumab (based on data from the PAKT study, DS8201-A-J101 study, and DS1062-A-J101 study, respectively).

Paclitaxel may still be administered as scheduled at the Investigator's discretion (for Arms 1 through 5). For each treatment arm, in the event that the novel oncology therapy is discontinued due to a toxicity attributed to that agent only, durvalumab and/or paclitaxel may still be administered as scheduled at the Investigator's discretion. In the event that paclitaxel is discontinued, durvalumab and/or the novel oncology therapy can be continued as scheduled at the Investigator's discretion.

Discontinuation of study treatment, for any reason, does not impact the patient's participation in the study. A patient who decides to discontinue IP will always be asked about the reason(s) for discontinuation and the presence of any AE. The patient should continue attending subsequent study visits, and data collection should continue according to the study protocol. If the patient does not agree to continue in-person study visits, a modified follow-up must be arranged to ensure the collection of endpoints and safety information. This follow-up could be a telephone contact with the patient, a contact with a relative or treating physician, or information from medical records. The approach taken should be recorded in the medical records. A patient that agrees to a modified follow-up is not considered to have withdrawn consent or to have withdrawn from the study.

Patients who are permanently discontinued from further receipt of IP, regardless of the reason, will be identified as having permanently discontinued treatment. Patients who have permanently discontinued will enter follow-up (see Table 7).

Patients who permanently discontinue drug for reasons other than objective RECIST PD should continue to have RECIST scans performed q8w ±1 week (Arms 1 through 5) and every 6 weeks (q6w) ±1 week (Arms 6, 7, and 8) for the first 48 weeks (relative to the date of treatment assignment), and then q12w ±1 week thereafter until RECIST 1.1-defined radiological progression or until the patient has been taken off-study (end of study, death, or patient withdrawal of consent) as defined the SoAs (see Section 1.1).

If a patient is discontinued for RECIST 1.1-defined progression, then the patient should have 1 additional follow-up scan performed preferably at the next (and no later than the next) scheduled imaging visit, and no less than 4 weeks after the prior assessment of PD.

All patients will be followed for survival until the end of the study.

Patients who decline to return to the site for evaluations should be contacted by telephone, following the timing and procedures indicated in the SoAs, as an alternative.

Patients who have permanently discontinued from further receipt of IP will need to be discontinued from the RTSM (IRT).

7.2 Lost to follow-up

Patients will be considered lost to follow-up only if no contact has been established by the time the study is completed (see Section 4.4), such that there is insufficient information to determine the patient's status at that time. Patients who refuse to continue participation in the study, including telephone contact, should be documented as "withdrawal of consent" rather than "lost to follow-up." Investigators should document attempts to re-establish contact with missing patients throughout the study period. If contact with a missing patient is re-established, the patient should not be considered lost to follow-up and evaluations should resume according to the protocol.

In order to support key endpoints of PFS and OS analyses, the survival status of all patients in the full analysis and the safety analysis sets should be re-checked, this includes those patients who withdrew consent or are classified as "lost to follow up."

- Lost to Follow-up – site personnel should check hospital records, the patients' current physician, and a publicly available death registry (if available) to obtain a current survival status. (The applicable case report form [CRF] modules will be updated.)
- In the event that the patient has actively withdrawn consent to the processing of their personal data, the survival status of the patient can be obtained by site personnel from publicly available death registries (if available), where it is possible to do so under applicable local laws to obtain a current survival status. (The applicable CRF modules will be updated.)

7.3 Withdrawal from the study

Patients are free to withdraw from the study at any time (IP and assessments) without prejudice to further treatment.

Patients who withdraw consent from further participation in the study will not receive any further IP or further study observation, with the exception of follow-up for survival, which will continue until the end of the study unless the patient has expressly withdrawn their

consent to survival follow-up. Note that the patient may be offered additional tests or tapering of treatment to withdraw safely.

A patient who withdraws consent will always be asked about the reason(s) for withdrawal and the presence of any AE. The Investigator will follow up AEs outside of the clinical study.

If a patient withdraws consent, they will be specifically asked if they are withdrawing consent to:

- All further participation in the study including any further follow-up (eg, survival contact telephone calls)
- Withdrawal from the use of any samples (see Section 8.8.6)

8 STUDY ASSESSMENTS AND PROCEDURES

Study procedures and their timing are summarized in the SoAs.

The Investigator will ensure that data are recorded on the eCRFs. The Web Based Data Capture system will be used for data collection and query handling.

The Investigator ensures the accuracy, completeness, and timeliness of the data recorded and of the provision of answers to data queries according to the Clinical Study Agreement. The Investigator will sign the completed eCRFs. A copy of the completed eCRFs will be archived at the study site.

Immediate safety concerns should be discussed with the Sponsor immediately upon occurrence or awareness to determine if the patient should continue or discontinue study treatment.

Adherence to the study design requirements, including those specified in the SoA, is essential and required for study conduct.

All pre-screening and screening evaluations must be completed and reviewed to confirm that potential patients meet all eligibility criteria. The Investigator will maintain pre-screening and screening logs to record details of all patients pre-screened/screened and to confirm eligibility or record reasons for screening failure, as applicable.

Procedures conducted as part of the patient's routine clinical management (eg, blood count and imaging assessments) and obtained before signing of the ICF may be utilized for screening or baseline purposes provided the procedures met the protocol-specified criteria and were performed within the time frame defined in the SoA.

Following the final DCO date by study arm, participants will be managed per SoC assessments at Investigator discretion.

8.1 Efficacy assessments

Part 1 will evaluate safety as the primary objective. Efficacy assessments in Part 1 of ORR, PFS, and DoR will be derived (by AstraZeneca) using Investigator RECIST 1.1 assessments (Appendix F). Part 2 will evaluate efficacy as the primary objective, by conducting a combined analysis of Part 1 and Part 2 data, with a primary endpoint of ORR. Efficacy assessments in the combined analysis of Part 1 and Part 2 data will be on the endpoints of ORR, PFS, DoR, and PFS6, which will be derived using RECIST 1.1 assessments. OS will also be evaluated using cumulative data from both Parts 1 and 2.

According to RECIST 1.1, objective tumor response (CR or PR) should be confirmed preferably at the next scheduled imaging visit and not less than 4 weeks after the visit when the response was last observed.

In Parts 1 and 2, all tumor assessments should be reported on the eCRF until enrollment is complete and all patients have had the opportunity to complete at least 2 on-treatment response evaluations; AstraZeneca will inform the sites once this milestone has been reached. Following the final DCO date by study arm, participants will be managed per SoC assessments at Investigator discretion.

8.1.1 Survival assessments

Assessments for survival must be made every [redacted] following treatment discontinuation. Survival information may be obtained via telephone contact with the patient or the patient's family, or by contact with the patient's current physician. The details of first and subsequent therapies for cancer, after discontinuation of treatment, will be collected.

In addition, patients on treatment or in survival follow-up will be contacted following the DCO for the final analysis to provide complete survival data. These contacts should generally occur within [redacted] of the DCO.

8.1.2 Central Reading of Scans

Images, including unscheduled visit scans, may be collected on an ongoing basis and sent to an AstraZeneca-appointed imaging CRO (iCRO) for quality control and storage, and for BICR. Guidelines for image acquisition, de-identification, storage of digital copies at the investigative site (as source documents), and transfer to the iCRO will be provided in the imaging acquisition guideline handling document. Digital copies of all original scans should be stored at the investigator site as source documents. Electronic image transfer from the sites to the iCRO is strongly encouraged. A [redacted] of [redacted]

Further details of the BICR will be documented in an Independent Review Charter.

8.2 Safety assessments

Planned timepoints for all safety assessments are provided in the SoA.

8.2.1 Clinical safety laboratory assessments

Blood and urine samples for determination of clinical chemistry, hematology, and urinalysis will be taken at the times indicated in the assessment schedules and as clinically indicated (see the SoAs). Individual sites are required to indicate in the unscheduled visit eCRF if a urinalysis was performed during study treatment.

Clinical laboratory safety tests, including serum pregnancy tests, will be performed in a licensed clinical laboratory according to local standard procedures. Sample tubes and sample sizes may vary depending on the laboratory method used and routine practice at the site. Pregnancy tests may be performed at the site using a licensed test (eg, urine or serum pregnancy test; a serum test is required within 7 days prior to the first dose of study drug; see additional details in the SoAs [Section 1.1]). Abnormal clinically significant laboratory results should be repeated as soon as possible (preferably within 24 to 48 hours).

Additional safety samples may be collected if clinically indicated at the discretion of the Investigator. The date, time of collection, and results (values, units, and reference ranges) will be recorded on the appropriate eCRF.

The laboratory variables to be measured are presented in Table 23 (clinical chemistry), Table 24 (hematology), Table 25 (coagulation), and Table 26 (urinalysis).

Other safety tests to be performed locally at screening include assessment for [redacted] antibodies, [redacted] antibodies (in Arm [redacted]), and [redacted] antibodies. For HBV active patients, [redacted] testing of [redacted] is required [redacted] See Section 6.4.1 for specification of action to take if HBV DNA becomes detectable during study treatment.

Prior HIV serology (anti-HIV with or without HIV RNA, as appropriate), hepatitis B serology (HBsAg, anti-HBs, and anti-HBc with or without HBV DNA, as appropriate), hepatitis C serology (anti-HCV antibody with or without HCV RNA, as appropriate), and hepatitis D serology (anti-HDV antibody) testing results can be used if performed within 120 days before enrolment. In this case, there is no need for a repeat test during the 28-day screening period.

Pregnancy tests may be performed at the site using a licensed test (urine beta-human chorionic gonadotropin pregnancy or serum test per institutional guideline) for all female participants of child-bearing potential. A negative serum pregnancy test (test must have a sensitivity of at least 25 mIU/mL) must be documented during screening for all female participants of child-bearing potential. If a serum pregnancy test is collected greater than 72 hours prior to the first dose of study intervention, perform a repeat pregnancy test (urine or serum per institutional guideline) within 72 hours before the first dose of study intervention. Perform repeat pregnancy tests (urine or serum per institutional guideline) within 72 hours before each infusion at each cycle, at EoT, and the first follow-up visit. A positive urine pregnancy test result must immediately be confirmed using a serum test. If a positive urine pregnancy test result is confirmed using a serum test, then the participant should not be treated.

Table 23 Clinical chemistry

Albumin	Creatinine[d]	Sodium
Alkaline phosphatase	Gamma glutamyltransferase[c]	Total bilirubin[a]
ALT[a]	Glucose[e]	Total protein
Amylase[b]	HbA1c[f]	TSH[g]
AST[a]	LDH CCI [redacted]	T3 free[h] (reflex)
Bicarbonate[c]	Lipase[b]	T4 free[h] (reflex)
Calcium	Magnesium[c]	Urea or blood urea nitrogen, depending on local practice
Chloride[c]	Potassium	

[a] Tests for ALT, AST, alkaline phosphatase, and total bilirubin must be conducted and assessed concurrently. If total bilirubin is ≥2 × upper limit of normal (and no evidence of Gilbert's syndrome), then fractionate into direct and indirect bilirubin.
[b] It is preferable that both amylase and lipase parameters are assessed. For sites where only 1 of these parameters is routinely measured, either lipase or amylase is acceptable.
[c] Bicarbonate (where available), chloride, creatinine clearance, gamma glutamyltransferase, and magnesium testing are to be performed at baseline, on Day 1 (first infusion day) (unless all screening laboratory clinical chemistry assessments are performed within 3 days prior to Day 1), and if clinically indicated.
[d] Creatinine clearance will be calculated by the site using Cockcroft-Gault (using actual body weight).
[e] Glucose will be evaluated in the fasted state according to the SoAs (Section 1.1). In all arms except Arm 2 (capivasertib), fasted glucose will be evaluated only at baseline (screening) for all other assessments, fasted or non-fasted glucose can be used.
[f] HbA1c will be evaluated according to the SoAs (Section 1.1). In all arms except Arm 2 (capivasertib), fasted HbA1c will be evaluated only at baseline (screening). HbA1c evaluation is not applicable to Arms 7 and 8.
[g] If TSH is measured within 14 days prior to Day 1 (first infusion day), it does not need to be repeated at Day 1.
[h] Free T3 or free T4 will only be measured if TSH is abnormal or if there is clinical suspicion of an AE related to the endocrine system.

AE Adverse event; ALT Alanine aminotransferase; AST Aspartate aminotransferase; HbA1c Glycated hemoglobin; LDH lactate dehydrogenase;T3 Triiodothyronine; T4 Thyroxine; TSH Thyroid-stimulating hormone.

Table 24 Hematology

Absolute neutrophil count[a]	Absolute lymphocyte count[a]
Hemoglobin	Platelet count
Total white cell count	

[a] Can be recorded as absolute counts or as percentages.

Table 25 Coagulation

Activated partial thromboplastin time	International normalized ratio
Prothrombin time	

Note: For coagulation parameters, activated partial thromboplastin time and international normalized ratio are only to be assessed at baseline on Cycle 1 Day 1 (unless all screening laboratory hematology assessments are performed within 3 days prior to Cycle 1 Day 1), and 24 hours prior to tumor biopsy (if applicable), unless clinically indicated. Assessments will also take place at screening and the end of treatment visit.

PD Progression of disease.

Table 26 Urinalysis

Bilirubin	Ketones
Blood	pH
Color and appearance	Protein
Glucose	Specific gravity

Note: Urinalysis should be done at study visits according to the SoA. In all arms, urinalysis will be done at baseline (screening) and then as clinically indicated.

Note: Microscopy should be used as appropriate to investigate white blood cells and use the high power field for red and white blood cells.

If a patient shows an AST or ALT ≥3 × ULN together with total bilirubin ≥2 × ULN, refer to Appendix E for further instructions on cases of increases in liver biochemistry and evaluation of potential Hy's Law. See also Section 8.4.6 on toxicity management. These cases should be reported as SAEs if, after evaluation, they meet the criteria for a Hy's law case or if any of the individual liver test parameters fulfill any of the SAE criteria.

All patients should have further chemistry profiles performed at [redacted] after permanent discontinuation of IP (see the SoAs).

Any clinically significant abnormal laboratory values should be repeated as clinically indicated and recorded on the eCRF. Situations in which laboratory safety results should be reported as AEs are described in Section 8.3.7.

All patients with Grade 3 or 4 laboratory values at the time of completion or discontinuation from IP must have further tests performed until the laboratory values have returned to Grade 1 or 2, unless these values are not likely to improve because of the underlying disease.

8.2.2 Physical examinations

Physical examinations will be performed according to the assessment schedules (see the SoAs). Full physical examinations will include assessments of the head, eyes, ears, nose, and

throat and the respiratory, cardiovascular, gastrointestinal, urogenital, musculoskeletal, neurological, dermatological, hematologic/lymphatic, and endocrine systems. Height will be measured at screening only. Targeted physical examinations are to be utilized by the Investigator on the basis of clinical observations and symptomatology. Situations in which physical examination results should be reported as AEs are described in Section 8.3.7.

8.2.3 Vital signs

Vital signs (BP, pulse, temperature, and respiration rate) are to be taken before blood collection for laboratory tests and will be evaluated according to the SoAs. Blood pressure assessment will include of one pulse and 3 blood pressure measurements (3 consecutive blood pressure readings will be recorded at intervals of at least one minute). The average of the 3 blood pressure readings will be recorded in the eCRF. Body weight is also recorded at each visit along with vital signs.

First infusion

On the first infusion day, patients in all treatment arms will be monitored and vital signs collected/recorded in eCRF prior to, during, and after infusion of IP as presented in the bulleted list below. Patients should be carefully monitored during the initial infusion for possible haemodynamic changes, associated with a possible infusion reaction.

BP and pulse will be collected from patients in all arms before and after each infusion at the following times (based on a [redacted] infusion or [redacted] infusion for DS-8201a [Arm 6] and Dato-DXd [Arms 7 and 8]):

- Prior to the beginning of the infusion (measured once from approximately [redacted] before up to [redacted] [ie, the beginning of the infusion])
- At the end of the infusion (approximately [redacted] following a [redacted] infusion or [redacted] following a [redacted] infusion for DS-8201a [Arm 6] and Dato-DXd [Arms 7 and 8])

If the infusion takes longer than [redacted] for Dato-DXd or 60 minutes for durvalumab, then BP and pulse measurements should follow the principles as described above or be taken more frequently if clinically indicated. A [redacted] observation period is recommended after the first infusion of durvalumab and any novel oncology therapy administered IV. If no infusion is done for the visit, vital signs will only be measured once for the visit.

Subsequent infusions

BP, pulse, and other vital signs should be measured, collected/recorded in eCRF prior to the start of the infusion. For Arms 7 and 8, BP and pulse are to be performed before and after subsequent infusions at Day 1 of each subsequent cycle and at the end of treatment (EOT). However, if the patient has tolerated previous infusions, BP and pulse may be collected prior

to the Dato-Dxd infusion (first infusion) and after completion of the durvalumab infusion (last infusion).

Patients should be carefully monitored, and BP and other vital signs should be measured during and post infusion per institution standard and as clinically indicated. Any clinically significant changes in vital signs should be entered onto an unscheduled vital signs CRF page.

Situations in which vital signs results should be reported as AEs are described in Section 8.3.7. For any AEs of infusion reactions, the vital signs values should be entered into the CRF.

8.2.4 Electrocardiograms

Resting 12-lead ECGs will be recorded in triplicate according to the SoAs. ECGs should be obtained after the patient has been in a supine position for 5 minutes ±2 minutes and recorded while the patient remains in that position.

Situations in which ECG results should be reported as AEs are described in Section 8.3.7.

Individual sites are required to indicate in the unscheduled visit eCRF if an ECG was performed during study treatment.

8.2.5 Echocardiograms/multiple gated acquisition scans

Echocardiogram/MUGA scans will be performed at screening, as clinically indicated throughout the study, and at additional timepoints in selected treatment arms (see the SoAs). The modality of the cardiac function assessments must be consistent within a patient (ie, if ECHO is used for the screening assessment, then ECHO should also be used for subsequent scans if required). The patients should also be examined using the same machine and operator whenever possible. Patients should have high-quality, standardized 2-dimensional with Doppler echocardiographic examinations performed by an experienced sonographer. LVEF determinations will be made quantitatively based on bi-plane measurements of end-diastolic and end-systolic left ventricular volume.

8.2.6 Early patient review for safety

It is recommended that patients are contacted [redacted] after receiving the durvalumab dose in each of the [redacted] of study treatment (Cycle 1 Day 15, [redacted]) to ensure early identification and management of toxicities. A study visit will occur on Day 15 of Cycle 1 and will include the assessments noted in Table 1, Table 2, Table 3, and Table 4. On Day 15 of Cycle 1 (Arms 7 and 8), and Cycle 2 and Cycle 3 (Arms 1 to 8), the form of contact and procedures conducted will be per the SoA or at the Investigator's discretion, if not specified in the SoA.

8.2.7 [redacted]

Patients receiving capivasertib will be given [redacted]. This dosing diary is to be completed each time capivasertib is taken.

8.2.8 WHO/ECOG performance status

WHO/ECOG PS will be assessed at the times specified in the assessment schedules (see the SoAs) based on the following:

- Fully active; able to carry out all usual activities without restrictions
- Restricted in strenuous activity, but ambulatory and able to carry out light work or work of a sedentary nature (eg, light housework or office work)
- Ambulatory and capable of self-care, but unable to carry out any work activities; up and about more than 50% of waking hours
- Capable of only limited self-care; confined to bed or chair more than 50% of waking hours
- Completely disabled; unable to carry out any self-care and totally confined to bed or chair
- Dead

Any significant change from baseline or screening must be reported as an AE.

8.2.9 Other safety assessments

8.2.9.1 Pneumonitis/ILD investigation

If new or worsening pulmonary symptoms (eg, dyspnea) or radiological abnormality suggestive of pneumonitis/ILD is observed, a full investigation is required as described in detail in the dosing modification and toxicity management guidelines for durvalumab (see Section 8.4.6.1), for DS-8201a (for patients in Arm 6; see Section 8.4.6.5), and for Dato-DXd (for patients in Arms 7 and 8; see Section 8.4.6.6); if this is a potential risk for the specific treatment arm, then specific safety management guidelines will be applied (see Section 8.4.6). The results of the full diagnostic workup (including high-resolution computed tomography [HRCT] per institutional guidelines, blood and sputum culture, hematological parameters, etc) will be captured in the eCRF. It is strongly recommended to perform a full diagnostic workup to exclude alternative causes such as lymphangitic carcinomatosis, infection, allergy, cardiogenic edema, or pulmonary hemorrhage. In the presence of confirmatory HRCT scans where other causes of respiratory symptoms have been excluded, a diagnosis of pneumonitis (ILD) should be considered and the dosing modification and toxicity management guidelines should be followed.

The following assessments, and additional assessments if required, will be performed to enhance the investigation and diagnosis of potential cases of pneumonitis. The results of the assessment will be collected.

- Physical examination
  - Signs and symptoms (cough, shortness of breath, and pyrexia, etc) including auscultation for lung field will be assessed.
- [redacted]
  - [redacted]
- Other items
  - When pneumonitis (ILD) is suspected during study treatment, the following markers should be measured where possible:
    - ILD markers ([redacted]) and [redacted]
    - Tumor markers: particular tumor markers [redacted]
    - Additional clinical chemistry: [redacted]
    - Blood sample for serum PK (only for patients in Arms 6, 7, and 8)

8.2.9.2 Safety assessments for durvalumab + DS-8201a (Arm 6) and durvalumab + Dato-DXd (Arms 7 and 8)

Pulmonary assessments

[redacted] should be obtained within [redacted] prior to Cycle 1 Day 1. [redacted] should be obtained before and after each infusion per the Arm 6 SoA in Table 4 and before and after each infusion at Day 1 for Cycle 1 to Cycle 3, prior to infusion from Cycle 4 onwards and at the EOT per Arms 7 and 8 SoA in Table 5 and Table 6. [redacted] should be evaluated by PI or the delegate physician prior to the administration of IMP at each visit.

Pulmonary function test (PFT), as a minimum should include spirometry. Diffusion capacity of the lungs for carbon monoxide (DLCO) will be performed (when feasible), but for patients with prior severe and/or clinically significant pulmonary disorders, DLCO is a requirement.

Pulmonary HRCT (or CT if HRCT is contraindicated) will be performed according to institutional guidelines as per the SoA in Table 4, Table 5, and Table 6.

[redacted] including but not limited to [redacted] and [redacted] will be performed as specified in the SoA, and as clinically indicated by an [redacted] or if unavailable, another [redacted]. A suitable alternative to [redacted]

[redacted] may be used in exceptional circumstances where [redacted]. [redacted] should be considered for any [redacted] including, but not limited to, [redacted] should be documented in the eCRF. Copies of all [redacted] should be filed in source notes. Please refer [redacted] for further details.

[redacted] The use of [redacted].

Further recommendations for preventing and treating [redacted] are available in the SoA and the TMGs (see the TMG Annex document to this CSP).

Any significant change from baseline must be reported as an AE (see Section 8.3.7).

8.2.9.3 [redacted]

As per Investigator judgement, a professional [redacted] before study drug initiation and [redacted], if indicated, may reduce the risk of [redacted] should be initiated as described in Section 6.1.1.7.

8.3 Collection of adverse events

The Principal Investigator is responsible for ensuring that all staff involved in the study are familiar with the content of this section.

The definitions of an AE or SAE can be found in Appendix B.

AE will be reported by the patient (or, when appropriate, by a caregiver, surrogate, or the patient's legally authorized representative).

The Investigator and any designees are responsible for detecting, documenting, and recording events that meet the definition of an AE or SAE. For information on how to follow up AEs, see Section 8.3.3.

8.3.1 Method of detecting AEs and SAEs

Care will be taken not to introduce bias when detecting AEs and/or SAEs. Open-ended and non-leading verbal questioning of the patient is the preferred method to inquire about AE occurrences.

8.3.2 Time period and frequency for collecting AE and SAE information

Only SAEs will be collected from the time of the patient signing the pre-screening ICF until the main ICF is signed. AEs and SAEs will be collected from the time of the patient signing the main ICF until the follow-up period is completed [redacted] after the last dose of study treatment). If an event that starts post the defined safety follow-up period noted above is considered to be due to a late-onset toxicity to study drug, then it should be reported as an AE or SAE as applicable [redacted]

All SAEs will be recorded and reported to the Sponsor or designee within 24 hours, as indicated in Appendix B. The Investigator will submit any updated SAE data to the Sponsor within 24 hours of it being available. Investigators are not obligated to actively seek an AE or SAE in former study patients. However, if the Investigator learns of any SAE, including a death, at any time after a patient's last visit and he/she considers the event to be reasonably related to the study treatment or study participation, the Investigator should notify the Sponsor.

The method of recording, evaluating, and assessing causality of AE and SAE and the procedures for completing and transmitting SAE reports are provided in Appendix B.

8.3.3 Follow-up of AEs and SAEs

After the initial AE/SAE report, the Investigator is required to proactively follow each patient at subsequent visits/contacts. All SAEs, non-serious AEs, and AESIs (as defined in Section 8.3.14) will be followed until resolution, stabilization, the event is otherwise explained, or the patient is lost to follow-up.

Any AEs that are unresolved at the patient's last AE assessment or other assessment/visit as appropriate in the study are followed up by the Investigator for as long as medically indicated (this may be beyond the [redacted] after the last dose of study treatment), but without further recording in the eCRF. AstraZeneca retains the right to request additional information for any patient with ongoing AE(s)/SAE(s) at the end of the study, if judged necessary.

8.3.4 Adverse event data collection

The following variables will be collected for each AE:

- AE (verbatim)
- The date when the AE started and stopped
- The maximum CTCAE grade reported
- Changes in CTCAE grade (report only the maximum CTCAE grade for a calendar day)
- Whether the AE is serious or not

- Investigator causality rating against the IPs (yes or no)
- Action taken with regard to IPs
- Administration of treatment for the AE
- Outcome

In addition, the following variables will be collected for SAEs where applicable:

- Date the AE met criteria for SAE
- Date the Investigator became aware of the SAE
- Seriousness criteria
- Date of hospitalization
- Date of discharge
- Probable cause of death
- Date of death
- Whether an autopsy was performed
- Causality assessment in relation to study procedure(s)
- Causality assessment in relation to other medication, as explained in Section 8.3.5
- Description of the SAE

The grading scales found in the revised NCI CTCAE version 4.03 will be utilized for all events with an assigned CTCAE grading. For those events without assigned CTCAE grades, the recommendation in the CTCAE criteria that converts mild, moderate, and severe events into CTCAE grades should be used. A copy of the CTCAE version 4.03 can be downloaded from the Cancer Therapy Evaluation Program website (http://ctep.cancer.gov).

8.3.5 Causality collection

The Investigator will assess the causal relationship between IP and each AE with respect to each IP (durvalumab, paclitaxel, and the novel oncology therapy [if applicable]), and answer ‘yes’ or ‘no’ to the question, ‘Do you consider that there is a reasonable possibility that the event may have been caused by the investigational product?’

For SAEs, causal relationship will also be assessed for other medication and study procedures. Note that for SAEs that could be associated with any study procedure, the causal relationship is implied as ‘yes.’

A guide to the interpretation of the causality question is found in Appendix B to the Clinical Study Protocol (CSP).

8.3.6 Adverse events based on signs and symptoms

All AEs spontaneously reported by the patient or reported in response to the open question from the study site staff: "Have you had any health problems since the previous visit/you were last asked?," or revealed by observation will be collected and recorded in the CRF. When collecting AEs, the recording of diagnoses is preferred (when possible) to recording a list of signs and symptoms. However, if a diagnosis is known and there are other signs or symptoms that are not generally part of the diagnosis, the diagnosis and each sign or symptom will be recorded separately.

8.3.7 Adverse events based on examinations and tests

The results from the CSP mandated laboratory tests and vital signs will be summarized in the CSR. Deterioration as compared to baseline in protocol-mandated laboratory values, vital signs, and CCI [redacted] should therefore only be reported as AEs if they fulfill any of the SAE criteria, are a DLT, are the reason for discontinuation of treatment with the IP, or are considered to be clinically relevant as judged by the investigator (which may include, but is not limited to consideration as to whether treatment or non-planned visits were required or other action was taken with the study treatment, eg, dose adjustment or drug interruption).

If deterioration in a laboratory value/vital sign/CCI [redacted] is associated with clinical signs and symptoms, the sign or symptom will be reported as an AE and the associated laboratory result/vital sign/CCI [redacted] will be considered as additional information. Wherever possible, the reporting Investigator uses the clinical rather than the laboratory term (eg, anemia versus low hemoglobin value). Any diagnosis of the undesirable clinical outcome of 'left ventricular dysfunction', a valid or qualifying reduction of LVEF (as measured by MUGA or ECHO) should be confirmed and included in the AE report. In the absence of clinical signs or symptoms, clinically relevant deteriorations in non-mandated parameters should be reported as AE(s).

Deterioration of a laboratory value, which is unequivocally due to PD, should not be reported as an AE/SAE.

Any new or aggravated clinically relevant abnormal medical finding at a physical examination as compared with the baseline assessment will be reported as an AE unless unequivocally related to the disease under study (DUS), see Sections 8.3.9 and 8.3.11.

8.3.8 Hy's law

Cases wherein a patient shows elevations in liver biochemistry may require further evaluation, and occurrences of AST or ALT $\geq 3 \times$ ULN together with total bilirubin $\geq 2 \times$ ULN may need to be reported as SAEs. Please refer to Appendix E for further instruction on cases of increases in liver biochemistry and evaluation of Hy's Law.

8.3.9 Pneumonitis/ILD Cases

All potential ILD/pneumonitis cases should be reported within 24 hours, including both serious and non-serious potential ILD/pneumonitis cases (refer to Section 8.2.9.1).

8.3.10 Disease under study

Symptoms of DUS are those that might be expected to occur as a direct result of TNBC. Events that are unequivocally due to DUS should not be reported as an AE during the study unless they meet SAE criteria or lead to discontinuation of the IP.

8.3.11 Disease progression

PD can be considered as a worsening of a patient's condition attributable to the disease for which the IP is being studied. It may be an increase in the severity of the DUS and/or increases in the symptoms of the disease. The development of new metastases, or progression of existing metastasis to the primary cancer under study, should be considered as PD and not an AE. Events that are unequivocally due to PD should not be reported as an AE during the study.

8.3.12 New cancers

The development of a new cancer should be regarded as an SAE. New primary cancers are those that are not the primary reason for the administration of the IP and have been identified after the patient's inclusion in this study.

8.3.13 Deaths

All deaths that occur during the study treatment period, or within the protocol-defined follow-up period after the administration of the last dose of study drug, must be reported as follows:

- Death clearly resulting from PD should be reported in the eCRF in the Statement of Death page. It should not be reported as an SAE.
- Where death is not due (or not clearly due) to progression of the DUS, the AE causing the death must be reported to the Study Clinical Lead as an SAE within 24 hours. It should also be documented in the Statement of Death page in the eCRF. The report should contain a comment regarding the co-involvement of PD, if appropriate, and should assign main and contributory causes of death.
- Deaths with an unknown cause should always be reported as an SAE. It should also be documented in the Statement of Death page in the eCRF. A postmortem may be helpful in the assessment of the cause of death, and if performed, a copy of the postmortem results should be forwarded to AstraZeneca Patient Safety or its representative within the usual timeframes.

Deaths occurring after the protocol-defined safety follow-up period after the administration of the last dose of study drug should be documented in the Statement of Death page. If the death

occurred as a result of an event that started post the defined safety follow-up period and the event is considered to be due to a late-onset toxicity to study drug, then it should also be reported as an SAE.

8.3.14 Adverse events of special interest

An AESI is an AE of scientific and medical interest specific to understanding of the IP and may require close monitoring. An AESI may be serious or non-serious. The rapid reporting of AESIs allows ongoing surveillance of these events in order to characterize and understand them in association with the use of the study treatment.

All AESIs, regardless of severity or seriousness, must be followed until either event resolution, end of study, including any post-treatment follow-up, trial termination, withdrawal of consent, or participant death.

Concomitant medications administered as treatment for drug-related AESIs should be recorded until either event resolution, end of study, trial termination, withdrawal of consent, or participant death.

8.3.14.1 Durvalumab

AESIs for durvalumab include, but are not limited to, events with a potential inflammatory or immune-mediated mechanism and which may require more frequent monitoring and/or interventions such as corticosteroids, immunosuppressants, and/or endocrine hormone replacement therapy. These AESIs are being closely monitored in clinical studies with durvalumab monotherapy and combination therapy. An imAE is defined as an AESI that is associated with drug exposure and is consistent with an immune-mediated MOA and where there is no clear alternate etiology.

Serologic, immunologic, and histologic (biopsy) data, as appropriate, should be used to support an imAE diagnosis. Appropriate efforts should be made to rule out neoplastic, infectious, metabolic, toxin, or other etiologic causes of the imAE.

If the Investigator has any questions in regards to an event being an imAE, the Investigator should promptly contact the Study Clinical Lead.

AESIs observed with anti PD-L/PD-1 agents such as durvalumab include the following:

- Diarrhea/colitis and intestinal perforation
- Pneumonitis/ILD
- Endocrinopathies (ie, events of hypophysitis/hypopituitarism, adrenal insufficiency, hyper- and hypo-thyroidism, and type I diabetes mellitus)
- Hepatitis/transaminase increases
- Nephritis/blood creatinine increases

- Pancreatitis/serum lipase and amylase increases
- Rash/dermatitis
- Myocarditis
- Myositis/polymyositis
- Neuropathy/neuromuscular toxicity (eg, Guillain-Barré syndrome and myasthenia gravis)
- Other inflammatory responses that are rare/less frequent with a potential immune-mediated etiology include, but are not limited to, pericarditis, sarcoidosis, uveitis, and other events involving the eye, skin, hematological, and rheumatological events, vasculitis, non-infectious meningitis and non-infectious encephalitis. It is possible that events with an inflammatory or immune-mediated mechanism could occur in nearly all organs.

In addition, IRRs and hypersensitivity/anaphylactic reactions with a different underlying pharmacological etiology are also considered AESIs.
Further information on these risks (eg, presenting symptoms) can be found in the current version of the durvalumab Investigator's Brochure. More specific guidelines for their evaluation and treatment are described in detail in the Dose Modification and Toxicity Management Guidelines (see Section 8.4.6). These guidelines have been prepared by the Sponsor to assist the Investigator in the exercise of his/her clinical judgment in treating these types of toxicities. These guidelines apply to AEs considered causally related to the study drug/study regimen by the reporting Investigator.

8.3.14.2 Capivasertib

Details on the following AESIs for capivasertib are provided in Section 2.3.2.3: hyperglycemia, rash, diarrhea, stomatitis, infection/lower respiratory tract infection, and [redacted]

8.3.14.3 Oleclumab

Guidelines for management of patients with hypersensitivity (including anaphylactic reaction) and IRRs are described in Section 8.4.6.

Infusion-related reactions

Administration of mAbs via IV can cause an acute reaction called an IRR. Acute allergic reactions may also occur during the infusion of study treatment. Manifestations of IRR and acute allergic reactions are similar and they are managed the same way. IRRs predominantly occur at the first exposure to drug and are uncommon at subsequent exposures.

Anaphylaxis

Anaphylaxis and IRRs have some common manifestations and may be difficult to distinguish from each other. IRRs are commonly observed during or shortly after the first time of

exposure to therapeutic mAbs delivered through IV infusion. These reactions are less common following subsequent exposures. Unlike IRRs, anaphylaxis is a rare event, usually occurring after subsequent exposure to an antigen, and it is most commonly accompanied by severe systemic skin and/or mucosal reactions. The Investigator is advised to carefully examine symptoms of adverse reactions observed during or shortly after exposure to oleclumab and consider the above-mentioned facts prior to making a final diagnosis. Reactions occurring at the time of or shortly after subsequent infusions of oleclumab are to be judged by the Investigator at his or her own discretion.

For the Investigator's convenience and in order to facilitate consistency in judgments, a copy of the National Institute of Allergy and Infectious Disease and Food Allergy and Anaphylaxis Network guidance for anaphylaxis diagnosis is provided in Appendix H.

Cardiac chest pain, transient ischemic attack, and thromboembolism

AEs of cardiac chest pain, transient ischemic attack, and thromboembolic events are of special interest due to oleclumab's potential risks of arterial calcifications, arterial ischemic disorder, and thrombosis. Because of this potential risk, patients with a prior history of myocardial infarction, stroke, or transient ischemic attack in the past 6 months are not eligible (see Section 5.2). These events require urgent medical management, which should be performed according to consensus guidelines developed by the American Heart Association or appropriate local standards of care.

Edema

Edema (eg, pulmonary or peripheral) is regarded as an AESI due to oleclumab's potential risks of increased microvascular permeability. For patients who develop Grade ≥3 edema, doses should be omitted per Section 6.5.2.4, and therapy may be discontinued at the discretion of the Investigator.

Immune complex disease

The immune system can respond to a foreign protein, even to a humanized mAb, by producing human anti-human antibodies, which may result in formation of immune complexes and their deposition in blood vessels, joints, and glomeruli causing symptomatic disease (eg, vasculitis, glomerulonephritis, arthritis, and serum sickness). Patients will be monitored clinically and for the presence of ADAs. Patients who experience an AE suspected to be immune-complex related and with confirmed presence of ADAs will discontinue treatment. Immune-complex disease will be managed in accordance with SoC.

8.3.14.4 DS-8201a (trastuzumab deruxtecan)

Based on the available non-clinical and clinical data, review of the cumulative literature, reported toxicities for the same class of agents and biological plausibility, the following events are considered to be AESIs:

Interstitial lung disease/Pneumonitis

ILD/pnuemonitis is considered an important identified risk based on a comprehensive cumulative review of potential ILD/pneumonitis cases reviewed by the [redacted], the available safety data from the clinical development program; available data from recent epidemiology/literature; biological plausibility; and safety information from drugs of similar class. Refer to the current IB for a summary of preliminary clinical study data.

LVEF decrease

LVEF decrease in association with DS-8201a is considered to be an important potential risk based on the available non-clinical data, literature and available safety information for drugs of similar class. Refer to the current IB for a summary of preliminary clinical trial data associated with the use of the study treatment.

8.3.14.5 Dato-DXd (datopotamab deruxtecan; DS-1062a)

Based on the available pre-clinical data, review of the cumulative literature, reported toxicities for drugs with similar monoclonal antibody and payload of Dato-DXd, and biological plausibility, the following events are considered to be AESIs:

Interstitial lung disease/Pneumonitis

ILD/pneumonitis is considered an important identified risk based on a comprehensive cumulative review of the available safety data from the clinical development program as well as the results of potential ILD cases reviewed by the independent ILD Adjudication Committee, available data from recent epidemiology/literature, biological plausibility, and safety information from drugs with similar monoclonal antibody and payload as Dato-DXd. All potential ILD/pneumonitis cases, including both serious and non-serious potential ILD/pneumonitis cases (potential ILD/pneumonitis is described in [redacted]). Refer to the current Investigator's Brochure for a summary of preliminary clinical study data.

Oral Mucositis/Stomatitis

Oral mucositis/stomatitis AEs are considered as identified risks associated with Dato-DXd treatment. Mucosal inflammation other than oral mucositis/stomatitis is also an identified risk but is considered as a separate AESI (see below). All events of stomatitis/mucosal inflammation, regardless of seriousness, must be reported. Recommendations for preventing stomatitis/mucosal inflammation can be found in Section 6.1.1.7 and recommendations for treatment of stomatitis/mucosal inflammation can be found in the Annex document to this CSP.

If a subject develops stomatitis/mucosal inflammation, it would be important for the Investigator to seek consultation with either a dentist or oral surgeon as needed, and have the subject monitored and treated appropriately.

Mucosal Inflammation Other than Oral Mucositis/Stomatitis

Mucosal inflammation AEs are considered as identified risks associated with Dato-DXd treatment and as a separate AESI from oral mucositis/stomatitis.

Ocular Surface Events

Ocular surface events (eg, dry eye, keratitis) is considered as an AESI associated with Dato-DXd treatment. Dry eye is considered as an identified risk and keratitis as an important identified risk within this AESI. All Grade ≥ 3 ocular surface events and Grade ≥ 2 keratitis events (includes keratitis, punctate keratitis, and ulcerative keratitis) for Arms 7 and 8 should be reported by the investigator in eCRF EDC AE page(s) within 24 hours of becoming aware. [redacted] provides assistance to the Investigator to assess any [redacted]. Every outlined [redacted] should be provided in a source document, as per SoA in a timely manner.

8.4 Safety reporting and medical management

8.4.1 Reporting of serious adverse events

All SAEs have to be reported, whether or not considered causally related to the IP or to the study procedure(s). All SAEs will be recorded in the eCRF.

If any SAE occurs during the course of the study, then Investigators or other site personnel inform the appropriate AstraZeneca representatives within 1 day (ie, immediately but **no later than 24 hours** of when he or she becomes aware of it).

The designated AstraZeneca representative works with the Investigator to ensure that all the necessary information is provided to the AstraZeneca Patient Safety data entry site **within 1 calendar day** of initial receipt for fatal or life-threatening events **and within 5 calendar days** of initial receipt for all other SAEs.

For fatal or life-threatening AEs where important or relevant information is missing, active follow-up is undertaken immediately. Investigators or other site personnel inform AstraZeneca representatives of any follow-up information on a previously reported SAE within 1 calendar day (ie, immediately but **no later than 24 hours** of when he or she becomes aware of it).

Once the Investigators or other site personnel indicate an AE is serious in the electronic data capture system, an automated email alert is sent to the designated AstraZeneca representative.

If the electronic data capture system is not available, then the Investigator or other study site staff reports an SAE to the appropriate AstraZeneca representative by telephone.

The AstraZeneca representative will advise the Investigator/study site staff how to proceed.

For further guidance on the definition of an SAE, see Appendix B.

8.4.2 Safety Data to be Collected Following the Final Data Cutoff of the Study

For participants continuing to receive study medication after the final DCO for each arm and database closure, AEs and SAEs will be collected and reported on a paper-based report forms or electronically as defined by the AstraZeneca.

In addition, it is recommended that participants continue the scheduled site visits and investigators monitor the participant's safety laboratory results periodically during treatment with study medication in order to manage AEs, consistent with the study medication dose modification guidelines for management of study intervention-related toxicities (see the Annex document to this CSP). All data after the final DCO for each arm and database closure will be recorded in the participant notes but only data for AEs and SAEs will be reported via paper-based report forms or electronically as defined by AstraZeneca.

All AEs and SAEs that occur in participants still receiving study medication (or within the follow-up period) after the final DCO must be reported within timelines detailed in Section 8.4.1.

8.4.3 Pregnancy

All pregnancies and outcomes of pregnancy with conception dates following the first date of study intervention, including pregnancy in the partners of male participants, should be reported to AstraZeneca, except if the pregnancy is discovered before the study participant has received any study intervention.

If a pregnancy is reported, the Investigator should inform the Sponsor within 24 hours of learning of the pregnancy.

Abnormal pregnancy outcomes (eg, spontaneous abortion, fetal death, stillbirth, congenital anomalies, and ectopic pregnancy) are considered SAEs.

8.4.3.1 Maternal exposure

If a patient becomes pregnant during the course of the study, IP should be discontinued immediately.

Pregnancy itself is not regarded as an AE unless there is a suspicion that the IP under study may have interfered with the effectiveness of a contraceptive medication. Congenital abnormalities or birth defects and spontaneous miscarriages should be reported and handled as SAEs. Elective abortions without complications should not be handled as AEs. The outcome of all pregnancies (spontaneous miscarriage, elective termination, ectopic pregnancy, normal birth, or congenital abnormality) should be followed up and documented even if the patient was discontinued from the study.

If any pregnancy occurs while the patient is on study, then the Investigator or other site personnel informs the appropriate AstraZeneca representatives within 1 day (ie, immediately but **no later than 24 hours** of when he or she becomes aware of it).

The designated AstraZeneca representative works with the Investigator to ensure that all relevant information is provided to the AstraZeneca Patient Safety data entry site within 1 or 5 calendar days for SAEs (see Section 9.2.5) and within 30 days for all other pregnancies.

The same timelines apply when outcome information is available.

8.4.3.2 Paternal exposure

Not applicable.

8.4.4 Overdose

Use of durvalumab, paclitaxel, and/or any novel oncology therapy in doses that are in excess of those specified in the protocol is considered to be an overdose. There is currently no specific treatment in the event of overdose of durvalumab or any of the novel oncology therapies, and possible symptoms of overdose are not established. Please refer to the local prescribing information for treatment in cases of an overdose related to paclitaxel. The Investigator will use clinical judgement to treat any overdose. Investigators should be advised that any patient who receives a higher dose than that intended should be monitored closely, managed with appropriate supportive care, and followed up expectantly.

- An overdose with associated AEs is recorded as the AE diagnosis/symptoms on the relevant AE modules in the CRF and on the Overdose CRF module.
- An overdose without associated symptoms is only reported on the Overdose CRF module and not the AE module.

If an overdose of a patient with durvalumab, paclitaxel, and/or any novel oncology therapy, with or without associated AEs/SAEs, occurs during the course of the study, then the Investigator or other site personnel must inform the appropriate AstraZeneca representative immediately, or **no later than 24 hours** of when he or she becomes aware of it, using the designated Safety e-mail address or by fax.

The designated Contract Research Organization/AstraZeneca representative works with the Investigator to ensure that all relevant information is provided to the AstraZeneca Patient Safety data entry site.

For overdoses associated with an SAE, the standard reporting timelines apply, see Section 8.3.2. For other overdoses, reporting must occur within 30 days.

8.4.5 Medication error, drug abuse, and drug misuse

8.4.5.1 Timelines

If an event of medication error, drug abuse, **or** drug misuse occurs during the study, then the Investigator or other site personnel informs the appropriate AstraZeneca representatives within **one calendar day,** ie, immediately but **no later than 24 hours** of when they become aware of it.

The designated AstraZeneca representative works with the Investigator to ensure that all relevant information is completed within **one** (initial fatal/life-threatening or follow-up fatal/life-threatening) **or 5** (other serious initial and follow-up) **calendar days** if there is an SAE associated with the event of medication error, drug abuse, or misuse (see Section 8.3.2) and **within 30 days** for all other events.

8.4.5.2 Medication error

For the purposes of this clinical study a medication error is an **unintended** failure or mistake in the treatment process for an IMP/study intervention or AstraZeneca NIMP that either causes harm to the participant or has the potential to cause harm to the participant.

The full definition and examples of Medication Error can be found in Appendix B-8.

8.4.5.3 Drug abuse

Drug abuse is the persistent or sporadic **intentional**, non-therapeutic excessive use of IMP/study intervention or AstraZeneca NIMP for a perceived reward or desired non-therapeutic effect.

The full definition and examples of drug abuse can be found in Appendix B-8.

8.4.5.4 Drug misuse

Drug misuse is the **intentional** and inappropriate use (by a study participant) of IMP/study intervention or AstraZeneca NIMP for medicinal purposes outside of the authorized product information, or for unauthorized IMPs/study intervention(s) or AstraZeneca NIMPs, outside the intended use as specified in the protocol and includes deliberate administration of the product by the wrong route.

The full definition and examples of Drug Misuse can be found in Appendix B-8.

8.4.6 Management of IP-related toxicities

The following general guidance should be followed for management of toxicities:

- Treat each of the toxicities with maximum supportive care (including withholding the agent suspected of causing the toxicity if required).
- If the symptoms promptly resolve with supportive care, consideration should be given to continuing the same dose of the assigned IP along with appropriate continuing supportive care. If medically appropriate, dose modifications are permitted
- All dose modifications should be documented with clear reasoning and documentation of the approach taken.

Durvalumab and oleclumab are non-cytotoxic agents and are generally classified as non-irritants although experience with their extravasation is limited.

All toxicities will be graded according to NCI CTCAE version 4.03.

8.4.6.1 Specific toxicity management and dose modification information – durvalumab and durvalumab in combination with other therapies

The Toxicity Management Guidelines have been developed to assist investigators with the recognition and management of toxicities associated with use of the immune-checkpoint inhibitors durvalumab (MEDI4736) (PD-L1 inhibitor) and tremelimumab (CTLA-4 inhibitor). Given the similar underlying mechanisms of toxicities observed with these 2 compounds, these guidelines are applicable to the management of patients receiving either drug as monotherapy or both in combination. Additionally, these guidelines are applicable when either drug is used alone or both drugs are used in combination and, also, is administered concurrently or sequentially with other anti-cancer drugs (ie, antineoplastic chemotherapy, targeted agents), as part of a protocol specific treatment regimen. The Toxicity Management Guidelines provide information for the management of immune-mediated reactions, IRRs, and non-immune mediated reactions that may be observed with checkpoint inhibitor monotherapy or combination checkpoint inhibitor regimens, with specific instructions for dose modifications (including discontinuations) and treatment interventions. Dose modifications with regards to hold or discontinuation of durvalumab are required actions for the management of potential immune-mediated events or non-immune-mediated adverse reactions, as described in the Dosing Modification and Toxicity Management Guideline; permanent discontinuation of study treatment for these adverse events is considered a study-specific requirement.

Investigators are advised however to use local practice guidelines and consult local references for the management of toxicities observed with other cancer treatment.

The most current version of the toxicity management guidelines entitled “Dosing Modification and Toxicity Management Guidelines for Durvalumab Monotherapy, Durvalumab in Combination with Other Products, or Tremelimumab Monotherapy” is provided to the investigative site as an Annex document and is maintained within the Site Master File.

Patients should be thoroughly evaluated and appropriate efforts should be made to rule out neoplastic, infectious, metabolic, toxic, or other etiologic causes of the imAE. Serologic, immunologic, and histologic (biopsy) data, as appropriate, should be used to support an imAE diagnosis. In the absence of a clear alternative etiology, events should be considered potentially immune-related.

In addition, there are certain circumstances in which durvalumab should be permanently discontinued (see Section 7.1 of this protocol and the Dosing Modification and Toxicity Management Guidelines).

Following the first dose of IP, subsequent administration of durvalumab can be modified based on toxicities observed as described in the Dosing Modification and Toxicity Management Guidelines. These guidelines have been prepared by the Sponsor to assist the Investigator in the exercise of his/her clinical judgment in treating these types of toxicities. These guidelines apply to AEs considered causally related to durvalumab by the reporting Investigator.

Dose reductions are not permitted. In case of doubt, the Investigator should consult with the Study Clinical Lead.

8.4.6.2 Specific toxicity management and dose modification information - paclitaxel

Guidelines for dose reduction to manage specific toxicities are provided in Section 6.5.2.2. In addition, Investigators should follow local standard clinical practice regarding toxicity management for paclitaxel. Please refer to the local prescribing information for paclitaxel.

8.4.6.3 Specific toxicity management and dose modification information - capivasertib

General guidelines for dose reduction, interruption, and permanent discontinuation are summarized in Section 6.5.2.3. Guidelines for the management of general toxicity (Table 20), hypersensitivity, hyperglycemia, diarrhea, and maculo-papular rash that may be attributable to capivasertib are provided in the Guidance for Management of Specific Adverse Events of capivasertib (see Appendix I).

Following the first dose of IP, subsequent administration of capivasertib can be modified based on toxicities observed as described in the Guidance for Management of Specific Adverse Events. These guidelines have been prepared by the Sponsor to assist the Investigator in the exercise of his/her clinical judgment in treating these types of toxicities. These

guidelines apply to AEs considered causally related to capivasertib by the reporting Investigator.

In addition, there are certain circumstances in which capivasertib should be permanently discontinued (see Sections 6.5.2.3 [capivasertib] and 7.1 [all IPs] of this protocol and the Guidance for Management of Specific Adverse Events for capivasertib).

8.4.6.4 Specific toxicity management and dose modification information - oleclumab

There are no guidelines specific to AEs attributable to oleclumab monotherapy. The guidelines for durvalumab in combination with other therapies may be followed.

8.4.6.5 Specific toxicity management and dose modification information - DS-8201a (trastuzumab deruxtecan)

Following the first dose of IP, subsequent administration of DS-8201 may be modified based on toxicities observed as described in the Guidance for Management of Specific Adverse events in studies of DS-8201 (Appendix L). Guidelines for the management of general toxicity, ILD/pneumonitis, LVEF reduction, transaminitis, and cytopenias that may be attributable to DS-8201 are also provided in Appendix L.

In addition, there are certain circumstances in which DS-8201 should be permanently discontinued. General guidelines for dose reduction, interruption, and permanent discontinuation are summarized in Section 6.5.2.5. All confirmed or suspected COVID-19 infection events must be recorded in the eCRF as AEs. Please refer to Appendix O for additional information on dose modification.

ILD/pneumonitis management guidance:

Early diagnosis and appropriate management of events of ILD/pneumonitis are essential to minimise serious outcomes. Patients should be monitored closely and advised to immediately report signs or symptoms of ILD/pneumonitis (eg, cough, dyspnea at rest or exertion, fever, not otherwise explained fatigue, decrease in oxygen saturation, and/or any other new or worsening respiratory symptoms). ILD education is to be re-emphasized in patients with moderate renal impairment. Physicians should consider more frequent patient contact in between visits (at least once in between cycles) in order to reinforce ILD education and the importance of prompt reporting of symptoms, until the investigator is confident the patient is familiar with the requirements.

The goal of ILD management is to supress inflammation and prevent irreversible fibrosis with potential fatal outcome. Corticosteroid treatment is considered to be most effective during the inflammatory phase of ILD. On occasions, ILD can present acutely and progress rapidly.

Initiate management promptly at the first suspicion of ILD/pneumonitis.

If the AE is suspected to be ILD/pneumonitis, treatment with study drug should be interrupted pending further evaluations. Evaluations should include:

- CT chest (HRCT preferred): the same modality should be employed as the baseline scan to allow for direct comparison of scans
- Pulmonologist consultation (infectious disease consultation if clinically indicated)
- [redacted] and [redacted] if clinically indicated and feasible
- Pulmonary function tests (including [redacted] and [redacted]) and [redacted]
- Clinical laboratory tests:
  - COVID-19 Test
  - Arterial blood gases, if clinically indicated
  - CBC, blood culture, differential WBC, CRP
  - One blood sample should be collected for pharmacokinetics as soon as ILD/pneumonitis is suspected, if feasible.
- Other tests could be considered, as needed, including [redacted] (eg, [redacted] especially in patients with 1 or more of the following risk factors: lymphopenia, long-term/intermittent steroid use, brain metastases, and chronic lung diseases.

If the AE is confirmed to be ILD/pneumonitis, promptly follow the management guidance outlined in the designated “Pulmonary Toxicity” Toxicity Management Guidelines.

For DS-8201a and Dato-DXd, [redacted] will be established to [redacted]. To ensure adequate and relevant evaluation, additional data may be collected to fully characterise medical history (eg, smoking, radiation and pulmonary history), diagnostic evaluation, treatment, and outcome of the event. To ensure adequate and relevant evaluation, systematic additional data collection will be conducted for all cases that will be brought for evaluation. This additional data collection will cover a more in-depth relevant medical history (eg, smoking, radiation, COPD and other chronic lung conditions), diagnostic evaluation, treatment, and outcome of the event.

LVEF decrease management guidance:

LVEF will be measured by either ECHO or MUGA scan. All ECHOs/MUGAs will be evaluated by the Investigator or delegated physician for monitoring cardiac function.

Troponin-T will be measured at screening, EOT, and as needed based on patient-reported cardiac signs or symptoms suggesting congestive heart failure, myocardial infarction, or other causes of cardiac myocyte necrosis.

ECGs will be performed at screening, prior to administration of IMP at Cycle 1 Day 1, and every fourth cycle (once) thereafter. Triplicate ECGs will be performed at screening. Subsequent ECGs will be performed in triplicate only if abnormalities are noted. If 12-lead ECG is abnormal, follow institutional guidelines. Twelve-lead ECGs will be performed, and standard ECG parameters will be measured, including RR, PR, QT intervals, and QRS duration. All ECGs must be evaluated by Investigator or delegated physician for the presence of abnormalities. Whether or not measurement is performed, date performed, results, and findings for each parameter is to be recorded in the eCRF. The summary flow chart for management of LVEF decrease is also available in Appendix L.

8.4.6.6 Specific toxicity management and dose modification information - Dato-DXd

Following the first dose of IP, subsequent administration of Dato-DXd may be modified based on toxicities observed as described in the Guidance for Management of Specific Adverse events in studies of Dato-DXd (Annex document to this CSP). Guidelines for the management of general toxicity, ILD/pneumonitis, combined elevations of aminotransferase and total bilirubin, IRRs including anaphylaxis, and stomatitis/mucosal inflammation that may be attributable to Dato-DXd are also provided in the Annex document to this CSP.

In addition, there are certain circumstances in which Dato-DXd should be permanently discontinued. General guidelines for dose reduction, interruption, and permanent discontinuation are summarized in Section 6.5.2.6. All confirmed or suspected COVID-19 infection events must be recorded in the eCRF as AEs. Please refer to Appendix O for additional information on dose modification.

ILD/pneumonitis management guidance:

All potential ILD/pneumonitis cases should be reported within 24 hours, including both serious and non-serious potential ILD/pneumonitis cases (potential ILD/pneumonitis is described in the Event Adjudication Site Manual). A [redacted] the eCRF to collect relevant [redacted] information for these potential cases regardless of seriousness.

ILD/pneumonitis should be ruled out if a patient develops radiographic changes potentially consistent with ILD/pneumonitis or develops an acute onset of new or worsening pulmonary or other related signs/symptoms such as dyspnea, cough, or fever. If the AE is confirmed to have an etiology other than ILD/pneumonitis, but the AE is assessed as possibly causally related to Dato-DXd, follow the management guidance outlined in the designated “Other Non-Laboratory Adverse Events” dose modification section of the Dato-DXd TMGs (see the Annex document to this CSP).

If the AE is suspected to be ILD/pneumonitis, treatment with study drug should be delayed pending further evaluations. Evaluations should include high resolution CT, pulmonologist

consultation (infectious disease consultation as clinically indicated), blood culture and CBC (other blood tests could be considered as needed), bronchoscopy and bronchoalveolar lavage if clinically indicated and feasible should be considered, PFTs and [redacted] arterial blood gases if clinically indicated, and one blood sample collection for PK analysis as soon as ILD/pneumonitis is suspected, if feasible. Other tests could be considered, as needed (eg, COVID-19 test).

[redacted] for the DS-1082a and Dato-DXd program is responsible for [redacted]. To ensure adequate and relevant [redacted], systematic additional data collection will be conducted for all cases that will be brought [redacted] These additional data collections will cover a more in-depth relevant medical history (eg, smoking, radiation, COPD, and other chronic lung conditions), diagnostic evaluation, treatment, and outcome of the event.

If the AE is confirmed to be ILD/pneumonitis, follow the management guidance outlined in the TMGs (see the Annex document to this CSP). All events of ILD/pneumonitis regardless of severity or seriousness will be followed [redacted] An autopsy in cases of Grade 5 ILD/pneumonitis is encouraged.

Infusion-related reaction management guidance:

All Grade ≥3 events of IRR, regardless of seriousness, must be reported in eCRF within 24 hours.

Premedication is required prior to any dose of Dato-DXd and must include [redacted] and [redacted] with or without [redacted] Refer to the management guidance outlined in the Dato-DXd TMGs (see the Annex document to this CSP).

Stomatitis/mucositis and mucosal inflammation management guidance:

Stomatitis/mucositis and mucosal inflammation events are considered as identified risks. As per Investigator's judgement, a [redacted] before study drug initiation and [redacted], if indicated, may reduce [redacted]

Dato-DXd treatment toxicity management guidelines for Stomatitis/ oral mucositis and mucosal inflammation are provided in the Annex document to this CSP.

Ocular surface events

All Grade ≥ 3 ocular surface events and Grade ≥ 2 keratitis events (including keratitis, punctate keratitis, and ulcerative keratitis) should be reported within 24 hours of becoming aware. All events of ocular surface toxicity and keratitis should be [redacted]

Patients are advised to [redacted] as preventative measure and [redacted] [redacted] as clinically needed and [redacted]. Recommendations for preventing and treating ocular surface toxicity are available in the SoA, Section 8.2.9.2, and the TMGs (see the Annex document to this CSP). See Appendix R for [redacted]

The [redacted] provides assistance to the [redacted] to assess [redacted]. Every outlined [redacted] should be provided in a source document, as per SoA in a timely manner.

8.5 Pharmacokinetics and Immunogenicity

PK and immunogenicity (ADAs) will be assessed only in Part 1 of the study for Arm 1 to Arm 6. Pharmacokinetics and ADAs will be assessed in both Part 1 and Part 2 in Arms 7 and 8.

8.5.1 Collection of samples

8.5.1.1 Collection of samples to measure serum or plasma concentrations

Blood samples for determination of concentrations of durvalumab, oleclumab, and T-DXd (in serum), and capivasertib and Dato-DXd (in plasma), will be obtained according to the SoAs. PK samples will be collected in Part 1 of the study only for Arms 1 to 6 and in both Parts 1 and 2 for Dato-DXd in Arms 7 and 8. As indicated in Table 4, Table 5, and Table 6 additional PK sampling and analyses will be conducted for patients in Arms 6, 7, and 8 in case treatment with chloroquine or hydroxychloroquine for COVID-19 is absolutely required.

Samples for determination of durvalumab and novel oncology therapy concentrations in serum or plasma will be analyzed by AstraZeneca or designated third party laboratories operating on behalf of AstraZeneca. Samples will be collected, labeled, stored, and shipped as detailed in the Laboratory Manual. Full details of the analytical method used will be described in a separate Bioanalytical Validation Report.

8.5.1.2 Collection of samples to measure for the presence of ADAs

The presence of ADA will be assessed in serum samples for durvalumab, oleclumab, and DS-8201a and in plasma samples for capivasertib and Dato-DXd, taken according to the SoAs.

Samples will be measured for the presence of ADAs for durvalumab, oleclumab, DS-8201a, and Dato-DXd (see Section 1.1) using validated assays. ADA samples will be collected in Part 1 of the study only for Arms 1 to 6 and in both Parts 1 and 2 for Dato-DXd in Arms 7 and 8. Tiered analysis will be performed to include screening, confirmatory, and titer assay components. ADA samples may also be [redacted] characterization of the [redacted]

8.5.2 Storage and destruction of pharmacokinetic and ADA samples

PK and ADA samples will be destroyed within 15 years of CSR finalization.

PK and ADA samples may be disposed of or destroyed and anonymized by pooling. Additional analyses may be conducted on the anonymized, pooled PK samples to further evaluate and validate the analytical method. Results from such analyses may be reported separately from the CSR.

Incurred sample reproducibility analysis, if any, will be performed alongside the bioanalysis of the test samples. The results from the evaluation will not be reported in the CSR but separately in a Bioanalytical Validation Report.

Any [redacted] samples may be used for future exploratory [redacted] research (in this case, [redacted] will be shipped to AstraZeneca-assigned [redacted] see details in the Laboratory Manual).

8.6 Pharmacodynamics

Pharmacodynamic parameters are not evaluated in this study.

8.7 [redacted]

8.7.1 Optional exploratory [redacted]

If the patient agrees to participate in the optional [redacted] study, a blood sample will be collected. Participation is optional. Patients who do not wish to participate in [redacted] may still participate in the study.

The blood sample will be obtained from the patients on Day 1 (first infusion day) prior to treatment administration. If for any reason the sample is not drawn on Day 1, it may be taken at any visit until the last study visit. [redacted] should be collected per patient for [redacted] during the study.

In the event of [redacted] or blood sample damage, a replacement [redacted] sample may be requested from the patient. Signed informed consent will be required to obtain a replacement sample unless it was included in the original consent.

See Appendix D for information regarding [redacted]. Details on processes for collection and shipment and destruction of these samples can be found in Appendix D or in the Laboratory Manual.

8.7.2 Storage and destruction of [redacted]

The processes adopted for [redacted] of samples for [redacted] are important to maintain patient confidentiality. Samples may be stored for a maximum [redacted] or per local regulations from the date of the Last Patient's Last Visit, after which they will be destroyed. [redacted] that may be used up during analyses. The results of any further analyses will be reported either in the CSR itself or as an addendum, or separately in a scientific report or publication.

No personal details identifying the individual will be available to AstraZeneca or designated organizations working with the DNA.

8.8 Biomarkers

By participating in this study, the patient consents to the mandatory collection and use of donated biological samples as described here. Tissue samples will be obtained from all screened patients.

Pre-treatment tumor PD-L1 expression will be evaluated in all patients assigned or randomized to treatment. Data will be assessed to determine if baseline PD-L1 status is prognostic and/or predictive of outcomes associated with durvalumab combination therapy. For patients in Arm 8, this data will be used to confirm local PD-L1 test results that supported eligibility. Baseline tumor requirements are briefly described in Section 8.8.1.

Based on availability of tissue, additional exploratory biomarkers may be collected as described in Section 8.8.1 and evaluated as described in Section 8.8.2 (descriptions of exploratory, peripheral measures are described in this section). Samples will be obtained according to the assessment schedules provided in the SoAs.

Details for collection, volumes, storage, and shipment of biologic samples are presented in a separate Laboratory Manual.

All samples collected for biomarker analyses will be stored at the study site, a reference laboratory, or at AstraZeneca facilities and may be used for subsequent research relevant to evaluating biological and/or clinical response to immunotherapy as described in the exploratory analyses section.

The results may be pooled with biomarker data from other studies to evaluate biological responses across indications and to compare results in combination settings.

8.8.1 Collection of patient tumor samples

Samples should not be collected specifically for this study but should be obtained as part of the patient's routine clinical care. Archived tumor specimens are acceptable but should be [redacted] old. Additional details on collection are provided below. Use of collected FFPE tissue samples will not be limited to PD-L1 testing and may be used for other exploratory analysis, as described in Section 8.8.2. Additional, optionally collected tissue samples may also be collected as outlined below for use in exploratory analyses. Provision of an [redacted] is mandatory. A recently acquired sample is preferred if available per site's routine clinical practice. Otherwise, the most recently collected pre-treatment assignment/pre-randomization archival tumor sample ([redacted] prior to enrollment) can be provided. An [redacted] is preferred over sections. If it is not possible to provide a tissue block, [redacted] freshly cut unstained serial tumor sections [redacted] are to be provided (see Laboratory Manual for details). If blocks are incomplete or fewer than [redacted] are available, participants may be eligible [redacted]. Specimens with

limited tumor content, fine needle aspirates and samples obtained from metastatic sites in bone are inadequate for testing and not acceptable. Samples should be collected via an image-guided core needle (at least 18-gauge) or by excision.

The [redacted] will be used for determination of PD-L1 expression and additional exploratory biomarker analyses. The results of these assessments will not be used for inclusion/exclusion or stratification purposes.

- OPTIONAL: The collection of additional archived [redacted] where such samples exist in a quantity sufficient to allow for analysis. [redacted] is preferred. If a [redacted] is unavailable, unstained sections from [redacted] may be submitted. Please consult the Laboratory Manual for specific instructions and guidelines regarding sections.
- OPTIONAL: A newly acquired [redacted] to be collected at [redacted] (Arms 1 to 5) and [redacted] (Arms 6, 7, and 8) for use in [redacted]
- OPTIONAL: The collection of biopsies upon [redacted] of patients in all treatment arms is strongly encouraged.
- OPTIONAL: [redacted] collected as part of clinical care (eg, [redacted]) can be submitted for further analyses.

When tissue is newly obtained by [redacted], effort should be made to maximize material for downstream analyses. Two [redacted] are requested, using an 18-guage or larger needle. These should be placed in formalin and processed to a single paraffin-embedded block, as described in the Laboratory Manual. As a guidance, it is anticipated that 4 passes of an 18-gauge core needle will provide sufficient [redacted] for [redacted] analyses.

See the Laboratory Manual for further details of requirements including sample quality control and shipping.

A brief description of exploratory tumor markers likely to be explored by [redacted] analysis is provided in Section 8.8.2.

8.8.2 Exploratory [redacted]

[redacted] for exploratory biomarker analyses will be obtained according to the schedules presented in the SoAs. Details for collection, volumes, storage, and shipment of biologic samples are presented in a separate Laboratory Manual.

Baseline measures will be correlated with outcomes. Note that samples will be obtained from patients in each treatment arm. Comparisons will be made between baseline measures to determine if biomarkers (or combination of markers) are prognostic or predictive of outcomes.

Exploratory biomarker analyses may include, but are not limited to, the following:

- [redacted]
- Additional [redacted], among other markers, by IHC in FFPE tumors at baseline to evaluate [redacted] in the corresponding treatment arms.
- Explore association of [redacted] such as, but not limited to, [redacted] [redacted] in FFPE tissue.
- [redacted] at baseline and [redacted].
- Explore correlation between [redacted] from FFPE biopsies and [redacted] (eg, for capivasertib and DS-8201: [redacted]
- Explore [redacted] in [redacted], including but not limited to [redacted] between screening/baseline [redacted] as a [redacted]
- Explore association of [redacted] in the blood with [redacted].
- Identify [redacted] of TNBC using [redacted]
- Based on emerging scientific knowledge, further analyses yet to be defined may be undertaken.

Additional sample collections and analyses may be completed at select study sites by site-specific amendment. All samples collected for such exploratory analyses will be stored at the study site, a reference laboratory, or at AstraZeneca's facilities and may be used for subsequent research relevant to evaluating response to immunotherapy.

The exploratory biomarker plan is described by sample type below.

[redacted]

MANDATORY: Whole blood will be collected as indicated in Table 1, Table 2, Table 3, Table 4, Table 5, and Table 6 in [redacted] to characterize baseline [redacted] or that may [redacted] who may [redacted] based on their [redacted].

[redacted]

MANDATORY: Plasma samples will be collected as indicated in Table 1, Table 2, Table 3, Table 4, Table 5, and Table 6 for measurement of baseline [redacted] features that associate with [redacted]

[redacted] to enable future [redacted]. The results of this exploratory research will be reported separately and will not form part of the CSR.

Details of sample collection, processing, shipping, and storage will be described in the Laboratory Manual.

Plasma samples for safety exploratory testing (for patients in Arms 6, 7, and 8 only)

MANDATORY: [redacted] to perform [redacted] or clinical benefit analyses to [redacted] that may [redacted] will be collected from patients in Arms 6, 7, and 8, as described in the SoAs (see Section 1.1). These analyses may include but are not limited to the monitoring of the [redacted] including but not limited to [redacted] and the characterization of the safety profile for patients with [redacted] treated with DS-8201a, Dato-DXd, or chemotherapy compared to the safety profile of patients with [redacted] treated with DS-8201a, Dato-DXd, or chemotherapy.

Management of biomarker data

The biomarker data will have unknown clinical significance. AstraZeneca will not provide [redacted] unless required to do so by law. The patient's samples will not be used for any purpose other than those described in the study protocol.

Individual patients will not be identified in any report or publication resulting from this work. The data and results of this research may be reviewed with collaborators and published, but neither the patient's name nor any other personal identifiers will appear in any publication or report.

Summaries and analyses for exploratory biomarkers will be documented in a separate analysis plan and will be reported outside the CSR in a separate report. The results of this biomarker research may be pooled with biomarker data from other studies to generate hypotheses to be tested in future research.

8.8.3 Storage, re-use, and destruction of biomarker samples

Samples will be stored for a maximum of [redacted] from the end of study, after which they will be destroyed.

8.8.4 Labeling and shipment of biological samples

The Principal Investigator will ensure that samples are labeled and shipped in accordance with the Laboratory Manual and the Biological Substance, Category B, Regulations (materials

containing or suspected to contain infectious substances that do not meet Category A criteria); see Appendix C ("IATA 6.2 Guidance Document").

Any samples identified as Infectious Category A materials will not be shipped, and no further samples will be taken from the involved patients unless agreed upon with AstraZeneca and appropriate labeling, shipment, and containment provisions are approved.

8.8.5 Chain of custody of biological samples

A full chain of custody will be maintained for all samples throughout their life cycle.

The Principal Investigator at each center will keep full traceability of collected biological samples from the patients while in storage at the center until shipment or disposal (where appropriate) and will keep documentation of sample shipments.

The sample receiver will keep full traceability of the samples while in storage and during use until used or disposed of or until further shipment and will keep documentation of sample shipments.

AstraZeneca will keep oversight of the entire life cycle through internal procedures, monitoring of study sites, and auditing or process checks and contractual requirements of external laboratory providers.

Samples retained for further use will be stored in the AstraZeneca-assigned Biobank and will be registered with the AstraZeneca Biobank Team during the entire life cycle.

8.8.6 Withdrawal of informed consent for donated biological samples

If a patient withdraws consent to the use of donated biological samples, the samples will be disposed of or destroyed and the action documented. If samples have already been analyzed, AstraZeneca is not obliged to destroy the results of this research.

The Principal Investigator will:

- Ensure that AstraZeneca is immediately notified of the patients' withdrawal of informed consent to the use of donated samples.
- Ensure that biological samples from that patient, if stored at the study site, are immediately identified, disposed of or destroyed and the action documented.
- Ensure that the organization(s) holding the samples is/are immediately informed about the withdrawn consent and that samples are disposed of or destroyed, the action is documented, and the study site is informed.
- Ensure that the patient and AstraZeneca are informed about the sample disposal.

9 STATISTICAL CONSIDERATIONS

All statistical analyses will be performed by AstraZeneca or its representatives.

A comprehensive SAP will be prepared, with final amendments completed prior to final reporting of the data.

The primary aim of the study is to assess the safety and tolerability profile of durvalumab in combination with paclitaxel and novel oncology therapies and durvalumab in combination with paclitaxel (Part 1) and to assess the efficacy of durvalumab in combination with paclitaxel and 1 or more novel oncology therapies (Part 2) in patients with first-line metastatic TNBC.

Part 1 of this study is considered Stage 1 of the [redacted], and Part 2 of this study is considered Stage 2.

9.1 Sample size determination

Part 1 of this study enrolled [redacted] patients in Arm 1 (now closed to enrollment) and will enroll [redacted] patients in all other treatment arms. Part 2 may enroll a further [redacted] patients in each treatment arm that meets the [redacted] noted below.

The primary objective for Part 1 is safety; [redacted] patients in the durvalumab + paclitaxel arm and [redacted] patients per novel treatment combination arm are considered appropriate to characterize the AE profile of each combination. Twenty patients in the durvalumab + paclitaxel arm (Arm 1) is sufficient to evaluate safety based on data for durvalumab + chemotherapy from other studies. Additional patients [redacted] for novel treatment combination arms is warranted because this study will be the only one to provide data on these novel combinations.

The study is sized to allow the use of a [redacted] for each treatment arm according to the targeted ORR improvement from [redacted] Each treatment arm (with the exception of Arm 1) requires [redacted] response-evaluable patients ([redacted] in Part 1 and [redacted] in Part 2). If at least [redacted] out of [redacted] patients achieve response, then the treatment arm may continue to Part 2; otherwise, further recruitment into this treatment arm will be stopped. If there are at least [redacted] out of [redacted] evaluable patients achieving response in a treatment arm, then the data for that cohort will be considered as having an adequate efficacy signal. Additional information is provided in the SAP.

Additional novel therapy arms may be added at the discretion of the Sponsor.

The final analysis for patients on Arms 1, 2, 5, and 6 will be performed when the last patient has had the opportunity to be followed for approximately 12 months from the date of first

dose. The final analysis for patients on Arm 7 and 8 will be performed when the last patient has had the opportunity to be followed for at least 6 months from the date of first dose.

9.2 Populations for analyses

Definitions of the analysis sets for each outcome variable are provided in Table 27.

Table 27 Summary of outcome variables and analysis populations

Outcome variable	Populations
**Safety data**	
Exposure[a]	Safety Analysis Set
AEs	Safety Analysis Set
Laboratory measurements	Safety Analysis Set
Vital signs	Safety Analysis Set
Physical exams	Safety Analysis Set
ADA[-]	Safety Analysis Set
**Efficacy data**	
ORR	Response Evaluable Analysis Set
DoR, PFS, change in tumor[b] size, PFS6[c], OS	Full Analysis Set (ITT population)
Demography	Full Analysis Set (ITT population)
PK data[b]	PK Analysis Set

[a] This includes durvalumab, paclitaxel, capivasertib, oleclumab, DS-8201a, and Dato-DXd dose delays, reductions, and discontinuations.

[b] Change in tumor size and PK data are applicable for Part 1 only.

[c] PFS6 is applicable for Part 2 only (using pooled data from Part 1 and Part 2 subjects).

[d] Subjects in the Safety Analysis Set (SAF) with a non-missing baseline anti-drug antibody (ADA) result for an agent and at least one non-missing post-baseline ADA result for the same agent will form a subset of the SAF, the ADA-evaluable set.

ADA Anti-drug antibody; AE Adverse event; CI Confidence interval; DoR Duration of response; Dato-DXd Datopotamab deruxtecan; DS-8201a Trastuzumab deruxtecan; FAS Full analysis set; ITT Intent-to-treat; ORR Objective response rate; OS Overall survival; PFS Progression-free survival; PFS6 Progression-free survival at 6 months following date of first dose; PK Pharmacokinetics.

9.2.1 Full Analysis Set

The Full Analysis Set (FAS) will include all patients who are assigned to treatment and received at least 1 dose of study treatment (at least 1 IP [durvalumab, paclitaxel, or novel oncology therapy]). Treatment groups will be summarized according to the treatment received. This is the same as the Safety Analysis Set.

9.2.2 Response Evaluable Analysis Set

A subset of the FAS will include all treated patients who have measurable disease at baseline. This will be the primary analysis set on which ORR analyses will be performed.

9.2.3 Safety Analysis Set

The Safety Analysis Set will consist of all patients who received at least 1 dose of study treatment (at least 1 IP [durvalumab, paclitaxel, or novel oncology therapy]). Safety data will not be formally analyzed but summarized using the SAF according to the treatment received. That is, erroneously treated patients (eg, those assigned/randomized to treatment A but actually given treatment B) will be summarized according to the treatment they actually received.

9.2.4 PK analysis set (Part 1 only in Arms 1 to 6 and Part 2 in Arms 7 and 8)

All patients who received at least 1 dose of durvalumab or novel oncology therapy per protocol and had at least 1 post-dose evaluable PK data point for durvalumab or the novel oncology therapy will be included in the PK analysis set. The population will be defined by AstraZeneca/MedImmune, the Pharmacokineticist, and the Statistician prior to any analyses being performed.

9.3 Outcome measures for analyses

9.3.1 Calculation or derivation of efficacy variables

9.3.1.1 RECIST 1.1-based endpoints

The analysis of the Part 1 key secondary endpoint (ORR) and the analyses of the other secondary endpoints (PFS and DoR) will be based on the site Investigator assessments using RECIST 1.1.

The analysis of the Part 2 primary endpoint (ORR) and the analyses of the secondary endpoints (PFS, DoR, and PFS6) will be based on the site Investigator assessments using RECIST 1.1 using pooled data from Part 1 and Part 2.

RECIST 1.1-based endpoints are derived using confirmed responses. Sensitivity analyses using unconfirmed responses may also be conducted.

9.3.1.1.1 INVESTIGATOR RECIST 1.1-BASED ASSESSMENTS

All RECIST 1.1 assessments, whether scheduled or unscheduled, will be included in the calculations. This is also regardless of whether a patient discontinues study treatment or receives another anticancer therapy, unless otherwise stated for that parameter/analysis.

At each visit, patients will be programmatically assigned a RECIST 1.1 visit response of CR, PR, SD, or PD depending on the status of their disease compared with baseline and previous assessments. Baseline will be assessed within the 28 days prior to enrollment. If a patient has had a tumor assessment that cannot be evaluated, then the patient will be assigned a visit response of not evaluable (unless there is evidence of progression in which case the response will be assigned as PD).

Please refer to Appendix F for the definitions of CR, PR, SD, and PD.

Start date for Time-to-Event endpoints:

The start date for efficacy time-to-event endpoints such as PFS, PFS6, and OS will be the date of first dose of any IP, namely, durvalumab, paclitaxel, or novel oncology therapy. For detailed definitions, see sections below.

9.3.1.2 Objective response rate

In Parts 1 and 2, ORR (per RECIST 1.1 using Investigator assessments) is defined as the number (%) of patients with at least 1 visit response of CR or PR that is subsequently confirmed on another scan. For the CCI [redacted], unconfirmed responses may be considered in decision making.

In both study parts, data obtained up until objective PD or death, or the last evaluable assessment in the absence of progression, will be included in the assessment of ORR. Patients who go off treatment without progression, receive a subsequent therapy, and then respond will not be included as responders in the ORR.

9.3.1.3 PFS

PFS (per RECIST 1.1, as assessed by the site Investigator) will be defined as the time from the date of first dose of IP (at least 1 IP [durvalumab, paclitaxel, or novel oncology therapy]) until the date of objective PD or death (by any cause in the absence of progression) regardless of whether the patient withdraws from therapy or receives another anticancer therapy prior to progression (ie, date of event or censoring – date of randomization/treatment assignment + 1). Patients who have not progressed or died at the time of analysis will be censored at the time of the latest date of assessment from their last evaluable RECIST 1.1 assessment. However, if the patient progresses or dies after 2 or more missed visits, the patient will be censored at the time of the latest evaluable RECIST 1.1 assessment prior to the 2 missed visits. If the patient has no evaluable visits or does not have baseline data, they will be censored at Day 1 unless they die within 2 visits of baseline, then they will be treated as an event with date of death as the event date.

The PFS time will always be derived based on assessment/scan dates and not visit dates.

RECIST 1.1 assessments/scans contributing toward a particular visit may be performed on different dates. The following rules will be applied:

- The date of progression will be determined based on the earliest of the RECIST assessment/scan dates of the component that indicates progression.
- When censoring a patient for PFS, the patient will be censored at the latest of the scan dates contributing to a particular overall visit assessment.

RECIST scans that are reported on the eCRF beyond RECIST 1.1-defined PD will be used for exploratory analyses only.

9.3.1.4 Duration of response

DoR (per RECIST 1.1 using Investigator assessment) will be defined as the time from the date of first documented response (which is subsequently confirmed) until the first date of documented progression or death in the absence of PD (ie, date of PFS event or censoring – date of first response + 1). The end of response should coincide with the date of progression or death from any cause used for the RECIST 1.1 PFS endpoint.

The time of the initial response will be defined as the latest of the dates contributing toward the first visit response of CR or PR. If a patient does not progress following a response, then their DoR will be censored at the PFS censoring time. DoR will not be defined for those patients who do not have documented response.

9.3.1.5 PFS 6 months

The PFS6 will be defined as the Kaplan-Meier estimate of PFS (per RECIST 1.1 as assessed by the site Investigator) at 6 months following date of first dose of IP.

9.3.1.6 Overall survival

OS is defined as the time from the date of first dose of IP until death due to any cause. Any patient not known to have died at the time of analysis will be censored based on the last recorded date on which the patient was known to be alive.

Note: Survival telephone calls will be made following the date of DCO for the analysis (these contacts should generally occur within 7 days of the DCO). If patients are confirmed to be alive or if the death date is post the DCO date, these patients will be censored at the date of DCO for the purposes of the CSR; further follow-up for OS may be conducted following CSR completion and the findings documented separately. Death dates may be found by checking publicly available death registries.

9.3.1.7 Change in tumor size

The best percentage change from pre-dose in tumor size is the largest decrease (or smallest increase) for a patient using RECIST 1.1 assessments. All measurements up until PD or the last evaluable assessment will be included in this evaluation. The percentage change in tumor size at a specific visit may also be presented and will be defined in the SAP.

RECIST scans that are reported on the eCRF beyond RECIST 1.1-defined PD will be used for exploratory analyses only.

9.3.2 Calculation or derivation of safety variables

9.3.2.1 Adverse events

Safety and tolerability will be assessed in terms of AEs (including SAEs), deaths, laboratory findings, vital signs, ECGs, ECHO/MUGA scan, and physical examination findings. These will be collected for all patients. Data from all cycles of treatment will be combined in the presentation of safety data. “On treatment” will be defined as assessments between date of start dose and 90 days following discontinuation of IP (ie, the last dose of durvalumab + paclitaxel combination therapy, durvalumab + paclitaxel + novel oncology therapy combinations, durvalumab + DS-8201a, or durvalumab + Dato-DXd). On-treatment AEs (or treatment-emergent AEs) will be defined as any AEs that started after dosing or that started prior to dosing and worsened following exposure to the treatment.

AEs observed up until 90 days following discontinuation of the last dose of study treatment or until the initiation of the first subsequent therapy following discontinuation of treatment (whichever occurs first) will be used for the reporting of the AE summary tables. This approach will more accurately depict AEs attributable to study treatment only because a number of AEs up to 90 days following discontinuation of study treatment are likely to be attributable to subsequent therapy (if received). However, to assess the longer-term toxicity profile, AE summaries will also be produced containing AEs observed up until 90 days following discontinuation of the study treatment (ie, without taking subsequent therapy into account). Any events in this period that occur after a patient has received further therapy for cancer (following discontinuation of study treatment) will be flagged in the data listings.

The Safety Analysis Set will be used for reporting of safety data.

A separate data listing of AEs occurring more than 90 days after discontinuation of IP will be produced. These events will not be included in AE summaries.

9.3.2.2 Safety assessments

For the change from baseline summaries for vital signs, laboratory data, and physical examinations, the baseline value will be the latest result obtained prior to the start of study treatment.

QTcF will be derived during creation of the reporting database using the reported ECG values (RR and QT) using the following formula:

QTcF = QT/RR^ (1/3) where RR is in seconds

Corrected calcium product will be derived during creation of the reporting database using the following formula:

Corrected calcium (mmol/L) = Total calcium (mmol/L) + ([40 – albumin (G/L)] × 0.02)

The denominator used in laboratory summaries will only include evaluable patients, that is, those who had sufficient data to have the possibility of an abnormality.

For example:

- If a CTCAE criterion involves a change from baseline, evaluable patients would have both a pre-dose and at least 1 post-dose values recorded.
- If a CTCAE criterion does not consider changes from baseline to be evaluable, the patient need only have 1 post-dose value recorded.

The denominator in vital signs data should include only those patients with recorded data.

9.3.3 Calculation or derivation of pharmacokinetic variables (Part 1 only)

PK variables only apply for Part 1 of the study, except for Dato-DXd in Arms 7 and 8.

9.3.3.1 Population pharmacokinetics and exposure-response/safety analysis

A population PK model may be developed using a non-linear mixed-effects modeling approach. The impact of physiologically relevant patient characteristics (covariates) and disease on PK will be evaluated. The relationship between the PK exposure and the effect on safety and efficacy endpoints may be evaluated. The results of such an analysis will be reported in a separate report. The PK, pharmacodynamic, demographic, safety, and efficacy data collected in this study may also be combined with similar data from other studies and explored using population PK and/or PK-pharmacodynamic methods.

9.3.3.2 Pharmacokinetic analysis

PK concentration data and summary statistics will be tabulated. PK parameters will be determined from the raw data. The following PK parameters will be determined after the first and steady-state doses: peak and trough concentrations (as data allow).

9.3.4 Immunogenicity analysis (Part 1 only)

Immunogenicity results will be analyzed descriptively by summarizing the number and percentage of patients who develop detectable ADAs against each applicable novel oncology therapy (see Section 1.1). The immunogenicity titer will be reported for samples confirmed positive for the presence of ADAs. The effect of immunogenicity on PK, pharmacodynamics, efficacy, and safety will be evaluated, if the data allow. ADA samples may also be further tested for characterization of the ADA response

9.3.5 Calculation or derivation of biomarker variables

Biomarker status, as defined in the exploratory objectives, may be assessed for evaluable patients in each cohort according to prespecified criteria that will be detailed in the SAP.

9.3.6 Calculation or derivation of pharmacogenetic variables

In the case of genetic data, only the date that the patient gave consent to participation in the genetic research and the date the blood sample was taken from the patient will be recorded in the eCRF and database. The genetic data generated from the study will be stored in the AstraZeneca Laboratory Information Management System (LIMS) database or other appropriate system. This database is a secure database, which is separate from the database used for the main study. Some or all of the dataset from the main study may be duplicated within the AstraZeneca LIMS database for exploratory [redacted]. Data will be reported outside the CSR (please see Appendix C).

9.4 Statistical analyses

Analyses will be performed by AstraZeneca or its representative. A comprehensive SAP will be developed and finalized before database lock and will describe the patient populations to be included in the analyses and procedures for accounting for missing, unused, and spurious data. This section is a summary of the planned statistical analyses of the primary and secondary endpoints. Any deviations from this plan will be reported in the CSR.

Descriptive statistics will be used for all variables, as appropriate, and will be presented by treatment group. Continuous variables will be summarized by the number of observations, mean, standard deviation, median, minimum, and maximum. Categorical variables will be summarized by frequency counts and percentages for each category. Unless otherwise stated, percentages will be calculated out of the population total for the corresponding treatment arm.

Baseline will be the last assessment of the variable under consideration prior to the intake of the first dose of IP, and the same holds for efficacy variables.

It is anticipated that data from Arm 2 will be presented as a single cohort. In the event potentially material differences are observed in either safety or efficacy outcomes between the groups of patients recruited into Arm 2 following CSP Version 3/Version 4 and those

recruited to a later version, further exploratory data presentations may be generated for these patients. For Arm 2, patients recruited following CSP Version 4 or beyond will be evaluated for ORR as per the [redacted] design.

9.4.1 Safety analyses

Safety is the primary objective for Part 1 and the secondary objective for Part 2.

9.4.1.1 Safety analyses for Part 1

Safety and tolerability data will be presented by treatment arm using the safety population.

Data from all cycles of treatment will be combined in the presentation of safety data.

AEs (both in terms of MedDRA preferred terms and CTCAE grade) will be listed individually by patient. The number of patients experiencing each AE will be summarized by treatment arm and CTCAE grade. Additionally, data presentations of the rate of AEs per person-years at risk may be produced.

Other safety data will be assessed in terms of clinical chemistry, hematology, and vital signs and summarized using appropriate summary statistics. Exposure to durvalumab + paclitaxel combination therapy and durvalumab + paclitaxel + novel oncology therapy with or without paclitaxel will be summarized. Time on study; time on the combination therapy; and (for applicable treatments) dose delays, dose interruptions, and dose reductions will also be summarized. At the end of the study, appropriate summaries of all safety data will be produced, as defined in the SAP.

9.4.1.2 Safety analyses for Part 2

The Safety Analysis Set will be used to summarize all safety data according to the treatment received. Analyses of AEs and other safety data will be the same as those described for Part 1. Safety analyses will be based on data pooled from Part 1 and Part 2 in each treatment arm.

9.4.2 Efficacy analyses

9.4.2.1 Planned Analyses

Efficacy will be assessed in terms of ORR, PFS, DoR, OS, and PFS6 based on patients pooled from Part 1 and Part 2 in each treatment arm (with the exception of Arm 1). Efficacy data will also be explored in terms of [redacted] status. Results of ORR statistical analysis will be presented using 95% exact Clopper-Pearson CIs.

Table 28 details which endpoints are to be summarized.

Table 28 Pre-planned statistical and sensitivity analyses to be conducted

Endpoints analyzed	Notes
Safety	Summary statistics for AEs, exposure, laboratory findings, and vital signs:<br>Primary analysis for Part 1 for the safety population
Objective response rate	Number and percentage of patients who achieve confirmed ORR as determined by the Investigator according to RECIST 1.1 in each cohort are summarized with 95% Clopper-Pearson confidence intervals for:<br>Key secondary analysis for Part 1 and primary analysis for Part 2 for the FAS population using Investigator RECIST 1.1 assessments
Duration of response	Summary statistics and KM plot by cohort for:<br>Secondary analysis for the FAS population using Investigator RECIST 1.1 assessments
Progression-free survival	Summary statistics and KM plot by cohort for:<br>Secondary analysis for the FAS population using Investigator RECIST 1.1 assessments
PFS6	The PFS6, along with its 95% CI, will be summarized (using the Kaplan-Meier curve) and presented by treatment arm.<br>Secondary analysis for the FAS population
Overall survival	Summary statistics and KM plot by cohort for:<br>Secondary analysis for the FAS population

AE Adverse event; FAS Full Analysis Set; KM Kaplan-Meier; ORR Objective response rate.

9.4.2.1.1 OBJECTIVE RESPONSE RATE

The ORR will be based on the programmatically derived RECIST 1.1 result using the Investigator-assessed tumor data. The ORR and 95% exact Clopper-Pearson CIs for each treatment arm will be utilized to enable decisions to be made regarding whether to expand the cohort with an additional [redacted] subjects.

Summaries will be produced that present the number and percentage of patients with a tumor response (CR/PR). Overall visit response data will be listed for all patients in the FAS. For each treatment arm, best overall response will be summarized.

9.4.2.1.2 PROGRESSION-FREE SURVIVAL, DURATION OF RESPONSE, AND OVERALL SURVIVAL

PFS and DoR will be derived for each treatment arm based on the Investigator tumor data. Descriptive data will be provided by cohort for PFS, DoR in responding patients, and OS, including the associated Kaplan-Meier curves (without any formal comparison of treatment arms or p-value attached).

9.4.2.1.3 PERCENTAGE CHANGE IN TUMOR SIZE

The absolute value, change from baseline, and percentage change from baseline in tumor size will be summarized using descriptive statistics and presented by actual treatment group.

9.4.2.2 Exploratory analyses to be conducted if deemed necessary

[redacted]

[redacted]

Subgroup analyses may be conducted for ORR and PFS (per RECIST 1.1 using Investigator assessments) within and between novel treatment combination arms in the following subgroups of the FAS (but not limited to the following subgroups):

- Age at treatment assignment/randomization (<65 versus ≥65 years of age)
- [redacted]
- Race (Asian versus non-Asian)
- Visceral metastases
- Time since completion of last chemotherapy
- BRCA1/2 mutation

Other baseline variables may also be assessed if there is clinical justification. The purpose of the subgroup analyses is to assess the consistency of treatment effect across expected prognostic and/or predictive factors. A forest plot may also be presented.

If there are too few events available for a meaningful analysis of a particular subgroup (it is not considered appropriate to present analyses where there are [redacted]), the relationship between that subgroup and ORR or PFS will not be formally analyzed. In this case, only descriptive summaries may be provided.

9.4.3 Pharmacokinetic data

PK concentration data, collected in Part 1 only for Arms 1 to 6 and Parts 1 and 2 for Arms 7 and 8, will be listed for each patient and each dosing day, and a summary will be tabulated and/or graphed for all evaluable patients. PK data collected in this study may be utilised for

population PK and exposure/response analysis. The population PK analysis and exposure/response analyses will be presented separately from the main CSR.

9.4.4 Pharmacokinetic/pharmacodynamic relationships

If the data are suitable, the relationship between PK exposure and efficacy/safety parameters may be investigated graphically or using an appropriate data modeling approach.

9.4.5 Biomarker data

Summaries and analyses for exploratory biomarkers may be documented in a separate analysis plan and may be reported outside the CSR in a separate report.

9.4.6 Methods for multiplicity control

No adjustment for multiplicity is required for between-treatment-arm comparison, as each arm is being evaluated independently. For testing ORR within each arm at the [redacted], the Type I error rate is controlled at the 5% significance level.

9.5 [redacted] analyses

The safety of all AstraZeneca clinical studies is closely monitored on an ongoing basis by AstraZeneca representatives in consultation with Patient Safety. Issues identified will be addressed; for instance, this could involve amendments to the study protocol and letters to Investigators.

An SRC will review the initial safety profile from the novel treatment combination arms in Part 1 after [redacted] DLT-evaluable patients have completed the first cycle or had a DLT during the first cycle and will review safety and tolerability data on an ongoing basis. Additional details on the use of the SRC during the Part 1 safety run-in period are provided in Section 4.1.4.3. An SRC will continue to be used throughout Part 1 and in Part 2 of the study, as appropriate.

At the end of Part 1, there will be a planned interim analysis for futility. The [redacted] will be applied to each cohort independently [redacted]

During Part 1, analyses will also be conducted by AstraZeneca in each cohort after approximately the [redacted] patient (durvalumab + paclitaxel arm) or 30th patient (novel treatment combination arms) who received at least 1 dose of study treatment (ie, evaluable patient) has had the opportunity for at least 2 on-treatment RECIST assessments or has discontinued due to PD or death prior to the second on-study scan. Study continuation onto Part 2 will depend on the ORR data for the first [redacted] evaluable patients in Part 1 for each cohort. Details of these analyses will be provided in the SAP.

10 REFERENCES

Adams et al 2016
Adams S, Diamond JR, Hamilton EP, Pohlmann PR, Tolaney SM, Molinero L, et al. A Phase 1b trial of atezolizumab in combination with nab-paclitaxel in patients with metastatic triple negative breast cancer (mTNBC). J Clin Oncol 2016;34(Suppl):abstr 1009.

Alexandrov et al 2013
Alexandrov LB, Nik-Zainal S, Wedge DC, Aparicio SAJR, Behjati S, Blankin AV, et al. Signatures of mutational processes in human cancer. Nature 2013;500:415-21.

Altomare et al 2005
Altomare DA, Testa JR. Perturbations of the AKT signaling pathway in human cancer. Oncogene 2005;24(50):7455-64.

Antonioli et al 2013
Antonioli L, Blandazzi C, Pacher P, Hasko G. Immunity, inflammation and cancer: a leading role for adenosine. Nature Rev Cancer 2013;13:842-57.

Ambrogi et al 2014
Ambrogi F, Fornili M, Boracchi P, Trerotola M, Relli V, Simeone P, et al. Trop-2 is a determinant of breast cancer survival. PLoS One. 2014 May 13;9(5):e96993. doi: 10.1371/journal.pone.0096993. Erratum in: PLoS One. 2014;9(10):e110606.

Banerji et al 2017
Banerji U, Dean E, Pérez-Fidalgo JA, Batist G, Bedard PL, You B, et al. A Phase 1 open-label study to identify a dosing regimen of the pan-AKT inhibitor AZD5363 for evaluation in solid tumors and in PIK3CA-mutated breast and gynecologic cancers. Clin Cancer Res 2018;24(9):2050-9.

Bardia et al 2017
Bardia A, Mayer IA, Diamond JR, Moroose RL, Isakoff SJ, Starodub AN, et al. Efficacy and safety of anti-Trop-2 antibody drug conjugate Sacituzumab Govitecan (IMMU-132) in heavily pretreated patients with metastatic triple-negative breast cancer. J Clin Oncol 2017;35(19):2141-48.

Bardia et al 2019
Bardia A, Mayer IA, Vahdat LT, Tolaney SM, Isakoff SJ, Diamond JR, et al. Sacituzumab Govitecan-hziy in refractory metastatic triple-negative breast cancer. N Engl J Med 2019;380:741-51.

Bardia et al 2020
Bardia A, Tolaney SM, Loirat D, Punie K, Oliveira M, Rugo HS, et al. LBA17 ASCENT: A randomized Phase III study of Sacituzumab Govitecan (SG) vs treatment of physician's choice (TPC) in patients (pts) with previously treated metastatic triple-negative breast cancer (mTNBC). Annals of Oncology (2020) 31 (suppl_4): S1142-S1215. 10.1016/annonc/annonc325.

Bardia 2021
Bardia A. LBA4-Datopotamab derutecan (Dato-DXd), a TROP2-directed antibody-drug conjugate (ADC), for triple-negative breast cancer (TNBC): preliminary results from an ongoing phase 1 trial. Annals of Oncology (2021) 32 (suppl_2): S60-S78. 10.1016/annonc/annonc508.

Brahmer et al 2012
Brahmer JR, Tykodi SS, Chow LQM, Hwu WJ, Topalian SL, Hwu P, et al. Safety and activity of anti-PD-L1 antibody in patients with advanced cancer. N Engl J Med 2012;366(26):2455-65.

Buisseret et al 2017
Buisseret L, Pommey S, Allard B, Garaud S, Bergeron M, Cousineau I, et al. Clinical significance of CD73 in triple-negative breast cancer: multiplex analysis of a phase III clinical trial. Ann Oncol 2017. doi: 10.1093/annonc/mdx730. [Epub ahead of print].

Cancer Genome Atlas Network 2012
The Cancer Genome Atlast Network. Comprehensive molecular portraits of human breast tumours. Nature 2012;490:61-70.

Cardillo et al 2015
Cardillo TM, Govindan SV, Sharkey RM, Trisal P, Arrojo R, Liu D, et al. Sacituzumab govitecan (IMMU-132), an anti-Trop-2/SN-38 antibody-drug conjugate: characterization and efficacy in pancreatic, gastric, and other cancers. Bioconjug Chem. 2015 May 20;26(5):919-31.

Cardillo et al 2020
Cardillo TM, Rossi DL, Zalath MB, Liu D, Arrojo R, Sharkey RM, et al. Predictive biomarkers for sacituzumab govitecan efficacy in Trop-2-expressing triple-negative breast cancer. Oncotarget. 2020 Oct 27;11(43):3849-62.

Chang et al 2004
Chang L, Chiang SH, Saltiel AR. Insulin signaling and the regulation of glucose transport. Mol Med 2004;10(7-12):65-71.

Ciriello et al 2015
Ciriello G, Gatza ML, Beck AH, Wilkerson MD, Rhie SK, Pastore A, et al. Comprehensive molecular portraits of invasive lobular breast cancer. Cell 2015;163:506-19.

Curtis et al 2012
Curtis C, Shah SP, Chin S-F, Turashvili G, Rueda OM, Dunning MJ, et al. The genomic transcriptomic architecture of 2,000 breast tumours reveals novel subgroups. Nature 2012;486(7403):346-52.

Davies et al 2012
Davies BR, Greenwood H, Dudley P, Crafter C, Yu DH, Zhang J, et al. Preclinical pharmacology of AZD5363, an inhibitor of AKT: pharmacodynamics, antitumor activity, and correlation of monotherapy activity with genetic background. Mol Cancer Ther 2012;11(4):873–87.

Demaria et al 2001
Demaria S, Volm MD, Shapiro RL, Yee HT, Oratz R, Formenti SC, et al. Development of tumor-infiltrating lymphocytes in breast cancer after neoadjuvant paclitaxel chemotherapy. Clin Cancer Res 2001;7(10):3025-30.

Desantis et al 2017
DeSantis CD, Ma J, Sauer AG, Newman LA, Jemal A. Breast cancer statistics, 2017, racial disparity in mortality by state. CA Cancer J Clin 2017;67:439-48.

Dunn et al 2004
Dunn GP, Old LJ, Schreiber RD. The three Es of cancer immunoediting. Annu Rev Immunol 2004;22:329-60.

Emens et al 2015
Emens LA, Braiteh FS, Cassier P, Delord JP, Eder JP, Shen X, et al. Inhibition of PD-L1 by MPDL3280A leads to clinical activity in patients with metastatic triple-negative breast cancer. Cancer Res 2015;75(Suppl 9);abstr PD1-6.

FDA Guidance 2007
Food and Drug Administration. Guidance for industry: toxicity grading scale for healthy adult and adolescent volunteers enrolled in preventive vaccine clinical trials [2007 Sep]. Available from: URL: https://www.fda.gov/downloads/BiologicsBloodVaccines/GuidanceComplianceRegulatoryInformation/Guidances/Vaccines/UCM091977.pdf.

FDA Guidance 2011
Food and Drug Administration. Guidance for industry: clinical trial endpoints for the approval of non-small cell lung cancer drugs and biologics [2011 Jun]. Available from: URL: http://www.fda.gov/downloads/Drugs/Guidances/UCM259421.pdf. Accessed 21 May 2014.

Ganpenrieder et al 2017
Ganpenrieder SP, Rinnerthaler G, and Greil R. SABCS 2016: systemic therapy for metastatic breast cancer. Memo 2017;10:86-89.

GLOBOCAN 2012
GLOBOCAN 2012: Lung cancer estimated incidence, mortality, and prevalence worldwide in 2012. Available from URL: http://globocan.iarc.fr/factsheets/cancers/lung.asp. Accessed 01 February 2018.

Goldenberg et al 2015.
Goldenberg DM, Cardillo TM, Govindan SV, Rossi EA, Sharkey RM. Trop-2 is a novel target for solid cancer therapy with sacituzumab govitecan (IMMU-132), an antibody-drug conjugate (ADC). Oncotarget. 2015 Sep 8;6(26):22496-512. doi: 10.18632/oncotarget.4318. Erratum in: Oncotarget. 2020 Mar 10;11(10):942.

Goldenberg et al 2018
Goldenberg DM, Stein R, Sharkey RM. The emergence of trophoblast cell-surface antigen 2 (TROP-2) as a novel cancer target. Oncotarget. 2018;9(48):28989-006.

Goldenberg et al 2020
Goldenberg DM, Cardillo TM, Govindan SV, Rossi EA, Sharkey RM. Trop-2 is a novel target for solid cancer therapy with sacituzumab govitecan (IMMU-132), an antibody-drug conjugate (ADC). Oncotarget. 2015 Sep 8;6(26):22496-512. doi: 10.18632/oncotarget.4318. Erratum in: Oncotarget. 2020 Mar 10;11(10):942.

Hammond et al 2010
Hammond MEH, Hayes DF, Dowsett M, Allred DC, Hagerty KL, Bavde S, et al. American Society of Clinical Oncology/College of American Pathologists Guideline Recommendations for Immunohistochemical Testing of Estrogen and Progesterone Receptors in Breast Cancer (Unabridged Version). Arch Pathol Lab Med 2010;134:e48-e72.

Heery et al 2017
Heery CR, O'Sullivan-Coyne G, Madan RA, Cordes L, Rajan A, Rauckhorst M, et al. Avelumab for metastatic or locally advanced previously treated solid tumours (JAVELIN Solid Tumor): A phase 1a, multicohort, dose-escalation trial. Lancet Oncol 2017;18:587-98.

Hirano et al 2005
Hirano F, Kaneko K, Tamura H, Dong H, Wang S, Ichikawa M, et al. Blockade of B7-H1 and PD-1 by monoclonal antibodies potentiates cancer therapeutic immunity. Cancer Res 2005;65(3):1089-96.

Hu et al 2015
Hu XC, Zhang J, Xu BH, Cai L, Ragaz J, Wang ZH, et al. Cisplatin plus gemcitabine versus paclitaxel plus gemcitabine as first-line therapy for metastatic triplenegative breast cancer (CBCSG006): A randomised, open-label, multicentre, phase 3 trial. Lancet Oncol 2015;16:436-46.

Huang et al 2005
Huang H, Groth J, Sossey-Alaoui K, Hawthorn L, Beall S, Geradts J. Aberrant expression of novel and previously described cell membrane markers in human breast cancer cell lines and tumors. Clin Cancer Res. 2005 Jun 15;11(12):4357-64. doi: 10.1158/1078-0432.CCR-04-2107.

Hudis and Gianni 2011
Hudis CA, Gianni L. Triple-negative breast cancer: An unmet medical need. Oncologist 2011;16(Suppl 1):1–11.

Isakoff et al 2015
Isakoff SJ, Mayer EL, He L, Traina TA, Carey LA, Krag KJ, et al. TBCRC009: A multicenter phase II clinical trial of platinum monotherapy with biomarker assessment in metastatic triple-negative breast cancer. J Clin Oncol 2015;33:1902–9.

Iwai et al 2002
Iwai Y, Ishida M, Tanaka Y, Okazaki T, Honjo T, Minato N. Involvement of PD-L1 on tumor cells in the escape from host immune system and tumor immunotherapy by PD-L1 blockade. Proc Natl Acad Sci USA 2002;99:12293-7.

Iwai et al 2018
Iwai T, Sugimoto M, Wakita D, Yorozu K, Kurasawa M, Yamamoto K. Topoisomerase I inhibitor, irinotecan, depletes regulatory T cells and up-regulates MHC class I and PD-L1 expression, resulting in a supra-additive antitumor effect when combined with anti-PD-L1 antibodies. Oncotarget. 2018 Jul 31;9(59):31411-21. doi: 10.18632/oncotarget.25830.

Jin et al 2010
Jin D, Fan J, Wang L, Thompson LF, Liu A, Daniel BJ, et al. CD73 on tumor cells impairs antitumor T-cell responses: A novel mechanism of tumor-induced immune suppression. Cancer Res 2010;70(6):2245-55.

Kalinsky et al 2020
Kalinsky K, Diamond JR, Vahdat LT, Tolaney SM, Juric D, O'Shaughnessy J, et al. Sacituzumab govitecan in previously treated hormone receptor-positive/HER2-negative metastatic breast cancer: final results from a phase I/II, single-arm, basket trial. Annals of Oncology 2020;31(12):1709-18.

Karnoub and Weinberg 2008
Karnoub AE, Weinberg RA. Ras oncogenes: split personalities. Nat Rev Mol Cell Biol 2008;9(7):517-31.

Keir et al 2008
Keir ME, Butte MJ, Freeman GJ, Sharpe AH. PD-1 and its ligands in tolerance and immunity. Annu Rev Immunol 2008;26:677-704.

Kempe et al 2010
Kempe DS, Siraskar G, Fröhlich H, Umbach AT, Stübs M, Weiss F, et al. Regulation of renal tubular glucose reabsorption by Akt2/PKBβ. Am J Physiol Renal Physiol 2010;298(5):F1113-7.

Kim et al 2017
Kim S-B, Dent R, Im S-A, Espié M, Blau S, Tan AR, et al. Ipatasertib plus paclitaxel versus placebo plus paclitaxel as first-line therapy for metastatic triple-negative breast cancer (LOTUS): a multicentre, randomised, double-blind, placebo-controlled, phase 2 trial. Lancet Oncol 2017:18(10):1360-72.

Lin et al 2013
Lin H, Huang JF, Qiu JR, Zhang HL, Tang XJ, Li H, et al. Significantly upregulated TACSTD2 and cyclin D1 correlate with poor prognosis of invasive ductal breast cancer. Exp Mol Pathol. 2013 Feb;94(1):73-8.

Lisberg et al 2020
Lisberg AE, Sands J, Shimizu T, et al. Dose escalation and expansion from the phase 1 study of DS 1062, a trophoblast cell-surface antigen 2 (TROP2) antibody drug conjugate, in patients with advanced non-small cell lung cancer. Poster presented at: American Society of Clinical Oncology; May 29-May 31, 2020.

LoRusso 2016
LoRusso PM. Inhibition of the P13K/AKT/mTOR pathway in solid tumors. J Clin Oncol 2016;34(31):3803-15.

Loi et al 2013
Loi S, Pommey S, Haibe-Kain B, Beavis P, Darcy PK, Smyth MJ, et al. CD73 promotes anthracycline resistance and poor prognosis in triple negative breast cancer. Proc Natl Acad Sci USA 2013;110(27):11091-6.

Loi et al 2016
Loi S, Dushyanthen S, Beavis PA, Salgado R, Denkert C, Savas P, et al. Clin Cancer Res 2016;22(6):1499-509. doi: 10.1158/1078-0432.CCR-15-1125. Epub 2015 Oct 29.

Marfella et al 2001
Marfella R, Rossi F, Giugliano D. Hyperglycemia and QT interval: time for re-evaluation. Diabetes Nutr Metab 2001;14(2):63-5.

Mauri et al 2010
Mauri D, Kamposioras K, Tsali L, Bristianou M, Valachis A, Karathanasi I, et al. Overall survival benefit for weekly vs. three-weekly taxanes regimens in advanced breast cancer: A meta-analysis. Cancer Treat Rev 2010;36(1):69-74.

McCubrey et al 2006
McCubrey JA, Steelman LS, Abrams SL, Lee JT, Chang F, Bertrand FE, et al. Roles of the RAF/MEK/ERK and PI3K/PTEN/AKT pathways in malignant transformation and drug resistance. Adv Enzyme Regul 2006;46:249-79.

Millis et al 2015
Millis SZ, Gatalica Z, Winkler J, Vranic S, Kimbrough J, Reddy S, et al. Predictive biomarker profiling of >6000 breast cancer patients shows heterogeneity in TNBC, with treatment implications. Clin Breast Cancer 2015;15(6):473-81.

Nanda et al 2016
Nanda R, Chow LQ, Dees EC, Berger R, Gupta R, Geva R, et al. Pembrolizumab in patients with advanced triple-negative breast cancer: Phase 1b KEYNOTE-012 study. J Clin Oncol 2016;34:2460-7.

NCCN Guidelines V3.2019
National Comprehensive Cancer Network Clinical Practice Guidelines in Oncology – Breast Cancer. Version 3.2019. Available at: https://www.nccn.org/professionals/physician_gls/pdf/breast.pdf.

Noone et al 2017
Noone AM, Cronin KA, Altekruse SF, Howlader N, Lewis DR, Petkov VI, Penberthy L. Cancer incidence and survival trends by subtype using data from the surveillance epidemiology and end results program, 1992-2013. Cancer Epidemiol Biomarkers Prev 2017;26(4):632-641. doi: 10.1158/1055-9965.EPI-16-0520. Epub 2016 Dec 12.

Ogitani et al 2016
Ogitani Y, Hagihara K, Oitate M, Naito H, Agatsuma T. Bystander killing effect of DS-8201a, a novel anti-human epidermal growth factor receptor 2 antibody-drug conjugate, in tumors with human epidermal growth factor receptor 2 heterogeneity. Cancer Sci. 2016;107(7):1039-46.

Okajima et al 2018
Okajima D, Yasuda S, Yokouchi Y, Fujitani T, Sakurai K, Yamaguchi J, et al. Preclinical efficacy studies of DS-1062a, a novel TROP2-targeting antibody-drug conjugate with a novel DNA topoisomerase I inhibitor DXd. J Clin Oncol 2018 36:15_suppl, e24206-e24206.

Okazaki and Honjo 2007
Okazaki T, Honjo T. PD-1 and PD-1 ligands: from discovery to clinical application. Int Immunol 2007;19(7):813-24.

Okudaira et al 2009
Okudaira K, Hokari R, Tsuzuki Y, Okada Y, Komoto S, Watanabe C, et al. Blockade of B7-H1 or B7-DC induces an antitumor effect in a mouse pancreatic cancer model. Int J Oncol 2009;35(4):741-9.

Pardoll 2012
Pardoll DM. The blockade of immune checkpoints in cancer immunotherapy. Nat Rev Cancer 2012;12:252-64.

Pazdur 2008
Pazdur R. Endpoints for assessing drug activity in clinical trials. Oncologist 2008;13(Suppl 2):19-21.

Pereira et al 2016
Pereira B, Chin SF, Rueda OM, Vollan HKM, Provenzano E, Bardwell HA, et al. The somatic mutation profiles of 2,433 breast cancers refine their genomic and transcriptomic landscapes. Nat Commun 2016;7(11479):1-16.

Powles et al 2014
Powles T, Eder JP, Fine GD, Braiteh FS, Loriot Y, Cruz C, et al. MPDL3280A (anti-PD-L1) treatment leads to clinical activity in metastatic bladder cancer. Nature 2014;515(7528):558-62.

Qin et al 2016
Mechanisms of immune evasion and current status of checkpoint inhibitors in non-small cell lung cancer. Cancer Med 2016; 9:2169–70.

Rizvi et al 2015
Rizvi N, Brahmer J, Ou S-H, Segal NH, Khleif SN, Hwu WJ. Safety and clinical activity of MEDI4736, an anti-programmed cell death-ligand-1 (PD-L1) antibody, in patients with nonsmall cell lung cancer (NSCLC). J Clin Oncol 2015;33:Abstract 8032.

Robson et al 2017
Robson M, Im S, Senkus E, Xu B, Domchek SM, Masuda N. Olaparib for metastatic breast cancer in patients with a germline BRCA mutation. N Engl J Med 2017;377:523-33.

Samanta et al 2018
Samanta D, Park Y, Ni X, Li H, Zahnow CA, Gabrielson E, et al. Chemotherapy induces enrichment of CD47+/CD73+/PDL1+ immune evasive triple-negative breast cancer cells. PNAS 2018; [epub ahead of print]. https://doi.org/10.1073/pnas.1718197115.

Savas et al 2016
Savas P, Salgado R, Denkert C, Sotiriou C, Darcy PK, Smyth MJ, Loi S. Clinical relevance of host immunity in breast cancer: from TILs to the clinic. Nat Rev Clin Oncol 2016;13(4):228-41.

Schmid et al 2018a
Schmid P, Cortes J, Bergh JCS, Pusztai L, Denkert C, Verma S, et al. KEYNOTE-522: Phase III study of pembrolizumab (pembro) + chemotherapy (chemo) vs placebo + chemo as neoadjuvant therapy followed by pembro vs placebo as adjuvant therapy for triple-negative breast cancer (TNBC). J Clin Oncol 2018;36(15_suppl):1007.

Schmid et al 2018b
Schmid P, Adams S, Rugo HS, Schneeweiss A, Barrios CH, Iwata H, et al. Atezolizumab and nab-paclitaxel in advanced triple-negative breast cancer. N Engl J Med 2018;379(22):2108-21.

Segal et al 2015
Segal NH, Ou S-HI, Balmanoukian AS, Fury MG, Massarelli E, Brahmer JR, et al. Safety and efficacy of MEDI4736, an anti-PD-L1 antibody, in patients from a squamous cell carcinoma of the head and neck (SCCHN) expansion cohort. J Clin Oncol 2015;33:Abstract 3011.

Shah et al 2012
Shah SP, Roth A, Goya R, Oloumi A, Ha G, Zhao Y, et al. The clonal and mutational evolution spectrum of the primary triple-negative breast cancers. Nature 2012;486(7403):395-6.

CCI [redacted]

Son et al 2018
Son S, Shin S, Rao NV, et al. Anti-Trop2 antibody-conjugated bioreducible nanoparticles for targeted triple negative breast cancer therapy. Int J Biol Macromol. 2018;110:406-15.

Stagg et al 2010
Stagg J, Divisekera U, McLaughlin N, Sharkey J, Pommey S, Denoyer D, et al. Anti-CD73 antibody therapy inhibits breast tumor growth and metastasis. PNAS 2010;107(4):1547-52.

Stagg et al 2011
Stagg J, Divisekera U, Duret H, Sparwasser T, Teng MWL, Darcy PK, et al. CD73-deficient mice have increased anti-tumor immunity and are resistant to experimental metastasis. Cancer Res 2011;71(8):2892-900.

Stewart et al 2015
Stewart R, Morrow M, Hammond SA, Mulgrew K, Marcus D, Poon E, et al. Identification and characterization of MEDI4736, an antagonistic anti-PD-L1 monoclonal antibody. Cancer Immunol Res 2015;3(9):1052-62.

Sun and Chen 2010
Sun X, Chen C: Comparison of Finkelstein's method with the conventional approach for interval-censored data analysis. Stat Biopharm Research 2010;2:97-108.

Tolaney et al 2016
Tolaney S, Savulsky C, Aktan G, Xing D, Almonte A, Karantza V, et al. Phase 1b/2 study to evaluated eribulin mesylate in combination with pembrolizumab in patients with metastatic triple-negative breast cancer. Presented at the 2016 San Antonio Breast Cancer Symposium; December 6-10, 2016, San Antonio, TX; abstr P5-15-02.

Topalian et al 2012
Topalian SL, Hodi FS, Brahmer JR, Gettinger SN, Smith DC, McDermott DF, et al. Safety, activity, and immune correlates of anti-PD-1 antibody in cancer. N Engl J Med 2012;366:2443-54.

Trerotola et al 2013
Trerotola M, Cantanelli P, Guerra E, Tripaldi R, Aloisi AL, Bonasera V, et al. Upregulation of Trop-2 quantitatively stimulates human cancer growth. Oncogene 2013;32:222-33.

TRODELVY
US Product Label for TRODELVY (sacituzumab govitecan-hziy) for injection, for intravenous use (Initial US Approval: 2020), available at: https://www.accessdata.fda.gov/drugsatfda_docs/label/2020/761115s000lbl.pdf.

Turner et al 2017
Turner N, Alarcón E, Armstrong A, Philco M, López Chuken YA, Sablin M-P, et al. BEECH: A randomised Phase 2 study assessing the efficacy of AKT inhibitor AZD5363 combined with paclitaxel in patients with ER1ve advanced or metastatic breast cancer, and in a PIK3CA mutant subpopulation [Abstract 241PD]. Ann Oncol 2017;28(suppl 5):v74-v108.

van Noord et al 2010
van Noord C, Sturkenboom MC, Straus SM, Hofman A, Kors JA, Witteman JC, et al. Serum glucose and insulin are associated with QTc and RR intervals in nondiabetic elderly. Eur J Endocrinol 2010;162(2):241-8.

von Lintig et al 2000
von Lintig FC, Dreilinger AD, Varki NM, Wallace AM, Casteel DE, Boss GR. Ras activation in human breast cancer. Breast Cancer Res Treat 2000;62(1):51-62.

Wang et al 2009
Wang DD, Zhang S, Zhao H, Men AY, Parivar K. Fixed dosing versus body size based dosing of monoclonal antibodies in adult clinical trials. J Clin Pharmacol 2009;49(9):1012-24.

Wilson et al 2019
Wilson TR, Udyavar AR, Chang C-W, Spoerke JM, Aimi J, Savage HM, et al. Genomic alterations associated with recurrence and TNBC subtype in high-risk early breast cancers. Mol Cancer Res 2019;17(1):97-108.

Wolff et al 2018
Wolff AC, Hammond EH, Allison KH, Harvey BE, Mangu PB, Bartlett JMS, et al. Human epidermal growth factor receptor 2 testing in breast cancer. Arch Pathol Lab Med 2018;142:1364-82.

Young et al 2016
Young A, Ngiow SF, Barkauskas DS, Sult E, Hay C, Blake SJ, et al. Co-inhibiton of CD73 and A2AR adenosine signaling improves anti-tumor immune responses. Cancer Cell 2016;30(3):391-403.

Zhang et al 2008
Zhang C, Wu S, Xue X, Li M, Qin X, Li W, et al. Antitumor immunotherapy by blockade of the PD-1/PD-L1 pathway with recombinant human PD-1-IgV. Cytotherapy 2008;10(7):711-9.

Zhao et al 2018
Zhao W, Kuai X, Zhou X, Jia L, Wang J, Yang X, et al. Trop2 is a potential biomarker for the promotion of EMT in human breast cancer. Oncol Rep. 2018 Aug;40(2):759-66.

Zhao et al 2019
Zhao W, Jia L, Zhang M, Huang X, Qian P, Tang Q, et al. The killing effect of novel bi-specific Trop2/PD-L1 CAR-T cell targeted gastric cancer. Am J Cancer Res. 2019 Aug 1;9(8):1846-56.

Zhi et al 2007
Zhi X, Chen S, Zhou P, Shao Z, Wang L, Zhoulou O, et al. RNA interference of ecto-5’-nucelotidase (CD73) inhibits human breast cancer cell growth and invasion. Clin Exp Metastasis 2007;24:439-48.

11 SUPPORTING DOCUMENTATION AND OPERATIONAL CONSIDERATIONS

Appendix A Regulatory, ethical and study oversight considerations

A-1 Regulatory and ethical considerations

This study will be conducted in accordance with the protocol and with the following:

- Consensus ethical principles derived from international guidelines including the Declaration of Helsinki as amended at 64th WMA General Assembly, Fortaleza, Brazil, October 2013 and Council for International Organizations of Medical Sciences (CIOMS) International Ethical Guidelines
- Applicable ICH Good Clinical Practice (GCP) Guidelines
- Applicable laws and regulations

The protocol, revised protocol, ICF, Investigator's Brochure, and other relevant documents (eg, advertisements) must be submitted to an IRB/IEC by the Investigator and reviewed and approved by the IRB/IEC before the study is initiated.

Any revised protocol will require IRB/IEC and applicable Regulatory Authority approval before implementation of changes made to the study design, except for changes necessary to eliminate an immediate hazard to study patients.

AstraZeneca will be responsible for obtaining the required authorizations to conduct the study from the concerned Regulatory Authority. This responsibility may be delegated to a CRO, but the accountability remains with AstraZeneca.

The Investigator will be responsible for providing oversight of the conduct of the study at the site and adherence to requirements of 21 CFR 312.120, ICH guidelines, the IRB/IEC, European Regulation 536/2014 for clinical studies (if applicable), European Medical Device Regulation 2017/745 for clinical device research (if applicable), and all other applicable local regulations.

The study will be performed in accordance with the AstraZeneca policy on Bioethics and Human Biological Samples.

Regulatory Reporting Requirements for SAEs

- Prompt notification by the investigator to AstraZeneca of an SAE is essential so that legal obligations and ethical responsibilities towards the safety of participants and the safety of a study intervention under clinical investigation are met.
- AstraZeneca has a legal responsibility to notify both the local regulatory authority and other regulatory agencies about the safety of a study intervention under clinical investigation. AstraZeneca will comply with country-specific regulatory requirements relating to safety reporting to the regulatory authority, IRB/IEC, and investigators.

- In the European Union, the Sponsor will comply with safety reporting requirements and procedures as described in the European Clinical Trials Regulation (EU) No 536/2014. All Suspected Unexpected Serious Adverse Reactions (SUSARs) to investigational medicinal product will be reported to the EudraVigilance database within the required regulatory timelines.
- For all studies except those utilising medical devices, investigator safety reports must be prepared for suspected unexpected serious adverse reactions (SUSAR) according to local regulatory requirements and sponsor policy and forwarded to investigators as necessary.
- Adherence to European Medical Device Regulation 2017/745 for clinical device research (if applicable), and all other applicable local regulations.
- An investigator who receives an investigator safety report describing an SAE or other specific safety information (e.g., summary or listing of SAEs) from AstraZeneca will review and then file it along with the IB and will notify the IRB/IEC, if appropriate according to local requirements.

Regulatory Reporting Requirements for Serious Breaches

- Prompt notification by the investigator to AstraZeneca of any (potential) serious breach of the protocol or regulations is essential so that legal and ethical obligations are met.
  - A ‘serious breach’ means a breach likely to affect to a significant degree the safety and rights of a participant or the reliability and robustness of the data generated in the clinical study.
- AstraZeneca will comply with country-specific regulatory requirements relating to serious breach reporting to the regulatory authority, IRB/IEC, and investigators.
  - Where the EU Clinical Trials Regulation 536/2014 applies, AstraZeneca has in place processes to enter details of serious breaches into the European Medicines Agency CTIS. It is important to note that redacted versions of serious breach reports will be available to the public via CTIS.
- If any (potential) serious breach occurs in the course of the study, investigators or other site personnel will inform the appropriate AstraZeneca representatives immediately.
- In certain regions/countries, AstraZeneca has a legal responsibility to notify both the local regulatory authority and other regulatory agencies about such breaches.
- The investigator should have a process in place to ensure that:
  - The site staff or service providers delegated by the investigator/institution are able to identify the occurrence of a (potential) serious breach
  - A (potential) serious breach is promptly reported to AstraZeneca or delegated party, through the contacts (e-mail address or telephone number) provided by AstraZeneca.

A-2 Financial disclosure

Investigators and sub-investigators will provide the Sponsor with sufficient, accurate financial information as requested to allow the Sponsor to submit complete and accurate financial certification or disclosure statements to the appropriate regulatory authorities. Investigators are responsible for providing information on financial interests during the course of the study and for 1 year after completion of the study.

A-3 Informed consent process

A pre-screen ICF is to be signed by patients in Arm 8 who do not have a prior determination of positive PD-L1 status, to permit determination of PD-L1 expression status by local testing prior to the 28-day screening window. When a pre-screen ICF is signed, the main ICF should not be signed until PD-L1 positive expression status has been established.

The Investigator or his/her representative will explain the nature of the study to the patient or his/her legally authorized representative and answer all questions regarding the study.

Patients must be informed that their participation is voluntary, and they are free to refuse to participate and may withdraw their consent at any time and for any reason during the study. Patients or their legally authorized representative will be required to sign a statement of informed consent that meets the requirements of 21 CFR 50, local regulations, ICH guidelines, privacy and data protection requirements, where applicable, and the IRB/IEC or study center.

The medical record must include a statement that written informed consent was obtained before the patient was enrolled in the study and the date the written consent was obtained. The authorized person obtaining the informed consent must also sign the ICF.

Patients must be re-consented to the most current version of the ICF(s) during their participation in the study.

A copy of the ICF(s) must be provided to the patient or the patient's legally authorized representative.

If a patient declines to participate in any voluntary exploratory genetic research component of the study, there will be no penalty or loss of benefit to the patient, and he/she will not be excluded from other aspects of the study.

If a patient's partner becomes pregnant during or within 90 days after the last dose of study treatment, the partner is asked to sign the "Adult Study Informed Consent Form for Pregnant Partners of Study Patients" and provide information about the pregnancy accordingly.

The ICF will contain a separate section that addresses the use of remaining mandatory samples for optional exploratory research. The Investigator or authorized designee will explain to each patient the objectives of the exploratory research. Patients will be told that they are free to refuse to participate and may withdraw their consent at any time and for any reason during the storage period. The patient will give a separate agreement to allow any remaining specimens to be used for exploratory research. Patients who decline to participate in this optional research will indicate this in the ICF. If a patient withdraws consent to the use of donated biological samples, the samples will be disposed of/destroyed, and the action documented. If samples already have been analyzed at the time of the request, AstraZeneca will not be obliged to destroy the results of this research.

A-4 Data protection

The ICF will incorporate wording that complies with relevant data protection and privacy legislation. In some cases, such wording will be in a separate accompanying document. AstraZeneca will not provide individual genotype results to patients, their family members, their general physician, any insurance company, any employer, or any other third party, unless required to do so by law.

Precautions are taken to preserve confidentiality and prevent genetic data from being linked to the identity of the patient. In exceptional circumstances, however, certain individuals might see both the genetic data and the personal identifiers of a patient. For example, in the case of a medical emergency, an AstraZeneca Physician or an Investigator might know a patient's identity and might also have access to his or her genetic data. Also, regulatory authorities may require access to the relevant files. Even so, the patient's medical information and the genetic files would remain physically separate.

Each patient will be assigned a unique identifier by the Sponsor. Any patient records or datasets transferred to the Sponsor will contain only the identifier; patient names or any information which would make the patient identifiable will not be transferred.

The patient must be informed that his/her personal study-related data will be used by the Sponsor in accordance with local data protection law. The level of disclosure must also be explained to the patient.

The patient must be informed that his/her medical records may be examined by Clinical Quality Assurance auditors or other authorized personnel appointed by the Sponsor, by appropriate IRB/IEC members, and by inspectors from regulatory authorities.

A-5 Committees` structure

The safety of all AstraZeneca clinical studies is closely monitored on an on-going basis by AstraZeneca representatives in consultation with Patient Safety. Issues identified will be

addressed; for instance, this could involve amendments to the Clinical Study Protocol and letters to Investigators.

A-6 Dissemination of clinical study data

Any results both technical and lay summaries for this trial, will be submitted to EU CTIS within a year from global End of Trial Date in all participating countries, due to scientific reasons, as otherwise statistical analysis is not relevant.

A description of this clinical study will be available on *http://astrazenecaclinicaltrials.com* and *http://www.clinicaltrials.gov* as will the summary of the *main* study results when they are available. The clinical study and/or summary of *main* study results may also be available on other websites according to the regulations of the countries in which the *main* study is conducted.

A-7 Data quality assurance

All patient data relating to the study will be recorded on printed or electronic CRF unless transmitted to the Sponsor or designee electronically (eg, laboratory data). The Investigator is responsible for verifying that data entries are accurate and correct by physically or electronically signing the eCRF.

The Investigator must maintain accurate documentation (source data) that supports the information entered in the eCRF.

The Investigator must permit study-related monitoring, audits, IRB/IEC review, and regulatory agency inspections and provide direct access to source data documents.

Monitoring details describing strategy, including definition of study-critical data items and processes (eg, risk-based initiatives in operations and quality such as Risk Management and Mitigation Strategies and Analytical Risk-Based Monitoring), methods, responsibilities and requirements, including handling of noncompliance issues and monitoring techniques (central, remote, or on-site monitoring) are included in the Monitoring Plan(s).

AstraZeneca or designee is responsible for medical oversight throughout the conduct of the study which includes clinical reviews of study data in accordance with the currently approved protocol. Monitoring details describing clinical reviews of study data from a medical perspective are included in more detail in the Monitoring Plan(s).

AstraZeneca or designee is responsible for the data management of this study including quality checking of the data.

AstraZeneca assumes accountability for actions delegated to other individuals (eg, Contract Research Organisations).

Study monitors will perform ongoing source data verification as per the Monitoring Plan(s) to confirm that data entered into the eCRF by authorized site personnel are accurate, complete, and verifiable from source documents; that the safety and rights of patients are being protected; and that the study is being conducted in accordance with the currently approved protocol and any other study agreements, ICH GCP, and all applicable regulatory requirements.

Records and documents, including signed ICFs, pertaining to the conduct of this study must be retained by the Investigator for a minimum of 25 years after study archiving or as required by local regulations, according to the AstraZeneca Global retention and Disposal (GRAD) Schedule. No records may be destroyed during the retention period without the written approval of AstraZeneca. No records may be transferred to another location or party without written notification to AstraZeneca.

A-8 Source documents

Source documents provide evidence for the existence of the patient and substantiate the integrity of the data collected. Source documents are filed at the Investigator's site.

Data reported on the CRF that are transcribed from source documents must be consistent with the source documents or the discrepancies must be explained. The Investigator may need to request previous medical records or transfer records, depending on the study. Also, current medical records must be available.

All information in original records and certified copies of original records of clinical findings, observations, or other activities in a clinical study necessary for the reconstruction and evaluation of the study are defined as source documents. Source data are contained in source documents (original records or certified copies).

A-9 Study and Site Closure

The Sponsor designee reserves the right to close the study site or terminate the study at any time for any reason at the sole discretion of the Sponsor. Study sites will be closed upon study completion. A study site is considered closed when all required documents and supplies have been collected and a study-site closure monitoring visit has been performed.

The Investigator may initiate study-site closure at any time, provided there is reasonable cause and sufficient notice is given in advance of the intended termination.

Reasons for the early closure of a study site by the Sponsor or Investigator may include but are not limited to:

- Failure of the Investigator to comply with the protocol, the requirements of the IRB/EC or local health authorities, the Sponsor's procedures, or GCP guidelines
- Inadequate recruitment of participants by the Investigator
- Discontinuation of further study intervention development

If the study is prematurely terminated or suspended, the Sponsor shall promptly inform the Investigators, the IECs/IRBs, the regulatory authorities, and any contract research organization(s) used in the study of the reason for termination or suspension, as specified by the applicable regulatory requirements. The Investigator shall promptly inform the participant and should assure appropriate participant therapy and/or follow-up.

A-10 Publication policy

The results of this study may be published or presented at scientific meetings. If this is foreseen, the Investigator agrees to submit all manuscripts or abstracts to the Sponsor before submission. This allows the Sponsor to protect proprietary information and to provide comments.

The Sponsor will comply with the requirements for publication of study results. In accordance with standard editorial and ethical practice, the Sponsor will generally support publication of multicenter studies only in their entirety and not as individual site data. In this case, a coordinating Investigator will be designated by mutual agreement.

Authorship will be determined by mutual agreement and in line with International Committee of Medical Journal Editors authorship requirements.

Appendix B Adverse event definitions and additional safety information

B-1 Definition of adverse events

An adverse event is the development of any untoward medical occurrence (other than progression of the malignancy under evaluation) in a patient or clinical study patient administered a medicinal product and which does not necessarily have a causal relationship with this treatment. An AE can therefore be any unfavorable and unintended sign (eg, an abnormal laboratory finding), symptom (for example nausea, chest pain), or disease temporally associated with the use of a medicinal product, whether or not considered related to the medicinal product.

The term AE is used to include both serious and non-serious AEs and can include a deterioration of a pre-existing medical occurrence. An AE may occur at any time, including run-in or washout periods, even if no Study treatment has been administered.

B-2 Definitions of serious adverse event

A serious adverse event (SAE) is an AE occurring during any study phase (ie, run-in, treatment, washout, follow-up), that fulfills one or more of the following criteria:

- Results in death
- Is immediately life-threatening
- Requires in-patient hospitalization or prolongation of existing hospitalization
- Results in persistent or significant disability or incapacity
- Is a congenital abnormality or birth defect
- Is an important medical event that may jeopardize the patient or may require medical treatment to prevent one of the outcomes listed above

Regulatory reporting requirements for serious adverse event

- Prompt notification by the Investigator to the Sponsor of an SAE is essential so that legal obligations and ethical responsibilities toward the safety of participants and the safety of a study intervention under clinical investigation are met.
- The Sponsor has a legal responsibility to notify both local authority and other regulatory agencies about the safety of a study intervention under clinical investigation. The Sponsor will comply with country-specific regulatory requirements relating to safety reporting to the regulatory authority, Institutional Review Boards (IRBs)/Independent Ethics Committees (IECs), and Investigators.
- Investigator safety reports must be prepared for suspected unexpected serious adverse reactions (SUSAR) according to local regulatory requirements and Sponsor policy and forwarded to Investigators as necessary.

- An Investigator who receives safety report describing an SAE or other safety information (eg, summary of listing of SAEs) from the Sponsor will review and then file it along with the Investigator's Brochure and will notify the IRB/IEC if appropriate according to local requirements.

B-3 Life threatening

'Life-threatening' means that the patient was at immediate risk of death from the AE as it occurred, or it is suspected that use or continued use of the product would result in the patient's death. 'Life-threatening' does not mean that had an AE occurred in a more severe form it might have caused death (eg, hepatitis that resolved without hepatic failure).

B-4 Hospitalization

Outpatient treatment in an emergency room is not in itself a serious AE, although the reasons for it may be (eg, bronchospasm, laryngeal edema). Hospital admissions and/or surgical operations planned before or during a study are not considered AEs if the illness or disease existed before the patient was enrolled in the study, provided that it did not deteriorate in an unexpected way during the study.

B-5 Important medical event or medical treatment

Medical and scientific judgement should be exercised in deciding whether a case is serious in situations where important medical events may not be immediately life threatening or result in death, hospitalization, disability or incapacity but may jeopardize the patient or may require medical treatment to prevent one or more outcomes listed in the definition of serious. These should usually be considered as serious.

Simply stopping the suspect drug does not mean that it is an important medical event; medical judgement must be used.

- Angioedema not severe enough to require intubation but requiring iv hydrocortisone treatment
- Hepatotoxicity caused by paracetamol (acetaminophen) overdose requiring treatment with N-acetylcysteine
- Intensive treatment in an emergency room or at home for allergic bronchospasm
- Blood dyscrasias (eg, neutropenia or anemia requiring blood transfusion, etc.) or convulsions that do not result in hospitalization
- Development of drug dependency or drug abuse

B-6 CTCAE grade

The grading scales found in the revised NCI CTCAE latest version will be utilized for all events with an assigned CTCAE grading. For those events without assigned CTCAE grades, the criteria recommended in the CTCAE manual that converts severity levels into CTCAE grades should be used. A copy of the CTCAE can be downloaded from the Cancer Therapy Evaluation Program website (http://ctep.cancer.gov). The applicable version of CTCAE should be described clearly.

It is important to distinguish between serious and severe AEs. Severity is a measure of intensity whereas seriousness is defined by the criteria in Appendix B-2. An AE of severe intensity need not necessarily be considered serious. For example, nausea that persists for several hours may be considered severe nausea, but not a SAE unless it meets the criteria shown in Appendix B-2. On the other hand, a stroke that results in only a limited degree of disability may be considered a mild stroke but would be a SAE when it satisfies the criteria shown in Appendix B-2.

B-7 A guide to interpreting the causality question

When making an assessment of causality consider the following factors when deciding if there is a 'reasonable possibility' that an AE may have been caused by the drug.

- Time Course. Exposure to suspect drug. Has the patient actually received the suspect drug? Did the AE occur in a reasonable temporal relationship to the administration of the suspect drug?
- Consistency with known drug profile. Was the AE consistent with the previous knowledge of the suspect drug (pharmacology and toxicology) or drugs of the same pharmacological class? Or could the AE be anticipated from its pharmacological properties?
- De-challenge experience. Did the AE resolve or improve on stopping or reducing the dose of the suspect drug?
- No alternative cause. The AE cannot be reasonably explained by another etiology such as the underlying disease, other drugs, other host or environmental factors.
- Re-challenge experience. Did the AE reoccur if the suspected drug was reintroduced after having been stopped? AstraZeneca would not normally recommend or support a re-challenge.
- Laboratory tests. A specific laboratory investigation (if performed) has confirmed the relationship.

In difficult cases, other factors could be considered such as:

- Is this a recognized feature of overdose of the drug?
- Is there a known mechanism?

Causality of 'related' is made if following a review of the relevant data, there is evidence for a 'reasonable possibility' of a causal relationship for the individual case. The expression 'reasonable possibility' of a causal relationship is meant to convey, in general, that there are facts (evidence) or arguments to suggest a causal relationship.

The causality assessment is performed based on the available data including enough information to make an informed judgment. With limited or insufficient information in the case, it is likely that the event(s) will be assessed as 'not related'.

Causal relationship in cases where the disease under study has deteriorated due to lack of effect should be classified as no reasonable possibility.

B-8 Medication error, drug abuse, and drug misuse

Medication error

For the purposes of this clinical study a medication error is an **unintended** failure or mistake in the treatment process for an IMP or AstraZeneca NIMP that either causes harm to the participant or has the potential to cause harm to the participant.

A medication error is not lack of efficacy of the drug, but rather a human or process related failure while the drug is in control of the study site staff or participant.

Medication error includes situations where an error:

- occurred
- **was identified and** intercepted before the participant received the drug
- did not occur, but circumstances were recognized that could have led to an error

Examples of events to be reported in clinical studies as medication errors:

- Drug name confusion
- Dispensing error (eg, medication prepared incorrectly, even if it was not actually given to the participant)
- Drug not administered as indicated, eg, wrong route, dose (error greater than ± 10%), or wrong site of administration
- Drug not taken as indicated (eg, tablet dissolved in water when it should be taken as a solid tablet)
- Drug not stored as instructed (eg, kept in the fridge when it should be at room temperature)
- Wrong participant received the medication (excluding RTSM (IRT) errors)
- Wrong drug administered to participant (excluding RTSM (IRT) errors)

Examples of events that **do not** require reporting as medication errors in clinical studies:

- Errors related to or resulting from RTSM (IRT), including those which lead to one of the above listed events that would otherwise have been a medication error
- Participant accidentally missed drug dose(s) (eg, forgot to take medication)
- Accidental overdose (will be captured as an overdose)
- Participant failed to return unused medication or empty packaging

Medication errors are not regarded as AEs but AEs may occur as a consequence of the medication error.

Drug abuse

For the purpose of this study, drug abuse is defined as the persistent or sporadic intentional, non-therapeutic excessive use of IMP/study intervention or AstraZeneca NIMP for a perceived reward or desired non-therapeutic effect.

Any events of drug abuse, with or without associated AEs, are to be captured and forwarded to the DES using the Drug Abuse Report Form. This form should be used both if the drug abuse happened in a study participant or if the drug abuse involves a person not enrolled in the study (such as a relative of the study participant).

Examples of drug abuse include but are not limited to:

- The drug is used with the intent of getting a perceived reward (by the study participant or a person not enrolled in the study)
- The drug in the form of a tablet is crushed and injected or snorted with the intent of getting high.

Drug misuse

Drug misuse is the intentional and inappropriate use (by a study participant) of IMP/study intervention or AstraZeneca NIMP for medicinal purposes outside of the authorized product information, or for unauthorized IMPs/study interventions or AstraZeneca NIMPs, outside the intended use as specified in the protocol, and includes deliberate administration of the product by the wrong route.

Events of drug misuse, with or without associated AEs, are to be captured and forwarded to the data entry site (DES) using the Drug Misuse Report Form. This form should be used both if the drug misuse happened in a study participant or if the drug misuse regards a person not enrolled in the study (such as a relative of the study participant).

Examples of drug misuse include but are not limited to:

- The drug is used with the intention to cause an effect in another person
- The drug is sold to other people for recreational purposes
- The drug is used to facilitate assault in another person
- The drug is deliberately administered by the wrong route
- The drug is split in half because it is easier to swallow, when it is stated in the protocol that it must be swallowed whole
- Only half the dose is taken because the study participant feels that they were feeling better when not taking the whole dose
- Someone who is not enrolled in the study intentionally takes the drug.

Appendix C Handling of human biological samples

C-1 Chain of custody of biological samples

A full chain of custody is maintained for all samples throughout their lifecycle.

The Investigator at each center keeps full traceability of collected biological samples from the patients while in storage at the center until shipment or disposal (where appropriate).

The sample receiver keeps full traceability of the samples while in storage and during use until used or disposed of or until further shipment and keeps documentation of receipt of arrival.

AstraZeneca will keep oversight of the entire life cycle through internal procedures, monitoring of study sites, auditing or process checks, and contractual requirements of external laboratory providers

Samples retained for further use will be stored in the AZ-assigned Biobanks and will be registered by the AstraZeneca Biobank Team during the entire life cycle.

If required, AstraZeneca will ensure that remaining biological samples are returned to the site according to local regulations or at the end of the retention period, whichever is the sooner.

C-2 Withdrawal of Informed Consent for donated biological samples

AstraZeneca ensures that biological samples are returned to the source or destroyed at the end of a specified period as described in the informed consent.

If a patient withdraws consent to the use of donated biological samples, the samples will be disposed of/destroyed, and the action documented. If samples are already analyzed, AstraZeneca is not obliged to destroy the results of this research.

As collection of the biological sample(s) is an optional part of the study, then the patient may continue in the study.

The Investigator:

- Ensures patients' withdrawal of informed consent to the use of donated samples is notified immediately to AstraZeneca
- Ensures that biological samples from that patient, if stored at the study site, are immediately identified, disposed of /destroyed, and the action documented
- Ensures the organization(s) holding the samples is/are informed about the withdrawn consent immediately and that samples are disposed of/destroyed, the action documented, and the signed document returned to the study site

- Ensures that the patient and AstraZeneca are informed about the sample disposal.

AstraZeneca ensures the organizations holding the samples is/are informed about the withdrawn consent immediately and that samples are disposed of/destroyed and the action documented and returned to the study site.

C-3 International Airline Transportation Association (IATA) 6.2 guidance document

LABELLING AND SHIPMENT OF BIOHAZARD SAMPLES

International Airline Transportation Association (IATA) classifies biohazardous agents into 3 categories (http://www.iata.org/whatwedo/cargo/dangerous_goods/infectious_substances.htm). For transport purposes the classification of infectious substances according to risk groups was removed from the Dangerous Goods Regulations in the 46th edition (2005). Infectious substances are now classified either as Category A, Category B or Exempt. There is no direct relationship between Risk Groups and Categories A and B.

Category A Infectious Substances are infectious substances in a form that, when exposure to it occurs, is capable of causing permanent disability, life-threatening or fatal disease in otherwise healthy humans or animals. Category A pathogens are eg, Ebola, Lassa fever virus:

- are to be packed and shipped in accordance with IATA Instruction 602.

Category B Infectious Substances are infectious Substances that do not meet the criteria for inclusion in Category A. Category B pathogens are eg, Hepatitis A, B, C, D, and E viruses, Human immunodeficiency virus types 1 and 2. They are assigned the following UN number and proper shipping name:

- UN 3373 – Biological Substance, Category B
- are to be packed in accordance with UN3373 and IATA 650

Exempt - all other materials with minimal risk of containing pathogens

- Clinical study samples will fall into Category B or exempt under IATA regulations
- Clinical study samples will routinely be packed and transported at ambient
- temperature in IATA 650 compliant packaging (http://www.iata.org/whatwedo/cargo/dangerous_goods/infectious_substances.htm)
- **Biological samples transported in dry ice require additional dangerous goods specification for the dry-ice content**

- IATA compliant courier and packaging materials should be used for packing and transportation and packing should be done by an IATA certified person, as applicable
- Samples routinely transported by road or rail are subject to local regulations which require that they are also packed and transported in a safe and appropriate way to contain any risk of infection or contamination by using approved couriers and packaging/containment materials at all times. The IATA 650 biological sample containment standards are encouraged wherever possible when road or rail transport is used.

Appendix D [redacted]

D-1 [redacted]

[redacted] may impact a patient's [redacted], [redacted] may be due to [redacted] that impact [redacted] herefore, where local regulations and IRB/IEC allow, [redacted] from consenting patients.

AstraZeneca intends to collect and store [redacted] for [redacted] to explore how [redacted]

In addition, collection of [redacted] from populations with well described [redacted] may lead to [redacted] and, possibly, to [redacted].

[redacted] may consist of the [redacted] of the patient´s [redacted]

The results of [redacted] may be reported in the clinical study report (CSR) or in a separate study summary.

The Sponsor will store [redacted] in a secure storage space with adequate measures to protect confidentiality.

The samples will be retained while research on durvalumab and other study treatments continues but no longer than [redacted] or other period per local requirements.

D-2 [redacted] plan and procedures

Selection of [redacted] population

Study selection record

All patients will be asked to participate in this [redacted]. Participation is voluntary and if a patient declines to participate there will be no penalty or loss of benefit. The patient will not be excluded from any aspect of the main study.

Inclusion criteria

- For inclusion in this [redacted], patients must fulfill all of the inclusion criteria described in the main body of the Clinical Study Protocol **and**: Provide informed consent for the [redacted] sampling and analyses.

Exclusion criteria

Exclusion from this [redacted] may be for any of the exclusion criteria specified in the main study or any of the following:

[redacted]

Withdrawal of consent for [redacted]:

Patients may withdraw from this [redacted] at any time, independent of any decision concerning participation in other aspects of the main study. Voluntary withdrawal will not prejudice further treatment. Procedures for withdrawal are outlined Appendix C-2.

Collection of samples for [redacted]

The blood sample for [redacted] will be obtained from the patients [redacted]. Although [redacted] such patients would be important to include in any [redacted] analysis. If for any reason the sample is not drawn at the first dosing visit, it may be taken at any visit until the last study visit. Only one sample should be collected per patient for [redacted] during the study. Samples will be collected, labeled, stored, and shipped as detailed in the Laboratory Manual.

[redacted] samples

The processes adopted for the [redacted] of samples for [redacted] are important to maintain patient confidentiality. Samples will be stored for a maximum of [redacted] from the date of last patient last visit, after which they will be destroyed. [redacted] that is used up during analyses. Samples will be stored and used until no further analyses are possible or the maximum storage time has been reached.

[redacted] No personal details identifying the individual will be available to any person (AstraZeneca employee or designated organizations working with [redacted]).

The link between the patient enrollment/randomization code and the second number will be maintained and stored in a secure environment, with restricted access at AstraZeneca or designated organizations. The link will be used to identify the relevant [redacted] for analysis, facilitate correlation [redacted] results with clinical data, allow regulatory audit, and permit tracing of samples for destruction in the case of withdrawal of consent.

Ethical and regulatory requirements

The principles for ethical and regulatory requirements for the study, including this [redacted], are outlined in Appendix A.

Informed consent

[redacted] of this study is optional and the patient may participate in other components of the main study without participating in the [redacted]. To participate in the [redacted] of the study the patient must sign and date the appropriate section(s) of the consent form. A copy of the signed and dated consent form must be given to the patient and the original filed at the study center. The Principal Investigator(s) is responsible for ensuring that consent is given freely and that the patient understands that they may freely withdrawal from the [redacted] of the study at any time.

Patient data protection

AstraZeneca will not provide individual [redacted], [redacted], [redacted] unless required to do so by law.

Extra precautions are taken to preserve confidentiality and prevent [redacted] In exceptional circumstances, however, certain individuals might see both [redacted]. For example, in the case of a medical emergency, [redacted] In addition, Regulatory authorities may require access to the relevant files, though the patient's medical information and the [redacted] would remain physically separate.

Data management

Any [redacted] generated in this study will be stored at a secure system at AstraZeneca and/or designated organizations to analyses the samples.

AstraZeneca and its designated organizations may share summary results (such as [redacted] from this [redacted] with other researchers, such as hospitals, academic organizations or health insurance companies. This can be done by placing the results in scientific databases, where they can be combined with the results of similar studies to learn even more about health and disease. The researchers can

only use this information for health related research purposes. Researchers may see summary results but they will not be able to see individual patient data or any personal identifiers.

Some or all of the clinical datasets from the main study may be merged with the [redacted] in a suitable secure environment separate from the clinical database.

Statistical methods and determination of sample size

The number of patients that will agree to participate in the [redacted] is unknown. It is therefore not possible to establish whether sufficient data will be collected to allow a formal statistical evaluation or whether only descriptive statistics will be generated. A Statistical Analysis Plan may be prepared where appropriate.

Appendix E Actions required in cases of increases in liver biochemistry and evaluation of Hy's law

E-1 Follow-up

E-2 Introduction

This Appendix describes the process to be followed in order to identify and appropriately report Potential Hy's Law (PHL) cases and Hy's Law (HL) cases. It is not intended to be a comprehensive guide to the management of elevated liver biochemistries. Specific guidance on managing liver abnormalities can be found in the Dosing Modification and Toxicity Management Guidelines (see Section 8.4.6).

During the course of the study, the Investigator will remain vigilant for increases in liver biochemistry. The Investigator is responsible for determining whether a subject meets potential PHL criteria at any point during the study.

All sources of laboratory data are appropriate for the determination of PHL and HL events; this includes samples taken at scheduled study visits and other visits including central and all local laboratory evaluations even if collected outside of the study visits; for example, PHL criteria could be met by an elevated ALT from a central laboratory and/or elevated TBL) from a local laboratory.

The Investigator will also review adverse event (AE) data (for example, for AEs that may indicate elevations in liver biochemistry) for possible PHL events.

The Investigator participates, together with AstraZeneca clinical project representatives, in the review and assessment of cases meeting PHL criteria to agree whether HL criteria are met. HL criteria are met if there is no alternative explanation for the elevations in liver biochemistry other than drug-induced liver injury (DILI) caused by the investigational medicinal product (IMP).

The Investigator is responsible for recording data pertaining to PHL/HL cases and for reporting serious adverse events (SAEs) and AEs according to the outcome of the review and assessment in line with standard safety reporting processes.

E-3 Definitions

Potential Hy's law

Aspartate aminotransferase (AST) or alanine aminotransferase (ALT) ≥3 × upper limit of normal (ULN) **together with** TBL ≥2 × ULN at any point during the study following the start of study medication irrespective of an increase in alkaline phosphatase (ALP).

Hy's law

AST or ALT ≥3 × ULN **together with** TBL ≥2 × ULN, where no other reason, other than the IMP, can be found to explain the combination of increases, eg, elevated ALP indicating cholestasis, viral hepatitis, another drug.

For PHL and HL, the elevation in transaminases must precede or be coincident with (ie, on the same day) the elevation in TBL, but there is no specified timeframe within which the elevations in transaminases and TBL must occur.

E-4 Identification of potential Hy's law cases

In order to identify cases of PHL, it is important to perform a comprehensive review of laboratory data for any patient who meets any of the following identification criteria in isolation or in combination:

- ALT ≥3 × ULN
- AST ≥3 × ULN
- TBL ≥2 × ULN

The Investigator will, without delay, review each new laboratory report and, if the identification criteria are met, will:

- Determine whether the patient meets PHL criteria (see Appendix E-3 for definition) by reviewing laboratory reports from all previous visits
- Promptly enter the laboratory data into the laboratory electronic case report form (eCRF)

E-4.1 Potential Hy's law criteria not met

If the patient does not meet PHL criteria, the Investigator will:

- Perform follow-up on subsequent laboratory results according to the guidance provided in the clinical study protocol.

E-4.2 Potential Hy's law criteria met

If the patient does meet PHL criteria, the Investigator will:

- Determine whether PHL criteria were met at any study visit prior to starting study treatment (see Section 8.4 Safety Reporting)
- Notify the AstraZeneca representative, who will then inform the central study team

- Within 1 day of PHL criteria being met, the Investigator will report the case as an SAE of potential Hy's law; serious criteria "important medical event" and causality assessment "yes/related" according to CSP process for SAE reporting.
- For patients who met PHL criteria prior to starting IMP, the Investigator is not required to submit a PHL SAE unless there is a significant change* in the patients' condition
- The Study Clinical Lead contacts the Investigator, to provide guidance, to discuss, and to agree to an approach for the study patients' follow-up and the continuous review of data. Subsequent to this contact, the Investigator will:
  – Monitor the patient until liver biochemistry parameters and appropriate clinical symptoms and signs return to normal or baseline levels or as long as medically indicated. Complete follow-up SAE Form as required.
  – Investigate the etiology of the event and perform diagnostic investigations as discussed with the Study Clinical Lead.
  – Complete the 3 Liver eCRF Modules as information becomes available
- Unless an alternate etiology for the elevated liver enzymes is known, report as an SAE using standard reporting procedures

*A "significant" change in the patient's condition refers to a clinically relevant change in any of the individual liver biochemistry parameters (ALT, AST, or TBL) in isolation or in combination or a clinically relevant change in associated symptoms. The determination of whether there has been a significant change will be at the discretion of the Investigator; this may be in consultation with the Study Clinical Lead if there is any uncertainty.

E-5 Review and assessment of potential Hy's law cases

The instructions in this section should be followed for all cases where PHL criteria are met.

As soon as possible after the biochemistry abnormality was initially detected, the Study Clinical Lead contacts the Investigator in order to review available data and agree on whether there is an alternative explanation for meeting PHL criteria other than DILI caused by the IMP, to ensure timely analysis and reporting to health authorities within 15 calendar days from the date PHL criteria were met. The AstraZeneca Global Clinical Lead or equivalent and Global Safety Physician will also be involved in this review together with other subject matter experts as appropriate.

According to the outcome of the review and assessment, the Investigator will follow the instructions below.

Where there is an agreed alternative explanation for the ALT or AST and TBL elevations, a determination of whether the alternative explanation is an AE will be made and subsequently whether the AE meets the criteria for an SAE:

- If the alternative explanation is **not** an AE, record the alternative explanation on the appropriate CRF
- If the alternative explanation is an AE/SAE, update the previously submitted Potential Hy's Law SAE and AE CRFs accordingly with the new information (reassessing event term; causality and seriousness criteria) following the AZ standard processes.

If it is agreed that there is **no** explanation that would explain the ALT or AST and TBL elevations other than the IMP:

- Send updated SAE (report term "Hy's Law") according to AstraZeneca standard processes.
- The "Medically Important" serious criterion should be used if no other serious criteria apply.
- As there is no alternative explanation for the HL case, a causality assessment of "related" should be assigned.

If, there is an unavoidable delay, of over 15 calendar days in obtaining the information necessary to assess whether or not the case meets the criteria for HL, then it is assumed that there is no alternative explanation until such time as an informed decision can be made:

- Provides any further update to the previously submitted SAE of Potential Hy's Law, (report term now "Hy's Law case") ensuring causality assessment is related to IMP and seriousness criteria is medically important, according to CSP process for SAE reporting.
- Continue follow-up and review according to agreed plan. Once the necessary supplementary information is obtained, repeat the review and assessment to determine whether HL criteria are still met. Update the previously submitted PHL SAE report following CSP process for SAE reporting, according to the outcome of the review and amending the reported term if an alternative explanation for the liver biochemistry elevations is determined.

E-6 Actions required when potential Hy's law criteria are met before and after starting study treatment

This section is applicable to patients with liver metastases who meet PHL criteria on study treatment, having previously met PHL criteria at a study visit prior to starting study treatment.

At the first on-study treatment occurrence of PHL criteria being met, the Investigator will determine if there has been a significant change* in the patient's condition compared with the last visit where PHL criteria were met.

- If there is no significant change, no action is required

- If there is a significant change, notify the AstraZeneca representative, who will inform the central Study Team, then follow the subsequent process described in Appendix E-4.2.

*A “significant” change in the patient’s condition refers to a clinically relevant change in any of the individual liver biochemistry parameters (ALT, AST, or TBL) in isolation or in combination or a clinically relevant change in associated symptoms. The determination of whether there has been a significant change will be at the discretion of the Investigator; this may be in consultation with the Study Clinical Lead if there is any uncertainty.

E-7 Actions required for repeat episodes of potential Hy’s law

This section is applicable when a patient meets PHL criteria on study treatment and has already met PHL criteria at a previous on-study treatment visit.

The requirement to conduct follow-up, review, and assessment of a repeat occurrence(s) of PHL is based on the nature of the alternative cause identified for the previous occurrence.

The Investigator should determine the cause for the previous occurrence of PHL criteria being met and answer the following question:

> Was the alternative cause for the previous occurrence of PHL criteria being met found to be the disease under study (eg, chronic or progressing malignant disease, severe infection or liver disease) or did the patient meet PHL criteria prior to starting study treatment and at first on-study treatment visit, as described in Appendix E-6?

If **No**: Follow the process described in Appendix E-4.2.

If **Yes**: Determine if there has been a significant* change in the patient’s condition compared with when PHL criteria were previously met.

> If there is no significant change, no action is required.
>
> If there is a significant change, follow the process described in Appendix E-4.2.

*A “significant” change in the patient’s condition refers to a clinically relevant change in any of the individual liver biochemistry parameters (ALT, AST, or TBL) in isolation or in combination or a clinically relevant change in associated symptoms. The determination of whether there has been a significant change will be at the discretion of the Investigator; this may be in consultation with the Study Clinical Lead if there is any uncertainty.

Appendix F Guidelines for evaluation of objective tumor response using RECIST 1.1 Criteria (Response Evaluation Criteria in Solid Tumors)

Introduction

This appendix details the implementation of Response Evaluation Criteria in Solid Tumors v1.1 (RECIST 1.1) guidelines (Eisenhauer et al 2009). Investigator assessments will use the RECIST 1.1 guidelines described in this appendix. Additional special guidance is provided for evaluation of scans collected after a RECIST 1.1-defined radiological progression.

Imaging modalities and acquisition specifications for RECIST 1.1

A summary of the imaging modalities that can be used for tumor assessment of target lesions (TLs), non-target lesions (NTLs), and new lesions (NLs) is provided in Table 29.

Table 29 Summary of imaging modalities for tumor assessment

Target lesions	Non-target lesions	New lesions
CT	CT	CT
MRI	MRI	MRI
	Plain X-ray	Plain X-ray
	Chest X-ray	Chest X-ray
		Bone scan (Scintigraphy)
		FDG-PET/CT

CT Computed tomography; MRI Magnetic resonance imaging; FDG-PET ^{18}F-Fluoro-deoxyglucose positron emission tomography

CT and MRI

Computed tomography (CT) with intravenous (IV) contrast, is the preferred imaging modality, and magnetic resonance imaging (MRI) with IV contrast being acceptable should CT be contraindicated, to generate reproducible anatomical images for tumor assessments, ie, for measurement of TLs, assessment of NTLs, and identification of NLs. It is essential that the same correct imaging modality, image acquisition parameters (eg, anatomic coverage, imaging sequences, etc), imaging facility, tumor assessor (eg, radiologist), and tumor assessment method (eg, RECIST 1.1) are used consistently for each patient throughout the study. The use of the same scanner for serial scans is recommended, if possible. It is important to follow the image collection/tumor assessment schedule as closely as possible (refer to the Clinical Study Protocol [CSP] Schedule of Assessments), and this on-study imaging schedule MUST be followed regardless of any delays in dosing or missed imaging visits. If an unscheduled assessment is performed (eg, to investigate clinical signs or symptoms of

progression), and the patient has not progressed, every attempt should be made to perform the subsequent scan acquisitions at the next scheduled imaging visit.

Due to its inherent rapid acquisition (seconds), CT is the imaging modality of choice. Body scans should be performed with breath-hold scanning techniques, if possible. Therefore, CT of the chest is recommended over MRI due to significant motion artifacts (heart, major blood vessels, and breathing) associated with MRI. MRI has excellent contrast and spatial and temporal resolutions; however, there are many image acquisition variables involved in MRI, which greatly impact image quality, lesion conspicuity, and measurement. Furthermore, the availability of MRI is variable globally. The modality used at follow-up should be the same as was used at baseline, and the lesions should be measured or assessed on the same pulse sequence. In general, local oncology diagnostic imaging parameters are applied for scan acquisition. It is beyond the scope of this appendix to prescribe specific MRI pulse sequence parameters for all scanners, body parts, and diseases.

The most critical CT and MRI image acquisition parameters for optimal tumor evaluation are anatomic coverage, contrast administration, slice thickness, and reconstruction interval.

a. **Anatomic coverage:** Optimal anatomic coverage for most solid tumors is the [redacted]. Coverage should encompass all areas of known predilection for metastases in the disease under evaluation and should additionally investigate areas that may be involved based on signs and symptoms of individual patients. Because a lesion later identified in a body part not scanned at baseline would be considered as a new lesion representing PD, careful consideration should be given to the extent of imaging coverage at baseline and at subsequent follow-up timepoints. This will enable better consistency not only of tumor measurements but also identification of a new disease.

   Required anatomical regions to be imaged for assessment of tumor burden (TLs and/or NTLs) at baseline and follow-up visits vary according to the study, and these are specified in the main CSP (eg, Schedule of Assessments). Examples include the following:

   - IV contrast-enhanced CT of [redacted] (including the [redacted]
   - Non-contrast CT of [redacted] and IV contrast enhanced [redacted] (including the [redacted]
   - IV contrast-enhanced CT or MRI of [redacted]
   - IV contrast-enhanced MRI (preferred) or CT of [redacted]

   For [redacted] imaging, the following are scanning options in decreasing order of preference, with additional options (2 to 4) when patients have sensitivity to IV contrast or have compromised renal function:

- [redacted] CT with IV CT contrast (most preferred)
- [redacted] CT without IV-contrast + [redacted] MRI with IV MRI contrast, if CT IV contrast (iodine-based) is medically contraindicated at any time during the study
- [redacted] CT without IV contrast, if both IV CT and MRI contrast are medically contraindicated, or the patient has compromised renal function
- [redacted] MRI with IV MRI contrast, if CT cannot be performed at any time during the study

b. **IV contrast administration**: Optimal visualization and measurement of metastases in solid tumors require consistent administration (dose and rate) of IV contrast as well as timing of scanning. An adequate volume of a suitable contrast agent should be given so that the tumor lesions are demonstrated to best effect, and a consistent method is used on subsequent examinations for any given patient. Oral contrast is recommended to help visualize and differentiate structures in [redacted]

c. **Slice thickness and reconstruction interval:** It is recommended that CT or MRI scans be acquired or reconstructed as contiguous (no gap) with [redacted] mm slice thickness throughout the entire anatomic region of interest for optimal lesion measurements. However, a slice thickness of [redacted] mm is preferred. Exceptionally, particular institutions may perform medically acceptable scans at slice [redacted] mm. If this occurs, the minimum size of measurable lesions at baseline should be twice the slice thickness of the baseline scans.

For CT scans, all window settings should be included in the assessment, particularly in the thorax where lung and soft tissue windows should be considered. When measuring lesions, the TL should be measured on the same window setting for repeated examinations throughout the study.

Chest X-ray

Chest X-ray assessment will not be used for assessment of TL. Chest X-ray can, however, be used to assess NTL and to identify the presence of NLs. However, there is preference that a higher resolution modality such as CT be used to confirm the presence of NLs.

Plain X-ray

Plain X-ray may be used as a method of assessment for bone NTL and to identify the presence of new bone lesions.

Isotopic bone scan

Bone lesions identified on an isotopic bone scan at baseline and confirmed by CT, MRI, or X-ray at baseline should be recorded as NTL and followed by the same method per baseline assessment (CT, MRI, or X-ray).

Isotopic bone scans may be used as a method of assessment to identify the presence of new bone lesions at follow-up visits. NLs may be recorded in case positive hot spots appear on a bone scan that were not present on a previous bone scan; however, a newly observed equivocal hot spot on a bone scan that cannot be verified with correlative imaging (CT, MRI, X-ray) of the same anatomical region shall not be the only trigger for PD assessment at that.

FDG-PET/CT

^{18}F-Fluoro-deoxyglucose positron emission tomography/CT (FDG-PET/CT) scans may be used as a method for identifying new extrahepatic lesions (but not intrahepatic lesions) for RECIST 1.1 assessments, according to the following algorithm: NLs will be recorded where there is positive ^{18}F-Fluoro-deoxyglucose uptake[1] not present in the baseline or a prior FDG-PET scan or in a location corresponding to an NL on a companion CT/MRI collected close in time to the FDG-PET scan. The positron emission tomography (PET) portion of the PET/CT introduces additional data that may bias an Investigator, if it is not routinely or serially performed. Therefore, if there is no baseline or prior FDG-PET scan available for comparison, and no evidence of NLs on companion CT/MRI scans, then follow-up CT/MRI assessments should continue per the regular imaging schedule in order to verify the unequivocal presence of NLs.

At present, low dose or attenuation correction CT portions of a combined FDG-PET/CT scan are of limited use in anatomically based efficacy assessments, and it is therefore suggested that they should not substitute for dedicated diagnostic contrast-enhanced CT scans for tumor measurements by RECIST 1.1. In exceptional situations, if a site can document that the CT performed, as part of a PET/CT examination, is of identical diagnostic quality (with IV contrast) to a dedicated diagnostic CT scan, and then the CT portion of the PET/CT can be used for RECIST 1.1 tumor assessments. Caution that this is not recommended because the PET portion of the CT introduces additional (PET) data that may bias an Investigator if it is not routinely or serially performed.

---

[1] A positive FDG-PET scan lesion should be reported only when an uptake (eg, standardized uptake value) greater than twice that of the surrounding tissue or liver is observed.

Ultrasound

Ultrasound examination will not be used for RECIST 1.1 assessment of tumors as it is not a reproducible acquisition method (operator-dependent), is subjective in interpretation, and may not provide an accurate assessment of true tumor size. Tumors identified by ultrasound will need to be assessed by correlative CT or MRI anatomical scan.

Other Tumor Assessments

Clinical examination

Clinical examination of skin/surface lesions (by visual inspection or manual palpation) will not be used for RECIST 1.1 assessments. Tumors identified by clinical examination will need to be assessed by correlative CT or MRI anatomical scans.

Endoscopy and laparoscopy

Endoscopy and laparoscopy will not be used for tumor assessments, as they are not validated in the context of tumor assessment.

Histology and cytology

Histology or tumor markers on tumor biopsy samples will not be used as part of the tumor response assessment per RECIST 1.1.

Results of cytological examination for the neoplastic origin of any effusion (eg, ascites, pericardial effusion, or pleural effusion) that appears or worsens during the study will not be used as part of the tumor response assessment per RECIST 1.1.

Furthermore, an overall assessment of complete response (CR; all other disease disappears or reverts to normal) would be changed to PR if an effusion remains present radiologically.

Measurability of Tumor Lesions at Baseline

RECIST 1.1-measurable lesions at baseline:

It is a tumor lesion that can be accurately measured at baseline as ≥10 mm in the longest diameter for non-nodal lesions or ≥15 mm in short axis[2] diameter for lymph node lesions with IV contrast-enhanced CT or MRI and that is suitable for accurate repeated measurements. Please see additional RECIST 1.1 guidance below on measurability of intrahepatic hepatocellular carcinoma lesions and porta hepatis lymph nodes.

[2] The short axis is defined as the longest in-plane axis perpendicular to long axis.

Non-measurable lesions at baseline:

- Truly non-measurable lesions include the following:
  - Bone lesions (see exception below for soft tissue component)
  - Leptomeningeal disease
  - Ascites, pleural, or pericardial effusion
  - Inflammatory breast disease
  - Lymphangitic involvement of the skin or lung
- All other lesions, including small lesions (longest diameter <10 mm or pathological lymph nodes with ≥10 mm to <15 mm short axis diameter at baseline[3]).
- Previously irradiated lesions[4]
- Brain metastasis

Special considerations regarding lesion measurability at baseline:

- Bone lesions
  - Bone scan, PET scan or plain X-ray alone are not considered adequate imaging techniques to measure bone lesions; however, these techniques can be used to confirm the presence or disappearance of bone lesions.
  - Lytic bone lesions or mixed lytic-blastic lesions, with identifiable soft tissue components, can be considered measurable if the soft tissue component meets the definition of measurability.
  - Blastic bone lesions are considered non-measurable.
- Cystic lesions thought to represent cystic metastases can be considered measurable lesions if they meet the criteria for measurability from a radiological point of view, but if non-cystic lesions are present in the same patient, these should be selected over cystic lesions as TLs.

RECIST 1.1 Target Lesion Selection at Baseline:

A maximum of 5 measurable lesions, with a maximum of 2 lesions per organ (including lymph nodes collectively considered as a single organ), representative of all lesions involved

---

[3] Lymph nodes with <10 mm short axis diameter are considered non-pathological and should not be recorded or followed as NTLs.

[4] Localized post-radiation changes which affect lesion sizes may occur. Therefore, lesions that have been previously irradiated are typically considered non-measurable and as NTL at baseline and followed up as part of the NTL assessment.

should be identified as TLs at baseline. TLs should be selected on the basis of their size (longest diameter for non-nodal lesions or short axis diameter for nodal lesions), but in addition they should be those that lend themselves to reproducible repeated measurements. It may be the case that, on occasion, the largest lesion does not lend itself to reproducible measurement, in which circumstance the next largest lesion, which can be measured reproducibly, should be selected.

Lymph nodes, in any location (local or regional and distant), are collectively considered as a single organ, with a maximum of 2 lymph node locations as TLs. A bilateral organ (eg, adrenal glands), a segmented organ (eg, liver), or a multi-lobed organ (eg, lung) is each considered as a single organ.

The site and location of each TL should be documented as well as the longest axis diameter for non-nodal lesions (or short axis diameter for lymph nodes). All measurements should be recorded in millimeters. At baseline the sum of the diameters for all TL will be calculated and reported as the baseline sum of diameters. At follow-up visits, the sum of diameters for all TL will be calculated and reported as the follow-up sum of diameters.

Special cases for target lesion assessment at baseline:

- For TL measurable in 2 or 3 dimensions, always report the longest diameter. For pathological lymph nodes measurable in 2 or 3 dimensions, always report the short axis diameter.
- When lymph nodes are coalesced and no longer separable in a conglomerate mass, the vector of the longest diameter should be used to determine the perpendicular vector for the maximal short axis diameter of the coalesced mass. Non- nodal lesions that coalesce should similarly be assessed by the longest axis diameter.
- If the CT/MRI slice thickness used is >5 mm, the minimum size of measurable disease at baseline should be twice the slice thickness of the baseline scan.
- Tumor lesions selected for fresh screening biopsy should not be selected as TLs, unless imaging occurred at least approximately 2 weeks after biopsy, allowing time for healing.

RECIST 1.1 Non-Target Lesion Selection at Baseline:

All other lesions, including non-measurable lesions and surplus measurable lesions not recorded as TLs should be identified as NTLs at baseline. Measurements of these lesions are not required, but the presence or absence of each should be noted throughout follow-up.

Evaluation of Tumor Response and Progression

RECIST 1.1 target lesion assessment at follow-up

This section defines the criteria used to determine objective tumor visit response for RECIST 1.1-defined TLs. The imaging modality, location, and scan date of each TL identified

previously at baseline should be documented at follow-up visits with the long axis diameter for non-nodal lesions or short axis diameter for lymph node lesions. All measurements should be recorded in millimeters. The sum of the diameters for all TL at each follow-up visit will be compared to the baseline sum of diameters (for response or SD) or to the smallest prior (nadir) sum of diameters (for progression).

Special cases for target lesion assessment at follow-up:

- If a lesion has completely disappeared, the diameter should be recorded as 0 mm. If a lesion appears in the same location on a subsequent scan, it will be recorded as an NL.
- If a TL splits into 2 or more parts, then record the sum of the diameters of those parts.
- If 2 or more TLs merge, then the sum of the diameters of the combined lesion should be recorded for 1 of the lesions and 0 mm recorded for the other lesion(s). If the merged TLs are non-nodal lesions, record the long axis diameter of the merged lesion. If pathologic lymph nodes coalesce and are no longer individually separable within a conglomerate mass, the vector of the longest diameter of the coalesced mass should be used to determine the perpendicular vector for the maximal short axis diameter.
- If a TL is believed to be present and is faintly seen but too small to measure, a default value of 5 mm should be assigned. If an accurate measure can be given, this should be recorded, even if it is below 5 mm.
- If a TL cannot be measured accurately due to it being too large, provide an estimate of the size of the lesion. The choice of 'Too large to measure' in the case report form will trigger an overall visit response of PD.
- When a TL has had any intervention eg, definitive radiotherapy, embolization, surgery, transarterial chemoembolization, etc, during the study, the size of the TL should still be provided where possible and the intervention recorded in the RECIST 1.1 case report form for the current imaging visit and all subsequent visits. If a TL has been completely removed (surgery) or disappears, the longest diameter should be recorded as 0 mm.

Table 30 RECIST 1.1 evaluation of target lesions

Complete response (CR)	Disappearance of all TLs since baseline. Any pathological lymph nodes selected as TLs must have a reduction in short axis diameter to <10 mm.
Partial response (PR)	At least a 30% decrease in the sum of the diameters of TL, taking as reference the baseline sum of diameters
Stable disease (SD)	Neither sufficient decrease in sum of diameters to qualify for PR nor sufficient increase to qualify for PD
Progression of disease (PD)	At least a 20% increase in the sum of diameters of TLs, taking as reference the smallest previous sum of diameters (nadir) – this includes the baseline sum if that is the smallest on study. In addition to the relative increase of 20%, the sum must also demonstrate an absolute increase of at least 5 mm from nadir.
Not evaluable (NE)	Only relevant if any of the TLs at follow-up were not assessed or not evaluable (e.g. missing anatomy) or had a lesion intervention at this visit. Note: if the sum of diameters meets the PD criteria, PD overrides not evaluable as a TL response
Not applicable (NA)	Only relevant if no TLs present at baseline

TL Target lesion.

RECIST 1.1 Non-Target Lesion Assessment at Follow-Up

All other lesions (or sites of disease) not recorded as TL should be identified as NTL at baseline. Measurements are not required for these lesions, but their status should be followed at subsequent visits. At each visit an overall assessment of the NTL response should be recorded by the Investigator.

To achieve ‘unequivocal progression’ on the basis of NTLs, there must be an overall level of substantial worsening in non-target disease such that, even in presence of SD or PR in TLs, the overall tumor burden has increased sufficiently to merit unequivocal progression by NTLs. A modest ‘increase’ in the size of one or more NTLs is usually not sufficient to qualify for unequivocal progression status. The designation of overall progression solely on the basis of change in non-target disease in the face of SD or PD of target disease will therefore be extremely rare.

Table 31 RECIST 1.1 evaluation of non-target lesions

Complete response (CR)	Disappearance of all NTLs since baseline. All lymph nodes must be non-pathological in size (<10 mm short axis).
Non CR/non PD	Persistence of one or more NTL.
Progression (PD)	Unequivocal progression of existing NTLs. Unequivocal progression may be due to an important progression in one lesion only or in several lesions. In all cases the progression MUST be clinically significant for the physician to consider changing (or stopping) therapy.
Not evaluable (NE)	Only relevant when one or some of the NTLs were not assessed and, in the Investigator's opinion, they are not able to provide an evaluable overall NTL assessment during the visit.<br>Note: for patients without TLs at baseline, this is relevant if any of the NTLs were not assessed at this visit and the progression criteria have not been met.
Not applicable (NA)	Only relevant if no NTLs present at baseline

PD Progression of disease; NTL Non-target lesion; TL Target lesion.

RECIST 1.1 New Lesion Identification at Follow-Up

Details including the imaging modality, the date of scan, and the location of any NLs will be recorded in the case report form. The presence of 1 or more NLs is assessed as progression. The finding of an NL should be unequivocal: ie, not attributable to differences in scanning technique, change in imaging modality, or findings thought to represent something other than tumor. If an NL is equivocal, for example because of its small size, the treatment and tumor assessments should be continued until the previously (pre-existing) new lesion has been assessed as unequivocal at a follow-up visit, and then the progression date should be declared using the date of the initial scan when the NL first appeared.

A lesion identified at a follow-up assessment in an anatomical location that was not scanned at baseline is considered a NL and will indicate PD.

RECIST 1.1 Evaluation of Overall Visit Response at Follow-Up

Derivation of overall visit response as a result of the combined assessment of TLs, NTLs, and NLs uses the algorithm shown in Table 32.

Table 32 RECIST 1.1 overall visit response

Target lesions	Non-target lesions	New lesions	Overall visit response
CR	CR	No	CR
CR	NA	No	CR
NA	CR	No	CR
CR	Non-CR/non-PD	No	PR
CR	NE	No	PR
PR	Non-PD, non-NE, or non-NA	No	PR
SD	Non-PD, non-NE, or non-NA	No	SD
NA	Non-CR/non-PD	No	SD (Non-CR/non-PD[a])
NE	Non-PD, non-NE, or non-NA	No	NE
NA	NE	No	NE
NA	NA	No	NED
PD	Any	Yes or No	PD
Any	PD	Yes or No	PD
Any	Any	Yes	PD

[a] Non-CR/non-PD for overall response if only NTLs (no TLs) are present at baseline.

Note: An overall assessment of CR (all other disease disappears/reverts to normal) would be changed to PR if ascites remains present radiologically.

CR Complete response; PR Partial response; SD Stable disease; PD Progression of disease; NA Not applicable (only relevant if there were no target lesions at baseline or no non-target lesions at baseline); NE Not evaluable; NED No evidence of diseases (only relevant if there were neither target lesions nor non-target lesions at baseline); NTL Non-target lesion; TL Target lesion.

The following overall visit responses are possible depending on the extent of tumor disease at baseline:

- For patients with TLs (at baseline): CR, PR, SD, PD, or NE
- For patients with NTLs only (at baseline): CR, Non-CR/Non-PD, PD, or NE
- For patients with no disease at baseline: no evidence of disease (available as an option in the electronic case report form), PD, or NE

Evaluation of Scans Subsequent to RECIST 1.1-Defined Progression

A follow-up scan is requested at least [redacted] after [redacted] and no longer than the next regularly scheduled imaging visit. The follow-up scans provide additional information to the Investigator for patient management and further treatment decisions, and since the published RECIST 1.1 guidelines (Eisenhauer et al 2009)

do not provide guidance on how to assess scans acquired after RECIST 1.1-defined PD, supplemental instructions for Investigators on how to evaluate these follow-up scans are provided below. A subsequent follow-up scan would be considered as having Progressive Disease if any of the following criteria are met:

- [redacted] increase and at least a [redacted] in the [redacted] compared with the [redacted] of [redacted] at [redacted], and a further increase of [redacted] in the [redacted] at the follow-up scan timepoint compared with [redacted] at the [redacted]
- [redacted] of [redacted] at the [redacted] timepoint compared with the [redacted]
- [redacted] of [redacted] at the [redacted] timepoint compared with the first instance of [redacted]
- Additional [redacted] at the [redacted] timepoint

Reference:

Eisenhauer et al 2009

Eisenhauer EA, Therasse P, Bogaerts J, Schwartz LH, Sargent D, Ford R, et al. New response evaluation criteria in solid tumors: revised RECIST guideline (version 1.1). Eur J Cancer 2009;45(2):228-47.

Appendix G International Airline Transportation Association (IATA) 6.2 guidance document

Labelling and shipment of biohazard samples

International Airline Transportation Association (IATA) classifies biohazardous agents into 3 categories. For transport purposes the classification of infectious substances according to risk groups was removed from the Dangerous Goods Regulations (DGR) in the 46th edition (2005). Infectious substances are now classified either as Category A, Category B or Exempt. There is no direct relationship between Risk Groups and categories A and B.

Category A Infectious Substances are infectious substances in a form that, when exposure to it occurs, is capable of causing permanent disability, life-threatening or fatal disease in otherwise healthy humans or animals. Category A pathogens are eg, Ebola, Lassa fever virus:

- are to be packed and shipped in accordance with IATA Instruction 602.

Category B Infectious Substances are infectious Substances that do not meet the criteria for inclusion in Category A. Category B pathogens are eg, Hepatitis A, B, C, D, and E viruses, Human immunodeficiency virus (HIV) types 1 and 2. They are assigned the following UN number and proper shipping name:

- UN 3373 – Biological Substance, Category B
- are to be packed in accordance with UN3373 and IATA 650

Exempt - all other materials with minimal risk of containing pathogens

- Clinical study samples will fall into Category B or exempt under IATA regulations
- Clinical study samples will routinely be packed and transported at ambient temperature in IATA 650 compliant packaging
- Biological samples transported in dry ice require additional dangerous goods specification for the dry-ice content
- IATA compliant courier and packaging materials should be used for packing and transportation and packing should be done by an IATA certified person, as applicable
- Samples routinely transported by road or rail are patient to local regulations which require that they are also packed and transported in a safe and appropriate way to contain any risk of infection or contamination by using approved couriers and packaging / containment materials at all times. The IATA 650 biological sample containment standards are encouraged wherever possible when road or rail transport is used.

Appendix H National Institute of Allergy and Infectious Disease and Food Allergy and Anaphylaxis Network guidance for anaphylaxis diagnosis

The National Institute of Allergy and Infectious Disease and Food Allergy and Anaphylaxis Network define anaphylaxis as a serious allergic reaction that is rapid in onset and may cause death. They recognize 3 categories of anaphylaxis, with criteria designated to capture from 80% of cases (category 1) to >95% of all cases of anaphylaxis (for all 3 categories).

Acute onset of an illness (minutes to several hours) with involvement of the skin, mucosal tissue, or both (eg, generalized hives, pruritus or flushing, swollen lips-tongue-uvula) AND AT LEAST ONE OF THE FOLLOWING:

a. Respiratory compromise (eg, dyspnea, wheeze-bronchospasm, stridor, reduced peak expiratory flow [PEF], hypoxemia)
b. Reduced blood pressure (BP) or associated symptoms of end-organ dysfunction (eg, hypotonia [collapse], syncope, incontinence)

Two or more of the following that occur rapidly after exposure to a likely allergen for that patient (minutes to several hours):

a. Involvement of the skin-mucosal tissue (eg, generalized hives, itch-flush, swollen lips-tongue-uvula)
b. Respiratory compromise (eg, dyspnea, wheeze-bronchospasm, stridor, reduced PEF, hypoxemia)
c. Reduced BP or associated symptoms (eg, hypotonia [collapse], syncope, incontinence)
d. Persistent gastrointestinal symptoms (eg, crampy abdominal pain, vomiting)

Reduced BP after exposure to known allergen for that patient (minutes to several hours):

a. Infants and children: low systolic BP (age-specific) or greater than 30% decrease in systolic BP
b. Adults: systolic BP of less than 90 mmHg or greater than 30% decrease from that person’s baseline

Reference:

Sampson HA, Muñoz-Furlong A, Campbell RL, Adkinson FN Jr, Bock SA, Branum A, et al. Second symposium on the definition and management of anaphylaxis: summary report - second National Institute of Allergy and Infectious Disease/Food Allergy and Anaphylaxis Network symposium. J Allergy Clin Immunol 2006;117:391-7.

Appendix I Guidance for management of specific adverse events in studies of capivasertib was removed to an Annex

Appendix J Guidance for management of specific adverse events in studies of selumetinib was removed

Appendix K Guidance regarding potential interactions of capivasertib with concomitant medications

Drugs that may influence capivasertib pharmacokinetics

Based on results from in vitro studies, capivasertib is a substrate of cytochrome P450 (CYP)3A4, although data suggests that glucuronidation may be the major metabolic route. Co-administration of some CYP3A4 inhibitors may increase exposure to capivasertib and hence potentially affect toxicity, while CYP3A4 inducers may decrease the exposure to capivasertib and may potentially affect efficacy.

The following list (Table 33) is not intended to be exhaustive, and a similar restriction will apply to other agents that are known to modulate CYP3A4 activity. Appropriate medical judgment is required. Please contact AstraZeneca with any queries you have on this issue.

Table 33 CYP3A4-interacting medication that should be avoided or used with caution

Medication	Recommendation	Rationale
Clarithromycin<br>Cobicistat<br>Itraconazole<br>Ketoconazole<br>Nefazodone<br>Posaconazole<br>Protease inhibitors (ritonavir, saquinavir, indinavir, tipranavir, telaprevir, elvitegravir, lopinavir, nelfinavir, and boceprevir)<br>Telithromycin<br>Troleandomycin<br>Voriconazole	**Should be avoided** 2 weeks prior to capivasertib administration and for 2 days following discontinuation of capivasertib.	Potent CYP3A4 inhibitors, which may increase the capivasertib exposure.
Carbamazepine<br>Enzalutamide<br>Phenobarbital<br>Phenytoin<br>Rifabutin<br>Rifampicin<br>Mitotane<br>St John's wort	**Should be avoided** 2 weeks (3 weeks for St John's wort and 4 weeks for enzalutamide) prior to capivasertib administration.	Potent CYP3A4 inducers, which may reduce the capivasertib exposure.

Medication	Recommendation	Rationale
Aprepitant<br>Diltiazem<br>Erythromycin<br>Fluconazole<br>Verapamil	May be used with caution.[a]	Moderate CYP3A4 inhibitors which might increase the capivasertib exposure.

[a] Drugs are permitted, but caution should be exercised, and patients monitored closely for possible drug interactions. Please refer to full prescribing information for all drugs prior to co-administration with capivasertib.

Drugs that may be influenced by capivasertib

There are currently no data confirming that there are any pharmacokinetic (PK) interactions between capivasertib and CYP3A4, CYP2D6, or CYP2C9 substrates. Likewise, there are no confirmed interactions with MATE1 or OCT2 substrates. The potential interactions detailed below (Table 34) are considered on the basis of pre-clinical data and physiologically-based PK modelling. The following list is not intended to be exhaustive, and a similar restriction will apply to other agents that are known to be sensitive to CYP3A4, CYP2D6, and/or CYP2C9 metabolism and/or MATE1 or OCT2 transport and have a narrow therapeutic window. Appropriate medical judgment is required. Please contact AstraZeneca with any queries you have on this issue.

Table 34 CYP3A4, CYP2D6, or CYP2C9 substrates that should be avoided or used with caution

Medication	Recommendation	Rationale
Alfentanil<br>Atorvastatin<br>Carbamazepine<br>Cerivastatin<br>Cyclosporin<br>Diergotamine<br>Ergotamine<br>Fentanyl<br>Lovastatin<br>Simvastatin<br>Sirolimus<br>Tacrolimus	**Should be avoided** 1 week prior to capivasertib administration and for 1 week following discontinuation of capivasertib.	CYP3A4 substrates, whose exposure may be increased by capivasertib.

Medication	Recommendation	Rationale
Amitriptyline<br>Atomoxetine<br>Desipramine<br>Doxepin<br>Metoprolol<br>Nefazodone<br>Nebivolol<br>Perphenazine<br>Tolterodine<br>Trimipramine<br>Tropisetron	**Should be avoided** 1 week prior to capivasertib administration and for 1 week following discontinuation of capivasertib.	CYP2D6 substrates, whose exposure may be increased by capivasertib.
Haloperidol<br>Tramadol	**Should be avoided** 1 week prior to capivasertib administration and for 1 week following discontinuation of capivasertib.	Combined CYP3A4 and CYP2D6 substrates, whose exposure may be increased by capivasertib.
Alprazolam<br>Domperidone<br>Erythromycin<br>Felodipine<br>Isradipine<br>Midazolam<br>Methylprednisolone<br>Nifedipine<br>Pimozide<br>Quinidine<br>Sertraline<br>Tamoxifen<br>Trazodone<br>Triazolam	May be used with caution.[a]	CYP3A4 substrates, whose exposure may be increased by capivasertib.
Fluoxetine<br>Paroxetine<br>Venlafaxine	May be used with caution.[a]	CYP2D6 substrates, whose exposure may be increased by capivasertib.
Warfarin	May be used with caution.[a]	CYP2C9 substrate, whose exposure may be increased by capivasertib.

[a] Drugs are permitted, but caution should be exercised, and patients monitored closely for possible drug interactions. Please refer to full prescribing information for all drugs prior to co-administration with capivasertib.

Table 35 MATE1 and/or OCT2 transporter substrates that should be used with caution

Medication	Recommendation	Rationale
Dofetilide	May be used with caution[a]	MATE1 and OCT2 substrate with a narrow therapeutic window whose exposure may be increased by capivasertib
Metformin	See Section 8.4.6.3	MATE1 and OCT2 substrate whose exposure may be increased by capivasertib
Procainamide	May be used with caution[a]	OCT2 substrate with a narrow therapeutic window whose exposure may be increased by capivasertib

a Drugs are permitted but caution should be exercised, and patients monitored closely for possible drug interactions. Please refer to full prescribing information for all drugs prior to co-administration with capivasertib.

Guidance for statins

Atorvastatin, cerivastatin, lovastatin, and simvastatin should be avoided due to the potential for increased exposure through inhibition of CYP3A4 by capivasertib (Table 34).

Fluvastatin, pravastatin, and rosuvastatin are minimally influenced by CYP3A4 inhibitors, conveying a relatively low potential for clinically significant drug-drug interactions via this mechanism.

Capivasertib also has a potential to inhibit the organic-anion-transporting polypeptide 1B1 transporter, which is implicated in the distribution and clearance of many of the statins. The predicted increase in the area under the plasma concentration-time curve is 1.3-fold for pravastatin and 1.5-fold for rosuvastatin. It is, therefore, recommended that doses of pravastatin be capped to 40 mg once daily (QD) and rosuvastatin be capped to 10 mg QD when combined with capivasertib, including 1 week prior to capivasertib administration and for 1 week following discontinuation of capivasertib.

In summary, rosuvastatin (up to 10 mg QD), pravastatin (up to 40 mg QD), and fluvastatin are appropriate agents to be used in patients included in capivasertib studies who require statin therapy.

Additional resources

For additional inhibitors, inducers, and substrates, please refer to:

https://drug-interactions.medicine.iu.edu/Clinical-Table.aspx

- https://www.fda.gov/Drugs/DevelopmentApprovalProcess/DevelopmentResources/DrugInteractionsLabeling/ucm093664.htm

Appendix L Guidance for management of specific adverse events in studies of DS-8201a (trastuzumab deruxtecan)

If AE causality is possibly, probably, or definitely related to both durvalumab and DS-8201a, the most conservative toxicity guidance (either durvalumab or DS-8201a) should be adopted. Discussion with the Sponsor's Study Clinical Lead is recommended in such cases.

Further guidance on ILD/pneumonitis management and LVEF management is provided in Section 6.5.2.5 and below.

Treat each of the toxicities with maximum supportive care (including withholding the agent suspected of causing the toxicity if required).

If medically appropriate, dose modifications are permitted for DS-8201a. In addition, guidelines on DS-8201a dose modifications are provided in Section 6.5.2.5. In the event of toxicity that cannot be managed by following the toxicity management guidelines for DS-8201a and durvalumab, consider stopping treatment with DS-8201a.

All dose modifications should be documented with clear reasoning and documentation of the approach taken. Dose reductions are not permitted without prior agreement with the Study Clinical Lead.

All dose modifications (interruption, reduction, and/or discontinuation) (Section 6.5.2.5) should be based on the worst preceding toxicity. Specific criteria for interruption, re-initiation, dose reduction, and/or discontinuation of DS-8201a are listed in Table 27 below, which is applicable only to TEAEs that are assessed as related to use of DS-8201a by the investigator. For non-drug related TEAEs, follow standard clinical practice. Appropriate clinical experts should be consulted as deemed necessary.

If AE causality is possibly, probably, or definitely related to both durvalumab and DS-8201a, the most conservative toxicity guidance (either durvalumab or DS-8201a) should be adopted. Discussion with the Sponsor's Study Clinical Lead is recommended in such cases. All confirmed or suspected COVID-19 infection events must be recorded in the eCRF as AE. Please refer to Appendix O for additional information on dose modification.

Table 36 Toxicity management guidelines for DS-8201a

**Worst toxicity CTCAE v4.03 Grade (unless otherwise specified)**	**Management guidelines for DS-8201a**
No toxicity	Maintain dose and schedule
**Infusion-related reaction**	
Grade 1<br>(Mild transient reaction; infusion interruption not indicated; intervention not indicated)	If infusion related reaction (such as fever and chills, with and without nausea/vomiting, pain, headache, dizziness, dyspnoea, and/or hypotension) is observed during administration, the infusion rate should be reduced by 50%, and subjects should be closely monitored.<br>If no other reactions appear, the subsequent infusion rate could be resumed at the initial planned rate.
Grade 2<br>(Therapy or infusion interruption indicated but responds promptly to symptomatic treatment (eg, antihistamines, nonsteroidal anti-inflammatory drugs (NSAIDs), narcotics, and/or IV fluids); prophylactic medications indicated for ≤24 hrs)	Administration of DS-8201a should be interrupted and symptomatic treatment started (eg, antihistamines, NSAIDs, narcotics, and/or IV fluids).<br>If the event resolves or improves to Grade 1, infusion can be restarted at a 50% reduced infusion rate.<br>Subsequent administrations should be conducted at the reduced rate.
Grade 3 or 4<br>Prolonged (eg, not rapidly responsive to symptomatic medication and/or brief interruption of infusion), recurrence of symptoms following initial improvement, hospitalization indicated for clinical sequelae or life-threatening consequences, and urgent intervention indicated	Administration of DS-8201a should be discontinued immediately and permanently.<br>Urgent intervention indicated. Antihistamines, steroids, epinephrine, bronchodilators, vasopressors, intravenous fluid therapy, oxygen inhalation, etc, should be administered.
**Haematologic toxicity** (if supportive therapy fails [as clinically indicated and according to local practice], consider additional Toxicity Management Guidelines as below)<br>For any Grade 4 hematological toxicity with significant clinical symptoms that does not resolve with treatment within 4 weeks, resuming the IMP may be possible if the toxicity resolves, in consultation with the study physician.	
**Neutrophil count decreased and/or white blood cell count decreased**	
Grade 3	Delay dose until resolved to ≤Grade 2, then maintain dose
Grade 4	Delay dose until resolved to ≤Grade 2, then Reduce dose 1 level
Febrile neutropenia (absolute neutrophil count $<1 \times 10^9$/L, fever >38.3°C, or a sustained temperature of ≥38 °C for more than 1 hour)	Delay dose until resolved, then Reduce dose by 1 level

Table 36 Toxicity management guidelines for DS-8201a

Worst toxicity CTCAE v4.03 Grade (unless otherwise specified)	Management guidelines for DS-8201a
**Lymphocyte count decreased**	
Grade 1 to Grade 3 lymphopenia	No dose modification
Grade 4<br>($<0.2 \times 10^9$/L)	Delay dose until resolved to ≤Grade 2:<br>– If resolved in ≤14 days from day of onset, then maintain dose<br>– If resolved in >14 days from day of onset, then reduce dose 1 level
**Anaemia**	
Grade 3<br>(Haemoglobin <8.0 g/dL); transfusion indicated	Delay dose until resolved to ≤Grade 2, then maintain dose
Grade 4<br>Life threatening consequences urgent intervention indicated	Delay dose until resolved to ≤Grade 2, then reduce dose 1 level
**Platelet count decreased**	
Grade 3<br>(platelets <50 to 25 × 10^9/L)	Delay dose until resolved to ≤Grade 1:<br>– If resolved in ≤7 days from day of onset, then maintain dose<br>– If resolved in >7 days from day of onset, then reduce dose 1 level
Grade 4<br>(platelets <25 × 10^9/L)	Delay dose until resolved to ≤Grade 1, then reduce dose 1 level
**Cardiac toxicity**	
Symptomatic CHF	Discontinue subject from study treatment
Decrease in LVEF 10% to 20% (absolute value), but LVEF >45%	Continue treatment with DS-8201a
LVEF 40% to ≤45% and decrease is <10% (absolute value) from baseline	Continue treatment with DS-8201a<br>Repeat LVEF assessment within 3 weeks
LVEF 40% to ≤45% and decrease is 10% to 20% (absolute value) from baseline	Interrupt DS-8201a dosing<br>Repeat LVEF assessment within 3 weeks.<br>If LVEF has not recovered to within 10% (absolute value) from baseline, discontinue subject from study treatment<br>If LVEF recovers to within 10% from baseline, resume study drug treatment
LVEF <40% or >20% (absolute value) drop from baseline	Interrupt DS-8201a dosing.<br>Repeat LVEF assessment within 3 weeks.<br>If LVEF <40% or >20% drop from baseline is confirmed, discontinue subject from study treatment<br>If LVEF has recovered to > 40% and decrease is < 20% from baseline, follow appropriate guidance above.

Table 36 Toxicity management guidelines for DS-8201a

Worst toxicity CTCAE v4.03<br>Grade (unless otherwise specified)	Management guidelines for DS-8201a
**Electrocardiogram QTc Prolonged**	
Grade 3<br>(Average QTc ≥ 501 ms or > 60 ms change from baseline)	Delay dose until resolved to ≤Grade 1 (corrected QT ≤480 ms), then determine if another medication the subject was taking may be responsible and can be adjusted or if there are any changes in serum electrolytes that can be corrected, then if attributed to DS-8201a, reduce dose 1 level
Grade 4<br>(Torsade de pointes or polymorphic ventricular tachycardia or signs/symptoms of serious arrhythmia)	Discontinue subject from study treatment
**Pulmonary Toxicity**	Any evidence of ILD/pneumonitis should be promptly investigated.<br>Work-up of suspected ILD/pneumonitis:<br>If a subject develops radiographic changes potentially consistent with ILD/pneumonitis or develops new or worsening pulmonary or other related signs/symptoms such as dyspnoea (resting or exertional), cough fever, unexplained fatigue or decrease in oxygen saturation, rule out ILD/pneumonitis.<br>Evaluations should include:<br>• High resolution CT (HRCT) (preferred)/CT; the same modality should be employed as the baseline scan to allow for direct comparison of scans<br>• Pulmonologist consultation (infectious disease consultation as clinically indicated)<br>• Bronchoscopy and bronchoalveolar lavage if clinically indicated and feasible<br>• Pulmonary function tests (including forced vital capacity (FVC) and carbon monoxide (CO) diffusing capacity) and pulse oximetry (SpO_2)<br>• Clinical laboratory tests:<br>– COVID-19 test<br>– Arterial blood gases if clinically indicated<br>– Blood culture, blood cell count (CBC), differential white blood cell count (WBC), C-reactive protein (CRP),<br>– One blood sample collection for PK analysis as soon as ILD/pneumonitis is suspected, if feasible<br>Other tests could be considered, as needed including PCP screening (eg, beta-D-glucan test, BAL) especially in patients with 1 or more of the following risk factors: lymphopenia, long-term/intermittent steroid use, brain metastases, and chronic lung diseases.<br>Appropriate management for ILD/pneumonitis should be instituted promptly as per management guideline below when ILD is suspected.

Table 36 Toxicity management guidelines for DS-8201a

**Worst toxicity CTCAE v4.03**<br>**Grade (unless otherwise specified)**	**Management guidelines for DS-8201a**
	If the AE is confirmed to have an aetiology other than treatment-related ILD/pneumonitis, follow the management guidance outlined in the "Other Non-Laboratory Adverse Events" dose modifications.<br>If another aetiology for the AE cannot be identified and it could be related to DS-8201a, then follow the ILD/pneumonitis management guidance as outlined below.<br>Consideration should be given to the possibility of an alternate etiology occurring concurrently with Drug-induced-ILD.<br>Whenever corticosteroids have been administered the dose should be tapered gradually over at least 4 weeks even if an alternate etiology is confirmed.<br>All events of ILD/pneumonitis regardless of severity or seriousness will be followed until resolution.
Grade 1	Management:<br>• Monitor and closely follow up in 2 to 7 days for onset of clinical symptoms and pulse oximetry then weekly as indicated<br>• Consider follow-up imaging in 1 to 2 weeks (or as clinically indicated)<br>• Consider starting systemic steroids (eg, at least 0.5 mg/kg/day prednisone or equivalent) until improvement, followed by gradual taper over at least 4 weeks<br>• If worsening of diagnostic observations despite initiation of corticosteroids, then follow Grade 2 guidelines*<br><br>Dose modification:<br>The administration of DS-8201a must be interrupted. DS-8201a can be restarted only if the event is fully resolved to Grade 0:<br>– If resolved in ≤28 days from day of onset, then maintain dose<br>– If resolved in >28 days from day of onset, then reduce dose 1 level<br>If the event Grade 1 ILD/pneumonitis occurs beyond cycle day 22 and has not resolved within 18 weeks from the last infusion, the drug should be discontinued.<br>*If a subject is asymptomatic, then the subject should still be considered as Grade 1 even if steroid treatment is given.

Table 36 Toxicity management guidelines for DS-8201a

**Worst toxicity CTCAE v4.03<br>Grade (unless otherwise specified)**	**Management guidelines for DS-8201a**
Grade 2	Dose Modification:<br>Permanently discontinue subject from study treatment.<br><br>Management:<br>Promptly start and treat with systemic steroids (eg, at least 1 mg/kg/day prednisone or equivalent) for at least 14 days, followed by **gradual taper** over at least 4 weeks<br>• Monitor symptoms closely<br>• Re-image as clinically indicated<br>• If worsening or no improvement in clinical or diagnostic observations in 3 to 5 days,<br>– Consider increasing dose of steroids (eg, 2 mg/kg/day prednisone or equivalent) and switching treatment administration to intravenous (eg, methylprednisolone)<br>– Re-consider additional work-up for alternative etiologies as described above<br>– Escalate care as clinically indicated
Grade 3 or 4	Dose modification:<br>Permanently discontinue subject from study treatment.<br><br>Management:<br>• Hospitalisation required<br>• Promptly initiate empiric high-dose methylprednisolone IV treatment (eg, 500-1000 mg/day for 3 days), followed by at least 1.0 mg/kg/day of prednisone (or equivalent) for at least 14 days, followed by **gradual taper** over at least 4 weeks<br>• Re-image as clinically indicated<br>• If still no improvement within 3 to 5 days,<br>– Re-consider additional work-up for alternative etiologies as described above<br>– Consider other immunosuppressants and/or treat per local practice
**Ocular**	
Grade 3	Delay dose until resolved to ≤Grade 1:<br>– If resolved in ≤7 days from day of onset, then maintain dose<br>– If resolved in >7 days from day of onset, then reduce dose 1 level
Grade 4	Discontinue subject from study treatment

Table 36 Toxicity management guidelines for DS-8201a

Worst toxicity CTCAE v4.03<br>Grade (unless otherwise specified)	Management guidelines for DS-8201a
**Blood creatinine increased**	
Grade 3<br>(>3.0 to 6.0 × ULN)	Delay dose until resolved to ≤Grade 2 or baseline, then reduce dose 1 level
Grade 4<br>(>6.0 × ULN)	Discontinue subject from study treatment
**Hepatic toxicity**	
**Aspartate aminotransferase (AST) or alanine aminotransferase (ALT) with simultaneous blood bilirubin increased**	
AST/ALT ≥3.0 x ULN with simultaneous total bilirubin ≥2.0 × ULN	Delay study medication until drug-induced liver injury can be ruled out.<br>If drug-induced liver injury is ruled out, the subject should be treated accordingly, and resumption of study drug may occur after discussion between the Investigator and Sponsor.<br>If drug-induced liver injury cannot be ruled out from diagnostic workup, permanently discontinue study treatment.<br>Monitor AST/ALT and total bilirubin twice weekly until resolution or return to baseline.
**Aspartate aminotransferase (AST) or alanine aminotransferase (ALT**	
Grade 2<br>(>3.0 to 5.0 × ULN if baseline was normal; >3.0 to 5.0 × baseline if baseline was abnormal)	No action for Grade 2 AST/ALT
(>5.0 to 20.0 × ULN if baseline was normal; >5.0 to 20.0 × baseline if baseline was abnormal)<br>In subjects without liver metastases and subjects with liver metastases and baseline level ≤3 × ULN	Repeat testing within 3 days. Delay dose until resolved to ≤Grade 1 if baseline ≤3 x ULN, otherwise delay dose until resolved to ≤ baseline, then:<br>– If resolved in ≤7 days from day of onset, then maintain dose<br>– If resolved in >7 days from day of onset, then reduce dose 1 level
(>8.0 to 20.0 × ULN if baseline was normal; >8.0 to 20.0 × baseline if baseline was abnormal)<br>In subjects with liver metastases, if the baseline level was >3 × ULN	Repeat testing within 3 days. Delay dose until resolved to ≤ baseline level:<br>– If resolved in ≤7 days from day of onset, then maintain dose<br>– If resolved in >7 days from day of onset, then reduce dose 1 level
Grade 4<br>(>20 × ULN if baseline was normal; >20.0 × baseline if baseline was abnormal)	Discontinue subject from study treatment

Table 36 Toxicity management guidelines for DS-8201a

Worst toxicity CTCAE v4.03<br>Grade (unless otherwise specified)	Management guidelines for DS-8201a
**Blood bilirubin increased**	
Grade 2<br>(>1.5 to 3.0 × ULN if baseline was normal; >1.5 to 3.0 × baseline if baseline was abnormal)	If no documented Gilbert's syndrome or liver metastases at baseline, delay dose until resolved to ≤Grade 1:<br>– If resolved in ≤7 days from day of onset, then maintain dose<br>– If resolved in >7 days from day of onset, then reduce dose 1 level<br>If documented Gilbert's syndrome or liver metastases at baseline, continue study treatment
Grade 3<br>(>3.0 to 10.0 × ULN if baseline was normal; >3.0 to 10.0 × baseline if baseline was abnormal)	If no documented Gilbert's syndrome or liver metastases at baseline, repeat testing within 3 days. Delay dose until resolved to ≤Grade 1:<br>– If resolved in ≤7 days from day of onset, then reduce dose 1 level<br>– If resolved in >7 days from day of onset, then discontinue DS-8201a<br><br>If documented Gilbert's syndrome or liver metastases at baseline, repeat testing within 3 days. Delay dose until resolved to ≤Grade 2:<br>– If resolved in ≤7 days from day of onset, then reduce dose 1 level<br>– If resolved in >7 days from day of onset, then discontinue DS-8201a
Grade 4<br>(>10.0 × ULN if baseline was normal; >10.0 × baseline if baseline was abnormal)	Discontinue subject from study treatment
**Blood alkaline phosphatase increased**	
Grade 3<br>(>5.0 to 20.0 × ULN if baseline was normal; >5.0 to 20.0 × baseline if baseline was abnormal)<br>Or Grade 4<br>(>20.0 × ULN if baseline was normal; >20.0 × baseline if baseline was abnormal)	No modification unless determined by the Investigator to be clinically significant or life-threatening.
**<u>Gastrointestinal</u>**	
**Nausea**	
Grade 3<br>Inadequate oral caloric or fluid intake, tube feeding, TPN, or hospitalization indicated	Delay dose until resolved to ≤Grade 1:<br>– If resolved in ≤7 days from day of onset, then maintain dose<br>– If resolved in >7 days from day of onset, then reduce dose 1 level

Table 36 Toxicity management guidelines for DS-8201a

**Worst toxicity CTCAE v4.03<br>Grade (unless otherwise specified)**	**Management guidelines for DS-8201a**
**Diarrhoea/Colitis**	
Grade 3<br>Increase of ≥ 7 stools per day over baseline, hospitalization indicated, severe increase in ostomy output compared to baseline; limiting self-care ADL<br>Severe abdominal pain, peritoneal signs	Delay dose until resolved to ≤Grade 1:<br>– If resolved in ≤3 days from day of onset, then maintain dose<br>– If resolved in >3 days from day of onset, then reduce dose 1 level
Grade 4<br>Life-threatening consequences, urgent intervention indicated	Discontinue subject from study treatment
**Other laboratory adverse events**	
Severe or medically significant but not immediately life-threatening, hospitalization or prolongation of existing hospitalization indicated, and limiting self-care ADL	Delay dose until resolved to ≤Grade 1 or baseline level:<br>– If resolved in ≤7 days from day of onset, then maintain dose<br>– If resolved in >7 days from day of onset, then reduce dose 1 level
Life-threatening consequences and urgent intervention indicated	Discontinue subject from study treatment
**Other non-laboratory adverse events**	
Grade 3	Delay dose until resolved to ≤Grade 1 or baseline:<br>– If resolved in ≤7 days from day of onset, then maintain dose<br>– If resolved in >7 days from day of onset, then reduce dose 1 level
Grade 4	Discontinue subject from study treatment

All dose modifications should be based on the worst preceding toxicity.

CTCAE: Common Terminology Criteria for Adverse Events; IMP Investigational medicinal product.

Appendix M Guidance for management of specific adverse events of Dato-DXd (datopotamab deruxtecan) was removed to an Annex

Appendix N Durvalumab weight-based dose calculation

For durvalumab dosing done depending on patient weight. Weight-based dosing should be utilized for patients ≤30 kg (for Arms 1 through 5 only):

1 Dose: X mg/kg

2 Patient weight: Y kg

3 Dose for patient: XY mg = X (mg/kg) × Y (kg)

4 Dose to be added into infusion bag: Dose (mL) = XY mg/50 (mg/mL)

where 50 mg/mL is durvalumab nominal concentration.

The corresponding volume of durvalumab should be rounded to the nearest tenth mL (0.1 mL).

5 The number of vials required for dose preparation is the next greatest whole number of vials from the following formula:

Number of vials = Dose (mL)/10.0 (mL/vial)

Example:

1 Dose: 20 mg/kg for Arms 1 through 5

2 Patient weight: 30 kg

3 Dose for patient (eg, patients in Arms 1 through 5: 600 mg = 20 (mg/kg) × 30 (kg)

4 Dose to be added into infusion bag:

Dose (mL) = 600 mg / 50 (mg/mL) = 12.0 mL

5 The number of vials required for dose preparation:

Number of vials = 12.0 (mL) / 10.0 (mL/vial) = 2 vials

Appendix O Instructions Related to COVID-19 (For Arms 6, 7, and 8 Only)

Benefit Risk Consideration for COVID-19

The emergence of the coronavirus disease 2019-nCoV (COVID-19) presents a potential safety risk for patients. Several risk mitigation factors have been implemented in this study. Notably, the eligibility criteria will exclude participants with COVID-19 infections (Section 5.2).

Moreover, with the outbreak of COVID-19, there is the potential for increased use of chloroquine and hydroxychloroquine to treat severely symptomatic patients or even for prophylactic use. Chloroquine and hydroxychloroquine have shown in vitro to substantially affect the pH of the lysosome, a key intracellular compartment involved in the trafficking and payload release of DS-8201a and Dato-DXd. As it is unknown whether chloroquine/hydroxychloroquine may affect the safety and efficacy of DS-8201a or Dato-DXd, to be eligible for this clinical trial, use of chloroquine and hydroxychloroquine treatment must be completed 14 days prior to the first dose of DS-8201a or Dato-DXd (Section 5.1). During study treatment, chloroquine and hydroxychloroquine are considered prohibited concomitant medications. However, in case treatment with chloroquine or hydroxychloroquine treatment is absolutely required for COVID-19, study treatment must be interrupted. After chloroquine or hydroxychloroquine is administered for COVID-19, then a washout period of at least 14 days is required before restarting study treatment.

Lastly, due to the potential overlapping impact of DS-8201a or Dato-DXd and COVID-19 on the lung, the Sponsor has also provided in this Appendix a dose modification and management plan for patients with confirmed or suspected COVID-19 who are being treated with DS-8201a or Dato-DXd.

With these measures in place, it is considered the anticipated potential benefits for the patients enrolled in this study outweigh the potential risks.

Inclusion criteria

- Has adequate treatment washout period before randomization/enrollment, defined as:
- Chloroquine/Hydroxychloroquine: ≥ 14 days

Prior and concomitant medications

Concomitant treatment with chloroquine or hydroxychloroquine is not allowed during the study treatment. If treatment with chloroquine or hydroxychloroquine treatment is absolutely required for COVID-19, study treatment must be interrupted. If chloroquine or hydroxychloroquine is administered, then a washout period of at least 14 days is required before restarting study treatment.

Dose modification criteria

All confirmed or suspected COVID-19 infection events must be recorded in the eCRF as AE. Dose modifications will be based on the worst CTCAE grade. All interruptions or modifications must be recorded on the AE and drug exposure eCRFs. Please use CTCAE v4.03 general grading criteria to evaluate COVID-19.

Dose modification criteria for suspected or confirmed COVID-19

If COVID-19 infection is suspected, delay DS-8201a or Dato-DXd and rule out COVID-19 per local guidance.

- If COVID-19 is ruled out, follow study protocol.
- If COVID-19 is confirmed or diagnosis is suspected after evaluation, manage COVID-19 per local guidance until recovery of COVID-19 defined as no signs/symptoms, at least 1 negative reverse transcription-polymerase chain reaction (RT-PCR) test result, and nearly or completely resolved chest CT findings. Then follow below dose modifications:

COVID-19 Severity	Toxicity Management Guidance
Grade 1<br>(asymptomatic incidental COVID-19 test positive on screening)	Resume DS-8201a or Dato-DXd and durvalumab at the same dose after at least 1 negative RT-PCR test result, and nearly or completely resolved chest CT findings
Grade 2<br>(moderate symptoms; oral intervention indicated)	One negative RT-PCR test result, no signs/symptoms<br>Imaging outcome:<br>– Maintain same dose if chest CT findings are completely resolved<br>– Reduce dose 1 level if chest CT findings are nearly resolved<br>– Durvalumab can be resumed
Grade 3<br>(dyspnea and pneumonia symptoms; hospitalization indicated)	One negative RT-PCR test result, no signs/symptoms<br>Imaging outcome:<br>– Reduce dose 1 level if chest CT findings are completely resolved<br>– Durvalumab can be resumed<br>– Otherwise, discontinue all study treatments
Grade 4<br>(life-threatening consequences; urgent intervention indicated)	Discontinue all study treatments

Note: If PCR testing is not available, the subject must not have any sign/symptoms for at least 2 weeks, in addition to meeting the requirement for chest CT imaging.

Any adverse event that is not related or possibly not related to study treatment, the study treatment can be delayed for the length of time required until it is safe to resume treatment. The maximum delay of 18 weeks for DS-8201a and 84 days for the Dato-DXd is only

applicable for adverse events causally related to study treatment and not applicable to isolated COVID-19 infections.

Durvalumab can be restarted with DS-8201a or Dato-DXd when appropriate and by following the guidance above.

- Closely monitor signs/symptoms after restarting DS-8201a or Dato-DXd, initially with a phone call every 3 days for the first week, and then with a weekly phone call thereafter, for a total of 6 weeks.
- If an event is suspected to be drug-related ILD/pneumonitis, treatment with the study drug should be delayed pending further evaluations, and this event should be managed as per the protocol. ILD/pneumonitis management guideline for DS-8201a (Appendix L) or Dato-DXd (Annex document to this CSP) should be followed.

If patients have a delay in dosing that results in one or more cycles of both durvalumab and DS-8201a/Dato-DXd being skipped, then when treatment is restarted, the next dose should be marked in the eCRF as the subsequent cycle with action taken of dose delayed due to an adverse event.

Blood sample for DS-8201a or Dato-DXd PK analysis in patients who received chloroquine/hydroxychloroquine

PK blood draws will be taken at the following timepoints for patients in the DS-8201a or Dato-DXd arms during the chloroquine or hydroxychloroquine treatment period:

- Before the first dose of chloroquine/hydroxychloroquine dosing
- Before chloroquine/hydroxychloroquine dosing on Day 3 or 4
- End of chloroquine/hydroxychloroquine treatment
- Pre-dose on the day of restarting DS-8201a or Dato-DXd treatment. If DS-8201a or Dato-DXd is restarted, then the normal DS-8201a or Dato-DXd PK blood sample collection can resume

Appendix P Changes related to mitigation of study disruptions due to Cases of civil crisis, natural disaster, or public health crisis

Note: The changes below should be implemented only during study disruptions due to any of or a combination of civil crisis, natural disaster, or public health crisis (eg, during quarantines and resulting site closures, regional travel restrictions, and considerations if site personnel or study patients become infected with SARS-CoV-2 or similar pandemic infection), during which patients may not wish to or may be unable to visit the study site for study visits. These changes should only be implemented if allowable by local/regional guidelines and following agreement from the Sponsor.

P-1 Reconsent of study patients during study interruptions

During study interruptions, it may not be possible for the patients to complete study visits and assessments on site, and alternative means for carrying out the visits and assessments may be necessary (eg, remote visits). Reconsent should be obtained for the alternative means of carrying out visits and assessments and should be obtained prior to performing the procedures. Local and regional regulations and/or guidelines regarding reconsent of study patients should be checked and followed. Reconsent may be verbal if allowed by local and regional guidelines. Visiting the study sites for the sole purpose of obtaining reconsent should be avoided.

P-2 Telemedicine visit to replace on-site visit (where applicable)

In this appendix, the term “telemedicine visit” refers to remote contact with the patients using telecommunications technology, including phone calls, virtual or video visits, and mobile health devices.

During a civil crisis, natural disaster, or public health crisis, on-site visits may be replaced by a telemedicine visit, if allowed by local/regional guidelines. A telemedicine contact with the patients will allow adverse events and concomitant medications to be reported and documented.

P-3 Data capture during telemedicine or remote visits

Data collected during telemedicine or remote visits will be captured by the qualified health care professional (HCP) from the study site or third-party vendor service (TPV) in the source documents, or by the patients themselves.

Appendix Q [redacted] was removed

Appendix R Non-CTCAE Corneal Toxicity Severity Grading Scale

CCI [redacted]

Appendix S Abbreviations

The following abbreviations and special terms are used in this study Clinical Study Protocol.

Abbreviation or special term	Explanation
5-HT3	5-Hydroxytryptamine receptor
ADA	Anti-drug antibody
ADC	Antibody-drug conjugate
AE	Adverse event
AESI	Adverse event of special interest
AKT	Protein kinase B
ALP	Alkaline phosphatase
ALT	Alanine aminotransferase
AMP	Adenosine monophosphate
aPTT	activated partial thromboplastin time
ASCO	American Society of Clinical Oncology
ASO	Antisense oligonucleotide
AST	Aspartate aminotransferase
AUC	Area under the plasma concentration-time curve
BC	Breast cancer
BD	Twice daily
BICR	Blinded independent central review
bid	Twice daily
BoR	Best overall response
BP	Blood pressure
BRCA	Breast cancer susceptibility gene
CAP	College of American Pathologists
CAR	Chimeric antigen receptor
CD	Cluster of differentiation
cEt	Constrained ethyl
CI	Confidence interval
C_{max}	Maximum concentration
CNS	Central nervous system
COPD	Chronic obstructive pulmonary disease
COVID-19	Coronavirus disease 2019-nCoV

Abbreviation or special term	Explanation
CR	Complete response
CRC	Colorectal cancer
CRF	Case report form
CSP	Clinical Study Protocol
CSR	Clinical study report
CT	Computed tomography
CTCAE	Common Terminology Criteria for Adverse Events
ctDNA	Circulating tumor deoxyribonucleic acid
CTIS	Clinical Trials Information System
CTLA-4	Cytotoxic T-lymphocyte–associated antigen 4
CYP	Cytochrome P450
Dato-DXd	Datopotamab deruxtecan; DS-1062a
DCO	Data cutoff
DCR	Disease control rate
DES	Data entry site
DISH	Dual in situ hybridization
DKA	Diabetic ketoacidosis
DLBCL	Diffuse large B cell lymphoma
DLT	Dose-limiting toxicity
DNA	Deoxyribonucleic acid
DoR	Duration of response
DUS	Disease under study
DS8201a	Trastuzumab deruxtecan; T-DXd
EC	Ethics Committee
ECG	Electrocardiogram
ECHO	Echocardiogram
ECOG	Eastern Cooperative Oncology Group
eCRF	Electronic Case Report Form
EOT	End of treatment
ER	Estrogen receptor
ERK	Extracellular signal regulated kinase
EU	European Union
FAS	Full Analysis Set

Abbreviation or special term	Explanation
FDA	Food and Drug Administration
FDG-PET	^{18}F-Fluoro-deoxyglucose positron emission tomography
FFPE	Formalin-fixed and paraffin-embedded
FISH	Fluorescent in-situ hybridization
GCP	Good Clinical Practice
G-CSF	Granulocyte colony-stimulation factor
GI	Gastrointestinal
GMP	Good Manufacturing Practice
HbA1c	Glycosylated hemoglobin
HBcAb	Hepatitis B core antibody
HBsAg	Hepatitis B surface antigen
HBV	Hepatitis B virus
HCC	Hepatocellular carcinoma
HCV	Hepatitis C virus
HDV	Hepatitis D virus
HER2	Human epidermal growth factor receptor 2
HIPAA	Health Insurance Portability and Accountability Act
HIV	Human immunodeficiency virus
HLA	Human-leukocyte antigen
HR	Hazard ratio
HRCT	High-resolution computed tomography
IBW	Ideal body weight
IC_{50}	Half maximal inhibitory concentration
ICF	Informed consent form
ICH	International Council on Harmonisation
iCRO	imaging Contract Research Organization
IEC	Independent Ethics Committee
IFN-γ	Interferon-gamma
IgG	Immunoglobulin G
IHC	Immunohistochemistry
IL	Interleukin
ILD	Interstitial lung disease
imAE	Immune-mediated adverse event

Abbreviation or special term	Explanation
IMP	Investigational medicinal product
INR	International normalized ratio
IP	Investigational product
irAE	Immune-related adverse event
IRB	Institutional Review Board
IRR	Infusion-related reaction
IRT	Interactive Response Technology
ITT	Intention-to-treat
IV	Intravenous(ly)
LFT	Liver function test
LIMS	Laboratory Information Management System
LVEF	Left ventricular ejection fraction
mAb	Monoclonal antibody
MAPK	Mitogen-activated protein kinase
MDSC	Myeloid-derived suppressor cell
MedDRA	Medical Dictionary for Regulatory Activities
MEK	Mitogen-activated protein kinase/extracellular signal-regulated kinase (MAPK/ERK kinase)
MHC	Major histocompatibility complex
MOA	Mechanism of action
MRI	Magnetic resonance imaging
mRNA	Messenger ribonucleic acid
MSS-CRC	Microsatellite stable colorectal cancer
MTD	Maximum tolerated dose
mTOR	Mammalian target of rapamycin
MUGA	Multiple gated acquisition
muSTAT3	Murine STAT3
NCCN	National Comprehensive Cancer Network
NCI	National Cancer Institute
NE	Not evaluable
NGS	Next generation sequencing
NK	Natural killer
NL	New lesion

Abbreviation or special term	Explanation
NSCLC	Non-small cell lung cancer
NTL	Non-target lesion
OCP	oral care protocol
ORR	Objective response rate
OS	Overall survival
PD	Progression of disease
PD-1	Programmed cell death 1
PD-L1	Programmed cell death ligand 1
PD-L2	Programmed cell death ligand 2
PET	Positron emission tomography
PFS	Progression-free survival
PFT	Pulmonary function test
PFS6	Progression-free survival at 6 months following date of first dose of IP
PgR	Progesterone receptor
PI3K	Phosphoinositide-3-kinase
PIK3CA	Phosphoinositide-3-kinase, catalytic, alpha polypeptide
PK	Pharmacokinetic(s)
PKB	protein kinase B
PR	Partial response
PS	Performance status
PSSR	Project Specific Safety Requirement
PT	Prothrombin time
PTAP	Post Trial Access Program
PTT	Partial thromboplastin time
PTEN	Phosphatase and tensin homolog
q12w	Every 12 weeks
q1w	Every 1 week
q2w	Every 2 weeks
q3w	Every 3 weeks
q4w	Every 4 weeks
q8w	Every 8 weeks
QD	Once daily

Abbreviation or special term	Explanation
QTcF	QT interval corrected for heart rate using Fridericia's formula
RECIST 1.1	Response Evaluation Criteria In Solid Tumors, version 1.1
RNA	Ribonucleic acid
RP2D	Recommended Phase II dose
RT-PCR	Reverse transcription-polymerase chain reaction
RTSM	Randomisation and Trial Supply Management System
SAE	Serious adverse event
SAF	Safety Analysis Set
SAP	Statistical analysis plan
SARS-CoV-2	Severe acute respiratory syndrome coronavirus 2
SC	Subcutaneous(ly)
SCCHN	Squamous cell carcinoma of the head and neck
SoA	Schedule of Activities
SoC	Standard of care
SMQ	Standardized MedDRA Queries
CCI [redacted]	
SRC	Safety Review Committee
STAT3	Signal transducer and activator of transcription 3
T_3	Triiodothyronine
T_4	Thyroxine
TAM	Tumor-associated macrophage
TBL	Total bilirubin
TdP	Torsade de Pointes
TEAE	Treatment-emergent adverse event
TESAE	Treatment-emergent serious adverse event
TIL	Tumor-infiltrating lymphocyte
TL	Target lesion
TMB	Tumor mutational burden
TMG	Toxicity Management Guidelines
TNBC	Triple negative breast cancer
TQ	Targeted questionnaire
TROP2	Trophoblast cell surface protein 2
TSH	Thyroid-stimulating hormone

Abbreviation or special term	Explanation
ULN	Upper limit of normal
US	United States
WHO	World Health Organization
w/v	Weight to volume

Appendix T Protocol Version History

The Summary of Changes Table for the current revision is located directly before the Table of Contents.

Version 10.0, 23 Mar 2023

This amendment is considered to be substantial based on the criteria set forth in Article 10(a) of Directive 2001/20/EC of the European Parliament and the Council of the EU.

A new study arm (Arm 8) to investigate Durvalumab + Dato-DXd in patients with PD-L1 positive TNBC was added to the protocol in order to further assess safety and efficacy in patients with PD-L1 positive tumors. The following changes were implemented:

- Reference to Arm 8 was incorporated throughout the protocol, as applicable. Changed the total number of planned patients from 140 to 170, due to the addition of Arm 8: Synopsis
- Specified that Arm 8 will not have a safety run-in given that it investigates the same durvalumab + Dato-DXd combination as was evaluated in Arm 7; the combination was found to be well tolerated with no DLT reported. SRC will review safety and tolerability data in Arm 8 on an ongoing basis: Synopsis, Section 1.3, 4.1.2, 4.1.4.1.
- In order to limit the number of patients with unknown PD-L1 status undergoing screening evaluations in Arm 8, a pre-screening period, and accompanying ICF, was added to permit determination of PD-L1 expression status by local testing prior to the 28-day screening window in patients in Arm 8 who do not have a pre-existing determination of positive PD-L1 status: Sections 1.1, Schedule of Assessments Table 6, Synopsis, Sections 4.1, 4.1.2, 5.1, 5.4, 6.2.1, 8, 8.3.2, Appendix A-3.
- Changed the total number of planned patients from 140 to 170, due to the addition of Arm 8: Synopsis.
- Added inclusion criteria 18 to require a PD-L1 positive tumor in patients in Arm 8: Section 5.1.

The following amendment changes were made due to revisions of the AstraZeneca protocol template, and durvalumab or Dato-DXd standard text revisions:

- Added test for [redacted] infection at screening: Schedule of Activities Table 6, Section 8.2.1.
- Study Monitor/Physician changed to Study Clinical Lead throughout the document .
- Removed requirement for durvalumab ADA sample for durvalumab and Dato-DXd and weight documentation 6 months post treatment discontinuation: Schedule of Activities Table 7.
- Added [redacted] as a safety endpoint in Parts 1 and 2: Synopsis, Section 3.

- Replaced the designation "MAAA-1181a" with "DXd": Sections 2.2.5, 2.2.8, 2.2.9, 2.3.4.2.
- Moved IRR, including anaphylaxis, from being an important identified risk for Dato-DXd to being an identified risk: Sections 2.2.9, 2.3.5.2, 8.3.14.5.
- Added embryo-fetal toxicity as an important potential risk for Dato-DXd: Section 2.3.5.2.
- Revised the recommendations concerning the toxicity management guidelines to apply in the case of overlapping toxicities of durvalumab + Dato-DXd: Section 2.3.5.3.
- Update to clarify SRC committee attendance requirements: Section 4.1.4.3.
- Deleted a statement concerning the mean terminal half-life (T½) of Dato-DXd: Section 4.3.6.
- Inclusion criteria 10: added specification that in Arm 8, patients with [redacted] infection must have [redacted] and [redacted] or [redacted] × ULN [redacted] are present and the elevation is not attributable to [redacted] infection: Section 5.1.
- Exclusion criteria 2 and 3: updated to categorize [redacted] appropriately and add [redacted], and [redacted] as cited immune disorders: Section 5.2.
- Removed consultation with the Study Monitor/Physician concerning eligibility of patients with [redacted] disease: Section 5.2.
- Revised eligibility for patients with [redacted] and [redacted] infection: Section 5.2.
- Reduced the period of mandatory contraception following durvalumab from [redacted] months to [redacted] months: Sections 5.2, 5.3.
- Revised the acceptable highly effective methods of contraception: Section 5.3.
- Add specification that patients should avoid prolonged exposure to the sun: Section 5.3.
- Added requirement to inspect prepared durvalumab vials: Section 6.1.1.2.
- Added specification that the use of elastomeric pumps and a pneumatic tube for transport of vials or a final prepared product should be avoided: Section 6.1.1.2.
- Added specification that aseptic technique in compliance with local regulations and site requirements should be followed in the preparation of Dato-DXd for administration: Section 6.1.1.7.
- Added specification concerning recording concomitant medications administered as treatment for drug-related AESIs: Section 6.4.
- Post-treatment interval for use of a live-attenuated vaccine increased to [redacted] days: Sections 5.2 and 6.4.
- Renamed Rescue medications section as Ancillary treatment for management of immuno-oncology-related toxicities and revised language to align with updated terminology: Section 6.4.2.
- Revised the Dato-DXd dose interruption and modification toxicity management guidelines language to align with updated terminology: Section 6.5.2.6.

- Added details on required assessments of [redacted] parameters and pregnancy tests: Section 8.2.1
- Revised [redacted] according to the Dato-DXd template language: Section 8.2.9.2.
- Added [redacted] section concerning AEs based on examinations and tests: Section 8.3.7.
- Added specifications concerning the follow-up of AESIs and recording of concomitant medication for AESIs: Section 8.3.14.
- Clarified required actions for treatment modifications for specific imAEs: Section 8.4.6.1
- Included specifications concerning drug abuse and drug misuse and revised text concerning medication errors: Section 8.4.5 and Appendix B-8.
- Appendix A-6 updated to include Clinical Trial Transparency guidelines on submitting results to EU CTIS.
- Appendix F updated with latest guidelines for BICR-assessed studies.
- Appendix Q [redacted] removed to a separate document: Schedule of Assessments Tables 5 and 6, Appendix Q.
- Added specification that evaluations of [redacted] prior to infusion from Cycle [redacted] onwards can be performed up to 3 days prior to the infusion and that [redacted] should be evaluated by the PI or the delegate physician prior to the administration of IMP at each visit, and at [redacted]: Schedule of Activities Tables 5 and 6.
- Provided further specifications concerning blood pressure assessment, updated with details of vital signs monitoring guidelines for initial infusion, and removed the mid-infusion vital signs assessment for DS-8201a and Dato-DXd: Section 8.2.3.

Other amendment changes include the following:

- [redacted]
- Added specification that final DCO date may differ by arm, so that the final analysis can be conducted for an arm prior to the end of follow-up in all arms: Sections 1.1, 6.6, 8, 8.1, 8.4.2, 9.1, Synopsis.
- Indicated that the expansion of an arm from Part 1 to Part 2 will be based on [redacted] as well as based on a futility analysis utilizing a [redacted]: Synopsis.
- Inclusion criteria 6: clarified that specification concerning prior treatment applies to patients with locally advanced unresectable TNBC as well as those with metastatic TNBC, for consistency with inclusion criteria 5: Section 5.1.
- Specified that AESIs, as well as SAEs, should be collected after the DCO date in each arm, in order to ensure that all occurrences of an AESI are reported: Sections 6.6, 8.4.2.

- Specified that [redacted] is to be evaluated only in Arms [redacted] and [redacted]: Section 8.2.1.
- Rearranged text concerning collection of tumor samples for the sake of clarity: Section 8.8.1.
- Clarified for which study parts and arms PK analyses will be performed in Sections 9.2.4 and 9.3.3.
- Added details concerning planned PK analyses: Section 9.4.3.
- Clarified that the mandatory FFPE tumor sample is to be recently acquired, rather than newly acquired: Schedule of Assessments, Sections 5.1 and 8.8.1.

Other minor corrections were made throughout the document.

Version 9.0, 17 Jan 2023

The focus of this amendment was to incorporate language for the Post-Trial Access Program (PTAP), update the timing of the final analysis, and to incorporate changes from revised standard templates. The following sections were updated and the synopsis was also updated accordingly:

- Schedule of Activities updated to reflect PTAP procedures
- Schedule of Activities updated to remove PK sample type as corrected and clarified in Section 8.5.1.
- Schedule of Activities Table 4 and Table 5, footnote u, corrected follow-up time.
- Schedule of Activities Table 5 updated footnotes p and u with details of pregnancy test requirements and AESI follow-up for Dato-DXd, respectively. Visit window updated to [redacted] per PSC.
- Section 1.2 revised study period to present duration of study.
- Section 2 and Section 4.3 updated to align with IB updates for Dato-DXd and durvalumab and potential risks for Dato-DXd, durvalumab and capivasertib
- Section 2.3.5.2 updated with potential risks of Dato-DXd.
- Section 4.1.4.3 aligned with SRC charter.
- Section 4.4 updated to remove text relating to continued treatment after the end of the study as it is covered by updates to Section 6.6 and to include clinical trial transparency information.
- Section 5.2, Exclusion criteria 21 clarified that steroids are allowed as a premedication as an anti-emetic.

Version 9.0, 17 Jan 2023
• Section 6.1.1.2 amended to include IP monitoring details and additional administration details.<br><br>• Section 6.1.1.4 clarified prednisone premedication period and corrected bottle numbers of capivasertib.<br><br>• Section 6.1.1.7 revised recommendations for use of [redacted]<br><br>• Section 6.1.3 removed restriction that patients in the immunotherapy arm(s) are not be permitted to continue immunotherapy if progression occurs after confirmed response to immunotherapy treatment in the TLs, for standardization.<br><br>• Section 6.4 Immunosuppressive medications in Table 15 updated to allow for use as premedication for multiple reasons.<br><br>• Section 6.5.2.5 updated with minimum interval for two consecutive doses of T-DXd<br><br>• Section 6.5.2.6 revised to increase the time period for delay of dosing of Dato-DXd, followed by discontinuation of treatment. Figure and text to clarify dose reduction guidelines added.<br><br>• Section 6.6 updated text with PTAP wording to clarify continuation of treatment after the final DCO for the study.<br><br>• Sections 8 and 8.1 updated and 8.4.2 added to reflect PTAP procedures.<br><br>• Section 8.2.1 test for lactate dehydrogenase added.<br><br>• Section 8.2.3 clarified vital sign measurement requirements for Arm 7.<br><br>• Section 8.2.9.2 clarified that all events of ocular surface toxicity and keratitis should be [redacted].<br><br>• Section 8.3.14.5 Dato-DXd AESIs updated according to most recent IB and ocular surface toxicity assessment instructions clarified.<br><br>• Section 8.4.3 pregnancy guidelines updated.<br><br>• Section 8.4.6.6 and Appendix O; management of Dato-DXd toxicities updated according to most recent TMGs<br><br>• Section 8.5.2 storage and destruction of PK and ADA samples updated to within [redacted] of CSR finalization.<br><br>• Section 9.1 updated final analysis to 12 months from the date of first dose

Version 9.0, 17 Jan 2023

- Sections 9.4.3 and 9.4.4 corrected which arms collected PK data.
- Appendix A-1 updated to include reporting requirements for serious breaches.
- Appendix A-7 updated to clarify AstraZeneca oversight.
- Included Appendix R [redacted]

Other minor corrections were made throughout the document.

Version 8.0, 25 Apr 2022

Program-wide updates for Dato-DXd from the updated Investigator Brochure and safety strategy were the focus of this amendment. The following sections/tables were updated:

- Section 1.1 Table 5 (Schedule of Activities [SoA] Arm 7) updated [redacted] to include [redacted]
- Section 1.1 Table 5 (Schedule of Activities [SoA] Arm 7) updated with [redacted]
- Section 2.3.5. updated identified risks for Dato-DXd.
- Section 4.3.6 updated the summary of this information to provide a succinct justification for the Dato-DXd dose.
- Section 4.3.6 updated the mean terminal half-life (T½) of Dato-DXd.
- Section 6.1.1.7 updated section about Dato-DXd administration and monitoring of Dato-DXd administration, added [redacted]
- Section 6.5.2.6 updated Dato-DXd dose delays, dose reductions, dose interruption and modifications.
- Section 8.2.9.2 Clarifications on additional ophthalmologic assessments for patients on Arm 7 included as well as the requirement to fill out [redacted]
- Section 8.3.14.5 removed combined elevations of Aminotransferases and Total Bilirubin from the AESI list. Added separate AESIs of Oral mucositis/stomatitis and Mucosal inflammation other than oral mucositis/stomatitis. Added AESI of Ocular surface toxicity, requirement to report all ocular surface toxicity Grade ≥ 3

**Version 8.0, 25 Apr 2022**
within 24 hours of becoming aware and requirement to [redacted] in a timely manner.<br>• Section 8.4.5.6 updated.<br>• Appendix M Guidance for management of specific adverse events in studies of Dato-DXd (datopotamab deruxtecan) removed from CSP and provided as an annex.<br>• Appendix Q [redacted] added to CSP.<br>Other amendment changes include the following:<br>• Section 1.1 Table 5 (Schedule of Activities [SoA] Arm 7) and Table 6 (Schedule of assessments for patients who have completed/discontinued treatment) updated with additional scan following PD for Arm 7.<br>• Section 6.1.1.6 updated recommendation on antiemetics for DS-8201a.<br>• Section 6.5.2.5 updated the interruption period for DS-8201a due to adverse event up to [redacted] from the last DS-8201a dose for those patients who have confirmed continued benefit per RECIST 1.1, in line with TMGs for DS-8201a and Durvalumab who did not received any prohibited concomitant medication since last dose of DS-8201a. In addition, it was clarified that scheduled CT/MRI scans should continue as per SoA while drug is being interrupted.<br>• Appendix F Updated language for provision of BICR/Collect and Hold, inclusion of description on Central imaging.<br>The following sections/tables provide administrative changes to correct an omission or provide clarity throughout the document:<br>• Section 1.1 Clarified regarding glucose assessments after screening measurement in the Schedule of Activities [SoA] and Table 22<br>• Section 1.1 Table 5 (Schedule of Activities [SoA] Arm 7) updated footnote for ADA samples.<br>• Section 1.1 Table 6 updated with clarification on PK, ADA and immunogenicity samples.<br>• Section 5.1 and 8.8.1 updated with clarification that samples obtained from metastatic sites in bone are inadequate for testing and not acceptable<br>• Section 6.4 Table 15 updated exceptions for prohibited immunosuppressive medications.

Version 8.0, 25 Apr 2022

- Section 6.4.1 updated the medication list for close monitoring when administrated concomitantly with Dato-DXd
- Section 8.2.9.3 removed section about CCI [redacted]
- Section 8.5.1. updated to clarify requirements for sample collection.
- Section 9.2. Table 26 Summary of outcome variables and analysis populations updated with clarification on ADA-evaluable set
- Section 9.3.4. updated to clarify immunogeneicity analysis.

Other minor corrections were made throughout the document.

Version 7.0, 30 August 2021

Program-wide updates for Dato-DXd from the updated Investigator Brochure and Product Safety Requirements (PSR) and program-wide updates to DS-8201a (T-DXd) from the updated Project Specific Safety Requirement (PSSR) were the focus of this amendment. The following sections/tables were updated:

- Section 1.1 Table 4 (Schedule of Activities [SoAs] Arm 6) electrocardiograms (ECGs) after baseline would be done at Cycle 5 and every 4 cycles thereafter instead of Cycle 4 and every 4 cycles thereafter to align with the DS-8201PSSR: Table 5 (SoA Arm 7) ECG after baseline was updated to only be done if clinically indicated and at end of treatment (EOT) from more frequent ECGs schedule. In addition, it was clarified that ECG should be done in triplicate at screening and if an abnormality is noted to align with the Dato-Dxd PSR.
- Section 2.2.1, background on triple negative breast cancer (TNBC), was updated to include Dato-DXd Phase 1 safety and efficacy data (DS1062-A-J101) from the TNBC cohort through 8 January 2021 that was presented at European Society for Medical Oncology (ESMO).
- Section 2.2.9 incorporated updated safety and efficacy Dato-DXd data from the Investigator's Brochure Version 5.0, 08 February 2021, and presentation of the ESMO, Tropion-Pan Tumor 01 - TNBC Cohort (Phase 1, First-in-Human, Dose Escalation and Expansion Study), 2021. Section 4.3.6 updated the summary of this information to provide a succinct justification for the Dato-DXd dose.
- Section 2.3.4.2 updated program-wide potential risks of DS-8201with epistaxis, hepatic adverse events, skin adverse events, pneumonia, peripheral edema, pyrexia, dry eye, and dehydration as per updated DS-8201 PSSR.

Version 7.0, 30 August 2021
• Section 2.3.5.2 updated program-wide potential risks of Dato-DXd to include other identified risks of dry eye and potential risks of aspartate aminotransferase/alanine aminotransferase increased and constipation.<br><br>• Section 8.2.3 updated Arm 7 (Dato-DXd) subsequent infusions for blood pressure and pulse to be done before and after infusion at Day 1 of each subsequent cycle (after the first infusion) and EOT to align with the Dato-DXd PSR.<br><br>• Added Section 8.2.9.3 to discuss [redacted] for Dato-DXd and [redacted] of oral mucositis and stomatitis. Sections 6.1.1.7, 6.5.2.6, 8.3.14.5, 8.4.5.6, and Appendix M (Table 40) updated the prevention and/or toxicity management guidelines for stomatitis/mucosal inflammation. Section 1.1, Table 5 added an [redacted] at screening and as clinically indicated.<br><br>• Appendix L (Table 39) and Appendix M (Table 40) were updated to align the toxicity management guidelines in the updated DS-8201a PSSR and Dato-DXd PSR, respectively.<br><br>Other amendment changes include the following:<br><br>• Section 1.1 (SoAs Table 1 to Table 5), inclusion criteria No. 11, and Section 8.8.1 changed the archival time a tumor sample could be provided to [redacted] old (from ≤ 3 years) to improve the quality of the tissue for biomarker analyses. Also, Section 8.8.1 added that if a formalin-fixed and paraffin-embedded (FFPE) tissue block could not be provided, [redacted] freshly cut unstained serial tumor sections ([redacted] micron) are to be provided to also improve the quality of the analysis. It also clearly defines the mandatory sample when a biopsy is not expected. Minor clarifications were made to the analysis description.<br><br>• Section 6.1.1.4 clarified that when paclitaxel infusion is not administered, capivasertib can be interrupted or continued based on Investigator discretion instead of interrupting capivasertib. If capivasertib is continued on a weekly schedule, there should be 3 days off between capivasertib and subsequent paclitaxel administration.<br><br>• Section 6.1.1 5 was updated to include the physical appearance of oleclumab; changed should to must be kept in original packaging until use to prevent prolonged light exposure; changed should to must for the total time from needle puncture to start of administration and added that if the final product is stored at both refrigerated and ambient temperatures, the total time must not exceed 24 hours; changed should to must for the total infusion time not to exceed 4 hours. It changed the instructions for intravenous line flush to be according to local practices.<br><br>• Section 6.1.1.7 changed the total cumulative time from needle puncture of the vial to the end of administration to not exceed 4.5 hours at room temperature if there are interruptions during the infusion. Previously it was 4 hours. This was based on new

Version 7.0, 30 August 2021
microbial challenge data. Included the primary prophylaxis for stomatitis to align with the 17 June 2021 memo to sites.<br>• Appendix O was updated to align with the letter sent 11 May 2021 to Investigators regarding Guidance on COVID-19 Toxicity Management for Arm 6 and Arm 7.<br>The following sections/tables provide administrative changes to correct an omission or provide clarity throughout the document:<br>• Section 1.1 (SoAs) corrected the cross-reference section for pharmacokinetic (PK) sampling and assessment (Section 8.5) for accuracy. Table 5 clarified [redacted] footnote, pulmonary function test (PFT) and pulmonary HRCT at screening, and TSH assessment was added for each cycle and at EOT as it was omitted in error. Table 4 and Table 5 added that serum and plasma clinical chemistry, hematology, and coagulation assessments can be done up to 2 days prior to treatment administration for clarity.<br>• Section 1.1 SoA Table 1 to Table 5, the footnote for vital signs was updated to clarify that blood pressure and pulse are to be performed before, during, and after the first infusion as per Section 8.2.3. Table 1 to Table 4 also clarified that blood pressure and pulse are to be performed before each subsequent infusion as per Section 8.2.3. Table 5 clarified that vital signs are to be performed before and after infusion at Day 1 of each subsequent cycle and EOT as per Section 8.2.3.<br>• Sections 4.1.4.2.2 and 6.5.2.6 clarified the definition of Hy's Law to include total bilirubin (TBL) ≥ 2 × ULN from TBL > 2 × ULN.<br>• Section 4.2.2 clarified that Arm 7 (Dato-DXd) will also include plasma PK samples for Part 2 of the study. Previously only Part 1 was listed in error.<br>• Exclusion criteria No. 19 regarding live attenuated vaccines clarified that a COVID-19 vaccine that is a messenger ribonucleic acid and replication-deficient advenoviral are not considered to be a live vaccine.<br>• Section 6.1.3 added the requirement for a signature on the informed consent form addendum for treatment beyond progression.<br>• Section 6.4 Table 15 added the cross-reference link to Appendix O for patients on Arm 6 and Arm 7 on chloroquine and hydroxychloroquine for completeness.<br>• Sections 6.5.1 (Table 17) and 6.5.2.5 (Table 20) corrected the unit of dose of DS-8201a to mg/kg.<br>• Sections 6.5.2.5 and 6.5.2.6 (DS-8201a and Dato-DXd) clarified that a dose delay of up [redacted] from the last infusion date requires discontinuation only when an

Version 7.0, 30 August 2021

adverse event (AE) is causally related to study drug. It also clarified that DS-8201a or Dato-DXd can be given without durvalumab and vice versa.

- Sections 8.2.9.2 clarified [redacted] requirements for Arm 7 to be before and after each infusion at Day 1 for Cycle 1 to Cycle 3, prior to infusion from Cycle 4 onwards and at the EOT. Prior it was before each infusion.
- Section 8.5 clarified that PK should be assessed only in Part 1 for Arm 1 to Arm 6 and for Part 1 and Part 2 in Arm 7.
- Appendix O updated dose modification criteria for suspected or confirmed COVID-19 (Arm 6 and Arm 7) that was clarified in the Dear Investigator Letter of 11 May 2021.

Other minor corrections were made throughout the document

Version 6.0, 12 January 2021

A new study arm (Arm 7) Durvalumab + Dato-DXd (datopotamab deruxtecan; DS-1062a) was added to the protocol.

Reference to Arm 7 for Dato-DXd was incorporated throughout the protocol, as applicable, including Section 1.1 (addition of Table 5 [Schedule of Assessments for Arm 7]) and updates to Table 6, Section 1.2 Synopsis, Section 1.3 Schema, and Section 3.

Specifically, sections/tables were updated, or new sections were added to the following areas of the protocol:

- Section 2.2 was updated to include summary of the ASCENT study. Section 2.2.5 was updated to add the background and rationale for inclusion of Dato-DXd. A new Section 2.2.9 was added to include background information on Dato-DXd based on literature and preliminary results from Study DS1062-A-J101.
- Section 2.3 was updated to add a new Section 2.3.5 to include the potential benefits and risks associated with Dato-DXd based on preliminary monotherapy data from the ongoing DS1062-A-J101 study.
- Section 4.1.4.2 was updated to include the DLTs related to Dato-DXd for Arm 7 based on the DS1062-A-J101 study.
- Section 4.3 was updated to add a new Section 4.3.6 to include the rationale for the selected dose of Dato-DXd for this study.

**Version 6.0, 12 January 2021**
• Section 5.1 and 5.2 were updated to include drug-specific eligibility criteria for Arm 7.<br>• Section 6.1.1 was updated to add a new Section 6.1.1.7 for the description, preparation, and administration of Dato-DXd. Table 12 was updated to add treatment details for Arm 7.<br>• Section 6.1.2 was updated to add a new Section 6.1.2.6 for the dose and treatment regimen for Dato-DXd.<br>• Section 6.5 was updated to add a new Section 6.5.2.6 to include dose reductions, interruptions, and modifications management guidelines for Dato-DXd. Table 17 in Section 6.5.1 was updated with treatment information for Arm 7.<br>• Section 8.3.14 was updated to add a new Section 8.3.14.5 to include AESIs associated with Dato-DXd.<br>• Section 8.4.5 was updated to add a new Section 8.4.5.6 for management of Dato-DXd-related toxicities.<br>• Appendix M (Guidance regarding potential interactions of Dato-DXd (datopotamab deruxtecan) with concomitant medications) was added.<br>• Appendix O was updated to include instructions related to COVID-19 for Arm 7.
Estimated date of last patient completed (Part 1 and Part 2) was updated to Q1 2023 and the total patients (durvalumab + paclitaxel arm and 4 novel treatment combination arms) was updated to approximately 140 in Section 1.2.
The potential risks of capivasertib in Section 2.3.2 and Section 8.3.14.2 were updated to delete hyperlipidemia as an expected AESI to align with the current IB.
The potential risks of DS-8201a were updated in Section 2.3.4 to align with current available clinical data.
Section 4.1.1 and Appendix P were added to include study mitigation language that will provide sites with measures that may be implemented if a patient is not able to visit a study site to ensure that the clinical trial can continue while minimizing risk to the patient, maintaining compliance with GCP, and minimizing risks to the study integrity.
Inclusion criterion 6 in Section 5.1 was updated to include [redacted]

**Version 6.0, 12 January 2021**
[redacted]
Inclusion criterion 10 in Section 5.1 was updated to include serum albumin [redacted] × ULN if with documented [redacted] for patients in Arms 6 and 7.
Exclusion criteria 12, 23, 30, and 31 in Section 5.2 were updated to apply to Arm 2 only as these are drug-specific eligibility criteria.
Exclusion criterion 15 in Section 5.2 was updated to apply to Arm 5 only as this is a drug-specific eligibility criterion.
Exclusion criterion 35 in Section 5.2 "clinically significant corneal disease in the opinion of the Investigator" was added back, specifically for Arm 7.
Exclusion criteria 42 and 43 were added for Arms 6 and 7; Exclusion criterion 44 was added for Arm 6 in Section 5.2.
The preparation and administration of IP durvalumab in Section 6.1.1.2 was updated to align with the latest template text.
Section 6.1.1 Table 12 was updated to clarify the dosage formulation for DS-8201a. The preparation and administration of IP DS-8201a in Section 6.1.1.6 was updated to align with the latest template text.
Prohibited concomitant medications for Arm 2 in Section 6.4 and Appendix I were updated to allow careful use of 5-HT3 antagonists during study treatment in addition to other QT-prolonging medications.
Table 22 in Section 8.2.1 was updated to delete serum phosphorus and creatine phosphokinase as these tests are not applicable to the treatment arms currently in this study.
Section 8.2.3 was updated to clarify the times for BP and pulse collection following a [redacted]
Section 8.2.9.2 was updated to delete MRI of the chest and replace with "Pulmonary HRCT (or CT if HRCT is contraindicated) will be performed according to institutional guidelines" to align with the SoA.
Section 8.3.7 was updated to incorporate text from DS-8201a Project Specific Safety Requirements (PSSR): "Any diagnosis of the undesirable clinical outcome of 'left ventricular

Version 6.0, 12 January 2021
dysfunction', a valid or qualifying reduction of LVEF (as measured by MUGA or ECHO) should be confirmed and included in the AE report."
Section 8.8 was updated to specify [redacted] and additional IHC analyses will be performed on patient tumor samples. Exploratory [redacted] including additional drug target expression among other markers, in FFPE tumors will be evaluated by IHC. The tumor markers section was deleted as it contained duplicate information.
Section 8.2.9.1 and SoA Table 4 were updated to clarify that the HRCT requirements are as per institutional guidelines.
Appendix E was updated to align with the current template. "If, at any time (in consultation with the Study Monitor/Physician, the PHL case meets serious criteria, report it as an SAE using standard reporting procedures" was deleted and replaced by "Unless an alternate etiology for the elevated liver enzymes is known, report as an SAE using standard reporting procedures".
The haemotologic and pulmonary toxicity management guidelines for DS-8201a were updated in Appendix L to align with the PSSR.
Replaced the name of the system used for patient enrollment and randomization (IWRS/IVRS) with RTSM (IRT) throughout the protocol. This change was made for consistency across the company. There is no change to the system.
Various administrative changes were made throughout the protocol.

Version 5.0, 24 July 2020
Program-wide updates to DS-8201a (T-DXd) safety information were incorporated to align with the Project Specific Safety Requirement (PSSR) of DS-8201a. The following sections were updated:<br><br>• Schedule of activities Table 4 in Section 1.1 was updated to include the following:<br><br>– The frequency of 12-lead ECG was updated to be performed at screening, prior to administration of IMP at C1D1, and every fourth cycle (once) thereafter. Triplicate ECGs will be performed at screening. Subsequent ECGs will be performed in triplicate only if abnormalities are noted. If 12-lead ECG is abnormal, follow institutional guidelines

Version 5.0, 24 July 2020

  - Troponin-T will be measured at screening, EOT, and as needed based on patient-reported cardiac signs or symptoms suggesting congestive heart failure, myocardial infarction, or other causes of cardiac myocyte necrosis

  - Addition of blood sample timepoints for plasma exploratory clinical benefit or safety analyses (mandatory)

  - PFT was updated to include minimum requirement of FVC (L), FVC% predicted, FEV1 (L), FEV1% predicted, and FEV1/FVC%. Optional components to include: PEF, FEV6, TLC, DLCO

  - Plan to collect AEs and AESIs was updated

  - Instruction for the administration of DS-8201a was updated to include a recommendation that patients receive prophylactic anti-emetic agents prior to infusion of DS-8201a and on subsequent days

  - Pulmonary HRCT was updated to be performed, only if feasible, otherwise CT is acceptable

- Potential pulmonary toxicities considered to be associated with administration of DS-8201a were updated throughout the CSP to include ILD/pneumonitis as an important identified risk in Sections 2.3.4.2, 5.2, 8.3.14.4, and 8.4.5.5.

- Inclusion criterion 16 in Section 5.1 was updated to include partial thromboplastin time[redacted] × upper limit of normal (ULN).

- Section 5.2 was updated for the following:

  - Exclusion Criterion 35 was deleted, as cumulative review of clinical data demonstrated no further need to exclude patients with significant corneal disease.

  - Exclusion Criterion 38 was deleted, as it is redundant due to pleural effusion already captured in Exclusion Criterion 37. In addition, CART therapy is not used in breast cancer patients.

  - Exclusion criterion 40 was updated to redefine spinal cord compression or clinically active central nervous system metastases as untreated and symptomatic, or requiring therapy with corticosteroids or anticonvulsants to control associated symptoms

  - Exclusion criterion 41 was added for use of hydroxychloroquine in ≤14 days prior to Day 1 of DS-8201a treatment in patients with confirmed or suspected COVID-19 infections

Version 5.0, 24 July 2020

- Section 5.3 was updated for the following:
  - to clarify the continued use of methods of contraception for female patients of child-bearing potential to [redacted] after the last dose of DS-8201a
  - to clarify the continued use of method of contraception for male patients with a female partner of childbearing potential to [redacted] after the last dose of DS-8201a
  - Restriction related to use of tobacco products, e-cigarettes and vaping for patients enrolled in Arm 6 (durvalumab + DS-8201a) was added in Section 5.3
- Section 6.1 was updated to include the use of prophylactic anti-emetic agents prior to infusion of DS-8201a and on subsequent days based on currently available clinical safety data.
- Section 6.4 was updated to add chloroquine and hydroxychloroquine to the list of prohibited medicines for Arm 6 only
- Section 6.5.2.5 was updated to clarify that if a patient is assessed as requiring a DS-8201a dose delay of longer than 28 days, the drug will be discontinued.
- Section 8.2.3 was updated to clarify the infusion of DS-8210a will be a [redacted] infusion.
- Section 8.4.5.5 updated:
  - To clarify timelines for the measurement of troponin-T at screening, EOT, and as needed based on patient-reported cardiac signs or symptoms suggesting congestive heart failure, myocardial infarction, or other causes of cardiac myocyte necrosis
  - The frequency of 12-lead ECG was updated to be performed at screening, prior to administration of IMP at C1D1, and every fourth cycle (once) thereafter. Triplicate ECGs will be performed at screening. Subsequent ECGs will be performed in triplicate only if abnormalities are noted. If 12-lead ECG is abnormal, follow institutional guidelines
- Section 8.8.2 was updated to include a section on the exploratory biomarker plan for plasma samples for safety exploratory testing (for patients in Arm 6 only).
- Appendix L Toxicity management guidelines for DS-8201a (Table 40) was updated for clarity and to align with the PSSR.

Version 5.0, 24 July 2020

- Appendix O (Instructions related to COVID-19) was added to include:
  - Benefit-risk considerations, inclusion criteria, prior and concomitant medication, and dose modification criteria for patients with COVID-19 infections.
  - Blood sample timepoints for DS-8201a PK analysis in patients who received chloroquine/hydroxychloroquine

Changes were made to the study design. The study will use a CCI [redacted] test to evaluate which cohorts may proceed to expansion.

The following sections were updated:

- The SoAs were updated to clarify that the timing of imaging will be relative to the date of treatment assignment.
- Section 1.2 and 3 was updated to clarify that Part 1 and Part 2 data will be pooled for efficacy analysis.
- Section 1.2 and 3 was updated to clarify that all mentioned endpoints for Part 2 will be analyzed in the dataset of Part 1 plus Part 2 patients in each cohort and additionally may be analysed for each part separately.
- Sections 1.2, 3, 4.1, 4.2, 8.1, 9.1, 9.2.2, 9.4, and 9.5.2.2 were updated to reflect the change in the primary endpoint for Part 2 of the study from PFS to ORR.
- Sections 1.2, 1.3, 3, 4.2, and 9 were updated to redefine the efficacy endpoints (PFS6 and OS) as the time from the date of first dose, not from the date of treatment assignment/randomization.
- The schema for the study design in Section 1.3 was updated to represent the change in criteria for proceeding to Part 2 and to exclude two of the treatment arms from Part 2 as these were removed from the study prior to any patient enrollment.
- Sections 1.2, 4.1, and 4.2 were updated to include the rationale for the change in criteria for proceeding to Part 2.
- Section 1.2, 8.1 and 9 was updated to clarify that Part 2 will evaluate efficacy as the primary objective, by conducting a combined analysis of Part 1 and Part 2 data.
- Sections 1.2, 4.1.2, 6.2.1.1, and 9.1 were updated to change the number of patients enrolled in the study.

Version 5.0, 24 July 2020
• Sections 6.2.1 and 6.2.3 were updated to reflect that methods for enrollment and assignment to treatment arms for Part 1 and Part 2 are now the same based on this design change.<br>• Section 1.2 and 9 Statistical considerations was updated to include:<br>– A rationale for the sample size determination<br>– Clarification on the FAS being the same as the Safety Analysis Set<br>– Define a subset of the FAS as the Response Evaluable Analysis Set, the primary analysis set on which ORR analyses will be performed<br>– Statistical and sensitivity efficacy analyses will be conducted on combined Parts 1 and 2<br>– An ORR interim analysis will be evaluated on an ongoing basis for the first 30 evaluable patients within a treatment arm to complete Stage 1 of the [redacted]<br>– Results of ORR statistical analysis will be presented using 95% exact Clopper-Pearson CI<br>• Section 9 Statistical Considerations was updated:<br>– Text on exploratory analyses, which involved [redacted], was deleted.<br>– Timing of final analysis was included: The final analysis will be performed when the last patient has had the opportunity to be followed up for approximately 6 months from the date of first dose.<br>– Duplicated verbiage was deleted.
Male patients were excluded from Part 2 of the study to align the patient population between Part 1 and Part 2, with changes in Sections 4.2.4, 5.1, 5.3, and 8.4.2.2.
Inclusion criterion 5 in Section 5.1 was updated to clarify the type of patient and disease characteristic as advanced/unresectable or metastatic TNBC. This change was made to be consistent with the rest of the protocol and is not a change in the study population.
Section 6.1 was updated to remove reference to capivasertib 80 mg film-coated tablets as these are no longer in clinical use.

**Version 5.0, 24 July 2020**
The AESIs for capivasertib were updated in Sections 2.3.2.3 and 8.3.14.2 to include hyperlipidemia, infection/lower respiratory tract infection, and [redacted] to align with the capivasertib AESI standards. The following AESIs were deleted: hypersensitivity, dry skin, pruritus, decreased appetite, nausea, and vomiting.
Section 8.4.5.5 was updated to clarify that for DS-8201a, [redacted] will be established to [redacted]. To ensure adequate and relevant evaluation, additional data may be collected to fully characterize medical history (eg, smoking, radiation and pulmonary history), diagnostic evaluation, treatment, and outcome of the event.
Section 8.5 was updated to remove erroneous text regarding blinding, as this is an open-label study.
Section 8.5.2 was updated to clarify that text regarding storage and destruction of residual backup PK samples also applies to ADA samples.
Various administrative changes were made throughout the protocol.

**Version 4.0, 20 December 2019**
Potential risks of durvalumab were updated in Section 2.3.1.2 based on the durvalumab Investigator's Brochure Version 15.0.
Durvalumab + DS-8201a arm (Arm 6) was added to the protocol. This change was made to the following areas of the protocol: Sections 1.1 (including addition of Table 5 [Schedule of Assessments for Arm 6]), 1.2, 4.1, 4.1.1, 4.1.3, 4.3.1, 5.1, 5.2, 6.1.1, 6.1.1.2, 6.2.1, 6.4, 6.5.1, 7.1.1, 8.2.9, 8.3.8, 8.8.1, and Appendix N.
The durvalumab + paclitaxel + danvatirsen arm (Arm 4) has been removed from the protocol. This change was made to the following areas of the protocol: Sections 1.1 (Table 4 [Schedule of Assessments for Arm 4], 1.2, 2.2.7, 2.2.7.1, 2.3.3.1, 2.3.3.2, 3, 4.3.4, 4.3.4.1, 4.3.4.2, 4.3.4.3, 6, 6.1.1, 6.1.1.5, 6.1.2, 6.1.2.4 (the Original Figure 4 was removed), 6.5.1, 6.5.2.4, 8.2.1 (Tables 21 and 23), 8.4.5, 8.4.5.4, 8.5.1.1, 8.5.1.2, and 9.3.
The durvalumab + paclitaxel + capivasertib arm (Arm 2) has been updated to reduce paclitaxel dosing to 80 mg/m^2 to be aligned with the Phase III CAPItello-290 protocol (NCT 03997123) (Sections 1.2, 4.3.2, 6.1.1, 6.1.1.3, 6.1.2.3, and 6.5.1).

**Version 4.0, 20 December 2019**
Program-wide updates to capivasertib safety and background information were incorporated in Sections 1.1, 2.2.6, 2.3.2.3, 2.3.2.4, 5.2, 6.1.1, 6.5.2.3, 8.3.14.2, 8.4.5.3, and 10, and in Appendix I and Appendix K.
Program-wide updates to durvalumab safety information were incorporated in Sections 2.3.1.2.2 and 8.3.14.1.
Sections 1.2, 4.1.3.1, and 6.5.1 were updated to clarify that any decisions to introduce additional cohorts in order to investigate an alternative dosing regimen will be at the discretion of AstraZeneca after discussion with the SRC.
Sections 1.2 and 9.6 were updated to clarify that the SRC will also review safety and tolerability data on an ongoing basis.
Section 2.2.6.1 was updated with a reference to the CAPItello-290 trial (NCT 03997123).
Section 4.1.3.1 was updated to allow for dose evaluation in a rolling 6-patient design if either the novel agent or paclitaxel were dose reduced for the cohort.
Section 4.1.3.2 was updated to change DLT-evaluable status to require [redacted] of the prescribed number of doses to allow for clinical flexibility for interruptions following toxicity management.
Dose-limiting toxicity criteria related to fibrinogen was removed from Section 4.1.3.2 since the durvalumab + paclitaxel + danvatirsen arm (Arm 4) has been removed from the protocol. DLT criteria related to left ventricular ejection fraction was added to Section 4.1.3.2 for durvalumab + DS-8201a arm (Arm 6).
Inclusion Criterion 5 in Section 5.1 was updated to reflect TNBC diagnosis based on the most current guidelines. Appendices H and I, which presented now outdated guidelines, were removed.
Exclusion Criterion 13 in Section 5.2 was updated to also exclude troponin levels consistent with myocardial infarction and stroke, consistent with program-wide DS-8201a safety information.
Cardiac ejection fraction outside institutional range of normal or <50% (whichever is higher) was added as an exclusion criterion in Section 5.2 for all treatment arms consistent with updated safety information for both capivasertib and DS-8201a.

**Version 4.0, 20 December 2019**
Section 5.3 was updated with restrictions on ova use, consistent with program-wide DS-8201a safety information.
Administration instructions were updated for capivasertib (AZD5363) in Section 6.1.1.4.
Management of oleclumab toxicity was updated in Section 6.1.1.6.
Text was added to Section 6.2.1 to clarify the process for treatment arm assignment in Part 2 that is already in place (ie, that patients who enter Part 2 will be randomized to treatment wherever possible).
List of prohibited medications was updated in Section 6.4, Table 15 related to capivasertib.
In Section 6.5.2.2, Table 18 (Recommended Paclitaxel Dose Reduction) was removed to ensure alignment with the CAPItello-290 trial (NCT 03997123) and alignment with global differences in local standard of care.
Clarification was provided regarding requirements for the FFPE sample in Sections 5.1 and 8.8.1.
Section 8.3.14 (Adverse events of special interest) and Section 8.4.5.1 (Specific toxicity management and dose modification information for durvalumab and durvalumab in combination with other therapies) were updated based on the amendment to the durvalumab Investigator's Brochure Version 15.0.
Appendix E (Actions required in cases of increases in liver biochemistry and evaluation of Hy's law) was updated based on Hy's Law Version 3.
Appendix M (Guidance regarding potential interactions of capivasertib with concomitant medications) was updated added Table 42 (MATE1 and/or OCT2 transporter substrates that should be used with caution).
Appendix L (Guidance regarding potential interactions of DS-8201a [trastuzumab deruxtecan] with concomitant medications) was added.
Various administrative changes were made throughout the protocol.

Version 3.0, 25 April 2019
A change of tablet formulation for capivasertib has been added to the protocol. A new 160 mg film-coated tablet has been added for dose reduction purposes to Section 6.1.1.4, Table 12 and Table 13.
The durvalumab + paclitaxel + selumetinib arm (Arm 3) has been removed from the protocol. This change was made to the following areas of the protocol: Table 3 (Schedule of Assessments for Arm 3), Footnote "i" in Table 5, Table 6, and Original Figure 4.
All mentions of selumetinib have been removed from the protocol. This change was made to the following areas of the protocol: Sections 1.1, 2.2.6, 2.3.2, 4.3.3, 6.1.1, 6.1.2, 6.1.4, 6.3.2, 6.4, 6.5.1, 6.5.2, 8.2.5, 8.2.7, 8.3.13, 8.4.5, and 8.5.1, 9.3; and Appendix L.
Each triplet cohort combination arm (Arms 2, 4, and 5) has been enlarged from 20 to 30 patients. This change was made to the following areas of the protocol: Sections 4.1, 4.1.1, 4.1.3.1, 6.2.1.1, 9.2.1, and 9.6.
12-lead ECG assessments, at C1D1, C2D1, and every 12 weeks as clinically indicated, from Cycle 3 to End of Treatment, were added to Table 2 (Arm 2) of the Schedule of Assessments.
Efficacy analyses were updated to include the exploration of [redacted] status (FAS set) in the following areas of the protocol: Section 9.5.2.1, and Table 29.
Study termination language was updated in Sections 4.5 and 7.1.
Various administrative changes were made in the Schedule of Assessments and Tables 1 to 5 and throughout the protocol.
Exclusion criteria in Section 5.2 were modified; Criterion 22 was removed.

Version 2.0, 4 February 2019
The title page was updated with the EudraCT number.
Table 2 glycosylated hemoglobin (HbA1c) assessment timepoint was updated to read "C4D1 and q12w thereafter."
Tables 1 through 6 and Sections 1.2, 6.1.3, and 7.1.1 were updated with revised text on Response Evaluation Criteria in Solid Tumors (RECIST) assessments.

**Version 2.0, 4 February 2019**
Tables 1 through 6 were updated to include references to Appendix F in the Tumor assessment (RECIST 1.1) rows.
Tables 1 through 5 were updated to clarify that complete blood count with differential may be checked on the day of paclitaxel administration or 1 day prior to paclitaxel administration.
Tables 1 through 5 were updated to clarify that paclitaxel doses can have a ±1-day dosing window, except at Cycle 1 Day 1.
Table 2 was updated to specify that the optional clinical care tumor sample can be obtained at any point during the study.
Table 3, Arm 3 row for MUGA scan or ECHO was updated to include assessments at the end of Cycle 1 and q12w thereafter. Text was updated in the corresponding footnote "f" and in Section 8.2.5 to reflect this change.
Table 4 was updated to reflect coagulation assessment once per cycle until progression of disease (PD), except in Cycle 1.
Table 4 was updated to include additional guidance on danvatirsen administration.
The original Table 4 was updated to move the danvatirsen immunogenicity assessment to Cycle 4.
Table 4 electrocardiogram (ECG) and pharmacokinetic (PK) footnotes were updated to clarify assessment timepoints and to align ECG wording with PK wording.
Table 5 was updated to include additional guidance on oleclumab administration.
Table 5 and Section 6.1.1.7 were updated to specify that durvalumab can be given no less than [redacted] after the infusion of oleclumab has finished.
Tables 1 through 5 were updated to clarify the timing of ECG assessments; the timing of these assessments remains unchanged.
Sections 1.2, 1.3, and 4.1.2 were modified to include ORR and paclitaxel exposure in the decision to initiate Part 2 of the study.
Sections 1.2 and 9.5.2.1 (Table 32) were updated to include both the 80% and 95% Clopper-Pearson CIs.

**Version 2.0, 4 February 2019**
Section 1.3 was updated to include at least 150 patients in the study design schema. The term "evaluable" was removed from Part 2 of the study design schema.
Section 2.2.1.1 was added to describe BRCA mutations in triple negative breast cancer (TNBC). Section 9.5.2.2 was also updated to reflect the addition of BRCA1/2 mutation.
Section 2.2.2 was modified to describe the IMPassion 130 study and its significant results for PFS data.
Section 2.2.3 was modified to specify that the IMPassion 131 study is ongoing at the time of this protocol amendment.
Sections 2.2.7.1 and 2.3.3.4 were updated to reflect that the SELECT-4 Phase I/II study has completed enrollment.
Section 4.1 was modified to change [redacted] old" to [redacted] old."
Sections 4.1, 4.2.3, 6.1.2, and 7.1 were updated to change "confirmed radiological progression" to "radiological progression."
Section 4.1.3.2 was modified to adjust the definition of a dose-limiting toxicity to include a new list of severe AEs. Text was added to clarify that any clinically insignificant electrolyte abnormality AE that resolves within 72 hours with appropriate medical management will not be considered a DLT.
Section 4.2.1 was modified to clarify that changes in paclitaxel dosing, including dose delays, reductions, and discontinuations, will be included in the evaluation of safety of the doublet and triplet combinations.
Section 4.2.4 was added to justify the exclusion of male patients in Part 1 of this study.
Sections 4.5 and 7.1 were updated to state that information from new emerging safety data from this study or another study can close any arm prematurely.
Section 5.1 was modified to remove the previous Inclusion Criterion 8 (life expectancy of at least 12 weeks).
Section 5.2 was modified to remove neuropathy criteria from Exclusion Criterion 8 and to add Exclusion Criterion 33 (patients with baseline peripheral neuropathy Grade >1).

Version 2.0, 4 February 2019
Section 5.2 was modified to change the time limitation of Exclusion Criterion 16 (history of venous thromboembolism) from the past 6 months to the past 3 months.
Section 5.2 was updated to remove exclusion of radiotherapy (Exclusion Criterion 18).
Sections 5.1 and 5.2 were modified so that the numbering of the inclusion and exclusion criteria reflects the removal of an inclusion criterion and the addition of an exclusion criterion. Section 6.1.3 was updated to reflect the changes in Sections 5.1 and 5.2.
Section 5.2, Exclusion Criterion 18 was modified to include prior exposure to anti-CD73, anti-CD39, or adenosine receptor inhibitors.
Section 5.2, Exclusion Criterion 31 was modified to define abnormalities of glucose metabolism.
Section 6 was modified to specify the order of investigational product drug administration.
Sections 6.1.1, 6.1.1.4, and 6.3.2 were modified to replace the term “capsule” with “tablet” when used to describe capivasertib.
Section 6.1.1.3 text was modified to describe a meta-analysis of randomized, controlled studies in advanced breast cancer using paclitaxel.
Section 6.1.1.3 was modified to add local SoC to the method of administration for paclitaxel premedication.
Section 6.1.1.4 was adjusted so that all mentions of 160 mg capivasertib tablets have been removed. Only 200 mg and 80 mg tablets remain in the protocol.
Table 14 was modified to clarify that 4 tablets at 80 mg will be administered bid for Dose -1 and will be supplied in two 60-count bottles.
Section 6.1.1.6 was updated to clarify that danvatirsen administration at the initial dose (dose level 1) and in the event of de-escalation (dose level -1) during the safety run-in period is summarized in Table 19.
Section 6.1.1.7 was updated to clarify that oleclumab administration at the initial dose (dose level 1) and in the event of de-escalation (dose level -1) during the safety run-in period is summarized in Table 19.

**Version 2.0, 4 February 2019**
Section 6.1.2.1 was updated to specify that if there are no clinically significant infusion reactions within the first cycle, and at the discretion of the Investigator, then for all other cycles, paclitaxel can be given immediately after the infusion of durvalumab has finished.
Sections 6.1.2.2 and 7.1.1 were updated to specify that in the event that paclitaxel is discontinued, durvalumab and/or the novel oncology therapy can be continued as scheduled at the Investigator's discretion.
Table 17 was updated with a row for the capivasertib arm only: Medications that may be QT prolonging. Appendix K was updated to reflect this change. Two rows were also added for sensitive drugs or herbal supplements that are known to be potent inhibitors or inducers of cytochrome P450 (CYP)3A4 and for drugs known to be sensitive to CYP3A4, CYP2C9, and/or CYP2D6 metabolism inhibition and/or have a narrow therapeutic window.
Table 18 was updated to describe the potential result of high doses of vitamin E.
Table 22 was added to Section 6.5.2.3 to describe dose modifications for general capivasertib-related toxicities.
Sections 6.5.2.3, 6.5.2.4, 6.5.2.5, and 6.5.2.6 were updated to describe the risk or severity of overlapping toxicities due to the combination of durvalumab and paclitaxel with any one of the following: Capivasertib, selumetinib, danvatirsen, or oleclumab.
Table 27 was updated to include serum phosphorus and creatine kinase.
Sections 4.5 and 7.1 were updated to include text that reads "As this is a multi-arm study, information from new emerging safety data from this study or another study can close any arm prematurely".
Section 8.1 was modified to remove text for consistency with the updated RECIST appendix.
Section 8.4.5 was modified to specify that durvalumab, oleclumab, and danvatirsen are non-cytotoxic agents and are generally classified as non-irritants, although experience is limited with their extravasion.
Section 8.4.5.3 was modified to describe metformin as the currently preferred oral antidiabetic recommended for the management of hyperglycemia occurring in patients participating in studies of capivasertib. Appendix K was updated to reflect this change.
Table 32 was updated to include a footnote specifying that exposure includes durvalumab, paclitaxel, capivasertib, selumetinib, danvatirsen, and oleclumab dose delays, reductions,

**Version 2.0, 4 February 2019**
and discontinuations. All subsequent footnotes were updated accordingly, from “b” to “d.” Section 9.5.1.1 was also updated to reflect this change.
References were added for Mauri et al 2010, Robson et al 2017, and Schmid et al 2018b.
Appendix F was updated to include the new RECIST 1.1 appendix.
Appendix J (Dose modification and toxicity management guidelines for immune-mediated, infusion related, and non-immune-mediated reactions [durvalumab and oleclumab]) was removed, and text references were updated with links to this content in a separate document, consistent with the new program-wide convention.
Original Appendix M (Amendment 2), Guidance regarding potential interactions of capivasertib with concomitant medications, has been updated to reflect text in the d3614c00001 Clinical Study Protocol.

**Version 1.0, 25 July 2018**
Initial creation.
