# Supplementary material for: First-line durvalumab in combination with trastuzumab deruxtecan in women with locally advanced unresectable or metastatic, hormone-receptor-negative, HER2-low breast cancer: multicenter, open-label, phase 1b/2 BEGONIA platform trial
Source: Nat Cancer. 2026 Jun 8;7(6):983–92. doi: 10.1038/s43018-026-01181-8 (PMC13309285; doi:10.1038/s43018-026-01181-8)
Supplement: Supplementary file 5 — Supplementary Tables 1–3. [file 43018_2026_1181_MOESM5_ESM.pdf]

Supplementary Table 1. Efficacy outcomes by local HER2 status

Outcome	Durvalumab + T-DXd (*N* = 58)
**Confirmed ORR, *n/N* (%; 95% CI)**	
IHC 1+/ISH untested and IHC 1+/ISH-	27/38 (71.1; 54.1–84.6)
IHC2+/ISH-	9/20 (45.0; 23.1–68.5)
**Median DoR (95% CI), months**	
IHC 1+/ISH untested and IHC 1+/ISH-	14.4 (6.8–23.3)
IHC2+/ISH-	NC (7.3–NC)
**Median PFS (95% CI), months**	
IHC 1+/ISH untested and IHC 1+/ISH-	11.0 (8.3–17.0)
IHC2+/ISH-	13.7 (6.3–NC)
**Median OS (95% CI), months**	
IHC 1+/ISH untested and IHC 1+/ISH-	33.5 (18.8–NC)
IHC2+/ISH-	27.6 (6.3–NC)

CI, confidence interval; DoR, duration of response; NC, not calculable; OS, overall survival; PFS, progression-free survival; T-DXd, trastuzumab deruxtecan.

Supplementary Table 2. Adverse events related to durvalumab in ≥10% of patients

AE by preferred term, *n* (%)	Durvalumab + T-DXd (*N* = 58)
**Any AE related to durvalumab**	**49 (84.5)**
Fatigue	16 (27.6)
Nausea	14 (24.1)
Hypothyroidism	12 (20.7)
Alanine aminotransferase increased	7 (12.1)
Aspartate aminotransferase increased	7 (12.1)
Decreased appetite	7 (12.1)
Diarrhea	7 (12.1)
Hyperthyroidism	7 (12.1)
Pneumonitis	7 (12.1)
Neutropenia	6 (10.3)
Vomiting	6 (10.3)

AE, adverse event; T-DXd, trastuzumab deruxtecan.

Supplementary Table 3. Adverse events related to T-DXd in ≥10% of patients

AE by preferred term, *n* (%)	Durvalumab + T-DXd (*N* = 58)
**Any AE related to T-DXd**	**56 (96.6)**
Nausea	41 (70.7)
Fatigue	28 (48.3)
Neutropenia	19 (32.8)
Vomiting	14 (24.1)
Alopecia	14 (24.1)
Anemia	13 (22.4)
Decreased appetite	12 (20.7)
Asthenia	9 (15.5)
Thrombocytopenia	8 (13.8)
Pneumonitis	8 (13.8)
Diarrhea	8 (13.8)
Alanine aminotransferase increased	8 (13.8)
Aspartate aminotransferase increased	7 (12.1)
Constipation	7 (12.1)
Stomatitis	6 (10.3)

AE, adverse event; T-DXd, trastuzumab deruxtecan.
